# Supplementary material for: predPhogly-Site: Predicting phosphoglycerylation sites by incorporating probabilistic sequence-coupling information into PseAAC and addressing data imbalance
Source: PLoS One. 2021 Apr 1;16(4):e0249396. doi: 10.1371/journal.pone.0249396 (PMC8016359; doi:10.1371/journal.pone.0249396)
Supplement: S1 File — The phosphoglycerylated proteins as well as the segmented sequences with respective protein ID and positions have been provided. (PDF) [file pone.0249396.s001.pdf]

Proteins in the Benchmark Dataset:

>sp|A2A5N1|Modified_Residue:51
MTMDKSELVQKAKLAEQAERYDDMAAAMKAVTEQGHELSNEERNLLSVAYKNVVGARRSSWRVISSIEQK
TERNEKKQQMGKEYREKIEAELQDICNDVLELLDKYLILNATQAESKVFYLKMKGDYFRYLSEVASGENK
QTTVSNSQQAYQEAFEISK

>sp|A2AQC3|Modified_Residue:190
MVRPCPSVGPRGRLRAWPGARDLAPALRARPARCRRLLPLPRGGAEAAGSAGGAAGGDMTDSIPLQPVRH
KKRVDSRPRAGCCEWLRCCGGGEPRPRTVWLGHPEKRDQRYPRNVINNQKYNFFTFLPGVLFSQFRYFFN
FYFLLLACSQFVPEMRLGALYTYWVPLGFVLAVTIIREAVEEIRCYVRDKEMNSQVYSRLTSRGTVKVKS
SNIQVGDLILVEKNQRVPADMIFLRTSEKNGSCFLRTDQLDGETDWKLRLPVACTQRLPTAADLLQIRSY
VYAEEPNIDIHNFLGTFTREDSDPPISESLSIENTLWAGTVIASGTVVGVVLYTGRELRSVMNTSDPRSK
IGLFDLEVNCLTKILFGALVVVSLVMVALQHFAGRWYLQIIRFLLLFSNIIPISLRVNLDMGKIVYSWVI
RRDSKIPGTVVRSSTIPEQLGRISYLLTDKTGTLTQNEMVFKRLHLGTVAYGLDSMDEVQSHIFSIYTQQ
SQDPPAQKGPTVTTKVRRTMSSRVHEAVKAIALCHNVTPVYESNGVTDQAEAEKQFEDSCRVYQASSPDE
VALVQWTESVGLTLVGRDQSSMQLRTPGDQVLNLTILQVFPFTYESKRMGIIVRDESTGEITFYMKGADV
VMAGIVQYNDWLEEECGNMAREGLRVLVVAKKSLTEEQYQDFEARYVQAKLSVHDRSLKVATVIESLEME
MELLCLTGVEDQLQADVRPTLETLRNAGIKVWMLTGDKLETATCTAKNAHLVTRNQDIHVFRLVTNRGEA
HLELNAFRRKHDCALVISGDSLEVCLKYYEYEFMELACQCPAVVCCRCAPTQKAQIVRLLQERTGKLTCA
VGDGGNDVSMIQESDCGVGVEGKEGKQASLAADFSITQFKHLGRLLMVHGRNSYKRSAALSQFVIHRSLC
ISTMQAVFSSVFYFASVPLYQGFLIIGYSTIYTMFPVFSLVLDKDVKSEVAMLYPELYKDLLKGRPLSYK
TFLIWVLISIYQGSTIMYGALLLFESEFVHIVAISFTSLILTELLMVALTIQTWHWLMTVAELLSLACYI
ASLVFLHEFIDVYFIATLSFLWKVSVITLVSCLPLYVLKYLRRRFSPPSYSKLTS

>sp|A6ZI44|Modified_Residue:254
MATRRPDGSSFNMTRLSLALAFSFPPVASEQPHSELGNTQQQTELGKESTATGTMPHPYPALTPEQKKEL
SDIAHRIVAPGKGILAADESTGSIAKRLQSIGTENTEENRRFYRQLLLTADDRVNPCIGGVILFHETLYQ
KADDGRPFPQVIKSKGGVVGIKVDKGVVPLAGTNGETTTQGLDGLSERCAQYKKDGADFAKWRCVLKIGE
HTPSALAIMENANVLARYASICQQNGIVPIVEPEILPDGDHDLKRCQYVTEKVLAAVYKALSDHHVYLEG
TLLKPNMVTPGHACTQKFSNEEIAMATVTALRRTVPPAVTGVTFLSGGQSEEEASINLNAINKCPLLKPW
ALTFSYGRALQASALKAWGGKKENLKAAQEEYIKRALANSLACQGKYTPSGQSGAAASESLFISNHAY

>sp|A8DUK4|Modified_Residue:18,83,96,145
MVHLTDAEKAAVSGLWGKVNADEVGGEALGRLLVVYPWTQRYFDSFGDLSSASAIMGNAKVKAHGKKVIT
AFNDGLNHLDSLKGTFASLSELHCDKLHVDPENFRLLGNMIVIVLGHHLGKDFTPAAQAAFQKVVAGVAA
ALAHKYH

>sp|B1AS29|Modified_Residue:367
MTAPWRRLRSLVWEYWAGFLVCAFWIPDSRGMPHVIRIGGIFEYADGPNAQVMNAEEHAFRFSANIINRN
RTLLPNTTLTYDIQRIHFHDSFEATKKACDQLALGVVAIFGPSQGSCTNAVQSICNALEVPHIQLRWKHH
PLDNKDTFYVNLYPDYASLSHAILDLVQSLKWRSATVVYDDSTGLIRLQELIMAPSRYNIRLKIRQLPID
SDDSRPLLKEMKRGREFRIIFDCSHTMAAQILKQAMAMGMMTEYYHFIFTTLDLYALDLEPYRYSGVNLT
GFRILNVDNPHVSAIVEKWAMERLQAAPRAESGLLDGVMMTDAALLYDAVHIVSVCYQRAPQMTVNSLQC
HRHKAWRFGGRFMNFIKEAQWEGLTGRIVFNKTSGLRTDFDLDIISLKEDGLEKVGVWSPADGLNITEVA
KGRGPNVTDSLTNRSLIVTTVLEEPFVMFRKSDRTLYGNDRFEGYCIDLLKELAHILGFSYEIRLVEDGK
YGAQDDKGQWNGMVKELIDHKADLAVAPLTITHVREKAIDFSKPFMTLGVSILYRKPNGTNPSVFSFLNP
LSPDIWMYVLLAYLGVSCVLFVIARFSPYEWYDAHPCNPGSEVVENNFTLLNSFWFGMGSLMQQGSELMP
KALSTRIIGGIWWFFTLIIISSYTANLAAFLTVERMESPIDSADDLAKQTKIEYGAVKDGATMTFFKKSK
ISTFEKMWAFMSSKPSALVKNNEEGIQRTLTADYALLMESTTIEYITQRNCNLTQIGGLIDSKGYGIGTP
MGSPYRDKITIAILQLQEEDKLHIMKEKWWRGSGCPEEENKEASALGIQKIGGIFIVLAAGLVLSVLVAV
GEFIYKLRKTAEREQRSFCSTVADEIRFSLTCQRRLKHKPQPPMMVKTDAVINMHTFNDRRLPGKDSMSC
STSLAPVFP

>sp|B1AU42|Modified_Residue:104
MEMYETLGKVGEGSYGTVMKCKHKDTGRIVAIKIFYEKPEKSVNKIATREIKFLKQFRHENLVNLIEVFR
QKKKIHLVFEFIDHTVLDELQHYCHGLESKRLRKYLFQILRAIEYLHNNNIIHRDIKPENILVSQSGITK
LCDFGFARTLAAPGDVYTDYVATRWYRAPELVLKDTSYGKPVDIWALGCMII

>sp|B4DPF6|Modified_Residue:208
MRIVYRMRGLLGDATEEFIESLDSTTDEEEDEEEVYKMAGVMAQCGGLECMLNRLAGIRDFKQGRHLLTV
LLKLFSYCVKVKVNRQQLVKLEMNTLNVMLGTLNLGNLLLTGDKDQLVMLLDQINSTFVRSNPSVLQGLL
RIIPYLSFGEVEKMQILVERFKPYCNFDKYDEDHSGDDKVFLDCFCKIAAGIKNNSNGHQLKDLILQKGI
TQNALDYMKKHIPSAKNLDADIWKKFLSRPALPFILRLLRGLAIQHPGTQVLIGTDSIPNLHKLEQVSSD
EGIGTLAENLLEALREHPDVNKKIDAARRETRAEKKRMAMAMRQKALGTLGMTTNEKGQVVTKTALLKQM
EELIEEPGLTCCICREGYKFQPTKVLGIYTFTKRVALEEMENKPRKQQGYSTVSHFNIVHYDCHLAAVRL
ARGREEWESAALQNANTKCNGLLPVWGPHVPESAFATCLARHNTYLQECTGQREPTYQLNIHDIKLLFLR
FAMEQSFSADTGGGGRESNIHLIPYIIHTVLYVLNTTRATSREEKNLQGFLEQPKEKWVESAFEVDGPYY
FTVLALHILPPEQWRATRVEILRRLLVTSQARAVAPGGATRLTDKAVKDYSAYRSSLLFWALVDLIYNMF
KKVPTSNTEGGWSCSLAEYIRHNDMPIYEAADKALKTFQEEFMPVETFSEFLDVAGLLSEITDPESFLKD
LLNSVP

>sp|B8ZZL8|Modified_Residue:56
MAGQAFRKFLPLFDRVLVERSAAETVTKGGIMLPEKSQGKVLQATVVAVGSGSKGKGGEIQPVSVKVGDK
VLLPEYGGTKVVLDDKVCKLNNSKKKSDICN

>sp|C9J5S8|Modified_Residue:23
METQADLVSQEPQALLDSALPSKVPAFSDKDSLGDEMLAAALLKAKSQELVTFEDVAVYFIRKEWKRLEP
AQRDLYRDVMLENYGNVFSLDRETRTENDQEISEDTRSHGVLLGRFQKDISQGLKFKEAYEREVSLKRPL
GNSPGERLNRKMPDFGQVTVEEKLTPRGERSEKYNDFGNSFTVNS

>sp|D3YU05|Modified_Residue:149,219,223,231,267
MVKVGVNGFGRIGRLVTRAAVCSGKISVEIVAINDPFIDLNYMVYLFQSDSTHGKFNRTVQAENGKLVIN
GKPITIFQERDTPPPLANIKWGDAGADYVVESTGVFTTMEKAGAHLKGGAKRVIISAPSADAPMFVMGVN
HEKYDNSLKIVSNASCTTNCLAPLAKVIHDNFGIMEGLMTTVHAITATQKTVDGPSGKLWRDGRGAAQNI
IPASTGAAKAVGKVIPELNGKLTGMAFRVPTRNVSVVDLTCRLEKHAKYDDIKKVVKQASEGPLKGILGY
TEDQVVSCDFNNNSHSSTFDAGAGIALNDNFVKLISWYDNEYGYSNRMVDLMAYMASKE

>sp|D3Z041|Modified_Residue:544,552
MEVHELFRYFRMPELIDIRQYVRTLPTNTLMGFGAFAALTTFWYATRPKALKPPCDLSMQSVEIAGTTDG
IRRSAVLEDDKLLVYYYDDVRTMYDGFQRGIQVSNNGPCLGSRKPNQPYEWISYKEVAELAECIGSGLIQ
KGFKPCSEQFIGLFSQNRPEWVIVEQGCFSYSMVVVPLYDTLGADAITYIVNKAELSVIFADKPEKAKLL
LEGVENKLTPCLKIIVIMDSYGSDLVERGKKCGVEIISLKALEDLGRVNRVKPKPPEPEDLAIICFTSGT
TGNPKGAMITHQNIINDCSGFIKATESALTLNASDTQISYLPLAHMYEQQLQCVMLCHGAKIGFFQGDIR
LLMDDLKVLQPTIFPVVPRLLNRMFDRIFGQANTSLKRWLLDFASKRKEAELRSGIVRNNSLWDKLIFHK
IQSSLGGKVRLMITGAAPVSATVLTFLRTALGCQFYEGYGQTECTAGCCLSLPGDWTAGHVGAPMPCNYV
KLVDVEEMNYLASKGEGEVCVKGANVFKGYLKDPARTAEALDKDGWLHTGDIGKWLPNGTLKIIDRKKHI
FKLAQGEYIAPEKIENIYLRSEAVAQVFVHGESLQAFLIAVVVPDVESLPSWAQKRGLQGSFEELCRNKD
INKAILDDLLKLGKEAGLKPFEQVKGIAVHPELFSIDNGLLTPTLKAKRPELRNYFRSQIDELYATIKI

>sp|D3Z563|Modified_Residue:31
MLFIYSHFKQATVGDVNTDRPGLLDLKGKAKWDSWNKLKGTSKESAMKTYVEKVDELKKKYGI

>sp|D3Z6C3|Modified_Residue:144
MAVGKNKRLTKGGKKGAKKKVVDPFSKKDWYDVKAPAMFNIRNIGKTLVTRTQGTKIASDGLKGRVFEVS
LADLQNDEVAFRKFKLITEDVQGKNCLTNFHGMDLTRDKMCSMVKKWQTMIEAHVDVKTTDGYLLRLFCV
GFTKKRNNQIRKTSYAQHQQVRQIRKKMMEIMTREVQTNDLKEVVNKLIPDSIGKDIEKACQSIYPLHDV
FVRKVKMLKKPKSELGKLMELHGEGGSSGKAAGDETGAKVERADGYEPPVQESV

>sp|E7EP94|Modified_Residue:506
MAKAAAIGIDLGTTYSCVGVFQHGKVQISSMVLTKMKEIAEAYLGYPVTNAVITVPAYFNDSQRQATKDA
GVIAGLNVLRIINEPTAAAIAYGLDRTGKGERNVLIFDLGGGTFDVSILTIDDGIFEVKATAGDTHLGGE
DFDNRLVNHFVEEFKRKHKKDISQNKRAVRRLRTACERAKRTLSSSTQASLEIDSLFEGIDFYTSITRAR
FEELCSDLFRSTLEPVEKALRDAKLDKAQIHDLVLVGGSTRIPKVQKLLQDFFNGRDLNKSINPDEAVAY
GAAVQAAILMGDKSENVQDLLLLDVAPLSLGLETAGGVMTALIKRNSTIPTKQTQIFTTYSDNQPGVLIQ
VYEGERAMTKDNNLLGRFELSGIPPAPRGVPQIEVTFDIDANGILNVTATDKSTGKANKITITNDKGRLS
KEEIERMVQEAEKYKAEDEVQRERVSAKNALESYAFNMKSAVEDEGLKGKISEADKKKVLDKCQEVISWL
DANTLAEKDEFEHKRKELEQVCNPIISGLYQGAGGPGPGGFGAQGPKGGSGSGPTIEEVD

>sp|E9PZS8|Modified_Residue:56
MYYKFSSFTQKLAGAWASEAYTPQGLKPVSTEAPPIIFATPTKLTSSVTAYDYSGKNKVPELQKFFQPPF
LKENSLLQFGPLANNGTFVQKADGFHLKRGLPDQMLYRTTMALTLGGTIYCLIALYMASQPRNK

>sp|E9Q070|Modified_Residue:297
MPREDRATWKSNYFLKIIQLLDDYPKCFIVGADNVGSKQMQQIRMSLRGKAVVLMGKNTMMRKAIRGHLE
NNPALEKLLPHIRGNVGFVFTKEDLTEIRDMLLANKVPAAARAGAIAPCEVTVPAQNTGLGPEKTSFFQA
LGITTKISSGTIEILSDVQLIKTGDKVRASEATLLNMLNISPFSFGLIIQQVFDNGSIYNPEVLDITEQA
LHSRFLEGVRNVASVCLQIGYPTVASVPHSIINGYKRVLALSVETEYTFPLAEKVKAFLADPSAFAAAAP
AAAATTAAPAAAAAPAKAEAKEESEESDEDMGFGLFD

>sp|E9Q1V0|Modified_Residue:115
MGGKVPPATHKAKSEENTKEEKRDKTTEENIKTEELSSEESDLEIDNEGVIEPDTDAPQEMGDENAEITE
EMMDEANEKKGAAIEALNDGELQKAIDLFTDAIKLNPRLAILYAKRASVFVKLQKPNAA

>sp|E9Q3T0|Modified_Residue:98
MASVSELACIYSALILHGDEVTVTEDKINALIKAAGVSVEPFWPGLFAKALANVNIGSLICNVGAGGPAP
AAGAAPAGGAALSTAAAPAEEKKVEAKKEESEESEDDMGFGLFD

>sp|E9QMI7|Modified_Residue:675
MRSSASRLSSFSSRDSLWNRMPDQISVSEFIAETTEDYNSPTTSSFTTRLHNCRNTVTLLEEALDQDRTA
LQKVKKSVKAIYNSGQDHVQNEENYAQVLDKFGSNFLSRDNPDLGTAFVKFSTLTKELSTLLKNLLQGLS
HNVIFTLDSLLKGDLKGVKGDLKKPFDKAWKDYETKFTKIEKEKREHAKQHGMIRTEITGAEIAEEMEKE
RRLFQLQMCEYLIKVNEIKTKKGVDLLQNLIKYYHAQCNFFQDGLKTADKLKQYIEKLAADLYNIKQTQD
EEKKQLTALRDLIKSSLQLDPKEVGGLYVASRANSVIRDSQSRQGGYSMHQLQGNKEYGSEKKGFLLKKS
DGIRKVWQRRKCAVKNGILTISHATSNRQPAKLNLLTCQVKPNAEDKKSFDLISHNRTYHFQAEDEQDYI
AWISVLTNSKEEALTMAFRGEQSTGENSLEDLTKAIIEDVQRLPGNDICCDCGSSEPTWLSTNLGILTCI
ECSGIHREMGVHISRIQSLELDKLGTSELLLAKNVGNNSFNDIMEANLPSPSPKPTPSSDMTVRKEYITA
KYVDHRFSRKTCASSSAKLNELLEAIKSRDLLALIQVYAEGVELMEPLLEPGQELGETALHLAVRTADQT
SLHLVDFLVQNCGNLDKQTSVGNTVLHYCSMYGKPECLKLLLRSKPTVDIVNQNGETALDIAKRLKATQC
EDLLSQAKSGKFNPHVHVEYEWNLRQDEMDESDDDLDDKPSPIKKERSPRPQSFCHSSSISPQDKLALPG
FSTPRDKQRLSYGAFTNQIFASTSTDLPTSPTSEAPPLPPRNAGKGNDVGPLSSSKTANKFEGLSQQAST
SSAKTALGPRVLPKLPQKVALRKTETSHHLSLDRTNIPPETFQKSSQLTELPQKPPLGELPPKPVELAPK
PQVGELPPKPGELPPKPQLGDLPPKPQLSDLPPKPQMKDLPPKPQLGDLLAKSQAGDVSAKVQPPSEVTQ
RSHTGDLSPNVQSRDAIQKQASEDSNDLTPTLPETPVPLPRKINTGKNKVRRVKTIYDCQADNDDELTFI
EGEVIIVTGEEDQEWWIGHIEGQPERKGVFPVSFVHILSD

>sp|E9QNN1|Modified_Residue:1027
MGDIKNFLYAWCGKRKMTPAYEIRAVGNKNRQKFMCEVRVEGFNYAGMGNSTNKKDAQSNAARDFVNYLV
RINEVKSEEVPAVGIVPPPPILSDTSDSTASAAEGLPAPMGGPLPPHLALKAEAENNSGVESSGYGSPGP
TWDRGANLKDYYSRKEEQEVQATLESEEVDLNAGLHGNWTLENAKARLNQYFQKEKIQGEYKYTQVGPDH
NRSFIAEMTIYIKQLGRRIFAREHGSNKKLAAQSCALSLVRQLYHLGVIEAYSGLTKKKEGERVEPYKVF
LSPDLELQLQNVVQELDLEIVPPPVDPSMPVILNIGKLAHFEPSQRQNAVGVVPWSPPQSNWNPWTSSNI

DEGPLAYASTEQISMDLKNELTYQMEQDHNLQSVLQERELLPVKKFEAEILEAISSNSVVIIRGATGCGK
TTQVPQYILDDFIQNDRAAECNIVVTQPRRISAVAVAERVAYERGEEPGKSCGYSVRFESILPRPHASIM
FCTVGVLLRKLEAGIRGISHVIVDEIHERDINTDFLLVVLRDVVLAYPEVRIVLMSATIDTTMFCEYFFN
CPIIEVYGRTFPVQEYFLEDCIQMTQFIPPPKDKKKKDKEDDGGEDDDANCNLICGDEYGPETKLSMSQL
NEKETPFELIEALLKYIETLNVPGAVLVFLPGWNLIYTMQKHLENNSHFGSHRYQILPLHSQIPREEQRK
VFDPVPDGVTKVILSTNIAETSITINDVVYVIDSCKQKVKLFTAHNNMTNYATVWASKTNLEQRKGRAGR
VRPGFCFHLCSRARFDRLETHMTPEMFRTPLHEIALSIKLLRLGGIGQFLAKAIEPPPLDAVIEAEHTLR
ELDALDANDELTPLGRILAKLPIEPRFGKMMIMGCIFYVGDAVCTISAATCFPEPFISEGKRLGYIHRNF
AGNRFSDHVALLSVFQAWDDARMSGEEAEIRFCEQKRLNMATLRMTWEAKVQLKEILINSGFPEDCLLTQ
VFTNTGPDNNLDVVISLLAFGVYPNVCYHKEKRKILTTEGRNALIHKSSVNCPFSSQDMKYPSPFFVFGE
KIRTRAISAKGMTLVTPLQLLLFASKKVQSDGQIVFIDDWIRLQISHEAAACITALRAAMEALVVEVSKQ
PNIISQLDPVNEHMLNTIRQISRPSAAGINLMIGSVRYGDGPRPPKMARYDNGSGYRRGYGGGGYGGGGY
GGGYGSGGFGGGFGSGGGFGGGFNSGGGGFGSGGGGFGSGGGGFGGGGGGFSGGGGGGFGGGRGGGGGGF
GGSGGFGSGGGGYGVGGGGYGGGGGGGYGGGSGGYGGGGGGYGGGEGYSISPNSYRGNYGGGGGGYRGGS
QGGYRNNFGGDYRGSSGDYRGSGGGYRGSGGFQRRGYGGGYFGQGRGGGGGGGY

>sp|F6UB20|Modified_Residue:63
SSSTALARLGLPGQPRSTWLGVAALGLAAVALGTVAWRRTRPRRRRQLQQVGTVSKVWIYPIKSCKGVSV
CETECTDMGLRCGKVRDRFWMVVKEDGHMVTARQEPRLVLVSITLENNYLTLEAPGMEQIVLPIKLPSSN
KIHNCRLFGLDIKGRDCGDEVAQWFTNYLKTQAYRLVQFDTSMKGRTTKKLYPSESYLQNYEVAYPDCSP
VHLISEASLVDLNTRLKKKVKMEYFRPNIVVSGCEAFEEVRSDYSGPRHRHHRQEGATGDPEELSPV

>sp|F6YLP3|Modified_Residue:48
IKLAGVHSVASCFVVPMATGMSLTLCFLTLRHKRPKAKYIIWPRIDQKSCFKSMVTAGFEPVVIENVLEG
DELRTDLKAVEAKIQELGPEHILCLHSTTACFAPRVPDRGLGLVELMLSFRAWTKILWFQ

>sp|F8WIT2|Modified_Residue:442,445,446
MAKIAQGAMYRGSVHDFPEFDANQDAEALYTAMKGFGSDKESILELITSRSNKQRQEICQNYKSLYGKDL
IEDLKYELTGKFERLIVNLMRPLAYCDAKEIKDAISGVGTDEKCLIEILASRTNEQMHQLVAAYKDAYER
DLESDIIGDTSGHFQKMLVVLLQGTRENDDVVSEDLVQQDVQDLYEAGELKWGTDEAQFIYILGNRSKQH
LRLVFDEYLKTTGKPIEASIRGELSGDFEKLMLAVVKCIRSTPEYFAERLFKAMKGLGTRDNTLIRIMVS
RSELDMLDIREIFRTKYEKSLYSMIKNDTSGEYKKALLKLCGGDDDAAGQFFPEAAQVAYQMWELSAVSR
VELKGTVCAANDFNPDADAKALRKAMKGIGTDEATIIDIVTHRSNAQRQQIRQTFKSHFGRDLMADLKSE
ISGDLARLILGLMMPPAHYDAKQLKKAMEGAGTDEKTLIEILATRTNAEIRAINEAYKEDYHKSLEDALS
SDTSGHFRRILISLATGNREEGGENRDQAQEDAQEIADTPSGDKTSLETRFMTVLCTRSYPHLRRVFQEF
IKKTNYDIEHVIKKEMSGDVKDAFVAIVQSVKNKPLFFADKLYKSMKGAGTDEKTLTRVMVSRSEIDLLN
IRREFIEKYDKSLHQAIEGDTSGDFMKALLALCGGED

>sp|G3UYJ7|Modified_Residue:249
XLELYLDLMSQPCRAVYIFAKKNGIPFQLRTIELLKGQQYTDSFAQVNPLRKVPALKDGDFVLAESVAIL
LYLSRKYKAPDHWYPQDLQTRARVDEYLAWQHTALRSCCTRAMWQKMMFPVFLGQPVPPEMLASTLAELD
GCLQVLEDKFLRNQAFLTGSHISVADLVAITELMHRVSVTIRPGMTLLMNKSTEPCAHLLVSSIGVVGTA
EQNRTHSASFFKFLTEELSLDQDRIVIRFFPLEAWQIGKKGT

>sp|H3BTN5|Modified_Residue:367
MSKPHSEAGTAFIQTQQLHAAMADTFLEHMCRLDIDSPPITARNTGIICTIGPASRSVETLKEMIKSGMN
VARLNFSHGTHEYHAETIKNVRTATESFASDPILYRPVAVALDTKGPEIRTGLIKGSGTAEVELKKGATL
KITLDNAYMEKCDENILWLDYKNICKVVEVGSKIYVDDGLISLQVKQKGADFLVTEVENGGSLGSKKGVN
LPGAAVDLPAVSEKDIQDLKFGVEQDVDMVFASFIRKASDVHEVRKVLGEKGKNIKIISKIENHEGVRRF
DEILEASDGIMVARGDLGIEIPAEKVFLAQKMMIGRCNRAGKPVICATQMLESMIKKPRPTRAEGSDVAN
AVLDGADCIMLSGETAKGDYPLEAVRMQHLIAREAEAAMFHRKLFEELVRASSHSTDLMEAMAMGSVEAS
YKCLAAALIVLTESGRSAHQVARYRPRAPIIAVTRNPQTARQAHLYRGIFPVLCKDPVQEAWAED

>sp|I7HPV9|Modified_Residue:971

MLLGGRKTTDIPLEGYLLSPIQRICKYPLLLKELAKRTPGKHPDHTAVQSALQAMKTVCSNINETKRQME
KLEALEQLQSHIEGWEGSNLTDICTELLLQGNLLKISAGNIQERAFFLFDNLLVYCKRKSRVTGSKKSTK
RTKSINGSLYIFRGRINTEVMEVENVEDGTADYHSNGYTVTNGWKIHNTAKNKWFVCMAKTAEEKQKWLD
ALIREREQRESLKLGMERDAYVMIAEKGEKLYHMMMSKKVNLIKDRRRKLSTVPKCFLGNEFVAWLLEIG
EISKTEEGVNLGQALLENGIIHHVSDKHQFKNEQVMYRFRYDDGTYKARSELEDIMSKGVRLYCRLHSLY
APVIKDRDYHLKTYKSVVPGSKLVDWLLAQGDCQTREEAVALGVGLCNNGFMHHVLEKSEFKDESQYFRF
HADEEMEGTSSKNKQLRNDFKLVENILAKRLLIPPQEDDYGFDLEEKNKAVVVKSVQRGSLAEMAGLQAG
RKIYSINEDLVFLRPFSEVETILNQFFCSRRPLRLLVATKAKETIKVPDHPEALSFQIRGTAPPCVFAVG
RGSEAVAAGLCAGQCILKVNGTSVANDGALEVLEHFQAFRNHREEALGLYQWVYHSHEDAQLARASQGAP
DEDPQEDDQPDSALPLLSLGPQLSLHEDSAVVSLTLDNVHLEHGVVYEYMSTAGAKCHVLEKIVEPRGCF
RLAAKILEAFAVDDSIFVQNCGRLMAMSSAIVTMSHYEFHNICDTKLESIGQRIACYQEFAAQLKSRVSP
PFKQASLEPHPLCGLDFCPTNCHVNLMEVSYPKTTPSVGRSFSIRFGRKPSLIGLDPEQGLNPMAYTQHC
ITTMAAPSWKCSPAVDEDSQGQGLNDSSYGSASGAPSQQDRGLSFLLKQEDREIQDAYLQLFTKLDVALK
EMKQYVTQINRLLSTITEPTSAAPAPCDPSLVEETSSSPPVSEESEVDRTDHSGIKKVCFKVSEDEQEDS
GHDTMSYRDSYSECNSNRDSVLSYTSVRSNSSYLGSDEMGSGDELPCDMRIPSDKQDKLHGCLEHLFNQV
DSIHALLKGPVMSRAFEETRHFPMKHSWQEFKQKEECTVRGRNLIQISIQEDPWNLPSSIRTLVDNIQQY
VEDGKNQLLLALLKCTDTELQLRRDAVFCQALVAAVCTFSEQLLAALDYRYNNNGEYEESSRDASRKWLE
QVAATGVLLHWQSLLAPASVKEERTMLEDIWVTLSELDNVTFSFKQLDENSVANTNVFYHIEGSRQALKV
VFYLDGFHFSRLPSRLEGGASLRLHTVLFTKALESVEGPPPPGNQAAEELQQEINAQSLEKVQQYYRKLR
AFYLERSNLPTDAGATAVKIDQLIRPINALDELYRLMKTFVHPKAGAAGSLGAGLIPVSSELCYRLGACQ
ITMCGTGMQRSTLSVSLEQAAILARSHGLLPKCVMQATDIMRKQGPRVEILAKNLRIKDPMPQGAPRLYQ
LCQPPVDGDL

>sp|J3KMM5|Modified_Residue:205
MENAHTKTVEEVLGHFGVNESTGLSLEQVKKLKERWGSNELPAEEGKTLLELVIEQFEDLLVRILLLAAC
ISFVLAWFEEGEETITAFVEPFVILLILVANAIVGVWQERNAENAIEALKEYEPEMGKVYRQDRKSVQRI
KAKDIVPGDIVEIAVGDKVPADIRLTSIKSTTLRVDQSILTGESVSVIKHTDPVPDPRAVNQDKKNMLFS
GTNIAAGKAMGVVVATGVNTEIGKIRDEMVATEQERTPLQQKLDEFGEQLSKVISLICIAVWIINIGHFN
DPVHGGSWIRGAIYYFKIAVALAVAAIPEGLPAVITTCLALGTRRMAKKNAIVRSLPSVETLGCTSVICS
DKTGTLTTNQMSVCRMFILDKVEGDTCSLNEFSITGSTYAPIGEVQKDDKPVKCHQYDGLVELATICALC
NDSALDYNEAKGVYEKVGEATETALTCLVEKMNVFDTELKGLSKIERANACNSVIKQLMKKEFTLEFSRD
RKSMSVYCTPNKPSRTSMSKMFVKGAPEGVIDRCTHIRVGSTKVPMTPGVKQKIMSVIREWGSGSDTLRC
LALATHDNPLKREEMHLEDSANFIKYETNLTFVGCVGMLDPPRIEVASSVKLCRQAGIRVIMITGDNKGT
AVAICRRIGIFGQDEDVTSKAFTGREFDELSPSAQRDACLNARCFARVEPSHKSKIVEFLQSFDEITAMT
GDGVNDAPALKKSEIGIAMGSGTAVAKTASEMVLADDNFSTIVAAVEEGRAIYNNMKQFIRYLISSNVGE
VVCIFLTAALGFPEALIPVQLLWVNLVTDGLPATALGFNPPDLDIMNKPPRNPKEPLISGWLFFRYLAIG
CYVGAATVGAAAWWFIAADGGPRVSFYQLSHFLQCKEDNPDFDGVDCAIFESPYPMTMALSVLVTIEMCN
ALNSLSENQSLLRMPPWENIWLVGSICLSMSLHFLILYVEPLPLIFQITPLNLTQWLMVLKISLPVILMD
ETLKFVARNYLEQPDCLE

>sp|J3KRX8|Modified_Residue:91
MSGYSSDRDRGRDRGFGAPRFGGSRAGPLSGKKFGNPGEKLVKKKWNLDELPKFEKNFYQEHPDLARRTA
QEVETYRRSKEITVRGHNCPKPVLNFYEANFPANVMDVIARQNFTEPTAI

>sp|J3QMG3|Modified_Residue:15,20
MCNTPTYCDLGKAAKDVFNKGYGFGMVKIDLKTKSCSGVVEFSTSGHAYTDTGKASGNLETKYKVCNYGL
TFTQKWNTDNTLGTEISWENKLAEGLKLTLDTIFVPNTGKKSGKLKASYRRDCFSLGSNVDIDFSGPTIY
GWAVLAFEGWLAGYQMSFDTAKSKLSQNNFALGYKAADFQLHTHVNDGTEFGGSIYQKVNERIETSINLA
WTAGSNNTRFGIAAKYKLDCRTSLSAKVNNASLIGLGYTQTLRPGVKLTLSALIDGKNFNAGGHKVGLGF
ELEA

>sp|J3QNG0|Modified_Residue:340
MSSKGSVVLAYSGGLDTSCILVWLKEQGYDVIAYLANIGQKEDFEEARKKALKLGAKKVFIEDVSKEFVE
EFIWPAVQSSALYEDRYLLGTSLARPCIARRQVEIAQREGAKYVSHGATGKGNDQVRFELTCYSLAPQIK

VIAPWRMPEFYNRFKGRNDLMEYAKQHGIPIPVSPKSPWSMDENLMHISYEAGILENPKNQAPPGLYTKT
QDPAKAPNTPDVLEIEFKKGVPVKVTNIKDGTTRTTSLELFMYLNEVAGKHGVGRIDIVENRFIGMKSRG
IYETPAGTILYHAHLDIEAFTMDREVRKIKQGLGLKFAELVYTGFWHSPECEFVRHCIQKSQERVEGKVQ
VSVFKGQVYILGRESPLSLYNEELVSMNVQGDYEPIDATGFININSLRLKEYHRLQSKVTAK

>sp|O09172|Modified_Residue:263
MGTDSRAAGALLARASTLHLQTGNLLNWGRLRKKCPSTHSEELRDCIQKTLNEWSSQISPDLVREFPDVL
ECTMSHAVEKINPDEREEMKVSAKLFIVGSNSSSSTRSAVDMACSVLGVAQLDSVIMASPPIEDGVNLSL
EHLQPYWEELENLVQSKKIVAIGTSDLDKTQLEQLYQWAQVKPNSNQVNLASCCVMPPDLTAFAKQFDIQ
LLTHNDPKELLSEASFQEALQESIPDIEAQDWVPLWLLRYSVIVKSRGIIKSKGYILQAKRRGS

>sp|O70250|Modified_Residue:100
MTTHRLVMVRHGESLWNQENRFCGWFDAELSEKGAEEAKRGATAIKDAKIEFDICYTSVLKRAIRTLWTI
LDVTDQMWVPVVRTWRLNERHYGGLTGLNKAETAAKHGEEQVKIWRRSFDTPPPPMDEKHNYYTSISKDR
RYAGLKPEELPTCESLKDTIARALPFWNEEIAPKIKAGQRVLIAAHGNSLRGIVKHLEGMSDQAIMELNL
PTGIPIVYELDQNLKPTKPMRFLGDEETVRKAMEAVAAQGKAK

>sp|O88844|Modified_Residue:224
MSRKIQGGSVVEMQGDEMTRIIWELIKEKLILPYVELDLHSYDLGIENRDATNDQVTKDAAEAIKKYNVG
VKCATITPDEKRVEEFKLKQMWKSPNGTIRNILGGTVFREAIICKNIPRLVTGWVKPIIIGRHAYGDQYR
ATDFVVPGPGKVEITYTPKDGTQKVTYMVHDFEEGGGVAMGMYNQDKSIEDFAHSSFQMALSKGWPLYLS
TKNTILKKYDGRFKDIFQEIYDKKYKSQFEAQKICYEHRLIDDMVAQAMKSEGGFIWACKNYDGDVQSDS
VAQGYGSLGMMTSVLICPDGKTVEAEAAHGTVTRHYRMYQKGQETSTNPIASIFAWSRGLAHRAKLDNNT
ELSFFAKALEDVCIETIEAGFMTKDLAACIKGLPNVQRSDYLNTFEFMDKLGENLKAKLAQAKL

>sp|P01942|Modified_Residue:12,17,57
MVLSGEDKSNIKAAWGKIGGHGAEYGAEALERMFASFPTTKTYFPHFDVSHGSAQVKGHGKKVADALASA
AGHLDDLPGALSALSDLHAHKLRVDPVNFKLLSHCLLVTLASHHPADFTPAVHASLDKFLASVSTVLTSK
YR

>sp|P06733|Modified_Residue:335,343
MSILKIHAREIFDSRGNPTVEVDLFTSKGLFRAAVPSGASTGIYEALELRDNDKTRYMGKGVSKAVEHIN
KTIAPALVSKKLNVTEQEKIDKLMIEMDGTENKSKFGANAILGVSLAVCKAGAVEKGVPLYRHIADLAGN
SEVILPVPAFNVINGGSHAGNKLAMQEFMILPVGAANFREAMRIGAEVYHNLKNVIKEKYGKDATNVGDE
GGFAPNILENKEGLELLKTAIGKAGYTDKVVIGMDVAASEFFRSGKYDLDFKSPDDPSRYISPDQLADLY
KSFIKDYPVVSIEDPFDQDDWGAWQKFTASAGIQVVGDDLTVTNPKRIAKAVNEKSCNCLLLKVNQIGSV
TESLQACKLAQANGWGVMVSHRSGETEDTFIADLVVGLCTGQIKTGAPCRSERLAKYNQLLRIEEELGSK
AKFAGRNFRNPLAK

>sp|P07724|Modified_Residue:236
MKWVTFLLLLFVSGSAFSRGVFRREAHKSEIAHRYNDLGEQHFKGLVLIAFSQYLQKCSYDEHAKLVQEV
TDFAKTCVADESAANCDKSLHTLFGDKLCAIPNLRENYGELADCCTKQEPERNECFLQHKDDNPSLPPFE
RPEAEAMCTSFKENPTTFMGHYLHEVARRHPYFYAPELLYYAEQYNEILTQCCAEADKESCLTPKLDGVK
EKALVSSVRQRMKCSSMQKFGERAFKAWAVARLSQTFPNADFAEITKLATDLTKVNKECCHGDLLECADD
RAELAKYMCENQATISSKLQTCCDKPLLKKAHCLSEVEHDTMPADLPAIAADFVEDQEVCKNYAEAKDVF
LGTFLYEYSRRHPDYSVSLLLRLAKKYEATLEKCCAEANPPACYGTVLAEFQPLVEEPKNLVKTNCDLYE
KLGEYGFQNAILVRYTQKAPQVSTPTLVEAARNLGRVGTKCCTLPEDQRLPCVEDYLSAILNRVCLLHEK
TPVSEHVTKCCSGSLVERRPCFSALTVDETYVPKEFKAETFTFHSDICTLPEKEKQIKKQTALAELVKHK
PKATAEQLKTVMDDFAQFLDTCCKAADKDTCFSTEGPNLVTRCKDALA

>sp|P11214|Modified_Residue:167
MKRELLCVLLLCGLAFPLPDQGIHGRFRRGARSYRATCRDEPTQTTYQQHQSWLRPMLRSSRVEYCRCNS
GLVQCHSVPVRSCSEPRCFNGGTCQQALYFSDFVCQCPDGFVGKRCDIDTRATCFEEQGITYRGTWSTAE
SGAECINWNSSVLSLKPYNARRPNAIKLGLGNHNYCRNPDRDLKPWCYVFKAGKYTTEFCSTPACPKGKS

EDCYVGKGVTYRGTHSLTTSQASCLPWNSIVLMGKSYTAWRTNSQALGLGRHNYCRNPDGDARPWCHVMK
DRKLTWEYCDMSPCSTCGLRQYKRPQFRIKGGLYTDITSHPWQAAIFVKNKRSPGERFLCGGVLISSCWV
LSAAHCFLERFPPNHLKVVLGRTYRVVPGEEEQTFEIEKYIVHEEFDDDTYDNDIALLQLRSQSKQCAQE
SSSVGTACLPDPNLQLPDWTECELSGYGKHEASSPFFSDRLKEAHVRLYPSSRCTSQHLFNKTVTNNMLC
AGDTRSGGNQDLHDACQGDSGGPLVCMINKQMTLTGIISWGLGCGQKDVPGVYTKVTNYLDWIHDNMKQ

>sp|P11881|Modified_Residue:569
MSDKMSSFLHIGDICSLYAEGSTNGFISTLGLVDDRCVVQPEAGDLNNPPKKFRDCLFKLCPMNRYSAQK
QFWKAAKPGANSTTDAVLLNKLHHAADLEKKQNETENRKLLGTVIQYGNVIQLLHLKSNKYLTVNKRLPA
LLEKNAMRVTLDEAGNEGSWFYIQPFYKLRSIGDSVVIGDKVVLNPVNAGQPLHASSHQLVDNPGCNEVN
SVNCNTSWKIVLFMKWSDNKDDILKGGDVVRLFHAEQEKFLTCDEHRKKQHVFLRTTGRQSATSATSSKA
LWEVEVVQHDPCRGGAGYWNSLFRFKHLATGHYLAAEVDPDFEEECLEFQPSVDPDQDASRSRLRNAQEK
MVYSLVSVPEGNDISSIFELDPTTLRGGDSLVPRNSYVRLRHLCTNTWVHSTNIPIDKEEEKPVMLKIGT
SPLKEDKEAFAIVPVSPAEVRDLDFANDASKVLGSIAGKLEKGTITQNERRSVTKLLEDLVYFVTGGTNS
GQDVLEVVFSKPNRERQKLMREQNILKQIFKLLQAPFTDCGDGPMLRLEELGDQRHAPFRHICRLCYRVL
RHSQQDYRKNQEYIAKQFGFMQKQIGYDVLAEDTITALLHNNRKLLEKHITAAEIDTFVSLVRKNREPRF
LDYLSDLCVSMNKSIPVTQELICKAVLNPTNADILIETKLVLSRFEFEGVSTGENALEAGEDEEEVWLFW
RDSNKEIRSKSVRELAQDAKEGQKEDRDILSYYRYQLNLFARMCLDRQYLAINEISGQLDVDLILRCMSD
ENLPYDLRASFCRLMLHMHVDRDPQEQVTPVKYARLWSEIPSEIAIDDYDSSGTSKDEIKERFAQTMEFV
EEYLRDVVCQRFPFSDKEKNKLTFEVVNLARNLIYFGFYNFSDLLRLTKILLAILDCVHVTTIFPISKMT
KGEENKGSNVMRSIHGVGELMTQVVLRGGGFLPMTPMAAAPEGNVKQAEPEKEDIMVMDTKLKIIEILQF
ILNVRLDYRISCLLCIFKREFDESNSQSSETSSGNSSQEGPSNVPGALDFEHIEEQAEGIFGGSEENTPL
DLDDHGGRTFLRVLLHLTMHDYPPLVSGALQLLFRHFSQRQEVLQAFKQVQLLVTSQDVDNYKQIKQDLD
QLRSIVEKSELWVYKGQGPDEPMDGASGENEHKKTEEGTSKPLKHESTSSYNYRVVKEILIRLSKLCVQE
SASVRKSRKQQQRLLRNMGAHAVVLELLQIPYEKAEDTKMQEIMRLAHEFLQNFCAGNQQNQALLHKHIN
LFLNPGILEAVTMQHIFMNNFQLCSEINERVVQHFVHCIETHGRNVQYIKFLQTIVKAEGKFIKKCQDMV
MAELVNSGEDVLVFYNDRASFQTLIQMMRSERDRMDENSPLMYHIHLVELLAVCTEGKNVYTEIKCNSLL
PLDDIVRVVTHEDCIPEVKIAYINFLNHCYVDTEVEMKEIYTSNHMWKLFENFLVDICRACNNTSDRKHA
DSILEKYVTEIVMSIVTTFFSSPFSDQSTTLQTRQPVFVQLLQGVFRVYHCNWLMPSQKASVESCIRVLS
DVAKSRAIAIPVDLDSQVNNLFLKSHNIVQKTALNWRLSARNAARRDSVLAASRDYRNIIERLQDIVSAL
EDRLRPLVQAELSVLVDVLHRPELLFPENTDARRKCESGGFICKLIKHTKQLLEENEEKLCIKVLQTLRE
MMTKDRGYGEKQISIDESENAELPQAPEAENSTEQELEPSPPLRQLEDHKRGEALRQILVNRYYGNIRPS
GRRESLTSFGNGPLSPGGPSKPGGGGGGPGSSSTSRGEMSLAEVQCHLDKEGASNLVIDLIMNASSDRVF
HESILLAIALLEGGNTTIQHSFFCRLTEDKKSEKFFKVFYDRMKVAQQEIKATVTVNTSDLGNKKKDDEV
DRDAPSRKKAKEPTTQITEEVRDQLLEASAATRKAFTTFRREADPDDHYQSGEGTQATTDKAKDDLEMSA
VITIMQPILRFLQLLCENHNRDLQNFLRCQNNKTNYNLVCETLQFLDCICGSTTGGLGLLGLYINEKNVA
LINQTLESLTEYCQGPCHENQNCIATHESNGIDIITALILNDINPLGKKRMDLVLELKNNASKLLLAIME
SRHDSENAERILYNMRPKELVEVIKKAYMQGEVEFEDGENGEDGAASPRNVGHNIYILAHQLARHNKELQ
TMLKPGGQVDGDEALEFYAKHTAQIEIVRLDRTMEQIVFPVPSICEFLTKESKLRIYYTTERDEQGSKIN
DFFLRSEDLFNEMNWQKKLRAQPVLYWCARNMSFWSSISFNLAVLMNLLVAFFYPFKGVRGGTLEPHWSG
LLWTAMLISLAIVIALPKPHGIRALIASTILRLIFSVGLQPTLFLLGAFNVCNKIIFLMSFVGNCGTFTR
GYRAMVLDVEFLYHLLYLLICAMGLFVHEFFYSLLLFDLVYREETLLNVIKSVTRNGRSIILTAVLALIL
VYLFSIVGYLFFKDDFILEVDRLPNETAVPETGESLANDFLYSDVCRVETGENCTSPAPKEELLPAEETE
QDKEHTCETLLMCIVTVLSHGLRSGGGVGDVLRKPSKEEPLFAARVIYDLLFFFMVIIIVLNLIFGVIID
TFADLRSEKQKKEEILKTTCFICGLERDKFDNKTVTFEEHIKEEHNMWHYLCFIVLVKVKDSTEYTGPES
YVAEMIRERNLDWFPRMRAMSLVSSDSEGEQNELRNLQEKLESTMKLVTNLSGQLSELKDQMTEQRKQKQ
RIGLLGHPPHMNVNPQQPA

>sp|P11983|Modified_Residue:243
MEGPLSVFGDRSTGEAVRSQNVMAAASIANIVKSSFGPVGLDKMLVDDIGDVTITNDGATILKLLEVEHP
AAKVLCELADLQDKEVGDGTTSVVIIAAELLKNADELVKQKIHPTSVISGYRLACKEAVRYINENLIINT
DELGRDCLINAAKTSMSSKIIGINGDYFANMVVDAVLAVKYTDARGQPRYPVNSVNILKAHGRSQIESML
INGYALNCVVGSQGMPKRIVNAKIACLDFSLQKTKMKLGVQVVITDPEKLDQIRQRESDITKERIQKILA
TGANVILTTGGIDDMYLKYFVEAGAMAVRRVLKRDLKHVAKASGASILSTLANLEGEETFEVTMLGQAEE

VVQERICDDELILIKNTKARTSASIILRGANDFMCDEMERSLHDALCVVKRVLELKSVVPGGGAVEAALS
IYLENYATSMGSREQLAIAEFARSLLVIPNTLAVNAAQDSTDLVAKLRAFHNEAQVNPERKNLKWIGLDL
VHGKPRDNKQAGVFEPTIVKVKSLKFATEAAITILRIDDLIKLHPESKDDKHGSYENAVHSGALDD

>sp|P12710|Modified_Residue:96
MNFSGKYQLQSQENFEPFMKAIGLPEDLIQKGKDIKGVSEIVHEGKKIKLTITYGPKVVRNEFTLGEECE
LETMTGEKVKAVVKLEGDNKMVTTFKGIKSVTELNGDTITNTMTLGDIVYKRVSKRI

>sp|P12790|Modified_Residue:237
MDPSVLLLLAVLLSLFLLLVRGHAKIHGHLPPGPHPLPLLGNLLQMDRGGLLKCFIQLQEKHGDVFTVHL
GPRPVVVLCGTQTIREALVDHAEAFSGRGTIAAAQLVMQDYGIFFASGQRWKTLRRFSLATMKEFGMGKR
SVEERIKEEAQCLVEELKKYQGVPLDPTFLFQCITANIICSIVFGERFDYTDDQFLHLLNLMYKIFSLLS
SFSGQMFELFSGFLKYFPGVHRQIVKKQQELLDYIAHSVEKHKATLDPSAPRDYIDTYLLRMEKEKSNHN
TEFHHQNLMMSVLSLFFVGTETTSATLHYGVLLMLKYPHVTEKVQKEIDQVIGSHRLPTLDDRTKMPYTD
AVIHEIQRFSDLVPIGLPHKVIKDTLFRGYLLPKNTEVYPVLSSALHDPQYFEQPDKFNPEQFLDANGAL
KKCEAFLPFSTGKRICLGESIARNELFIFFTTILQNFSVASPVAPKDIDLTPKESGIGKIPPAHQIYFLA
R

>sp|P14174|Modified_Residue:78
MPMFIVNTNVPRASVPDGFLSELTQQLAQATGKPPQYIAVHVVPDQLMAFGGSSEPCALCSLHSIGKIGG
AQNRSYSKLLCGLLAERLRISPDRVYINYYDMNAANVGWNNSTFA

>sp|P16015|Modified_Residue:64
MAKEWGYASHNGPDHWHELYPIAKGDNQSPIELHTKDIKHDPSLQPWSASYDPGSAKTILNNGKTCRVVF
DDTYDRSMLRGGPLSGPYRLRQFHLHWGSSDDHGSEHTVDGVKYAAELHLVHWNPKYNTFGEALKQPDGI
AVVGIFLKIGREKGEFQILLDALDKIKTKGKEAPFTHFDPSCLFPACRDYWTYHGSFTTPPCEECIVWLL
LKEPMTVSSDQMAKLRSLFSSAENEPPVPLVGNWRPPQPVKGRVVRASFK

>sp|P16879|Modified_Residue:161
MGFSSELCSPQGHGAVQQMQEAELRLLEGMRKWMAQRVKSDREYAGLLHHMSLQDSGGQSWSSGPDSPVS
QSWAEITSQTENLSRVLRQHAEDLNSGPLSKLSVLIRERQHLRKTYNEQWQQLQQELTKTHSQDIEKLKT
QYRTLVRDSTQARRKYQEASKDKDRDKAKDKYVRSLWKLFAHHNRYVLGVRAAQLHHHHHHRFMLPGLLQ
SLQDLHEEMAGILKDILQEYLEISSLVQDDVASIHRELAAAAARIQPEFEYLGFLRQYGSTPDVPPCVTF
DESLLEDGEQLEPGELQLNELTLESVQHTLTSVTDELAVATKEVLSRQEMVSQLQRELQSEEQNTHPRER
VQLLSKRQMLQEAIQGLQIALCSQDKLQAQQELLQSKMEQLGTGEPPAVPLLQDDRHSTSSTEQEREGGR
TPTLEILKSHFSGIFRPKFSIPPPLQLVPEVQKPLYEQLWYHGAIPRAEVAELLTHSGDFLVRESQGKQE
YVLSVMWDGQPRHFIIQSSDNLYRLEGDGFPSIPLLITHLLSSQQPLTKKSGVVLFRAVPKDKWVLKHED
LVLGEQIGRGNFGEVFSGRLRADNTPVAVKSCRETLPPDLKAKFLQEARILKQYNHPNIVRLIGVCTQKQ
PIYIVMELVQGGDFLTFLRTEGARLRVKTLLQMVGDAAAGMEYLESKCCIHRDLAARNCLVTEKNVLKIS
DFGMSREEADGIYAASAGLRQVPVKWTAPEALNYGRYSSESDVWSFGILLWETFSLGASPYPNLTNQQTR
EFVEKGHRLPCPELCPDAVFRLMEQCWAYEPGQRPSFSIICQELHSIRKRHR

>sp|P18894|Modified_Residue:259,263
MRVAVIGAGVIGLSTALCIHERYHPTQPLHMKIYADRFTPFTTSDVAAGLWQPYLSDPSNPQEAEWSQQT
FDYLLSCLHSPNAEKMGLALISGYNLFRDEVPDPFWKNAVLGFRKLTPSEMDLFPDYGYGWFNTSLLLEG
KSYLPWLTERLTERGVKLIHRKVESLEEVARGVDVIINCTGVWAGALQADASLQPGRGQIIQVEAPWIKH
FILTHDPSLGIYNSPYIIPGSKTVTLGGIFQLGNWSGLNSVRDHNTIWKSCCKLEPTLKNARIVGELTGF
RPVRPQVRLEREWLRHGSSSAEVIHNYGHGGYGLTIHWGCAMEAANLFGKILEEKKLSRLPPSHL

>sp|P22752|Modified_Residue:96
MSGRGKQGGKARAKAKTRSSRAGLQFPVGRVHRLLRKGNYSERVGAGAPVYLAAVLEYLTAEILELAGNA
ARDNKKTRIIPRHLQLAIRNDEELNKLLGRVTIAQGGVLPNIQAVLLPKKTESHHKAKGK

>sp|P23881|Modified_Residue:199

MGLEEELLRIAKKLEKMVSRKKTEGALDLLKKLNSCQMSIQLLQTTRIGVAVNGVRKHCSDKEVVSLAKV
LIKNWKRLLDSPRTTKGEREEREKAKKEKGLGCSDWKPEAGLSPPRKKGGGEPKTRRDSVDSRSSTTSSP
KRPSLERSNSSKSKVETPTTPSSPSTPTFAPAVCLLAPCYLTGDSVRDKCVEMLSAALKAEDNFKDYGVN
CDKLASEIEDHIYQELKSTDMKYRNRVRSRISNLKDPRNPGLRRNVLSGAISPELIAKMTAEEMASDELR
ELRNAMTQEAIREHQMAKTGGTTTDLLRCSKCKKKNCTYNQVQTRSADEPMTTFVLCNECGNRWKFC

>sp|P26040|Modified_Residue:60
MPKPINVRVTTMDAELEFAIQPNTTGKQLFDQVVKTIGLREVWYFGLQYVDNKGFPTWLKLDKKVSAQEV
RKENPVQFKFRAKFYPEDVAEELIQDITQKLFFLQVKDGILSDEIYCPPETAVLLGSYAVQAKFGDYNKE
MHKSGYLSSERLIPQRVMDQHKLSRDQWEDRIQVWHAEHRGMLKDSAMLEYLKIAQDLEMYGINYFEIKN
KKGTDLWLGVDALGLNIYEKDDKLTPKIGFPWSEIRNISFNDKKFVIKPIDKKAPDFVFYAPRLRINKRI
LQLCMGNHELYMRRRKPDTIEVQQMKAQAREEKHQKQLERQQLETEKKRRETVEREKEQMLREKEELMLR
LQDYEQKTKRAEKELSEQIEKALQLEEERRRAQEEAERLEADRMAALRAKEELERQAQDQIKSQEQLAAE
LAEYTAKIALLEEARRRKEDEVEEWQHRAKEAQDDLVKTKEELHLVMTAPPPPPPPVYEPVNYHVQEGLQ
DEGAEPMGYSAELSSEGILDDRNEEKRITEAEKNERVQRQLLTLSNELSQARDENKRTHNDIIHNENMRQ
GRDKYKTLRQIRQGNTKQRIDEFEAM

>sp|P26443|Modified_Residue:503
MYRRLGEALLLSRAGPAALGSAAADSAALLGWARGQPSAAPQPGLTPVARRHYSEAAADREDDPNFFKMV
EGFFDRGASIVEDKLVEDLKTRESEEQKRNRVRGILRIIKPCNHVLSLSFPIRRDDGSWEVIEGYRAQHS
QHRTPCKGGIRYSTDVSVDEVKALASLMTYKCAVVDVPFGGAKAGVKINPKNYTDNELEKITRRFTMELA
KKGFIGPGIDVPAPDMSTGEREMSWIADTYASTIGHYDINAHACVTGKPISQGGIHGRISATGRGVFHGI
ENFINEASYMSILGMTPGFGDKTFVVQGFGNVGLHSMRYLHRFGAKCVGVGESDGSIWNPDGIDPKELED
FKLQHGSILGFPKAKVYEGSILEADCDILIPAASEKQLTKSNAPRVKAKIIAEGANGPTTPEADKIFLER
NIMVIPDLYLNAGGVTVSYFEWLKNLNHVSYGRLTFKYERDSNYHLLMSVQESLERKFGKHGGTIPVVPT
AEFQDRISGASEKDIVHSGLAYTMERSARQIMRTAMKYNLGLDLRTAAYVNAIEKVFKVYNEAGVTFT

>sp|P35585|Modified_Residue:296
MSASAVYVLDLKGKVLICRNYRGDVDMSEVEHFMPILMEKEEEGMLSPILAHGGVRFMWIKHNNLYLVAT
SKKNACVSLVFSFLYKVVQVFSEYFKELEEESIRDNFVIIYELLDELMDFGYPQTTDSKILQEYITQEGH
KLETGAPRPPATVTNAVSWRSEGIKYRKNEVFLDVIEAVNLLVSANGNVLRSEIVGSIKMRVFLSGMPEL
RLGLNDKVLFDNTGRGKSKSVELEDVKFHQCVRLSRFENDRTISFIPPDGEFELMSYRLNTHVKPLIWIE
SVIEKHSHSRIEYMVKAKSQFKRRSTANNVEIHIPVPNDADSPKFKTTVGSVKWVPENSEIVWSVKSFPG
GKEYLMRAHFGLPSVEAEDKEGKPPISVKFEIPYFTTSGIQVRYLKIIEKSGYQALPWVRYITQNGDYQL
RTQ

>sp|P47806|Modified_Residue:79
MFNPMTPPQVNSYSEPCCLRPLHSQGVPSMGTEGLSGLPFCHQANFMSGSQGYGAARETSSCTEGSLFPP
PPPPRSSVKLTKKRALSISPLSDASLDLQTVIRTSPSSLVAFINSRCTSPGGSYGHLSIGTMSPSLGFPP
QMSHQKGTSPPYGVQPCVPHDSTRGSMMLHPQSRGPRATCQLKSELDMMVGKCPEDPLEGDMSSPNSTGT
QDHLLGMLDGREDLEREEKPEPESVYETDCRWDGCSQEFDSQEQLVHHINSEHIHGERKEFVCHWGGCSR
ELRPFKAQYMLVVHMRRHTGEKPHKCTFEGCRKSYSRLENLKTHLRSHTGEKPYMCEQEGCSKAFSNASD
RAKHQNRTHSNEKPYVCKLPGCTKRYTDPSSLRKHVKTVHGPDAHVTKRHRGDGPLPRAQPLSTVEPKRE
REGGSGREESRLTVPESAMPQQSPGAQSSCSSDHSPAGSAANTDSGVEMAGNAGGSTEDLSSLDEGPCVS
ATGLSTLRRLENLRLDQLHQLRPIGSRGLKLPSLTHAGAPVSRRLGPPVSLDRRSSSSSSMSSAYTVSRR
SSLASPFPPGTPPENGASSLPGLTPAQHYMLRARYASARGSGTPPTAAHSLDRMGGLSVPPWRSRTEYPG
YNPNAGVTRRASDPARAADHPAPARVQRFKSLGCVHTPPSVATGRNFDPHHPTSVYSPQPPSITENVAMD
TRGLQEEPEVGTSVMGNGLNPYMDFSSTDTLGYGGPEGTAAEPYEARGPGSLPLGPGPPTNYGPGHCAQQ
VSYPDPTPENWGEFPSHAGVYPSNKAPGAAYSQCPRLEHYGQVQVKPEQGCPVGSDSTGLAPCLNAHPSE
GSPGPQPLFSHHPQLPQPQYPQSGPYPQPPHGYLSTEPRLGLNFNPSSSHSTGQLKAQLVCNYVQSQQEL
LWEGRNRGGLPNQELPYQSPKFLGGSQVSQSPAKTPAAAAAAYGSGFAPASANHKSGSYPAPSPCHETFT
VGVNRPSHRPAAPPRLLPPLSPCYGPLKVGDTNPSCGHPEVGRLGAGPALYPPPEGQVCNALDSLDLDNT
QLDFVAILDEAQGLSPPLSHEQGDSSKNTPSPSGPPNMAVGNMSVLLGSLPGETQFLNSSA

>sp|P47857|Modified_Residue:678
MTHEEHHAAKTLGIGKAIAVLTSGGDAQGMNAAVRAVVRVGIFTGARVFFVHEGYQGLVDGGEHIREATW
ESVSMMLQLGGTVIGSARCKDFREREGRLRAAHNLVKRGITNLCVIGGDGSLTGADTFRSEWSDLLNDLQ
KDGKITAEEATKSSYLNIVGLVGSIDNDFCGTDMTIGTDSALHRIVEIVDAITTTAQSHQRTFVLEVMGR
HCGYLALVTSLSCGADWVFIPECPPDDDWEEHLCRRLSETRTRGSRLNIIIVAEGAIDKNGKPITSEDIK
NLVVKRLGYDTRVTVLGHVQRGGTPSAFDRILGSRMGVEAVMALLEGTPDTPACVVSLSGNQAVRLPLME
CVQVTKDVTKAMDEKRFDEAIKLRGRSFMNNWEVYKLLAHVRPPVSKGGLHTVAVMNVGAPAAGMNAAVR
STVRIGLIQGNRVLVVHDGFEGLAKGQIEEAGWSYVGGWTGQGGSKLGTKRTLPKKNLEQISANITKFNI
QGLVIIGGFEAYTGGLELMEGRKQFDELCIPFVVIPATVSNNVPGSDFSIGADTALNTICTTCDRIKQSA
AGTKRRVFIIETMGGYCGYLATMAGLAAGADAAYIFEEPFTIRDLQVNVEHLVQKMKTTVKRGLVLRNEK
CNENYTTDFIFNLYSEEGKGIFDSRKNVLGHMQQGGSPTPFDRNFATKMGAKAMNWMSGKIKESYRNGRI
FANTPDSGCVLGMRKRALVFQPVTELKDQTDFEHRIPKEQWWLKLRPILKILAKYEIDLDTSDHAHLEHI
SRKRSGEAAV

>sp|P48962|Modified_Residue:92,96
MGDQALSFLKDFLAGGIAAAVSKTAVAPIERVKLLLQVQHASKQISAEKQYKGIIDCVVRIPKEQGFLSF
WRGNLANVIRYFPTQALNFAFKDKYKQIFLGGVDRHKQFWRYFAGNLASGGAAGATSLCFVYPLDFARTR
LAADVGKGSSQREFNGLGDCLTKIFKSDGLKGLYQGFSVSVQGIIIYRAAYFGVYDTAKGMLPDPKNVHI
IVSWMIAQSVTAVAGLVSYPFDTVRRRMMMQSGRKGADIMYTGTLDCWRKIAKDEGANAFFKGAWSNVLR
GMGGAFVLVLYDEIKKYV

>sp|P49006|Modified_Residue:144
MGSQSSKAPRGDVTAEEAAGASPAKANGQENGHVKSNGDLSPKGEGESPPVNGTDEAAGATGDAIEPAPP
SQGAEAKGEVPPKETPKKKKKFSFKKPFKLSGLSFKRNRKEGGGDSSASSPTEEEQEQGEIGACSDEGTA
QEGKAAATPESQEPQAKGAEASAASEEEAGPQATEPSTPSGPESGPTPASAEQNE

>sp|P49722|Modified_Residue:92
MAERGYSFSLTTFSPSGKLVQIEYALAAVAGGAPSVGIKAANGVVLATEKKQKSILYDERSVHKVEPITK
HIGLVYSGMGPDYRVLVHRARKLAQQYYLVYQEPIPTAQLVQRVASVMQEYTQSGGVRPFGVSLLICGWN
EGRPYLFQSDPSGAYFAWKATAMGKNYVNGKTFLEKRYNEDLELEDAIHTAILTLKESFEGQMTEDNIEV
GICNEAGFRRLTPTEVRDYLAAIA

>sp|P51814|Modified_Residue:297
MAANGDSPPWSPALAAEGRGSSCEVRRERTPEARIHSVKRYPDLSPGPKGRSSADHAALNSIVSLQASVS
FEDVTVDFSKEEWQHLDPAQRRLYWDVTLENYSHLLSVGYQIPKSEAAFKLEQGEGPWMLEGEAPHQSCS
GEAIGKMQQQGIPGGIFFHCERFDQPIGEDSLCSILEELWQDNDQLEQRQENQNNLLSHVKVLIKERGYE
HKNIEKIIHVTTKLVPSIKRLHNCDTILKHTLNSHNHNRNSATKNLGKIFGNGNNFPHSPSSTKNENAKT
GANSCEHDHYEKHLSHKQAPTHHQKIHPEEKLYVCTECVMGFTQKSHLFEHQRIHAGEKSRECDKSNKVF
PQKPQVDVHPSVYTGEKPYLCTQCGKVFTLKSNLITHQKIHTGQKPYKCSECGKAFFQRSDLFRHLRIHT
GEKPYECSECGKGFSQNSDLSIHQKTHTGEKHYECNECGKAFTRKSALRMHQRIHTGEKPYVCADCGKAF
IQKSHFNTHQRIHTGEKPYECSDCGKSFTKKSQLHVHQRIHTGEKPYICTECGKVFTHRTNLTTHQKTHT
GEKPYMCAECGKAFTDQSNLIKHQKTHTGEKPYKCNGCGKAFIWKSRLKIHQKSHIGERHYECKDCGKAF
IQKSTLSVHQRIHTGEKPYVCPECGKAFIQKSHFIAHHRIHTGEKPYECSDCGKCFTKKSQLRVHQKIHT
GEKPNICAECGKAFTDRSNLITHQKIHTREKPYECGDCGKTFTWKSRLNIHQKSHTGERHYECSKCGKAF
IQKATLSMHQIIHTGKKPYACTECQKAFTDRSNLIKHQKMHSGEKRYKASD

>sp|P60174|Modified_Residue:256
MAEDGEEAEFHFAALYISGQWPRLRADTDLQRLGSSAMAPSRKFFVGGNWKMNGRKQSLGELIGTLNAAK
VPADTEVVCAPPTAYIDFARQKLDPKIAVAAQNCYKVTNGAFTGEISPGMIKDCGATWVVLGHSERRHVF
GESDELIGQKVAHALAEGLGVIACIGEKLDEREAGITEKVVFEQTKVIADNVKDWSKVVLAYEPVWAIGT
GKTATPQQAQEVHEKLRGWLKSNVSDAVAQSTRIIYGGSVTGATCKELASQPDVDGFLVGGASLKPEFVD
IINAKQ

>sp|P62814|Modified_Residue:48

MALRAMRGIVNGAAPELPVPTGGPMAGAREQALAVSRNYLSQPRLTYKTVSGVNGPLVILDHVKFPRYAE
IVHLTLPDGTKRSGQVLEVSGSKAVVQVFEGTSGIDAKKTSCEFTGDILRTPVSEDMLGRVFNGSGKPID
RGPVVLAEDFLDIMGQPINPQCRIYPEEMIQTGISAIDGMNSIARGQKIPIFSAAGLPHNEIAAQICRQA
GLVKKSKDVVDYSEENFAIVFAAMGVNMETARFFKSDFEENGSMDNVCLFLNLANDPTIERIITPRLALT
TAEFLAYQCEKHVLVILTDMSSYAEALREVSAAREEVPGRRGFPGYMYTDLATIYERAGRVEGRNGSITQ
IPILTMPNDDITHPIPDLTGYITEGQIYVDRQLHNRQIYPPINVLPSLSRLMKSAIGEGMTRKDHADVSN
QLYACYAIGKDVQAMKAVVGEEALTSDDLLYLEFLQKFEKNFITQGPYENRTVYETLDIGWQLLRIFPKE
MLKRIPQSTLSEFYPRDSAKH

>sp|P62900|Modified_Residue:70
MAPAKKGGEKKKGRSAINEVVTREYTINIHKRIHGVGFKKRAPRALKEIRKFAMKEMGTPDVRIDTRLNK
AVWAKGIRNVPYRIRVRLSRKRNEDEDSPNKLYTLVTYVPVTTFKNLQTVNVDEN

>sp|P62908|Modified_Residue:201
MAVQISKKRKFVADGIFKAELNEFLTRELAEDGYSGVEVRVTPTRTEIIILATRTQNVLGEKGRRIRELT
AVVQKRFGFPEGSVELYAEKVATRGLCAIAQAESLRYKLLGGLAVRRACYGVLRFIMESGAKGCEVVVSG
KLRGQRAKSMKFVDGLMIHSGDPVNYYVDTAVRHVLLRQGVLGIKVKIMLPWDPSGKIGPKKPLPDHVSI
VEPKDEILPTTPISEQKGGKPEPPAMPQPVPTA

>sp|P68104|Modified_Residue:330
MGKEKTHINIVVIGHVDSGKSTTTGHLIYKCGGIDKRTIEKFEKEAAEMGKGSFKYAWVLDKLKAERERG
ITIDISLWKFETSKYYVTIIDAPGHRDFIKNMITGTSQADCAVLIVAAGVGEFEAGISKNGQTREHALLA
YTLGVKQLIVGVNKMDSTEPPYSQKRYEEIVKEVSTYIKKIGYNPDTVAFVPISGWNGDNMLEPSANMPW
FKGWKVTRKDGNASGTTLLEALDCILPPTRPTDKPLRLPLQDVYKIGGIGTVPVGRVETGVLKPGMVVTF
APVNVTTEVKSVEMHHEALSEALPGDNVGFNVKNVSVKDVRRGNVAGDSKNDPPMEAAGFTAQVIILNHP
GQISAGYAPVLDCHTAHIACKFAELKEKIDRRSGKKLEDGPKFLKSGDAAIVDMVPGKPMCVESFSDYPP
LGRFAVRDMRQTVAVGVIKAVDKKAAGAGKVTKSAQKAQKAK

>sp|P99028|Modified_Residue:83
MGLEDERKMLTGSGDPKEEEEEEELVDPLTTVREHCEQLEKCVKARERLELCDNRVSSRSQTEEDCTEELF
DFLHARDHCVAHKLFKNLK

>sp|Q13541|Modified_Residue:69
MSGGSSCSQTPSRAIPATRRVVLGDGVQLPPGDYSTTPGGTLFSTTPGGTRIIYDRKFLMECRNSPVTKT
PPRDLPTIPGVTSPSSDEPPMEASQSHLRNSPEDKRAGGEESQFEMDI

>sp|Q3UJB0|Modified_Residue:853
MAAEHPEPPKGELQLPPPPPPGHYGAWAAQELQARLAEIGAPIQGSREELVERLQTYTRQTGIVLNRPVL
RGEDGDKAAPPPMSAQLSGIPMPPPPMGLPPLQPPPPPPPPPPGLGLGFPMAHPPNLGPPPPLRVGEPVA
LSEEERLKLAQQQAALLMQQEERAKQAAVLMEQERQQEIAKMGTAVPRPPQDMGQLGVRTPLGPRVAAPV
GPVVPTPTVLPMGAPVPRPRGPPPPPGDENREMDDPSVGPKIPQALEKILQLKESRQEEMNSQQEEEEME
TDTRSSLGQSASETEEDTVSISKKEKNRKRRNRKKKKKPQRVRAASSESSGDREKDSGRSRGSDPPAADV
EIEYVTEEPEIYEPNFIFFKRIFEAFKLTDDVKKEKEKEPEKLDKMESSAVPKKKGFEEEHKDSDDDSSD
DEQEKKPEAPKLSKKKLRRMNRFTVAELKQLVARPDVVEMHDVTAQDPKLLVHLKATRNSVPVPRHWCFK
RKYLQGKRGIEKPPFELPDFIKRTGIQEMREALQEKEEQKTMKSKMREKVRPKMGKIDIDYQKLHDAFFK
WQTKPKLTIHGDLYYEGKEFETRLKEKKPGDLSDELRISLGMPVGPNAHKVPPPWLIAMQRYGPPPSYPN
LKIPGLNSPIPESCSFGYHAGGWGKPPVDETGKPLYGDVFGTNAAEFQTKTEEEEIDRTPWGELEPSDEE
SSEEEEEEESDEDKPDETGFITPADSGLITPGGFSSVPAGMETPELIELRKKKIEEAMDGSETPQLFTVL
PEKRTATVGGAMMGSTHIYDMSTVMSRKGPAPELQGVEVALAPEELELDPMAMTQKYEEHVREQQAQVEK
EDFSDMVAEHAAKQKQKKRKAQPQDSRGGSKKYKEFKF

>sp|Q3UL97|Modified_Residue:53
MESPDRKRQKVLKAKKTMPTSYQKQLEILNKSTNVEAPKTTVGTNIPNGHNQKMFSKNKENVKVMKVSEQ
INENACGALERHTALLEQVKHWIRQEICMINCNLFDKKLNELNERIGKTQCKSRHEAIAGELFVKIRRLQ

KRIKTVLSSQRNCLEPNTLPSNTVCKVTDSEAMNLNVTQKSVKSRSKRISSVNHTPLNSSEKAGRKTNLP
STCVEFASESNTDDVMLISVKNSNLTTSITSEQTEIRKNTSRNLSNSPNSMIKVGPVEKKFDFVIDLTRE
GPSNYSIESPSFTLKSTSKAVLRSKEIIPVAENGNEGFGSFEHLPPLPEPPAPLPEMADKIKDTLPPQKP
ELKVKWVLRPTSIALTWNIPKVNPNCAPVESYHLFLYYENSDHLTWKKIAEIKALPLPMACTLSQNLAST
KYYFAVQSKDIFGRYGPFCNIKSIPRFSENLT

>sp|Q3UNI1|Modified_Residue:697
MADEVALALQAAGSPSAAAAMEAASQPADEPLRKRPRRDGPGLGRSPGEPSAAVAPAAAGCEAASAAAPA
ALWREAAGAAASAEREAPATAVAGDGDNGSGLRREPRAADDFDDDEGEEEDEAAAAAAAAAIGYRGPYTF
VQQHLMIGTDPRTILKDLLPETIPPPELDDMTLWQIVINILSEPPKRKKRKDINTIEDAVKLLQECKKII
VLTGAGVSVSCGIPDFRSRDGIYARLAVDFPDLPDPQAMFDIEYFRKDPRPFFKFAKEIYPGQFQPSLCH
KFIALSDKEGKLLRNYTQNIDTLEQVAGIQRILQCHGSFATASCLICKYKVDCEAVRGDIFNQVVPRCPR
CPADEPLAIMKPEIVFFGENLPEQFHRAMKYDKDEVDLLIVIGSSLKVRPVALIPSSIPHEVPQILINRE
PLPHLHFDVELLGDCDVIINELCHRLGGEYAKLCCNPVKLSEITEKPPRPQKELVHLSELPPTPLHISED
SSSPERTVPQDSSVIATLVDQATNNNVNDLEVSESSCVEEKPQEVQTSRNVENINVENPDFKAVGSSTAD
KNERTSVAETVRKCWPNRLAKEQISKRLEGNQYLFVPPNRYIFHGAEVYSDSEDDVLSSSSCGSNSDSGT
CQSPSLEEPLEDESEIEEFYNGLEDDTERPECAGGSGFGADGGDQEVVNEAIATRQELTDVNYPSDKS

>sp|Q5JSZ5|Modified_Residue:386,398
MSDRLGQITKGKDGKSKYSTLSLFDKYKGKSVDAIRSSVIPRHGLQSLGKVAAARRMPPPANLPSLKSEN
KGNDPNIVIVPKDGTGWANKQDQQDPKSSSATASQPPESLPQPGLQKSVSNLQKPTQSISQENTNSVPGG
PKSWAQLNGKPVGHEGGLRGSSRLLSFSPEEFPTLKAAGGQDKAGKEKGVLDLSYGPGPSLRPQNVTSWR
EGGGRHIISATSLSTSPTELGSRNSSTGDGAPSSACTSDSKDPSLRPAQPVRKGASQFMGNVYHPPTYHD
MLPAFMCSPKSSENQGTVERGSFPLPQLRLEPRVPFRQFQMNDQDGKENRLGLSRPLRPLRQLVERAPRP
TIINAENLKGLDDLDADADDGWAGLHEEVDYSEKLKFSDDEEEEEVVKDGRPKWNSWDPRRQRQLSMSSA
DSADAKRTREEGKDWAEAVGASRVVRKAPDPQPPPRKLHGWAPGPDYQKSSMGSMFRQQSIEDKEDKPPP
RQKFIQSEMSEAVERARKRREEEERRAREERLAACAAKLKQLDQKCKQARKAGEARKQAEKEVPWSPSAE
KASPQENGPAVHKGSPEFPAQETPTTFPEEAPTVSPAVAQSNSSEEEAREAGSPAQEFKYQKSLPPRFQR
QQQQQQQEQLYKMQHWQPVYPPPSHPQRTFYPHHPQMLGFDPRWMMMPSYMDPRITPTRTPVDFYPSALH
PSGLMKPMMPQESLNGTGCRSEDQNCVPPLQERKVTPIDSPPVWSPEGYMALQSKGYPLPHPKSSDTLAM
DMRVRNESSFSASLGRAGGVSAQRDLFEERGEEYLSAFDKKAQADFDSCISSQRIGQELLFPPQENVQDA
GAPGGHTQNLRCSPLEPDFVPDEKKPECGSWDVSHQPETADTAHGVERETPREGTAFNISSWDKNGSPNK
QPSSEPEWTPEPRSSSSQHPEQTGRTRRSGPIKKPVLKALKVEDKEKELEKIKQELGEESTRLAKEKEQS
PTAEKDEDEENDASLANSSTTTLEDKGPGHATFGREATKFEEEEKPDKAWEARPPRESSDVPPMKRNNWI
FIDEEQAFGVRGQARGRGRGFREFTFRGRPAGGNGSGLCGGGVLGARSIYCSSQRSGRGRGLREFARPED
CPRAKPRRRVASETHSEGSEYEELPKRRRQRGSENGNEGSLLEREESTLKKGDCRDSWRSNKGCSEDHSG
LDAKSRGPRAFGRALPPRLSNCGYGRRTFVSKESPHWQSKSPGSSWQEYGPSDTCGSRRPTDRDYVPDSY
RHPDAFGGRGFEDSRAEDKRSFFQDEHVADSENAENRPFRRRRPPRQDKPPRFRRLRQERESLGLWGPEE
EPHLLAGQWPGRPKLCSGDKSGTVGRRSPELSYQNSSDHANEEWETASESSDFSERRERREGPGSEPDSQ
VDGGLSGASLGEKKELAKRSFSSQRPVVDRQSRKLEPGGFGEKPVRPGGGDTSPRYESQQNGTPLKVKRS
PDEALPGGLSGCSSGSGHSPYALERAAHASADLPEASSKKAEKEAKLAAPRAGEQGEAMKQFDLNYGSAI
IENCGSSPGEESEVGSMVGEGFIEVLTKKQRRLLEEERRKKEQAVQVPVKGRGLSSRIPPRFAKKQNNLC
LEQGDVTVPGSSLGTEIWESSSQALPVQAPANDSWRKAVTAFSSTETGSAEQGFKSSQGDSGVDLSAESR
ESSATSSQRSSPYGTLKPEEMSGPGLAEPKADSHKEQAPKPSEQKDSEQGSGQSKEHRPGPIGNERSLKN
RKGSEGAERLQGAVVPPVNGVEIHVDSVLPVPPIEFGVSPKDSDFSLPPGSASGPTGSPVVKLQDALASN
AGLTQSIPILRRDHHIQRAIGLSPMSFPTADLTLKMESARKAWENSPSLPEQSSPGGAGSGIQPPSSVGA
SSGVNYSSFGGVSMPPMPVASVAPSASMPGSHLPPLYLDGHVFASQPRLVPQTIPQQQSYQQAAAAQQIP
ISLHTSLQAQAQLGLRGGLPVSQSQEIFSSLQPFRSQVYMHPSLSPPSTMILSGGTALKPPYSAFPGMQP
LEMVKPQSGSPYQPMSGNQALVYEGQLSQAAGLGASQMLDSQLPQLTMPLPRYGSGQQPLILPQSIQLPP
GQSLSVGAPRRIPPPGSQPPVLNTSREPSQMEMKGFHFADSKQNVPSGGPVPSPQTYRPSSASPSGKPSG
SAVNMGSVQGHYVQQAKQRVDEKPSLGAVKLQEAPSAASQMKRTGAIKPRAVKVEESKA

>sp|Q5RL73|Modified_Residue:351
MASSGGELGSLFDHHVQRAVCDTRAKYREGRRPRAVKVYTINLESQYLLIQGVPAVGVMKELVERFALYG

AIEQYNALDEYPAEDFTEVYLIKFMNLQSARTAKRKMDEQSFFGGLLHVCYAPEFETVEETRKKLQMRKA
YVVKTTENKDHYVTKKKLVTEHKDTEDFRQDFHSEMSGFCKAALNTSAGNSNPYLPYSCELPLCYFSSKC
MCSSGGPVDRAPDSSKDGRNHHKTMGHYNHNDSLRKTQINSLKNSVACPGAQKAITSSEAVDRFMPRTTQ
LQERKRRREDDRKLGTFLQTNPTGNEIMIGPLLPDISKVDMHDDSLNTTANLIRHKLKEVISSVPKPPED
KPEDVHTSHPLKQRRRI

>sp|Q64FW2|Modified_Residue:55
MWITALLLAVLLLVILHRVYVGLYAASSPNPFAEDVKRPPEPLVTDKEARKKVLKQAFSVSRVPEKLDAV
VIGSGIGGLASAAVLAKAGKRVLVLEQHTKAGGCCHTFGENGLEFDTGIHYIGRMREGNIGRFILDQITE
GQLDWAPMASPFDLMILEGPNGRKEFPMYSGRKEYIQGLKKKFPKEEAVIDKYMELVKVVARGVSHAVLL
KFLPLPLTQLLSKFGLLTRFSPFCRASTQSLAEVLQQLGASRELQAVLSYIFPTYGVTPSHTAFSLHALL
VDHYIQGAYYPRGGSSEIAFHTIPLIQRAGGAVLTRATVQSVLLDSAGRACGVSVKKGQELVNIYCPVVI
SNAGMFNTYQHLLPETVRHLPDVKKQLAMVRPGLSMLSIFICLKGTKEDLKLQSTNYYVYFDTDMDKAME
RYVSMPKEKAPEHIPLLFIAFPSSKDPTWEERFPDRSTMTALVPMAFEWFEEWQEEPKGKRGVDYETLKN
AFVEASMSVIMKLFPQLEGKVESVTGGSPLTNQYYLAAPRGATYGADHDLARLHPHAMASIRAQTPIPNL
YLTGQDIFTCGLMGALQGALLCSSAILKRNLYSDLQALGSKVKAQKKKM

>sp|Q6PKG0|Modified_Residue:1083
MATQVEPLLPGGATLLQAEEHGGLVRKKPPPAPEGKGEPGPNDVRGGEPDGSARRPRPPCAKPHKEGTGQ
QERESPRPLQLPGAEGPAISDGEEGGGEPGAGGGAAGAAGAGRRDFVEAPPPKVNPWTKNALPPVLTTVN
GQSPPEHSAPAKVVRAAVPKQRKGSKVGDFGDAINWPTPGEIAHKSVQPQSHKPQPTRKLPPKKDMKEQE
KGEGSDSKESPKTKSDESGEEKNGDEDCQRGGQKKKGNKHKWVPLQIDMKPEVPREKLASRPTRPPEPRH
IPANRGEIKGSESATYVPVAPPTPAWQPEIKPEPAWHDQDETSSVKSDGAGGARASFRGRGRGRGRGRGR
GRGGTRTHFDYQFGYRKFDGVEGPRTPKYMNNITYYFDNVSSTELYSVDQELLKDYIKRQIEYYFSVDNL
ERDFFLRRKMDADGFLPITLIASFHRVQALTTDISLIFAALKDSKVVEIVDEKVRRREEPEKWPLPPIVD
YSQTDFSQLLNCPEFVPRQHYQKETESAPGSPRAVTPVPTKTEEVSNLKTLPKGLSASLPDLDSENWIEV
KKRPRPSPARPKKSEESRFSHLTSLPQQLPSQQLMSKDQDEQEELDFLFDEEMEQMDGRKNTFTAWSDEE
SDYEIDDRDVNKILIVTQTPHYMRRHPGGDRTGNHTSRAKMSAELAKVINDGLFYYEQDLWAEKFEPEYS
QIKQEVENFKKVNMISREQFDTLTPEPPVDPNQEVPPGPPRFQQVPTDALANKLFGAPEPSTIARSLPTT
VPESPNYRNTRTPRTPRTPQLKDSSQTSRFYPVVKEGRTLDAKMPRKRKTRHSSNPPLESHVGWVMDSRE
HRPRTASISSSPSEGTPTVGSYGCTPQSLPKFQHPSHELLKENGFTQHVYHKYRRRCLNERKRLGIGQSQ
EMNTLFRFWSFFLRDHFNKKMYEEFKQLALEDAKEGYRYGLECLFRYYSYGLEKKFRLDIFKDFQEETVK
DYEAGQLYGLEKFWAFLKYSKAKNLDIDPKLQEYLGKFRRLEDFRVDPPMGEEGNHKRHSVVAGGGGGEG
RKRCPSQSSSRPAAMISQPPTPPTGQPVREDAKWTSQHSNTQTLGK

>sp|Q80ZV3|Modified_Residue:47
MLRGGSMTAELGVGFALRAVNERVQQSVARRPRDLPAIQPRLVAVSKTKPADMVIEAYGHGQRTFGENYI
LSSCPEIKWHFIGHLQKQNVNKLMAVPNLSMLETVDSVKLADKVNSSWQKKGPTEPLKVMVQINTSGEDS
K

>sp|Q86UP2|Modified_Residue:292
MEFYESAYFIVLIPSIVITVIFLFFWLFMKETLYDEVLAKQKREQKLIPTKTDKKKAEKKKNKKKEIQNG
NLHESDSESVPRDFKLSDALAVEDDQVAPVPLNVVETSSSVRERKKKEKKQKPVLEEQVIKESDASKIPG
KKVEPVPVTKQPTPPSEAAASKKKPGQKKSKNGSDDQDKKVETLMVPSKRQEALPLHQETKQESGSGKKK
ASSKKQKTENVFVDEPLIHATTYIPLMDNADSSPVVDKREVIDLLKPDQVEGIQKSGTKKLKTETDKENA
EVKFKDFLLSLKTMMFSEDEALCVVDLLKEKSGVIQDALKKSSKGELTTLIHQLQEKDKLLAAVKEDAAA
TKDRCKQLTQEMMTEKERSNVVITRMKDRIGTLEKEHNVFQNKIHVSYQETQQMQMKFQQVREQMEAEIA
HLKQENGILRDAVSNTTNQLESKQSAELNKLRQDYARLVNELTEKTGKLQQEEVQKKNAEQAATQLKVQL
QEAERRWEEVQSYIRKRTAEHEAAQQDLQSKFVAKENEVQSLHSKLTDTLVSKQQLEQRLMQLMESEQKR
VNKEESLQMQVQDILEQNEALKAQIQQFHSQIAAQTSASVLAEELHKVIAEKDKQIKQTEDSLASERDRL
TSKEEELKDIQNMNFLLKAEVQKLQALANEQAAAAHELEKMQQSVYVKDDKIRLLEEQLQHEISNKMEEF
KILNDQNKALKSEVQKLQTLVSEQPNKDVVEQMEKCIQEKDEKLKTVEELLETGLIQVATKEEELNAIRT
ENSSLTKEVQDLKAKQNDQVSFASLVEELKKVIHEKDGKIKSVEELLEAELLKVANKEKTVQDLKQEIKA
LKEEIGNVQLEKAQQLSITSKVQELQNLLKGKEEQMNTMKAVLEEKEKDLANTGKWLQDLQEENESLKAH

VQEVAQHNLKEASSASQFEELEIVLKEKENELKRLEAMLKERESDLSSKTQLLQDVQDENKLFKSQIEQL
KQQNYQQASSFPPHEELLKVISEREKEISGLWNELDSLKDAVEHQRKKNNDLREKNWEAMEALASTEKML
QDKVNKTSKERQQQVEAVELEAKEVLKKLFPKVSVPSNLSYGEWLHGFEKKAKECMAGTSGSEEVKVLEH
KLKEADEMHTLLQLECEKYKSVLAETEGILQKLQRSVEQEENKWKVKVDESHKTIKQMQSSFTSSEQELE
RLRSENKDIENLRREREHLEMELEKAEMERSTYVTEVRELKDLLTELQKKLDDSYSEAVRQNEELNLLKA
QLNETLTKLRTEQNERQKVAGDLHKAQQSLELIQSKIVKAAGDTTVIENSDVSPETESSEKETMSVSLNQ
TVTQLQQLLQAVNQQLTKEKEHYQVLE

>sp|Q8BH04|Modified_Residue:262
MAAMYLPGLRLSRHGLRPWCWSPCRSIQTLHVLSGDMSQLPAGVRDFVARSAHLCQPEGIHICDGTEAEN
TAILALLEEQGLIRKLPKYKNCWLARTDPKDVARVESKTVIVTPSQRDTVPLLAGGARGQLGNWMSPDEF
QRAVDERFPGCMQGRIMYVLPFSMGPVGSPLSRIGVQLTDSAYVVASMRIMTRLGTPVLQALGDGDFIKC
LHSVGQPLTGHGDPVGQWPCNPEKTLIGHVPDQREIVSFGSGYGGNSLLGKKCFALRIASRLARDEGWLA
EHMLILGITNPAGKKRYVAAAFPSACGKTNLAMMRPALPGWKVECVGDDIAWMRFDSEGQLRAINPENGF
FGVAPGTSAATNPNAMATIQSNTLFTNVAETSDGGVYWEGIDQPLPPGVTITSWLGKPWKPGDKEPCAHP
NSRFCVPARQCPIMDPAWEAPEGVPIDAIIFGGRRPKGVPLVYEAFNWRHGVFVGSAMRSESTAAAEHKG
KTIMHDPFAMRPFFGYNFGRYLEHWLSMEGQKGARLPRIFHVNWFRRDEAGRFLWPGFGENARVLDWICR
RLEGEDSAQETPIGLVPKEGALDLSGLSAVDTSQLFSIPKDFWEQEVRDIRGYLTEQVNQDLPKEVLAEL
EALEGRVQKM

>sp|Q8BMS1|Modified_Residue:214
MVASRAIGSLSRFSAFRILRSRGCICRSFTTSSALLTRTHINYGVKGDVAVIRINSPNSKVNTLNKEVQS
EFIEVMNEIWANDQIRSAVLISSKPGCFVAGADINMLSSCTTPQEATRISQEGQRMFEKLEKSPKPVVAA
ISGSCLGGGLELAIACQYRIATKDRKTVLGVPEVLLGILPGAGGTQRLPKMVGVPAAFDMMLTGRNIRAD
RAKKMGLVDQLVEPLGPGIKSPEERTIEYLEEVAVNFAKGLADRKVSAKQSKGLVEKLTTYAMTVPFVRQ
QVYKTVEEKVKKQTKGLYPAPLKIIDAVKAGLEQGSDAGYLAESQKFGELALTKESKALMGLYNGQVLCK
KNKFGAPQKNVQQLAILGAGLMGAGIAQVSVDKGLKTLLKDTTVTGLGRGQQQVFKGLNDKVKKKALTSF
ERDSIFSNLIGQLDYKGFEKADMVIEAVFEDLGVKHKVLKEVESVTPEHCIFASNTSALPINQIAAVSKR
PEKVIGMHYFSPVDKMQLLEIITTDKTSKDTTASAVAVGLRQGKVIIVVKDGPGFYTTRCLAPMMSEVMR
ILQEGVDPKKLDALTTGFGFPVGAATLADEVGVDVAQHVAEDLGKAFGERFGGGSVELLKQMVSKGFLGR
KSGKGFYIYQEGSKNKSLNSEMDNILANLRLPAKPEVSSDEDVQYRVITRFVNEAVLCLQEGILATPAEG
DIGAVFGLGFPPCLGGPFRFVDLYGAQKVVDRLRKYESAYGTQFTPCQLLLDHANNSSKKFYQ

>sp|Q8C196|Modified_Residue:214,228,811,1479
MTRILTACKVVKTLKSGFGFANVTTKRQWDFSRPGIRLLSVKAKTAHIVLEDGTKMKGYSFGHPSSVAGE
VVFNTGLGGYPEALTDPAYKGQILTMANPIIGNGGAPDTTARDELGLNKYMESDGIKVAGLLVLNYSNDY
NHWLATKSLGQWLQEEKVPAIYGVDTRMLTKIIRDKGTMLGKIEFEGQSVDFVDPNKQNLIAEVSTKDVK
VFGKGNPTKVVAVDCGIKNNVIRLLVKRGAEVHLVPWNHDFTQMEYDGLLIAGGPGNPALAQPLIQNVKK
ILESDRKEPLFGISTGNIITGLAAGAKSYKMSMANRGQNQPVLNITNRQAFITAQNHGYALDNTLPAGWK
PLFVNVNDQTNEGIMHESKPFFAVQFHPEVSPGPTDTEYLFDSFFSLIKKGKGTTITSVLPKPALVASRV
EVSKVLILGSGGLSIGQAGEFDYSGSQAVKAMKEENVKTVLMNPNIASVQTNEVGLKQADAVYFLPITPQ
FVTEVIKAERPDGLILGMGGQTALNCGVELFKRGVLKEYGVKVLGTSVESIMATEDRQLFSDKLNEINEK
IAPSFAVESMEDALKAADTIGYPVMIRSAYALGGLGSGICPNKETLIDLGTKAFAMTNQILVERSVTGWK
EIEYEVVRDADDNCVTVCNMENVDAMGVHTGDSVVVAPAQTLSNAEFQMLRRTSVNVVRHLGIVGECNIQ
FALHPTSMEYCIIEVNARLSRSSALASKATGYPLAFIAAKIALGIPLPEIKNVVSGKTSACFEPSLDYMV
TKIPRWDLDRFHGTSSRIGSSMKSVGEVMAIGRTFEESFQKALRMCHPSVDGFTPRLPMNKEWPANLDLK
KELSEPSSTRIYAIAKALENNMSLDEIVRLTSIDKWFLYKMRDILNMDKTLKGLNSDSVTEETLRKAKEI
GFSDKQISKCLGLTEAQTRELRLKKNIHPWVKQIDTLAAEYPSVTNYLYVTYNGQEHDIKFDEHGIMVLG
CGPYHIGSSVEFDWCAVSSIRTLRQLGKKTVVVNCNPETVSTDFDECDKLYFEELSLERILDIYHQEACN
GCIISVGGQIPNNLAVPLYKNGVKIMGTSPLQIDRAEDRSIFSAVLDELKVAQAPWKAVNTLNEALEFAN
SVGYPCLLRPSYVLSGSAMNVVFSEDEMKRFLEEATRVSQEHPVVLTKFVEGAREVEMDAVGKEGRVISH
AISEHVEDAGVHSGDATLMLPTQTISQGAIEKVKDATRKIAKAFAISGPFNVQFLVKGNDVLVIECNLRA
SRSFPFVSKTLGVDFIDVATKVMIGESIDEKRLPTLEQPIIPSDYVAIKAPMFSWPRLRDADPILRCEMA
STGEVACFGEGIHTAFLKAMLSTGFKIPQKGILIGIQQSFRPRFLGVAEQLHNEGFKLFATEATSDWLNA

NNVPATPVAWPSQEGQNPSLSSIRKLIRDGSIDLVINLPNNNTKFVHDNYVIRRTAVDSGIALLTNFQVT
KLFAEAVQKSRTVDSKSLFHYRQYSAGKAA

>sp|Q8VD72-2|Modified_Residue:33
MGSEMEPLLRAWSYFRRRKFQLCADLCTQMLEKSPYDQAAWILKARALTEMVYIDEIDVDQEGIAEMILD
ENAIAQVPRPGTSLKLPGTNQTGGPTQAVRPITQAGRPITGFLRPSTQSGRPGTMEQAIRTPRTAYTARP
ITSSSGRFVRLGTASMLTSPDGPFINLSRLNLTKYSQKPKLAKALFEYILHHENDVKMALDLASLSTEYS
QYKDWWWKVQIGKCYYRLGMYREAEKQFKSALKQQEMVDTFLYLAKVYIILDQPVTALNLFKQGLDKFPG
EVTLLCGIARIYEEMNNSSSAAEYYKEVLKQDNTHVEAIACIGSNHFYSDQPEVALRFYRRLLQMGVYNC
QLFNNLGLCCFYAQQYDMTLTSFERALSLAENEEEAADVWYNLGHIAVGIGDTNLAHQCFRLALVHNNHH
AEAYNNLAVLEMRKGHVEQARALLQTASSLAPHMYEPHFNFATVSDKIGDLQRSYVAAQKSEVAFPEHVD
TQHLIKQLKQHFAML

>sp|Q91V76|Modified_Residue:75
MACSEFSFHMPSLEELAEVLQKGLTDNFADVQVSVVDCPDLTKEPFTFPVRGICGQTRIAEVGGVPYLLP
LVNKKKVYDLNEIAKVIKLPGAFILGAGAGPFQTLGFNSEFMPIVQTASEHNQPVNGSYFAHKNPADGAC
LLEKYSQKYHDFGCALLANLFASEGQPGKVIEVQAKRRTGELNFVSCMRQTLEEHYGDKPVGMGGTFIVQ
KGKVKAHIMPAEFSSCPLNSDEAVNKWLHFYEMKAPLVCLPVFVSKDPGLDLRLEHTHFFSHHGEGGHYH
YDTTPDTVEYLGYFSPAQFLYRIDQPKETHAFGRD

>sp|Q920E5|Modified_Residue:57
MNGNQKLDAYNQEKQNFIQHFSQIVKVLTEKELGHPEIGDAIARLKEVLEYNALGGKYNRGLTVVQAFQE
LVEPKKQDAESLQRALTVGWCVELLQAFFLVSDDIMDSSLTRRGQICWYQKPGIGLDAINDALLLEASIY
RLLKFYCREQPYYLNLLELFLQSSYQTEIGQTLDLMTAPQGHVDLGRYTEKRYKSIVKYKTAFYSFYLPI
AAAMYMAGIDGEKEHANALKILMEMGEFFQVQDDYLDLFGDPSVTGKVGTDIQDNKCSWLVVQCLLRASP
QQRQILEENYGQKDPEKVARVKALYEALDLQSAFFKYEEDSYNRLKSLIEQCSAPLPPSIFMELANKIYK
RRK

>sp|Q93092|Modified_Residue:314
MSGSPVKRQRMESALDQLKQFTTVVADTGDFNAIDEYKPQDATTNPSLILAAAQMPAYQELVEEAIAYGK
KLGGPQEEQIKNAIDKLFVLFGAEILKKIPGRVSTEVDARLSFDKDAMVARARRLIELYKEAGVGKDRIL
IKLSSTWEGIQAGKELEEQHGIHCNMTLLFSFAQAVACAEAGVTLISPFVGRILDWHVANTDKKSYEPQE
DPGVKSVTKIYNYYKKFGYKTIVMGASFRNTGEIKALAGCDFLTISPKLLGELLKDNSKLAPALSVKAAQ
TSDSEKIHLDEKAFRWLHNEDQMAVEKLSDGIRKFAADAIKLERMLTERMFSAENGK

>sp|Q9BYV9|Modified_Residue:177
MSVDEKPDSPMYVYESTVHCTNILLGLNDQRKKDILCDVTLIVERKEFRAHRAVLAACSEYFWQALVGQT
KNDLVVSLPEEVTARGFGPLLQFAYTAKLLLSRENIREVIRCAEFLRMHNLEDSCFSFLQTQLLNSEDGL
FVCRKDAACQRPHEDCENSAGEEEDEEEETMDSETAKMACPRDQMLPEPISFEAAAIPVAEKEEALLPEP
DVPTDTKESSEKDALTQYPRYKKYQLACTKNVYNASSHSTSGFASTFREDNSSNSLKPGLARGQIKSEPP
SEENEEESITLCLSGDEPDAKDRAGDVEMDRKQPSPAPTPTAPAGAACLERSRSVASPSCLRSLFSITKS
VELSGLPSTSQQHFARSPACPFDKGITQGDLKTDYTPFTGNYGQPHVGQKEVSNFTMGSPLRGPGLEALC
KQEGELDRRSVIFSSSACDQVSTSVHSYSGVSSLDKDLSEPVPKGLWVGAGQSLPSSQAYSHGGLMADHL
PGRMRPNTSCPVPIKVCPRSPPLETRTRTSSSCSSYSYAEDGSGGSPCSLPLCEFSSSPCSQGARFLATE
HQEPGLMGDGMYNQVRPQIKCEQSYGTNSSDESGSFSEADSESCPVQDRGQEVKLPFPVDQITDLPRNDF
QMMIKMHKLTSEQLEFIHDVRRRSKNRIAAQRCRKRKLDCIQNLECEIRKLVCEKEKLLSERNQLKACMG
ELLDNFSCLSQEVCRDIQSPEQIQALHRYCPVLRPMDLPTASSINPAPLGAEQNIAASQCAVGENVPCCL
EPGAAPPGPPWAPSNTSENCTSGRRLEGTDPGTFSERGPPLEPRSQTVTVDFCQEMTDKCTTDEQPRKDY
T

>sp|Q9C026|Modified_Residue:347
MEEMEEELKCPVCGSFYREPIILPCSHNLCQACARNILVQTPESESPQSHRAAGSGVSDYDYLDLDKMSL
YSEADSGYGSYGGFASAPTTPCQKSPNGVRVFPPAMPPPATHLSPALAPVPRNSCITCPQCHRSLILDDR
GLRGFPKNRVLEGVIDRYQQSKAAALKCQLCEKAPKEATVMCEQCDVFYCDPCRLRCHPPRGPLAKHRLV

PPAQGRVSRRLSPRKVSTCTDHELENHSMYCVQCKMPVCYQCLEEGKHSSHEVKALGAMWKLHKSQLSQA
LNGLSDRAKEAKEFLVQLRNMVQQIQENSVEFEACLVAQCDALIDALNRRKAQLLARVNKEHEHKLKVVR
DQISHCTVKLRQTTGLMEYCLEVIKENDPSGFLQISDALIRRVHLTEDQWGKGTLTPRMTTDFDLSLDNS
PLLQSIHQLDFVQVKASSPVPATPILQLEECCTHNNSATLSWKQPPLSTVPADGYILELDDGNGGQFREV
YVGKETMCTVDGLHFNSTYNARVKAFNKTGVSPYSKTLVLQTSEVAWFAFDPGSAHSDIILSNDNLTVTC
SSYDDRVVLGKTGFSKGIHYWELTVDRYDNHPDPAFGVARMDVMKDVMLGKDDKAWAMYVDNNRSWFMHN
NSHTNRTEGGITKGATIGVLLDLNRKNLTFFINDEQQGPIAFDNVEGLFFPAVSLNRNVQVTLHTGLPVP
DFYSSRASIA

>sp|Q9D020|Modified_Residue:123
MDRAAVARVGAVASASVCAVVAGVVLAQYIFTLKRKTGRKTKIIEMMPEFQKSSVRIKNPTRVEEIICGL
IKGGAAKLQIITDFDMTLSRFSYNGKRCPTCHNIIDNCKLVTDECRRKLLQLKEQYYAIEVDPVLTVEEK
FPYMVEWYTKSHGLLIEQGIPKAKLKEIVADSDVMLKEGYENFFGKLQQHGIPVFIFSAGIGDVLEEVIR
QAGVYHSNVKVVSNFMDFDENGVLKGFKGELIHVFNKHDGALKNTDYFSQLKDNSNIILLGDSQGDLRMA
DGVANVEHILKIGYLNDRVDELLEKYMDSYDIVLVKEESLEVVNSILQKTL

>sp|Q9DAY2|Modified_Residue:206
MALLLSQPHFSGPLLLLVVSNLLLWEKAASNLPCVAEEGGCWNPLLETFNSATQKAETLHNLADQLYVEL
YYNQFSSGQFWDFSSQIIRQDKTVVRAGSYCHSSLTNPPNTGVHINIEIASYLKTLINFVGSWISPLFHL
VIELSATKDVPETILSKAKEIEENNRQILSDLRWILTKVSPAAEMTEEFPHWEYLSFLKSSDKNNKFLAM
FNLSYCIDHDSKYILLQLRLLKCLITGKDC

>sp|Q9DB16|Modified_Residue:3
MKKMPLFSKSHKNPAEIVKILKDNLAILEKQDKKTDKASEEVSKSLQAMKEILCGTNDKEPPTEAVAQLA
QELYSSGLLVTLIADLQLIDFEGKKDVTQIFNNILRRQIGTRCPTVEYISSHPHILFMLLKGYEAPQIAL
RCGIMLRECIRHEPLAKIILFSNQFRDFFKYVELSTFDIASDAFATFKDLLTRHKVLVADFLEQNYDTIF
EDYEKLLQSENYVTKRQSLKLLGELILDRHNFTIMTKYISKPENLKLMMNLLRDKSPNIQFEAFHVFKVF
VASPHKTQPIVEILLKNQPKLIEFLSSFQKERTDDEQFADEKNYLIKQIRDLKKAAP

>sp|Q9EP89|Modified_Residue:383
MYRLLSSVTARAAATAGPAWDGGRRGAHRRPGLPVLGLGWAGGLGLGLGLALGAKLVVGLRGAVPIQSPA
DPEASGTTELSHEQALSPGSPHTPAPPAARGFSRAIESSRDLLHRIKDEVGAPGIVVGVSVDGKEVWSEG
LGYADVENRVPCKPETVMRIASISKSLTMVALAKLWEAGKLDLDLPVQHYVPEFPEKEYEGEKVSVTTRL
LISHLSGIRHYEKDIKKVKEEKAYKALKMVKGTPPPSDQEKELKEKGGKNNEKSDAPKAKVEQDSEARCR
SAKPGKKKNDFEQGELYLKEKFENSIESLRLFKNDPLFFKPGSQFLYSTFGYTLLAAIVERASGYKYLDY
MQKIFHDLDMLTTVQEENEPVIYNRARFYVYNKKKRLVNTPYVDNSYKWAGGGFLSTVGDLLKFGNAMLY
GYQVGQFKNSNENLLPGYLKPETMVMMWTPVPNTEMSWDKEGKYAMAWGVVEKKQTYGSCRKQRHYASHT
GGAVGASSVLLVLPEELDSEAVNNKVPPRGIIVSIICNMQSVGLNSTALKIALEFDKDRAD

>sp|Q9EQF5|Modified_Residue:256
MAPQGRLLIRGGRIVNDDFSQVADVLVEDGVVRALGRDLLPPEDASRGLRILDAAGKLVLPGGIDTHTHM
QFPFMGSQSVDDFYQGTKAALAGGTTMIIDFAIPQKGSSLIEAFETWRNWADPKVCCDYSLHVAVTWWSD
KVKEEMKTLARDKGVNSFKMFMAYKGLYMVQDEQLYAAFSQCKEIGAIAQVHAENGDLIAEGAKKMLALG
ITGPEGHELCRPEAVEAEATLRAITIASAVNCPLYVVHVMSKSAAKVVADARRAGNVVYGEPIAAGLGTD
GRQYWSEEWSHAAHHVMGPPLRPDPLTPGFLMDLLANGDLTTTGSDNCTFNTCQKALGKDDFTKIPNGVN
GVEDRMSVIWEKGVHSGKMDENRFVAVTSTNAAKIFNLYPKKGRIAVGSDADIVIWDPEATRRISAKTHH
QAVNFNIFEGMVCHGVPLVTISRGRVVYEAGVFNVTAGHGKFIPRQPFAEYIYKRIKQRDQTCTPVPVKR
APYKGEVTTLKARETKEDDTAGTRMQGHS

>sp|Q9EQP2|Modified_Residue:35
MFSWMGRQAGGRERSGGMDAVQTVTGGLRSLYQRKVLPLEEAYRFHEFHSPALEDADFENKPMILLVGQY
STGKTTFIRYLLEQDFPGMRIGPEPTTDSFIAVMYGETEGSTPGNALVVDPKKPFRKLSRFGNAFLNRFM
CSQLPNQVLKSISIIDSPGILSGEKQRISRGYDFCQVLQWFAERVDRIILLFDAHKLDISDEFSEAIKAF
RGQDDKIRVVLNKADQVDTQQLMRVYGALMWSLGKVINTPEVLRVYIGSFWAQPLQNTDNRRLFEAEAQD

LFRDIQSLPQKAAVRKLNDLIKRARLAKVHAYIISYLKKEMPNMFGKENKKRELIYRLPEIYVQLQREYQ
ISAGDFPEVKAMQEQLENYDFTKFHSLKPKLIEAVDNMLTNKISSLMGLISQEEMNMPTQMVQGGAFDGT
TEGPFNQGYGEGAKEGADEEEWVVAKDKPVYDELFYTLSPINGKISGVNAKKEMVTSKLPNSVLGKIWKL
ADCDCDGMLDEEEFALAKHLIKIKLDGYELPNSLPPHLVPPSHRKSLPKAD

>sp|Q9H1E3|Modified_Residue:228
MSRPVRNRKVVDYSQFQESDDADEDYGRDSGPPTKKIRSSPREAKNKRRSGKNSQEDSEDSEDKDVKTKK
DDSHSAEDSEDEKEDHKNVRQQRQAASKAASKQREMLMEDVGSEEEQEEEDEAPFQEKDSGSDEDFLMED
DDDSDYGSSKKKNKKMVKKSKPERKEKKMPKPRLKATVTPSPVKGKGKVGRPTASKASKEKTPSPKEEDE
EPESPPEKKTSTSPPPEKSGDEGSEDEAPSGED

>sp|Q9QXX4|Modified_Residue:177
MAAAKVALTKRADPAELKAIFLKYASIEKNGEFFMSPHDFVTRYLNIFGESQPNPKTVELLSGVVDQTKD
GLISFQEFVAFESVLCAPDALFMVAFQLFDKAGKGEVTFEDVKQIFGQTTIHQHIPFNWDSEFVQLHFGK
ERKRHLTYAEFTQFLLEIQLEHAKQAFVQRDNAKTGKVSAIDFRDIMVTIRPHVLTPFVEECLVAAAGGT
RSHQVSFSYFNGFNSLLNNMELIRKIYSTLAGNRKDVEVTKEEFALAAQKFGQVTPMEVDILFQLADLYE
PRGRMTLADIERIAPLEEGMLPFNLAEAQRQQKASGDAARPFLLQLAESAYRFGLGSIAGAVGATAVYPI
DLVKTRMQNQRSTGSFVGELMYKNSFDCFKKVLRYEGFFGLYRGLLPQLLGVAPEKAIKLTVNDFVRDKF
MHKDGSVPLLAEIFAGGCAGGSQVIFTNPLEIVKIRLQVAGEITTGPRVSALSVVRDLGFFGIYKGAKAC
FLRDIPFSAIYFPCYAHVKASFANEDGQVSPGSLLLAGAIAGMPAASLVTPADVIKTRLQVAARAGQTTY
NGVTDCFRKILREEGPKALWKGVAARVFRSSPQFGVTLLTYELLQRWFYVDFGGVKPVGSEPVPKSRITL
PAPNPDHVGGYKLAVATFAGIENKFGLYLPLFKPSASTSKVTAGDS

>sp|Q9Y597|Modified_Residue:796
MAGGHCGSFPAAAAGSGEIVQLNVGGTRFSTSRQTLMWIPDSFFSSLLSGRISTLRDETGAIFIDRDPAA
FAPILNFLRTKELDLRGVSINVLRHEAEFYGITPLVRRLLLCEELERSSCGSVLFHGYLPPPGIPSRKIN
NTVRSADSRNGLNSTEGEARGNGTQPVLSGTGEETVRLGFPVDPRKVLIVAGHHNWIVAAYAHFAVCYRI
KESSGWQQVFTSPYLDWTIERVALNAKVVGGPHGDKDKMVAVASESSIILWSVQDGGSGSEIGVFSLGVP
VDALFFIGNQLVATSHTGKVGVWNAVTQHWQVQDVVPITSYDTAGSFLLLGCNNGSIYYIDMQKFPLRMK
DNDLLVTELYHDPSNDAITALSVYLTPKTSVSGNWIEIAYGTSSGAVRVIVQHPETVGSGPQLFQTFTVH
RSPVTKIMLSEKHLVSVCADNNHVRTWTVTRFRGMISTQPGSTPLASFKILSLEETESHGSYSSGNDIGP
FGERDDQQVFIQKVVPITNKLFVRLSSTGKRICEIQAVDCTTISSFTVRECEGSSRMGSRPRRYLFTGHT
NGSIQMWDLTTAMDMVNKSEDKDVGGPTEEELLKLLDQCDLSTSRCATPNISPATSVVQHSHLRESNSSL
QLQHHDTTHEAATYGSMRPYRESPLLARARRTESFHSYRDFQTINLNRNVERAVPENGNLGPIQAEVKGA
TGECNISERKSPGVEIKSLRELDSGLEVHKIAEGFSESKKRSSEDENENKIEFRKKGGFEGGGFLGRKKV
PYLASSPSTSDGGTDSPGTASPSPTKTTPSPRHKKSDSSGQEYSL

>sp|Q9Y619|Modified_Residue:145
MKSNPAIQAAIDLTAGAAGGTACVLTGQPFDTMKVKMQTFPDLYRGLTDCCLKTYSQVGFRGFYKGTSPA
LIANIAENSVLFMCYGFCQQVVRKVAGLDKQAKLSDLQNAAAGSFASAFAALVLCPTELVKCRLQTMYEM
ETSGKIAKSQNTVWSVIKSILRKDGPLGFYHGLSSTLLREVPGYFFFFGGYELSRSFFASGRSKDELGPV
PLMLSGGVGGICLWLAVYPVDCIKSRIQVLSMSGKQAGFIRTFINVVKNEGITALYSGLKPTMIRAFPAN
GALFLAYEYSRKLMMNQLEAY

Segmented Sequences with Respective Protein ID and Positions:

#	PID:Position	Peptide Sequences	Labels
1.	A2A5N1:51	ELSNEERNLLSVAYKNVVGARRSSWRVIS	Positive
2.	A2AQC3:190	IREAVEEIRCYVRDKEMNSQVYSRLTSRG	Positive
3.	A6ZI44:254	IVEPEILPDGDHDLKRCQYVTEKVLAAVY	Positive
4.	A8DUK4:18	LTDAEKAAVSGLWGKVNADEVGGEALGRL	Positive
5.	A8DUK4:83	ITAFNDGLNHLDSLKGTFASLSELHCDKL	Positive
6.	A8DUK4:96	LKGTFASLSELHCDKLHVDPENFRLLGNM	Positive
7.	A8DUK4:145	FQKVVAGVAAALAHKYHXXXXXXXXXXXX	Positive
8.	B1AS29:367	HKAWRFGGRFMNFIKEAQWEGLTGRIVFN	Positive
9.	B1AU42:104	LQHYCHGLESKRLRKYLFQILRAIEYLHN	Positive
10.	B4DPF6:208	NNSNGHQLKDLILQKGITQNALDYMKKHI	Positive
11.	B8ZZL8:56	LQATVVAVGSGSKGKGGEIQPVSVKVGDK	Positive
12.	C9J5S8:23	SQEPQALLDSALPSKVPAFSDKDSLGDEM	Positive
13.	D3YU05:149	FVMGVNHEKYDNSLKIVSNASCTTNCLAP	Positive
14.	D3YU05:219	GAAQNIIPASTGAAKAVGKVIPELNGKLT	Positive
15.	D3YU05:223	NIIPASTGAAKAVGKVIPELNGKLTGMAF	Positive
16.	D3YU05:231	AAKAVGKVIPELNGKLTGMAFRVPTRNVS	Positive
17.	D3YU05:267	LEKHAKYDDIKKVVKQASEGPLKGILGYT	Positive
18.	D3Z041:544	ALDKDGWLHTGDIGKWLPNGTLKIIDRKK	Positive
19.	D3Z041:552	HTGDIGKWLPNGTLKIIDRKKHIFKLAQG	Positive
20.	D3Z563:31	NTDRPGLLDLKGKAKWDSWNKLKGTSKES	Positive
21.	D3Z6C3:144	TDGYLLRLFCVGFTKKRNNQIRKTSYAQH	Positive
22.	E7EP94:506	ANTLAEKDEFEHKRKELEQVCNPIISGLY	Positive
23.	E9PZS8:56	TKLTSSVTAYDYSGKNKVPELQKFFQPPF	Positive
24.	E9Q070:297	AATTAAPAAAAAPAKAEAKEESEESDEDM	Positive
25.	E9Q1V0:115	DAIKLNPRLAILYAKRASVFVKLQKPNAA	Positive
26.	E9Q3T0:98	TAAAPAEEKKVEAKKEESEESEDDMGFGL	Positive
27.	E9QMI7:675	MYGKPECLKLLLRSKPTVDIVNQNGETAL	Positive
28.	E9QNN1:1027	RKILTTEGRNALIHKSSVNCPFSSQDMKY	Positive
29.	F6UB20:63	QQVGTVSKVWIYPIKSCKGVSVCETECTD	Positive
30.	F6YLP3:48	RPKAKYIIWPRIDQKSCFKSMVTAGFEPV	Positive
31.	F8WIT2:442	LILGLMMPPAHYDAKQLKKAMEGAGTDEK	Positive
32.	F8WIT2:445	GLMMPPAHYDAKQLKKAMEGAGTDEKTLI	Positive
33.	F8WIT2:446	LMMPPAHYDAKQLKKAMEGAGTDEKTLIE	Positive
34.	G3UYJ7:249	IVIRFFPLEAWQIGKKGTXXXXXXXXXXX	Positive
35.	H3BTN5:367	LDGADCIMLSGETAKGDYPLEAVRMQHLI	Positive
36.	I7HPV9:971	VDRTDHSGIKKVCFKVSEDEQEDSGHDTM	Positive
37.	J3KMM5:205	TDPVPDPRAVNQDKKNMLFSGTNIAAGKA	Positive
38.	J3KRX8:91	RRSKEITVRGHNCPKPVLNFYEANFPANV	Positive
39.	J3QMG3:15	MCNTPTYCDLGKAAKDVFNKGYGFGMVKI	Positive
40.	J3QMG3:20	TYCDLGKAAKDVFNKGYGFGMVKIDLKTK	Positive
41.	J3QNG0:340	WHSPECEFVRHCIQKSQERVEGKVQVSVF	Positive
42.	O09172:263	RYSVIVKSRGIIKSKGYILQAKRRGSXXX	Positive
43.	O70250:100	RLNERHYGGLTGLNKAETAAKHGEEQVKI	Positive
44.	O88844:224	STKNTILKKYDGRFKDIFQEIYDKKYKSQ	Positive
45.	P01942:12	XXXMVLSGEDKSNIKAAWGKIGGHGAEYG	Positive
46.	P01942:17	LSGEDKSNIKAAWGKIGGHGAEYGAEALE	Positive
47.	P01942:57	YFPHFDVSHGSAQVKGHGKKVADALASAA	Positive
48.	P06733:335	TVTNPKRIAKAVNEKSCNCLLLKVNQIGS	Positive
49.	P06733:343	AKAVNEKSCNCLLLKVNQIGSVTESLQAC	Positive
50.	P07724:236	MKCSSMQKFGERAFKAWAVARLSQTFPNA	Positive

51.	P11214:167	LSLKPYNARRPNAIKLGLGNHNYCRNPDR	Positive
52.	P11881:569	LCYRVLRHSQQDYRKNQEYIAKQFGFMQK	Positive
53.	P11983:243	IVNAKIACLDFSLQKTKMKLGVQVVITDP	Positive
54.	P12710:96	VVKLEGDNKMVTTFKGIKSVTELNGDTIT	Positive
55.	P12790:237	FLKYFPGVHRQIVKKQQELLDYIAHSVEK	Positive
56.	P14174:78	SIGKIGGAQNRSYSKLLCGLLAERLRISP	Positive
57.	P16015:64	SYDPGSAKTILNNGKTCRVVFDDTYDRSM	Positive
58.	P16879:161	RDSTQARRKYQEASKDKDRDKAKDKYVRS	Positive
59.	P18894:259	WSGLNSVRDHNTIWKSCCKLEPTLKNARI	Positive
60.	P18894:263	NSVRDHNTIWKSCCKLEPTLKNARIVGEL	Positive
61.	P22752:96	RHLQLAIRNDEELNKLLGRVTIAQGGVLP	Positive
62.	P23881:199	SVRDKCVEMLSAALKAEDNFKDYGVNCDK	Positive
63.	P26040:60	GLQYVDNKGFPTWLKLDKKVSAQEVRKEN	Positive
64.	P26443:503	PTAEFQDRISGASEKDIVHSGLAYTMERS	Positive
65.	P35585:296	VIEKHSHSRIEYMVKAKSQFKRRSTANNV	Positive
66.	P47806:79	GSLFPPPPPPRSSVKLTKKRALSISPLSD	Positive
67.	P47857:678	QGGSPTPFDRNFATKMGAKAMNWMSGKIK	Positive
68.	P48962:92	VIRYFPTQALNFAFKDKYKQIFLGGVDRH	Positive
69.	P48962:96	FPTQALNFAFKDKYKQIFLGGVDRHKQFW	Positive
70.	P49006:144	EIGACSDEGTAQEGKAAATPESQEPQAKG	Positive
71.	P49722:92	GMGPDYRVLVHRARKLAQQYYLVYQEPIP	Positive
72.	P51814:297	NSCEHDHYEKHLSHKQAPTHHQKIHPEEK	Positive
73.	P60174:256	TRIIYGGSVTGATCKELASQPDVDGFLVG	Positive
74.	P62814:48	AVSRNYLSQPRLTYKTVSGVNGPLVILDH	Positive
75.	P62900:70	EMGTPDVRIDTRLNKAVWAKGIRNVPYRI	Positive
76.	P62908:201	KIMLPWDPSGKIGPKKPLPDHVSIVEPKD	Positive
77.	P68104:330	SVKDVRRGNVAGDSKNDPPMEAAGFTAQV	Positive
78.	P99028:83	LFDFLHARDHCVAHKLFKNLKXXXXXXXX	Positive
79.	Q13541:69	DRKFLMECRNSPVTKTPPRDLPTIPGVTS	Positive
80.	Q3UJB0:853	EKEDFSDMVAEHAAKQKQKKRKAQPQDSR	Positive
81.	Q3UL97:53	KTTVGTNIPNGHNQKMFSKNKENVKVMKV	Positive
82.	Q3UNI1:697	ATRQELTDVNYPSDKSXXXXXXXXXXXXX	Positive
83.	Q5JSZ5:386	WAGLHEEVDYSEKLKFSDDEEEEEVVKDG	Positive
84.	Q5JSZ5:398	KLKFSDDEEEEEVVKDGRPKWNSWDPRRQ	Positive
85.	Q5RL73:351	LKEVISSVPKPPEDKPEDVHTSHPLKQRR	Positive
86.	Q64FW2:55	EPLVTDKEARKKVLKQAFSVSRVPEKLDA	Positive
87.	Q6PKG0:1083	PPTPPTGQPVREDAKWTSQHSNTQTLGKX	Positive
88.	Q80ZV3:47	RDLPAIQPRLVAVSKTKPADMVIEAYGHG	Positive
89.	Q86UP2:292	ENAEVKFKDFLLSLKTMMFSEDEALCVVD	Positive
90.	Q8BH04:262	SFGSGYGGNSLLGKKCFALRIASRLARDE	Positive
91.	Q8BMS1:214	MMLTGRNIRADRAKKMGLVDQLVEPLGPG	Positive
92.	Q8C196:214	LIAEVSTKDVKVFGKGNPTKVVAVDCGIK	Positive
93.	Q8C196:228	KGNPTKVVAVDCGIKNNVIRLLVKRGAEV	Positive
94.	Q8C196:811	EVMAIGRTFEESFQKALRMCHPSVDGFTP	Positive
95.	Q8C196:1479	TNFQVTKLFAEAVQKSRTVDSKSLFHYRQ	Positive
96.	Q8VD72-2:33	KFQLCADLCTQMLEKSPYDQAAWILKARA	Positive
97.	Q91V76:75	EVGGVPYLLPLVNKKKVYDLNEIAKVIKL	Positive
98.	Q920E5:57	ARLKEVLEYNALGGKYNRGLTVVQAFQEL	Positive
99.	Q93092:314	EDQMAVEKLSDGIRKFAADAIKLERMLTE	Positive
100.	Q9BYV9:177	EEDEEEETMDSETAKMACPRDQMLPEPIS	Positive
101.	Q9C026:347	QLLARVNKEHEHKLKVVRDQISHCTVKLR	Positive
102.	Q9D020:123	KLVTDECRRKLLQLKEQYYAIEVDPVLTV	Positive
103.	Q9DAY2:206	WEYLSFLKSSDKNNKFLAMFNLSYCIDHD	Positive
104.	Q9DB16:3	XXXXXXXXXXXXMKKMPLFSKSHKNPAEI	Positive

105.	Q9EP89:383	EPVIYNRARFYVYNKKKRLVNTPYVDNSY	Positive
106.	Q9EQF5:256	CPLYVVHVMSKSAAKVVADARRAGNVVYG	Positive
107.	Q9EQP2:35	VQTVTGGLRSLYQRKVLPLEEAYRFHEFH	Positive
108.	Q9H1E3:228	SPPEKKTSTSPPPEKSGDEGSEDEAPSGE	Positive
109.	Q9QXX4:177	AKQAFVQRDNAKTGKVSAIDFRDIMVTIR	Positive
110.	Q9Y597:796	GGTDSPGTASPSPTKTTPSPRHKKSDSSG	Positive
111.	Q9Y619:145	KCRLQTMYEMETSGKIAKSQNTVWSVIKS	Positive
112.	A2A5N1:5	XXXXXXXXXXMTMDKSELVQKAKLAEQAE	Negative
113.	A2A5N1:11	XXXXMTMDKSELVQKAKLAEQAERYDDMA	Negative
114.	A2A5N1:13	XXMTMDKSELVQKAKLAEQAERYDDMAAA	Negative
115.	A2A5N1:29	AEQAERYDDMAAAMKAVTEQGHELSNEER	Negative
116.	A2A5N1:70	ARRSSWRVISSIEQKTERNEKKQQMGKEY	Negative
117.	A2A5N1:76	RVISSIEQKTERNEKKQQMGKEYREKIEA	Negative
118.	A2A5N1:77	VISSIEQKTERNEKKQQMGKEYREKIEAE	Negative
119.	A2A5N1:82	EQKTERNEKKQQMGKEYREKIEAELQDIC	Negative
120.	A2A5N1:87	RNEKKQQMGKEYREKIEAELQDICNDVLE	Negative
121.	A2A5N1:105	ELQDICNDVLELLDKYLILNATQAESKVF	Negative
122.	A2A5N1:117	LDKYLILNATQAESKVFYLKMKGDYFRYL	Negative
123.	A2A5N1:122	ILNATQAESKVFYLKMKGDYFRYLSEVAS	Negative
124.	A2A5N1:124	NATQAESKVFYLKMKGDYFRYLSEVASGE	Negative
125.	A2A5N1:140	DYFRYLSEVASGENKQTTVSNSQQAYQEA	Negative
126.	A2A5N1:159	SNSQQAYQEAFEISKXXXXXXXXXXXXXX	Negative
127.	A2AQC3:71	GDMTDSIPLQPVRHKKRVDSRPRAGCCEW	Negative
128.	A2AQC3:72	DMTDSIPLQPVRHKKRVDSRPRAGCCEWL	Negative
129.	A2AQC3:106	GEPRPRTVWLGHPEKRDQRYPRNVINNQK	Negative
130.	A2AQC3:120	KRDQRYPRNVINNQKYNFFTFLPGVLFSQ	Negative
131.	A2AQC3:207	NSQVYSRLTSRGTVKVKSSNIQVGDLILV	Negative
132.	A2AQC3:209	QVYSRLTSRGTVKVKSSNIQVGDLILVEK	Negative
133.	A2AQC3:223	KSSNIQVGDLILVEKNQRVPADMIFLRTS	Negative
134.	A2AQC3:239	QRVPADMIFLRTSEKNGSCFLRTDQLDGE	Negative
135.	A2AQC3:257	CFLRTDQLDGETDWKLRLPVACTQRLPTA	Negative
136.	A2AQC3:350	RELRSVMNTSDPRSKIGLFDLEVNCLTKI	Negative
137.	A2AQC3:363	SKIGLFDLEVNCLTKILFGALVVVSLVMV	Negative
138.	A2AQC3:413	NIIPISLRVNLDMGKIVYSWVIRRDSKIP	Negative
139.	A2AQC3:425	MGKIVYSWVIRRDSKIPGTVVRSSTIPEQ	Negative
140.	A2AQC3:450	IPEQLGRISYLLTDKTGTLTQNEMVFKRL	Negative
141.	A2AQC3:462	TDKTGTLTQNEMVFKRLHLGTVAYGLDSM	Negative
142.	A2AQC3:498	FSIYTQQSQDPPAQKGPTVTTKVRRTMSS	Negative
143.	A2AQC3:505	SQDPPAQKGPTVTTKVRRTMSSRVHEAVK	Negative
144.	A2AQC3:519	KVRRTMSSRVHEAVKAIALCHNVTPVYES	Negative
145.	A2AQC3:544	VYESNGVTDQAEAEKQFEDSCRVYQASSP	Negative
146.	A2AQC3:607	NLTILQVFPFTYESKRMGIIVRDESTGEI	Negative
147.	A2AQC3:626	IVRDESTGEITFYMKGADVVMAGIVQYND	Negative
148.	A2AQC3:661	GNMAREGLRVLVVAKKSLTEEQYQDFEAR	Negative
149.	A2AQC3:662	NMAREGLRVLVVAKKSLTEEQYQDFEARY	Negative
150.	A2AQC3:680	EEQYQDFEARYVQAKLSVHDRSLKVATVI	Negative
151.	A2AQC3:689	RYVQAKLSVHDRSLKVATVIESLEMEMEL	Negative
152.	A2AQC3:730	DVRPTLETLRNAGIKVWMLTGDKLETATC	Negative
153.	A2AQC3:738	LRNAGIKVWMLTGDKLETATCTAKNAHLV	Negative
154.	A2AQC3:747	MLTGDKLETATCTAKNAHLVTRNQDIHVF	Negative
155.	A2AQC3:780	NRGEAHLELNAFRRKHDCALVISGDSLEV	Negative
156.	A2AQC3:797	CALVISGDSLEVCLKYYEYEFMELACQCP	Negative
157.	A2AQC3:823	QCPAVVCCRCAPTQKAQIVRLLQERTGKL	Negative
158.	A2AQC3:836	QKAQIVRLLQERTGKLTCAVGDGGNDVSM	Negative

159.	A2AQC3:863	SMIQESDCGVGVEGKEGKQASLAADFSIT	Negative
160.	A2AQC3:866	QESDCGVGVEGKEGKQASLAADFSITQFK	Negative
161.	A2AQC3:880	KQASLAADFSITQFKHLGRLLMVHGRNSY	Negative
162.	A2AQC3:895	HLGRLLMVHGRNSYKRSAALSQFVIHRSL	Negative
163.	A2AQC3:954	TIYTMFPVFSLVLDKDVKSEVAMLYPELY	Negative
164.	A2AQC3:957	TMFPVFSLVLDKDVKSEVAMLYPELYKDL	Negative
165.	A2AQC3:969	DVKSEVAMLYPELYKDLLKGRPLSYKTFL	Negative
166.	A2AQC3:973	EVAMLYPELYKDLLKGRPLSYKTFLIWVL	Negative
167.	A2AQC3:980	ELYKDLLKGRPLSYKTFLIWVLISIYQGS	Negative
168.	A2AQC3:1073	FIDVYFIATLSFLWKVSVITLVSCLPLYV	Negative
169.	A2AQC3:1089	SVITLVSCLPLYVLKYLRRRFSPPSYSKL	Negative
170.	A2AQC3:1102	LKYLRRRFSPPSYSKLTSXXXXXXXXXXX	Negative
171.	A6ZI44:47	HSELGNTQQQTELGKESTATGTMPHPYPA	Negative
172.	A6ZI44:67	GTMPHPYPALTPEQKKELSDIAHRIVAPG	Negative
173.	A6ZI44:68	TMPHPYPALTPEQKKELSDIAHRIVAPGK	Negative
174.	A6ZI44:82	KELSDIAHRIVAPGKGILAADESTGSIAK	Negative
175.	A6ZI44:96	KGILAADESTGSIAKRLQSIGTENTEENR	Negative
176.	A6ZI44:141	CIGGVILFHETLYQKADDGRPFPQVIKSK	Negative
177.	A6ZI44:153	YQKADDGRPFPQVIKSKGGVVGIKVDKGV	Negative
178.	A6ZI44:155	KADDGRPFPQVIKSKGGVVGIKVDKGVVP	Negative
179.	A6ZI44:162	FPQVIKSKGGVVGIKVDKGVVPLAGTNGE	Negative
180.	A6ZI44:165	VIKSKGGVVGIKVDKGVVPLAGTNGETTT	Negative
181.	A6ZI44:193	TQGLDGLSERCAQYKKDGADFAKWRCVLK	Negative
182.	A6ZI44:194	QGLDGLSERCAQYKKDGADFAKWRCVLKI	Negative
183.	A6ZI44:201	ERCAQYKKDGADFAKWRCVLKIGEHTPSA	Negative
184.	A6ZI44:207	KKDGADFAKWRCVLKIGEHTPSALAIMEN	Negative
185.	A6ZI44:262	DGDHDLKRCQYVTEKVLAAVYKALSDHHV	Negative
186.	A6ZI44:269	RCQYVTEKVLAAVYKALSDHHVYLEGTLL	Negative
187.	A6ZI44:284	ALSDHHVYLEGTLLKPNMVTPGHACTQKF	Negative
188.	A6ZI44:297	LKPNMVTPGHACTQKFSNEEIAMATVTAL	Negative
189.	A6ZI44:343	QSEEEASINLNAINKCPLLKPWALTFSYG	Negative
190.	A6ZI44:348	ASINLNAINKCPLLKPWALTFSYGRALQA	Negative
191.	A6ZI44:366	LTFSYGRALQASALKAWGGKKENLKAAQE	Negative
192.	A6ZI44:371	GRALQASALKAWGGKKENLKAAQEEYIKR	Negative
193.	A6ZI44:372	RALQASALKAWGGKKENLKAAQEEYIKRA	Negative
194.	A6ZI44:376	ASALKAWGGKKENLKAAQEEYIKRALANS	Negative
195.	A6ZI44:384	GKKENLKAAQEEYIKRALANSLACQGKYT	Negative
196.	A6ZI44:396	YIKRALANSLACQGKYTPSGQSGAAASES	Negative
197.	A8DUK4:9	XXXXXXMVHLTDAEKAAVSGLWGKVNADE	Negative
198.	A8DUK4:60	FGDLSSASAIMGNAKVKAHGKKVITAFND	Negative
199.	A8DUK4:62	DLSSASAIMGNAKVKAHGKKVITAFNDGL	Negative
200.	A8DUK4:66	ASAIMGNAKVKAHGKKVITAFNDGLNHLD	Negative
201.	A8DUK4:67	SAIMGNAKVKAHGKKVITAFNDGLNHLDS	Negative
202.	A8DUK4:121	LGNMIVIVLGHHLGKDFTPAAQAAFQKVV	Negative
203.	A8DUK4:133	LGKDFTPAAQAAFQKVVAGVAAALAHKYH	Negative
204.	B1AS29:96	DIQRIHFHDSFEATKKACDQLALGVVAIF	Negative
205.	B1AS29:97	IQRIHFHDSFEATKKACDQLALGVVAIFG	Negative
206.	B1AS29:138	ICNALEVPHIQLRWKHHPLDNKDTFYVNL	Negative
207.	B1AS29:145	PHIQLRWKHHPLDNKDTFYVNLYPDYASL	Negative
208.	B1AS29:171	ASLSHAILDLVQSLKWRSATVVYDDSTGL	Negative
209.	B1AS29:203	QELIMAPSRYNIRLKIRQLPIDSDDSRPL	Negative
210.	B1AS29:219	RQLPIDSDDSRPLLKEMKRGREFRIIFDC	Negative
211.	B1AS29:222	PIDSDDSRPLLKEMKRGREFRIIFDCSHT	Negative
212.	B1AS29:243	IIFDCSHTMAAQILKQAMAMGMMTEYYHF	Negative

213.	B1AS29:298	ILNVDNPHVSAIVEKWAMERLQAAPRAES	Negative
214.	B1AS29:354	APQMTVNSLQCHRHKAWRFGGRFMNFIKE	Negative
215.	B1AS29:382	EAQWEGLTGRIVFNKTSGLRTDFDLDIIS	Negative
216.	B1AS29:398	SGLRTDFDLDIISLKEDGLEKVGVWSPAD	Negative
217.	B1AS29:404	FDLDIISLKEDGLEKVGVWSPADGLNITE	Negative
218.	B1AS29:421	VWSPADGLNITEVAKGRGPNVTDSLTNRS	Negative
219.	B1AS29:451	IVTTVLEEPFVMFRKSDRTLYGNDRFEGY	Negative
220.	B1AS29:471	YGNDRFEGYCIDLLKELAHILGFSYEIRL	Negative
221.	B1AS29:490	ILGFSYEIRLVEDGKYGAQDDKGQWNGMV	Negative
222.	B1AS29:497	IRLVEDGKYGAQDDKGQWNGMVKELIDHK	Negative
223.	B1AS29:505	YGAQDDKGQWNGMVKELIDHKADLAVAPL	Negative
224.	B1AS29:511	KGQWNGMVKELIDHKADLAVAPLTITHVR	Negative
225.	B1AS29:527	DLAVAPLTITHVREKAIDFSKPFMTLGVS	Negative
226.	B1AS29:533	LTITHVREKAIDFSKPFMTLGVSILYRKP	Negative
227.	B1AS29:546	SKPFMTLGVSILYRKPNGTNPSVFSFLNP	Negative
228.	B1AS29:631	GMGSLMQQGSELMPKALSTRIIGGIWWFF	Negative
229.	B1AS29:678	ERMESPIDSADDLAKQTKIEYGAVKDGAT	Negative
230.	B1AS29:681	ESPIDSADDLAKQTKIEYGAVKDGATMTF	Negative
231.	B1AS29:688	DDLAKQTKIEYGAVKDGATMTFFKKSKIS	Negative
232.	B1AS29:697	EYGAVKDGATMTFFKKSKISTFEKMWAFM	Negative
233.	B1AS29:698	YGAVKDGATMTFFKKSKISTFEKMWAFMS	Negative
234.	B1AS29:700	AVKDGATMTFFKKSKISTFEKMWAFMSSK	Negative
235.	B1AS29:706	TMTFFKKSKISTFEKMWAFMSSKPSALVK	Negative
236.	B1AS29:714	KISTFEKMWAFMSSKPSALVKNNEEGIQR	Negative
237.	B1AS29:720	KMWAFMSSKPSALVKNNEEGIQRTLTADY	Negative
238.	B1AS29:763	RNCNLTQIGGLIDSKGYGIGTPMGSPYRD	Negative
239.	B1AS29:778	GYGIGTPMGSPYRDKITIAILQLQEEDKL	Negative
240.	B1AS29:791	DKITIAILQLQEEDKLHIMKEKWWRGSGC	Negative
241.	B1AS29:796	AILQLQEEDKLHIMKEKWWRGSGCPEEEN	Negative
242.	B1AS29:798	LQLQEEDKLHIMKEKWWRGSGCPEEENKE	Negative
243.	B1AS29:811	EKWWRGSGCPEEENKEASALGIQKIGGIF	Negative
244.	B1AS29:820	PEEENKEASALGIQKIGGIFIVLAAGLVL	Negative
245.	B1AS29:846	LVLSVLVAVGEFIYKLRKTAEREQRSFCS	Negative
246.	B1AS29:849	SVLVAVGEFIYKLRKTAEREQRSFCSTVA	Negative
247.	B1AS29:877	ADEIRFSLTCQRRLKHKPQPPMMVKTDAV	Negative
248.	B1AS29:879	EIRFSLTCQRRLKHKPQPPMMVKTDAVIN	Negative
249.	B1AS29:887	QRRLKHKPQPPMMVKTDAVINMHTFNDRR	Negative
250.	B1AS29:905	VINMHTFNDRRLPGKDSMSCSTSLAPVFP	Negative
251.	B1AU42:9	XXXXXXMEMYETLGKVGEGSYGTVMKCKH	Negative
252.	B1AU42:20	TLGKVGEGSYGTVMKCKHKDTGRIVAIKI	Negative
253.	B1AU42:22	GKVGEGSYGTVMKCKHKDTGRIVAIKIFY	Negative
254.	B1AU42:24	VGEGSYGTVMKCKHKDTGRIVAIKIFYEK	Negative
255.	B1AU42:33	MKCKHKDTGRIVAIKIFYEKPEKSVNKIA	Negative
256.	B1AU42:38	KDTGRIVAIKIFYEKPEKSVNKIATREIK	Negative
257.	B1AU42:41	GRIVAIKIFYEKPEKSVNKIATREIKFLK	Negative
258.	B1AU42:45	AIKIFYEKPEKSVNKIATREIKFLKQFRH	Negative
259.	B1AU42:52	KPEKSVNKIATREIKFLKQFRHENLVNLI	Negative
260.	B1AU42:55	KSVNKIATREIKFLKQFRHENLVNLIEVF	Negative
261.	B1AU42:72	RHENLVNLIEVFRQKKKIHLVFEFIDHTV	Negative
262.	B1AU42:73	HENLVNLIEVFRQKKKIHLVFEFIDHTVL	Negative
263.	B1AU42:74	ENLVNLIEVFRQKKKIHLVFEFIDHTVLD	Negative
264.	B1AU42:100	VLDELQHYCHGLESKRLRKYLFQILRAIE	Negative
265.	B1AU42:127	IEYLHNNNIIHRDIKPENILVSQSGITKL	Negative
266.	B1AU42:140	IKPENILVSQSGITKLCDFGFARTLAAPG	Negative

267.	B1AU42:174	YVATRWYRAPELVLKDTSYGKPVDIWALG	Negative
268.	B1AU42:180	YRAPELVLKDTSYGKPVDIWALGCMIIXX	Negative
269.	B4DPF6:37	DSTTDEEEDEEEVYKMAGVMAQCGGLECM	Negative
270.	B4DPF6:62	LECMLNRLAGIRDFKQGRHLLTVLLKLFS	Negative
271.	B4DPF6:73	RDFKQGRHLLTVLLKLFSYCVKVKVNRQQ	Negative
272.	B4DPF6:80	HLLTVLLKLFSYCVKVKVNRQQLVKLEMN	Negative
273.	B4DPF6:82	LTVLLKLFSYCVKVKVNRQQLVKLEMNTL	Negative
274.	B4DPF6:90	SYCVKVKVNRQQLVKLEMNTLNVMLGTLN	Negative
275.	B4DPF6:114	LGTLNLGNLLLTGDKDQLVMLLDQINSTF	Negative
276.	B4DPF6:153	LLRIIPYLSFGEVEKMQILVERFKPYCNF	Negative
277.	B4DPF6:162	FGEVEKMQILVERFKPYCNFDKYDEDHSG	Negative
278.	B4DPF6:169	QILVERFKPYCNFDKYDEDHSGDDKVFLD	Negative
279.	B4DPF6:179	CNFDKYDEDHSGDDKVFLDCFCKIAAGIK	Negative
280.	B4DPF6:187	DHSGDDKVFLDCFCKIAAGIKNNSNGHQL	Negative
281.	B4DPF6:193	KVFLDCFCKIAAGIKNNSNGHQLKDLILQ	Negative
282.	B4DPF6:202	IAAGIKNNSNGHQLKDLILQKGITQNALD	Negative
283.	B4DPF6:219	ILQKGITQNALDYMKKHIPSAKNLDADIW	Negative
284.	B4DPF6:220	LQKGITQNALDYMKKHIPSAKNLDADIWK	Negative
285.	B4DPF6:226	QNALDYMKKHIPSAKNLDADIWKKFLSRP	Negative
286.	B4DPF6:234	KHIPSAKNLDADIWKKFLSRPALPFILRL	Negative
287.	B4DPF6:235	HIPSAKNLDADIWKKFLSRPALPFILRLL	Negative
288.	B4DPF6:273	TQVLIGTDSIPNLHKLEQVSSDEGIGTLA	Negative
289.	B4DPF6:302	ENLLEALREHPDVNKKIDAARRETRAEKK	Negative
290.	B4DPF6:303	NLLEALREHPDVNKKIDAARRETRAEKKR	Negative
291.	B4DPF6:315	NKKIDAARRETRAEKKRMAMAMRQKALGT	Negative
292.	B4DPF6:316	KKIDAARRETRAEKKRMAMAMRQKALGTL	Negative
293.	B4DPF6:325	TRAEKKRMAMAMRQKALGTLGMTTNEKGQ	Negative
294.	B4DPF6:337	RQKALGTLGMTTNEKGQVVTKTALLKQME	Negative
295.	B4DPF6:343	TLGMTTNEKGQVVTKTALLKQMEELIEEP	Negative
296.	B4DPF6:348	TNEKGQVVTKTALLKQMEELIEEPGLTCC	Negative
297.	B4DPF6:369	EEPGLTCCICREGYKFQPTKVLGIYTFTK	Negative
298.	B4DPF6:374	TCCICREGYKFQPTKVLGIYTFTKRVALE	Negative
299.	B4DPF6:383	KFQPTKVLGIYTFTKRVALEEMENKPRKQ	Negative
300.	B4DPF6:393	YTFTKRVALEEMENKPRKQQGYSTVSHFN	Negative
301.	B4DPF6:396	TKRVALEEMENKPRKQQGYSTVSHFNIVH	Negative
302.	B4DPF6:438	REEWESAALQNANTKCNGLLPVWGPHVPE	Negative
303.	B4DPF6:485	GQREPTYQLNIHDIKLLFLRFAMEQSFSA	Negative
304.	B4DPF6:535	LYVLNTTRATSREEKNLQGFLEQPKEKWV	Negative
305.	B4DPF6:545	SREEKNLQGFLEQPKEKWVESAFEVDGPY	Negative
306.	B4DPF6:547	EEKNLQGFLEQPKEKWVESAFEVDGPYYF	Negative
307.	B4DPF6:605	ARAVAPGGATRLTDKAVKDYSAYRSSLLF	Negative
308.	B4DPF6:608	VAPGGATRLTDKAVKDYSAYRSSLLFWAL	Negative
309.	B4DPF6:631	LLFWALVDLIYNMFKKVPTSNTEGGWSCS	Negative
310.	B4DPF6:632	LFWALVDLIYNMFKKVPTSNTEGGWSCSL	Negative
311.	B4DPF6:663	YIRHNDMPIYEAADKALKTFQEEFMPVET	Negative
312.	B4DPF6:666	HNDMPIYEAADKALKTFQEEFMPVETFSE	Negative
313.	B4DPF6:699	AGLLSEITDPESFLKDLLNSVPXXXXXXX	Negative
314.	B8ZZL8:8	XXXXXXXMAGQAFRKFLPLFDRVLVERSA	Negative
315.	B8ZZL8:28	DRVLVERSAAETVTKGGIMLPEKSQGKVL	Negative
316.	B8ZZL8:36	AAETVTKGGIMLPEKSQGKVLQATVVAVG	Negative
317.	B8ZZL8:40	VTKGGIMLPEKSQGKVLQATVVAVGSGSK	Negative
318.	B8ZZL8:54	KVLQATVVAVGSGSKGKGGEIQPVSVKVG	Negative
319.	B8ZZL8:66	GSKGKGGEIQPVSVKVGDKVLLPEYGGTK	Negative
320.	B8ZZL8:70	KGGEIQPVSVKVGDKVLLPEYGGTKVVLD	Negative

321.	B8ZZL8:80	KVGDKVLLPEYGGTKVVLDDKVCKLNNSK	Negative
322.	B8ZZL8:86	LLPEYGGTKVVLDDKVCKLNNSKKKSDIC	Negative
323.	B8ZZL8:89	EYGGTKVVLDDKVCKLNNSKKKSDICNXX	Negative
324.	B8ZZL8:94	KVVLDDKVCKLNNSKKKSDICNXXXXXXX	Negative
325.	B8ZZL8:95	VVLDDKVCKLNNSKKKSDICNXXXXXXXX	Negative
326.	B8ZZL8:96	VLDDKVCKLNNSKKKSDICNXXXXXXXXX	Negative
327.	C9J5S8:30	LDSALPSKVPAFSDKDSLGDEMLAAALLK	Negative
328.	C9J5S8:44	KDSLGDEMLAAALLKAKSQELVTFEDVAV	Negative
329.	C9J5S8:46	SLGDEMLAAALLKAKSQELVTFEDVAVYF	Negative
330.	C9J5S8:63	ELVTFEDVAVYFIRKEWKRLEPAQRDLYR	Negative
331.	C9J5S8:66	TFEDVAVYFIRKEWKRLEPAQRDLYRDVM	Negative
332.	C9J5S8:118	EDTRSHGVLLGRFQKDISQGLKFKEAYER	Negative
333.	C9J5S8:125	VLLGRFQKDISQGLKFKEAYEREVSLKRP	Negative
334.	C9J5S8:127	LGRFQKDISQGLKFKEAYEREVSLKRPLG	Negative
335.	C9J5S8:137	GLKFKEAYEREVSLKRPLGNSPGERLNRK	Negative
336.	C9J5S8:151	KRPLGNSPGERLNRKMPDFGQVTVEEKLT	Negative
337.	C9J5S8:163	NRKMPDFGQVTVEEKLTPRGERSEKYNDF	Negative
338.	C9J5S8:173	TVEEKLTPRGERSEKYNDFGNSFTVNSXX	Negative
339.	D3YU05:3	XXXXXXXXXXXXMVKVGVNGFGRIGRLVT	Negative
340.	D3YU05:25	RIGRLVTRAAVCSGKISVEIVAINDPFID	Negative
341.	D3YU05:55	NYMVYLFQSDSTHGKFNRTVQAENGKLVI	Negative
342.	D3YU05:66	THGKFNRTVQAENGKLVINGKPITIFQER	Negative
343.	D3YU05:72	RTVQAENGKLVINGKPITIFQERDTPPPL	Negative
344.	D3YU05:90	IFQERDTPPPLANIKWGDAGADYVVESTG	Negative
345.	D3YU05:111	DYVVESTGVFTTMEKAGAHLKGGAKRVII	Negative
346.	D3YU05:117	TGVFTTMEKAGAHLKGGAKRVIISAPSAD	Negative
347.	D3YU05:121	TTMEKAGAHLKGGAKRVIISAPSADAPMF	Negative
348.	D3YU05:143	SADAPMFVMGVNHEKYDNSLKIVSNASCT	Negative
349.	D3YU05:166	SNASCTTNCLAPLAKVIHDNFGIMEGLMT	Negative
350.	D3YU05:190	EGLMTTVHAITATQKTVDGPSGKLWRDGR	Negative
351.	D3YU05:198	AITATQKTVDGPSGKLWRDGRGAAQNIIP	Negative
352.	D3YU05:255	TRNVSVVDLTCRLEKHAKYDDIKKVVKQA	Negative
353.	D3YU05:258	VSVVDLTCRLEKHAKYDDIKKVVKQASEG	Negative
354.	D3YU05:263	LTCRLEKHAKYDDIKKVVKQASEGPLKGI	Negative
355.	D3YU05:264	TCRLEKHAKYDDIKKVVKQASEGPLKGIL	Negative
356.	D3YU05:275	DIKKVVKQASEGPLKGILGYTEDQVVSCD	Negative
357.	D3YU05:313	FDAGAGIALNDNFVKLISWYDNEYGYSNR	Negative
358.	D3YU05:338	YSNRMVDLMAYMASKEXXXXXXXXXXXXX	Negative
359.	D3Z041:49	AFAALTTFWYATRPKALKPPCDLSMQSVE	Negative
360.	D3Z041:52	ALTTFWYATRPKALKPPCDLSMQSVEIAG	Negative
361.	D3Z041:81	TTDGIRRSAVLEDDKLLVYYYDDVRTMYD	Negative
362.	D3Z041:114	GIQVSNNGPCLGSRKPNQPYEWISYKEVA	Negative
363.	D3Z041:125	GSRKPNQPYEWISYKEVAELAECIGSGLI	Negative
364.	D3Z041:141	VAELAECIGSGLIQKGFKPCSEQFIGLFS	Negative
365.	D3Z041:144	LAECIGSGLIQKGFKPCSEQFIGLFSQNR	Negative
366.	D3Z041:193	YDTLGADAITYIVNKAELSVIFADKPEKA	Negative
367.	D3Z041:203	YIVNKAELSVIFADKPEKAKLLLEGVENK	Negative
368.	D3Z041:206	NKAELSVIFADKPEKAKLLLEGVENKLTP	Negative
369.	D3Z041:208	AELSVIFADKPEKAKLLLEGVENKLTPCL	Negative
370.	D3Z041:217	KPEKAKLLLEGVENKLTPCLKIIVIMDSY	Negative
371.	D3Z041:223	LLLEGVENKLTPCLKIIVIMDSYGSDLVE	Negative
372.	D3Z041:240	VIMDSYGSDLVERGKKCGVEIISLKALED	Negative
373.	D3Z041:241	IMDSYGSDLVERGKKCGVEIISLKALEDL	Negative
374.	D3Z041:250	VERGKKCGVEIISLKALEDLGRVNRVKPK	Negative

375.	D3Z041:262	SLKALEDLGRVNRVKPKPPEPEDLAIICF	Negative
376.	D3Z041:264	KALEDLGRVNRVKPKPPEPEDLAIICFTS	Negative
377.	D3Z041:285	LAIICFTSGTTGNPKGAMITHQNIINDCS	Negative
378.	D3Z041:303	ITHQNIINDCSGFIKATESALTLNASDTQ	Negative
379.	D3Z041:341	YEQQLQCVMLCHGAKIGFFQGDIRLLMDD	Negative
380.	D3Z041:357	GFFQGDIRLLMDDLKVLQPTIFPVVPRLL	Negative
381.	D3Z041:387	RMFDRIFGQANTSLKRWLLDFASKRKEAE	Negative
382.	D3Z041:396	ANTSLKRWLLDFASKRKEAELRSGIVRNN	Negative
383.	D3Z041:398	TSLKRWLLDFASKRKEAELRSGIVRNNSL	Negative
384.	D3Z041:415	ELRSGIVRNNSLWDKLIFHKIQSSLGGKV	Negative
385.	D3Z041:420	IVRNNSLWDKLIFHKIQSSLGGKVRLMIT	Negative
386.	D3Z041:428	DKLIFHKIQSSLGGKVRLMITGAAPVSAT	Negative
387.	D3Z041:491	TAGHVGAPMPCNYVKLVDVEEMNYLASKG	Negative
388.	D3Z041:504	VKLVDVEEMNYLASKGEGEVCVKGANVFK	Negative
389.	D3Z041:512	MNYLASKGEGEVCVKGANVFKGYLKDPAR	Negative
390.	D3Z041:518	KGEGEVCVKGANVFKGYLKDPARTAEALD	Negative
391.	D3Z041:522	EVCVKGANVFKGYLKDPARTAEALDKDGW	Negative
392.	D3Z041:533	GYLKDPARTAEALDKDGWLHTGDIGKWLP	Negative
393.	D3Z041:557	GKWLPNGTLKIIDRKKHIFKLAQGEYIAP	Negative
394.	D3Z041:558	KWLPNGTLKIIDRKKHIFKLAQGEYIAPE	Negative
395.	D3Z041:562	NGTLKIIDRKKHIFKLAQGEYIAPEKIEN	Negative
396.	D3Z041:573	HIFKLAQGEYIAPEKIENIYLRSEAVAQV	Negative
397.	D3Z041:615	VVVPDVESLPSWAQKRGLQGSFEELCRNK	Negative
398.	D3Z041:629	KRGLQGSFEELCRNKDINKAILDDLLKLG	Negative
399.	D3Z041:633	QGSFEELCRNKDINKAILDDLLKLGKEAG	Negative
400.	D3Z041:641	RNKDINKAILDDLLKLGKEAGLKPFEQVK	Negative
401.	D3Z041:644	DINKAILDDLLKLGKEAGLKPFEQVKGIA	Negative
402.	D3Z041:649	ILDDLLKLGKEAGLKPFEQVKGIAVHPEL	Negative
403.	D3Z041:655	KLGKEAGLKPFEQVKGIAVHPELFSIDNG	Negative
404.	D3Z041:676	ELFSIDNGLLTPTLKAKRPELRNYFRSQI	Negative
405.	D3Z041:678	FSIDNGLLTPTLKAKRPELRNYFRSQIDE	Negative
406.	D3Z041:698	NYFRSQIDELYATIKIXXXXXXXXXXXXX	Negative
407.	D3Z563:9	XXXXXXMLFIYSHFKQATVGDVNTDRPGL	Negative
408.	D3Z563:27	VGDVNTDRPGLLDLKGKAKWDSWNKLKGT	Negative
409.	D3Z563:29	DVNTDRPGLLDLKGKAKWDSWNKLKGTSK	Negative
410.	D3Z563:37	LLDLKGKAKWDSWNKLKGTSKESAMKTYV	Negative
411.	D3Z563:39	DLKGKAKWDSWNKLKGTSKESAMKTYVEK	Negative
412.	D3Z563:43	KAKWDSWNKLKGTSKESAMKTYVEKVDEL	Negative
413.	D3Z563:48	SWNKLKGTSKESAMKTYVEKVDELKKKYG	Negative
414.	D3Z563:53	KGTSKESAMKTYVEKVDELKKKYGIXXXX	Negative
415.	D3Z563:58	ESAMKTYVEKVDELKKKYGIXXXXXXXXX	Negative
416.	D3Z563:59	SAMKTYVEKVDELKKKYGIXXXXXXXXXX	Negative
417.	D3Z563:60	AMKTYVEKVDELKKKYGIXXXXXXXXXXX	Negative
418.	D3Z6C3:5	XXXXXXXXXXMAVGKNKRLTKGGKKGAKK	Negative
419.	D3Z6C3:7	XXXXXXXXMAVGKNKRLTKGGKKGAKKKV	Negative
420.	D3Z6C3:11	XXXXMAVGKNKRLTKGGKKGAKKKVVDPF	Negative
421.	D3Z6C3:14	XMAVGKNKRLTKGGKKGAKKKVVDPFSKK	Negative
422.	D3Z6C3:15	MAVGKNKRLTKGGKKGAKKKVVDPFSKKD	Negative
423.	D3Z6C3:18	GKNKRLTKGGKKGAKKKVVDPFSKKDWYD	Negative
424.	D3Z6C3:19	KNKRLTKGGKKGAKKKVVDPFSKKDWYDV	Negative
425.	D3Z6C3:20	NKRLTKGGKKGAKKKVVDPFSKKDWYDVK	Negative
426.	D3Z6C3:27	GKKGAKKKVVDPFSKKDWYDVKAPAMFNI	Negative
427.	D3Z6C3:28	KKGAKKKVVDPFSKKDWYDVKAPAMFNIR	Negative
428.	D3Z6C3:34	KVVDPFSKKDWYDVKAPAMFNIRNIGKTL	Negative

429.	D3Z6C3:46	DVKAPAMFNIRNIGKTLVTRTQGTKIASD	Negative
430.	D3Z6C3:56	RNIGKTLVTRTQGTKIASDGLKGRVFEVS	Negative
431.	D3Z6C3:63	VTRTQGTKIASDGLKGRVFEVSLADLQND	Negative
432.	D3Z6C3:83	VSLADLQNDEVAFRKFKLITEDVQGKNCL	Negative
433.	D3Z6C3:85	LADLQNDEVAFRKFKLITEDVQGKNCLTN	Negative
434.	D3Z6C3:94	AFRKFKLITEDVQGKNCLTNFHGMDLTRD	Negative
435.	D3Z6C3:109	NCLTNFHGMDLTRDKMCSMVKKWQTMIEA	Negative
436.	D3Z6C3:115	HGMDLTRDKMCSMVKKWQTMIEAHVDVKT	Negative
437.	D3Z6C3:116	GMDLTRDKMCSMVKKWQTMIEAHVDVKTT	Negative
438.	D3Z6C3:128	VKKWQTMIEAHVDVKTTDGYLLRLFCVGF	Negative
439.	D3Z6C3:145	DGYLLRLFCVGFTKKRNNQIRKTSYAQHQ	Negative
440.	D3Z6C3:152	FCVGFTKKRNNQIRKTSYAQHQQVRQIRK	Negative
441.	D3Z6C3:166	KTSYAQHQQVRQIRKKMMEIMTREVQTND	Negative
442.	D3Z6C3:167	TSYAQHQQVRQIRKKMMEIMTREVQTNDL	Negative
443.	D3Z6C3:182	MMEIMTREVQTNDLKEVVNKLIPDSIGKD	Negative
444.	D3Z6C3:187	TREVQTNDLKEVVNKLIPDSIGKDIEKAC	Negative
445.	D3Z6C3:195	LKEVVNKLIPDSIGKDIEKACQSIYPLHD	Negative
446.	D3Z6C3:199	VNKLIPDSIGKDIEKACQSIYPLHDVFVR	Negative
447.	D3Z6C3:214	ACQSIYPLHDVFVRKVKMLKKPKSELGKL	Negative
448.	D3Z6C3:216	QSIYPLHDVFVRKVKMLKKPKSELGKLME	Negative
449.	D3Z6C3:219	YPLHDVFVRKVKMLKKPKSELGKLMELHG	Negative
450.	D3Z6C3:220	PLHDVFVRKVKMLKKPKSELGKLMELHGE	Negative
451.	D3Z6C3:222	HDVFVRKVKMLKKPKSELGKLMELHGEGG	Negative
452.	D3Z6C3:227	RKVKMLKKPKSELGKLMELHGEGGSSGKA	Negative
453.	D3Z6C3:240	GKLMELHGEGGSSGKAAGDETGAKVERAD	Negative
454.	D3Z6C3:249	GGSSGKAAGDETGAKVERADGYEPPVQES	Negative
455.	E7EP94:3	XXXXXXXXXXXXMAKAAAIGIDLGTTYSC	Negative
456.	E7EP94:25	LGTTYSCVGVFQHGKVQISSMVLTKMKEI	Negative
457.	E7EP94:35	FQHGKVQISSMVLTKMKEIAEAYLGYPVT	Negative
458.	E7EP94:37	HGKVQISSMVLTKMKEIAEAYLGYPVTNA	Negative
459.	E7EP94:68	TVPAYFNDSQRQATKDAGVIAGLNVLRII	Negative
460.	E7EP94:99	PTAAAIAYGLDRTGKGERNVLIFDLGGGT	Negative
461.	E7EP94:129	DVSILTIDDGIFEVKATAGDTHLGGEDFD	Negative
462.	E7EP94:155	DFDNRLVNHFVEEFKRKHKKDISQNKRAV	Negative
463.	E7EP94:157	DNRLVNHFVEEFKRKHKKDISQNKRAVRR	Negative
464.	E7EP94:159	RLVNHFVEEFKRKHKKDISQNKRAVRRLR	Negative
465.	E7EP94:160	LVNHFVEEFKRKHKKDISQNKRAVRRLRT	Negative
466.	E7EP94:166	EEFKRKHKKDISQNKRAVRRLRTACERAK	Negative
467.	E7EP94:180	KRAVRRLRTACERAKRTLSSSTQASLEID	Negative
468.	E7EP94:228	LCSDLFRSTLEPVEKALRDAKLDKAQIHD	Negative
469.	E7EP94:234	RSTLEPVEKALRDAKLDKAQIHDLVLVGG	Negative
470.	E7EP94:237	LEPVEKALRDAKLDKAQIHDLVLVGGSTR	Negative
471.	E7EP94:254	IHDLVLVGGSTRIPKVQKLLQDFFNGRDL	Negative
472.	E7EP94:257	LVLVGGSTRIPKVQKLLQDFFNGRDLNKS	Negative
473.	E7EP94:270	QKLLQDFFNGRDLNKSINPDEAVAYGAAV	Negative
474.	E7EP94:293	AYGAAVQAAILMGDKSENVQDLLLLDVAP	Negative
475.	E7EP94:324	LGLETAGGVMTALIKRNSTIPTKQTQIFT	Negative
476.	E7EP94:332	VMTALIKRNSTIPTKQTQIFTTYSDNQPG	Negative
477.	E7EP94:360	GVLIQVYEGERAMTKDNNLLGRFELSGIP	Negative
478.	E7EP94:402	DIDANGILNVTATDKSTGKANKITITNDK	Negative
479.	E7EP94:406	NGILNVTATDKSTGKANKITITNDKGRLS	Negative
480.	E7EP94:409	LNVTATDKSTGKANKITITNDKGRLSKEE	Negative
481.	E7EP94:416	KSTGKANKITITNDKGRLSKEEIERMVQE	Negative
482.	E7EP94:421	ANKITITNDKGRLSKEEIERMVQEAEKYK	Negative

483.	E7EP94:433	LSKEEIERMVQEAEKYKAEDEVQRERVSA	Negative
484.	E7EP94:435	KEEIERMVQEAEKYKAEDEVQRERVSAKN	Negative
485.	E7EP94:448	YKAEDEVQRERVSAKNALESYAFNMKSAV	Negative
486.	E7EP94:459	VSAKNALESYAFNMKSAVEDEGLKGKISE	Negative
487.	E7EP94:468	YAFNMKSAVEDEGLKGKISEADKKKVLDK	Negative
488.	E7EP94:470	FNMKSAVEDEGLKGKISEADKKKVLDKCQ	Negative
489.	E7EP94:476	VEDEGLKGKISEADKKKVLDKCQEVISWL	Negative
490.	E7EP94:477	EDEGLKGKISEADKKKVLDKCQEVISWLD	Negative
491.	E7EP94:478	DEGLKGKISEADKKKVLDKCQEVISWLDA	Negative
492.	E7EP94:482	KGKISEADKKKVLDKCQEVISWLDANTLA	Negative
493.	E7EP94:498	QEVISWLDANTLAEKDEFEHKRKELEQVC	Negative
494.	E7EP94:504	LDANTLAEKDEFEHKRKELEQVCNPIISG	Negative
495.	E7EP94:537	AGGPGPGGFGAQGPKGGSGSGPTIEEVDX	Negative
496.	E9PZS8:4	XXXXXXXXXXXMYYKFSSFTQKLAGAWAS	Negative
497.	E9PZS8:11	XXXXMYYKFSSFTQKLAGAWASEAYTPQG	Negative
498.	E9PZS8:27	AGAWASEAYTPQGLKPVSTEAPPIIFATP	Negative
499.	E9PZS8:43	VSTEAPPIIFATPTKLTSSVTAYDYSGKN	Negative
500.	E9PZS8:58	LTSSVTAYDYSGKNKVPELQKFFQPPFLK	Negative
501.	E9PZS8:64	AYDYSGKNKVPELQKFFQPPFLKENSLLQ	Negative
502.	E9PZS8:72	KVPELQKFFQPPFLKENSLLQFGPLANNG	Negative
503.	E9PZS8:91	LQFGPLANNGTFVQKADGFHLKRGLPDQM	Negative
504.	E9PZS8:98	NNGTFVQKADGFHLKRGLPDQMLYRTTMA	Negative
505.	E9PZS8:134	YCLIALYMASQPRNKXXXXXXXXXXXXXX	Negative
506.	E9Q070:10	XXXXXMPREDRATWKSNYFLKIIQLLDDY	Negative
507.	E9Q070:16	PREDRATWKSNYFLKIIQLLDDYPKCFIV	Negative
508.	E9Q070:26	NYFLKIIQLLDDYPKCFIVGADNVGSKQM	Negative
509.	E9Q070:38	YPKCFIVGADNVGSKQMQQIRMSLRGKAV	Negative
510.	E9Q070:50	GSKQMQQIRMSLRGKAVVLMGKNTMMRKA	Negative
511.	E9Q070:57	IRMSLRGKAVVLMGKNTMMRKAIRGHLEN	Negative
512.	E9Q070:63	GKAVVLMGKNTMMRKAIRGHLENNPALEK	Negative
513.	E9Q070:77	KAIRGHLENNPALEKLLPHIRGNVGFVFT	Negative
514.	E9Q070:92	LLPHIRGNVGFVFTKEDLTEIRDMLLANK	Negative
515.	E9Q070:106	KEDLTEIRDMLLANKVPAAARAGAIAPCE	Negative
516.	E9Q070:134	EVTVPAQNTGLGPEKTSFFQALGITTKIS	Negative
517.	E9Q070:146	PEKTSFFQALGITTKISSGTIEILSDVQL	Negative
518.	E9Q070:162	SSGTIEILSDVQLIKTGDKVRASEATLLN	Negative
519.	E9Q070:166	IEILSDVQLIKTGDKVRASEATLLNMLNI	Negative
520.	E9Q070:246	PTVASVPHSIINGYKRVLALSVETEYTFP	Negative
521.	E9Q070:264	ALSVETEYTFPLAEKVKAFLADPSAFAAA	Negative
522.	E9Q070:266	SVETEYTFPLAEKVKAFLADPSAFAAAAP	Negative
523.	E9Q070:301	AAPAAAAAPAKAEAKEESEESDEDMGFGL	Negative
524.	E9Q1V0:4	XXXXXXXXXXXMGGKVPPATHKAKSEENT	Negative
525.	E9Q1V0:11	XXXXMGGKVPPATHKAKSEENTKEEKRDK	Negative
526.	E9Q1V0:13	XXMGGKVPPATHKAKSEENTKEEKRDKTT	Negative
527.	E9Q1V0:19	VPPATHKAKSEENTKEEKRDKTTEENIKT	Negative
528.	E9Q1V0:22	ATHKAKSEENTKEEKRDKTTEENIKTEEL	Negative
529.	E9Q1V0:25	KAKSEENTKEEKRDKTTEENIKTEELSSE	Negative
530.	E9Q1V0:32	TKEEKRDKTTEENIKTEELSSEESDLEID	Negative
531.	E9Q1V0:79	NAEITEEMMDEANEKKGAAIEALNDGELQ	Negative
532.	E9Q1V0:80	AEITEEMMDEANEKKGAAIEALNDGELQK	Negative
533.	E9Q1V0:94	KGAAIEALNDGELQKAIDLFTDAIKLNPR	Negative
534.	E9Q1V0:104	GELQKAIDLFTDAIKLNPRLAILYAKRAS	Negative
535.	E9Q1V0:122	RLAILYAKRASVFVKLQKPNAAXXXXXXX	Negative
536.	E9Q1V0:125	ILYAKRASVFVKLQKPNAAXXXXXXXXXX	Negative

537.	E9Q3T0:27	ALILHGDEVTVTEDKINALIKAAGVSVEP	Negative
538.	E9Q3T0:33	DEVTVTEDKINALIKAAGVSVEPFWPGLF	Negative
539.	E9Q3T0:49	AGVSVEPFWPGLFAKALANVNIGSLICNV	Negative
540.	E9Q3T0:92	GGAALSTAAAPAEEKKVEAKKEESEESED	Negative
541.	E9Q3T0:93	GAALSTAAAPAEEKKVEAKKEESEESEDD	Negative
542.	E9Q3T0:97	STAAAPAEEKKVEAKKEESEESEDDMGFG	Negative
543.	E9QMI7:73	LLEEALDQDRTALQKVKKSVKAIYNSGQD	Negative
544.	E9QMI7:75	EEALDQDRTALQKVKKSVKAIYNSGQDHV	Negative
545.	E9QMI7:76	EALDQDRTALQKVKKSVKAIYNSGQDHVQ	Negative
546.	E9QMI7:79	DQDRTALQKVKKSVKAIYNSGQDHVQNEE	Negative
547.	E9QMI7:101	DHVQNEENYAQVLDKFGSNFLSRDNPDLG	Negative
548.	E9QMI7:120	FLSRDNPDLGTAFVKFSTLTKELSTLLKN	Negative
549.	E9QMI7:126	PDLGTAFVKFSTLTKELSTLLKNLLQGLS	Negative
550.	E9QMI7:133	VKFSTLTKELSTLLKNLLQGLSHNVIFTL	Negative
551.	E9QMI7:152	GLSHNVIFTLDSLLKGDLKGVKGDLKKPF	Negative
552.	E9QMI7:156	NVIFTLDSLLKGDLKGVKGDLKKPFDKAW	Negative
553.	E9QMI7:159	FTLDSLLKGDLKGVKGDLKKPFDKAWKDY	Negative
554.	E9QMI7:163	SLLKGDLKGVKGDLKKPFDKAWKDYETKF	Negative
555.	E9QMI7:164	LLKGDLKGVKGDLKKPFDKAWKDYETKFT	Negative
556.	E9QMI7:168	DLKGVKGDLKKPFDKAWKDYETKFTKIEK	Negative
557.	E9QMI7:171	GVKGDLKKPFDKAWKDYETKFTKIEKEKR	Negative
558.	E9QMI7:176	LKKPFDKAWKDYETKFTKIEKEKREHAKQ	Negative
559.	E9QMI7:179	PFDKAWKDYETKFTKIEKEKREHAKQHGM	Negative
560.	E9QMI7:182	KAWKDYETKFTKIEKEKREHAKQHGMIRT	Negative
561.	E9QMI7:184	WKDYETKFTKIEKEKREHAKQHGMIRTEI	Negative
562.	E9QMI7:189	TKFTKIEKEKREHAKQHGMIRTEITGAEI	Negative
563.	E9QMI7:209	RTEITGAEIAEEMEKERRLFQLQMCEYLI	Negative
564.	E9QMI7:224	ERRLFQLQMCEYLIKVNEIKTKKGVDLLQ	Negative
565.	E9QMI7:229	QLQMCEYLIKVNEIKTKKGVDLLQNLIKY	Negative
566.	E9QMI7:231	QMCEYLIKVNEIKTKKGVDLLQNLIKYYH	Negative
567.	E9QMI7:232	MCEYLIKVNEIKTKKGVDLLQNLIKYYHA	Negative
568.	E9QMI7:242	IKTKKGVDLLQNLIKYYHAQCNFFQDGLK	Negative
569.	E9QMI7:256	KYYHAQCNFFQDGLKTADKLKQYIEKLAA	Negative
570.	E9QMI7:260	AQCNFFQDGLKTADKLKQYIEKLAADLYN	Negative
571.	E9QMI7:262	CNFFQDGLKTADKLKQYIEKLAADLYNIK	Negative
572.	E9QMI7:267	DGLKTADKLKQYIEKLAADLYNIKQTQDE	Negative
573.	E9QMI7:276	KQYIEKLAADLYNIKQTQDEEKKQLTALR	Negative
574.	E9QMI7:283	AADLYNIKQTQDEEKKQLTALRDLIKSSL	Negative
575.	E9QMI7:284	ADLYNIKQTQDEEKKQLTALRDLIKSSLQ	Negative
576.	E9QMI7:294	DEEKKQLTALRDLIKSSLQLDPKEVGGLY	Negative
577.	E9QMI7:302	ALRDLIKSSLQLDPKEVGGLYVASRANSV	Negative
578.	E9QMI7:336	SRQGGYSMHQLQGNKEYGSEKKGFLLKKS	Negative
579.	E9QMI7:342	SMHQLQGNKEYGSEKKGFLLKKSDGIRKV	Negative
580.	E9QMI7:343	MHQLQGNKEYGSEKKGFLLKKSDGIRKVW	Negative
581.	E9QMI7:348	GNKEYGSEKKGFLLKKSDGIRKVWQRRKC	Negative
582.	E9QMI7:349	NKEYGSEKKGFLLKKSDGIRKVWQRRKCA	Negative
583.	E9QMI7:355	EKKGFLLKKSDGIRKVWQRRKCAVKNGIL	Negative
584.	E9QMI7:361	LKKSDGIRKVWQRRKCAVKNGILTISHAT	Negative
585.	E9QMI7:365	DGIRKVWQRRKCAVKNGILTISHATSNRQ	Negative
586.	E9QMI7:382	ILTISHATSNRQPAKLNLLTCQVKPNAED	Negative
587.	E9QMI7:391	NRQPAKLNLLTCQVKPNAEDKKSFDLISH	Negative
588.	E9QMI7:397	LNLLTCQVKPNAEDKKSFDLISHNRTYHF	Negative
589.	E9QMI7:398	NLLTCQVKPNAEDKKSFDLISHNRTYHFQ	Negative

590.	E9QMI7:430	EQDYIAWISVLTNSKEEALTMAFRGEQST	Negative
591.	E9QMI7:454	GEQSTGENSLEDLTKAIIEDVQRLPGNDI	Negative
592.	E9QMI7:513	MGVHISRIQSLELDKLGTSELLLAKNVGN	Negative
593.	E9QMI7:523	LELDKLGTSELLLAKNVGNNSFNDIMEAN	Negative
594.	E9QMI7:544	FNDIMEANLPSPSPKPTPSSDMTVRKEYI	Negative
595.	E9QMI7:555	PSPKPTPSSDMTVRKEYITAKYVDHRFSR	Negative
596.	E9QMI7:561	PSSDMTVRKEYITAKYVDHRFSRKTCASS	Negative
597.	E9QMI7:570	EYITAKYVDHRFSRKTCASSSAKLNELLE	Negative
598.	E9QMI7:578	DHRFSRKTCASSSAKLNELLEAIKSRDLL	Negative
599.	E9QMI7:587	ASSSAKLNELLEAIKSRDLLALIQVYAEG	Negative
600.	E9QMI7:647	HLVDFLVQNCGNLDKQTSVGNTVLHYCSM	Negative
601.	E9QMI7:664	SVGNTVLHYCSMYGKPECLKLLLRSKPTV	Negative
602.	E9QMI7:669	VLHYCSMYGKPECLKLLLRSKPTVDIVNQ	Negative
603.	E9QMI7:693	DIVNQNGETALDIAKRLKATQCEDLLSQA	Negative
604.	E9QMI7:696	NQNGETALDIAKRLKATQCEDLLSQAKSG	Negative
605.	E9QMI7:708	RLKATQCEDLLSQAKSGKFNPHVHVEYEW	Negative
606.	E9QMI7:711	ATQCEDLLSQAKSGKFNPHVHVEYEWNLR	Negative
607.	E9QMI7:739	RQDEMDESDDDLDDKPSPIKKERSPRPQS	Negative
608.	E9QMI7:744	DESDDDLDDKPSPIKKERSPRPQSFCHSS	Negative
609.	E9QMI7:745	ESDDDLDDKPSPIKKERSPRPQSFCHSSS	Negative
610.	E9QMI7:765	PQSFCHSSSISPQDKLALPGFSTPRDKQR	Negative
611.	E9QMI7:777	QDKLALPGFSTPRDKQRLSYGAFTNQIFA	Negative
612.	E9QMI7:815	PTSEAPPLPPRNAGKGNDVGPLSSSKTAN	Negative
613.	E9QMI7:826	NAGKGNDVGPLSSSKTANKFEGLSQQAST	Negative
614.	E9QMI7:830	GNDVGPLSSSKTANKFEGLSQQASTSSAK	Negative
615.	E9QMI7:844	KFEGLSQQASTSSAKTALGPRVLPKLPQK	Negative
616.	E9QMI7:854	TSSAKTALGPRVLPKLPQKVALRKTETSH	Negative
617.	E9QMI7:858	KTALGPRVLPKLPQKVALRKTETSHHLSL	Negative
618.	E9QMI7:863	PRVLPKLPQKVALRKTETSHHLSLDRTNI	Negative
619.	E9QMI7:884	LSLDRTNIPPETFQKSSQLTELPQKPPLG	Negative
620.	E9QMI7:894	ETFQKSSQLTELPQKPPLGELPPKPVELA	Negative
621.	E9QMI7:903	TELPQKPPLGELPPKPVELAPKPQVGELP	Negative
622.	E9QMI7:910	PLGELPPKPVELAPKPQVGELPPKPGELP	Negative
623.	E9QMI7:919	VELAPKPQVGELPPKPGELPPKPQLGDLP	Negative
624.	E9QMI7:926	QVGELPPKPGELPPKPQLGDLPPKPQLSD	Negative
625.	E9QMI7:935	GELPPKPQLGDLPPKPQLSDLPPKPQMKD	Negative
626.	E9QMI7:944	GDLPPKPQLSDLPPKPQMKDLPPKPQLGD	Negative
627.	E9QMI7:948	PKPQLSDLPPKPQMKDLPPKPQLGDLLAK	Negative
628.	E9QMI7:953	SDLPPKPQMKDLPPKPQLGDLLAKSQAGD	Negative
629.	E9QMI7:962	KDLPPKPQLGDLLAKSQAGDVSAKVQPPS	Negative
630.	E9QMI7:971	GDLLAKSQAGDVSAKVQPPSEVTQRSHTG	Negative
631.	E9QMI7:999	GDLSPNVQSRDAIQKQASEDSNDLTPTLP	Negative
632.	E9QMI7:1022	LTPTLPETPVPLPRKINTGKNKVRRVKTI	Negative
633.	E9QMI7:1027	PETPVPLPRKINTGKNKVRRVKTIYDCQA	Negative
634.	E9QMI7:1029	TPVPLPRKINTGKNKVRRVKTIYDCQADN	Negative
635.	E9QMI7:1034	PRKINTGKNKVRRVKTIYDCQADNDDELT	Negative
636.	E9QMI7:1077	QEWWIGHIEGQPERKGVFPVSFVHILSDX	Negative
637.	E9QNN1:5	XXXXXXXXXXMGDIKNFLYAWCGKRKMTP	Negative
638.	E9QNN1:14	XMGDIKNFLYAWCGKRKMTPAYEIRAVGN	Negative
639.	E9QNN1:16	GDIKNFLYAWCGKRKMTPAYEIRAVGNKN	Negative
640.	E9QNN1:29	RKMTPAYEIRAVGNKNRQKFMCEVRVEGF	Negative
641.	E9QNN1:33	PAYEIRAVGNKNRQKFMCEVRVEGFNYAG	Negative
642.	E9QNN1:54	VEGFNYAGMGNSTNKKDAQSNAARDFVNY	Negative
643.	E9QNN1:55	EGFNYAGMGNSTNKKDAQSNAARDFVNYL	Negative

644.	E9QNN1:76	ARDFVNYLVRINEVKSEEVPAVGIVPPPP	Negative
645.	E9QNN1:121	PAPMGGPLPPHLALKAEAENNSGVESSGY	Negative
646.	E9QNN1:149	YGSPGPTWDRGANLKDYYSRKEEQEVQAT	Negative
647.	E9QNN1:155	TWDRGANLKDYYSRKEEQEVQATLESEEV	Negative
648.	E9QNN1:185	LNAGLHGNWTLENAKARLNQYFQKEKIQG	Negative
649.	E9QNN1:194	TLENAKARLNQYFQKEKIQGEYKYTQVGP	Negative
650.	E9QNN1:196	ENAKARLNQYFQKEKIQGEYKYTQVGPDH	Negative
651.	E9QNN1:202	LNQYFQKEKIQGEYKYTQVGPDHNRSFIA	Negative
652.	E9QNN1:223	DHNRSFIAEMTIYIKQLGRRIFAREHGSN	Negative
653.	E9QNN1:238	QLGRRIFAREHGSNKKLAAQSCALSLVRQ	Negative
654.	E9QNN1:239	LGRRIFAREHGSNKKLAAQSCALSLVRQL	Negative
655.	E9QNN1:267	LYHLGVIEAYSGLTKKKEGERVEPYKVFL	Negative
656.	E9QNN1:268	YHLGVIEAYSGLTKKKEGERVEPYKVFLS	Negative
657.	E9QNN1:269	HLGVIEAYSGLTKKKEGERVEPYKVFLSP	Negative
658.	E9QNN1:278	GLTKKKEGERVEPYKVFLSPDLELQLQNV	Negative
659.	E9QNN1:317	PPVDPSMPVILNIGKLAHFEPSQRQNAVG	Negative
660.	E9QNN1:368	PLAYASTEQISMDLKNELTYQMEQDHNLQ	Negative
661.	E9QNN1:394	NLQSVLQERELLPVKKFEAEILEAISSNS	Negative
662.	E9QNN1:395	LQSVLQERELLPVKKFEAEILEAISSNSV	Negative
663.	E9QNN1:420	SNSVVIIRGATGCGKTTQVPQYILDDFIQ	Negative
664.	E9QNN1:470	VAERVAYERGEEPGKSCGYSVRFESILPR	Negative
665.	E9QNN1:500	HASIMFCTVGVLLRKLEAGIRGISHVIVD	Negative
666.	E9QNN1:592	LEDCIQMTQFIPPPKDKKKKDKEDDGGED	Negative
667.	E9QNN1:594	DCIQMTQFIPPPKDKKKKDKEDDGGEDDD	Negative
668.	E9QNN1:595	CIQMTQFIPPPKDKKKKDKEDDGGEDDDA	Negative
669.	E9QNN1:596	IQMTQFIPPPKDKKKKDKEDDGGEDDDAN	Negative
670.	E9QNN1:597	QMTQFIPPPKDKKKKDKEDDGGEDDDANC	Negative
671.	E9QNN1:599	TQFIPPPKDKKKKDKEDDGGEDDDANCNL	Negative
672.	E9QNN1:624	NCNLICGDEYGPETKLSMSQLNEKETPFE	Negative
673.	E9QNN1:633	YGPETKLSMSQLNEKETPFELIEALLKYI	Negative
674.	E9QNN1:645	NEKETPFELIEALLKYIETLNVPGAVLVF	Negative
675.	E9QNN1:671	LVFLPGWNLIYTMQKHLENNSHFGSHRYQ	Negative
676.	E9QNN1:700	ILPLHSQIPREEQRKVFDPVPDGVTKVIL	Negative
677.	E9QNN1:711	EQRKVFDPVPDGVTKVILSTNIAETSITI	Negative
678.	E9QNN1:736	SITINDVVYVIDSCKQKVKLFTAHNNMTN	Negative
679.	E9QNN1:738	TINDVVYVIDSCKQKVKLFTAHNNMTNYA	Negative
680.	E9QNN1:740	NDVVYVIDSCKQKVKLFTAHNNMTNYATV	Negative
681.	E9QNN1:758	AHNNMTNYATVWASKTNLEQRKGRAGRVR	Negative
682.	E9QNN1:765	YATVWASKTNLEQRKGRAGRVRPGFCFHL	Negative
683.	E9QNN1:809	EMFRTPLHEIALSIKLLRLGGIGQFLAKA	Negative
684.	E9QNN1:822	IKLLRLGGIGQFLAKAIEPPPLDAVIEAE	Negative
685.	E9QNN1:860	DANDELTPLGRILAKLPIEPRFGKMMIMG	Negative
686.	E9QNN1:869	GRILAKLPIEPRFGKMMIMGCIFYVGDAV	Negative
687.	E9QNN1:901	SAATCFPEPFISEGKRLGYIHRNFAGNRF	Negative
688.	E9QNN1:946	RMSGEEAEIRFCEQKRLNMATLRMTWEAK	Negative
689.	E9QNN1:960	KRLNMATLRMTWEAKVQLKEILINSGFPE	Negative
690.	E9QNN1:964	MATLRMTWEAKVQLKEILINSGFPEDCLL	Negative
691.	E9QNN1:1010	SLLAFGVYPNVCYHKEKRKILTTEGRNAL	Negative
692.	E9QNN1:1012	LAFGVYPNVCYHKEKRKILTTEGRNALIH	Negative
693.	E9QNN1:1014	FGVYPNVCYHKEKRKILTTEGRNALIHKS	Negative
694.	E9QNN1:1040	HKSSVNCPFSSQDMKYPSPFFVFGEKIRT	Negative
695.	E9QNN1:1051	QDMKYPSPFFVFGEKIRTRAISAKGMTLV	Negative
696.	E9QNN1:1060	FVFGEKIRTRAISAKGMTLVTPLQLLLFA	Negative
697.	E9QNN1:1076	MTLVTPLQLLLFASKKVQSDGQIVFIDDW	Negative

698.	E9QNN1:1077	TLVTPLQLLLFASKKVQSDGQIVFIDDWI	Negative
699.	E9QNN1:1119	ALRAAMEALVVEVSKQPNIISQLDPVNEH	Negative
700.	E9QNN1:1166	MIGSVRYGDGPRPPKMARYDNGSGYRRGY	Negative
701.	F6UB20:56	PRRRRQLQQVGTVSKVWIYPIKSCKGVSV	Negative
702.	F6UB20:66	GTVSKVWIYPIKSCKGVSVCETECTDMGL	Negative
703.	F6UB20:84	VCETECTDMGLRCGKVRDRFWMVVKEDGH	Negative
704.	F6UB20:94	LRCGKVRDRFWMVVKEDGHMVTARQEPRL	Negative
705.	F6UB20:135	TLEAPGMEQIVLPIKLPSSNKIHNCRLFG	Negative
706.	F6UB20:141	MEQIVLPIKLPSSNKIHNCRLFGLDIKGR	Negative
707.	F6UB20:153	SNKIHNCRLFGLDIKGRDCGDEVAQWFTN	Negative
708.	F6UB20:170	DCGDEVAQWFTNYLKTQAYRLVQFDTSMK	Negative
709.	F6UB20:184	KTQAYRLVQFDTSMKGRTTKKLYPSESYL	Negative
710.	F6UB20:189	RLVQFDTSMKGRTTKKLYPSESYLQNYEV	Negative
711.	F6UB20:190	LVQFDTSMKGRTTKKLYPSESYLQNYEVA	Negative
712.	F6UB20:227	LISEASLVDLNTRLKKKVKMEYFRPNIVV	Negative
713.	F6UB20:228	ISEASLVDLNTRLKKKVKMEYFRPNIVVS	Negative
714.	F6UB20:229	SEASLVDLNTRLKKKVKMEYFRPNIVVSG	Negative
715.	F6UB20:231	ASLVDLNTRLKKKVKMEYFRPNIVVSGCE	Negative
716.	F6YLP3:2	XXXXXXXXXXXXXIKLAGVHSVASCFVVP	Negative
717.	F6YLP3:33	TGMSLTLCFLTLRHKRPKAKYIIWPRIDQ	Negative
718.	F6YLP3:36	SLTLCFLTLRHKRPKAKYIIWPRIDQKSC	Negative
719.	F6YLP3:38	TLCFLTLRHKRPKAKYIIWPRIDQKSCFK	Negative
720.	F6YLP3:52	KYIIWPRIDQKSCFKSMVTAGFEPVVIEN	Negative
721.	F6YLP3:78	IENVLEGDELRTDLKAVEAKIQELGPEHI	Negative
722.	F6YLP3:83	EGDELRTDLKAVEAKIQELGPEHILCLHS	Negative
723.	F6YLP3:125	LGLVELMLSFRAWTKILWFQXXXXXXXXX	Negative
724.	F8WIT2:3	XXXXXXXXXXXXMAKIAQGAMYRGSVHDF	Negative
725.	F8WIT2:34	FDANQDAEALYTAMKGFGSDKESILELIT	Negative
726.	F8WIT2:40	AEALYTAMKGFGSDKESILELITSRSNKQ	Negative
727.	F8WIT2:53	DKESILELITSRSNKQRQEICQNYKSLYG	Negative
728.	F8WIT2:63	SRSNKQRQEICQNYKSLYGKDLIEDLKYE	Negative
729.	F8WIT2:68	QRQEICQNYKSLYGKDLIEDLKYELTGKF	Negative
730.	F8WIT2:75	NYKSLYGKDLIEDLKYELTGKFERLIVNL	Negative
731.	F8WIT2:81	GKDLIEDLKYELTGKFERLIVNLMRPLAY	Negative
732.	F8WIT2:99	LIVNLMRPLAYCDAKEIKDAISGVGTDEK	Negative
733.	F8WIT2:102	NLMRPLAYCDAKEIKDAISGVGTDEKCLI	Negative
734.	F8WIT2:113	KEIKDAISGVGTDEKCLIEILASRTNEQM	Negative
735.	F8WIT2:135	SRTNEQMHQLVAAYKDAYERDLESDIIGD	Negative
736.	F8WIT2:156	LESDIIGDTSGHFQKMLVVLLQGTRENDD	Negative
737.	F8WIT2:191	VQQDVQDLYEAGELKWGTDEAQFIYILGN	Negative
738.	F8WIT2:208	TDEAQFIYILGNRSKQHLRLVFDEYLKTT	Negative
739.	F8WIT2:220	RSKQHLRLVFDEYLKTTGKPIEASIRGEL	Negative
740.	F8WIT2:224	HLRLVFDEYLKTTGKPIEASIRGELSGDF	Negative
741.	F8WIT2:240	IEASIRGELSGDFEKLMLAVVKCIRSTPE	Negative
742.	F8WIT2:247	ELSGDFEKLMLAVVKCIRSTPEYFAERLF	Negative
743.	F8WIT2:262	CIRSTPEYFAERLFKAMKGLGTRDNTLIR	Negative
744.	F8WIT2:265	STPEYFAERLFKAMKGLGTRDNTLIRIMV	Negative
745.	F8WIT2:296	SELDMLDIREIFRTKYEKSLYSMIKNDTS	Negative
746.	F8WIT2:299	DMLDIREIFRTKYEKSLYSMIKNDTSGEY	Negative
747.	F8WIT2:306	IFRTKYEKSLYSMIKNDTSGEYKKALLKL	Negative
748.	F8WIT2:314	SLYSMIKNDTSGEYKKALLKLCGGDDDAA	Negative
749.	F8WIT2:315	LYSMIKNDTSGEYKKALLKLCGGDDDAAG	Negative
750.	F8WIT2:319	IKNDTSGEYKKALLKLCGGDDDAAGQFFP	Negative
751.	F8WIT2:354	YQMWELSAVSRVELKGTVCAANDFNPDAD	Negative

752.	F8WIT2:370	TVCAANDFNPDADAKALRKAMKGIGTDEA	Negative
753.	F8WIT2:374	ANDFNPDADAKALRKAMKGIGTDEATIID	Negative
754.	F8WIT2:377	FNPDADAKALRKAMKGIGTDEATIIDIVT	Negative
755.	F8WIT2:406	HRSNAQRQQIRQTFKSHFGRDLMADLKSE	Negative
756.	F8WIT2:418	TFKSHFGRDLMADLKSEISGDLARLILGL	Negative
757.	F8WIT2:456	KQLKKAMEGAGTDEKTLIEILATRTNAEI	Negative
758.	F8WIT2:478	TRTNAEIRAINEAYKEDYHKSLEDALSSD	Negative
759.	F8WIT2:483	EIRAINEAYKEDYHKSLEDALSSDTSGHF	Negative
760.	F8WIT2:534	QEDAQEIADTPSGDKTSLETRFMTVLCTR	Negative
761.	F8WIT2:562	RSYPHLRRVFQEFIKKTNYDIEHVIKKEM	Negative
762.	F8WIT2:563	SYPHLRRVFQEFIKKTNYDIEHVIKKEMS	Negative
763.	F8WIT2:573	EFIKKTNYDIEHVIKKEMSGDVKDAFVAI	Negative
764.	F8WIT2:574	FIKKTNYDIEHVIKKEMSGDVKDAFVAIV	Negative
765.	F8WIT2:581	DIEHVIKKEMSGDVKDAFVAIVQSVKNKP	Negative
766.	F8WIT2:592	GDVKDAFVAIVQSVKNKPLFFADKLYKSM	Negative
767.	F8WIT2:594	VKDAFVAIVQSVKNKPLFFADKLYKSMKG	Negative
768.	F8WIT2:601	IVQSVKNKPLFFADKLYKSMKGAGTDEKT	Negative
769.	F8WIT2:604	SVKNKPLFFADKLYKSMKGAGTDEKTLTR	Negative
770.	F8WIT2:607	NKPLFFADKLYKSMKGAGTDEKTLTRVMV	Negative
771.	F8WIT2:614	DKLYKSMKGAGTDEKTLTRVMVSRSEIDL	Negative
772.	F8WIT2:638	SEIDLLNIRREFIEKYDKSLHQAIEGDTS	Negative
773.	F8WIT2:641	DLLNIRREFIEKYDKSLHQAIEGDTSGDF	Negative
774.	F8WIT2:657	LHQAIEGDTSGDFMKALLALCGGEDXXXX	Negative
775.	G3UYJ7:21	DLMSQPCRAVYIFAKKNGIPFQLRTIELL	Negative
776.	G3UYJ7:22	LMSQPCRAVYIFAKKNGIPFQLRTIELLK	Negative
777.	G3UYJ7:36	KNGIPFQLRTIELLKGQQYTDSFAQVNPL	Negative
778.	G3UYJ7:52	QQYTDSFAQVNPLRKVPALKDGDFVLAES	Negative
779.	G3UYJ7:57	SFAQVNPLRKVPALKDGDFVLAESVAILL	Negative
780.	G3UYJ7:76	VLAESVAILLYLSRKYKAPDHWYPQDLQT	Negative
781.	G3UYJ7:78	AESVAILLYLSRKYKAPDHWYPQDLQTRA	Negative
782.	G3UYJ7:116	HTALRSCCTRAMWQKMMFPVFLGQPVPPE	Negative
783.	G3UYJ7:149	TLAELDGCLQVLEDKFLRNQAFLTGSHIS	Negative
784.	G3UYJ7:191	VSVTIRPGMTLLMNKSTEPCAHLLVSSIG	Negative
785.	G3UYJ7:222	GTAEQNRTHSASFFKFLTEELSLDQDRIV	Negative
786.	G3UYJ7:250	VIRFFPLEAWQIGKKGTXXXXXXXXXXXX	Negative
787.	H3BTN5:3	XXXXXXXXXXXXMSKPHSEAGTAFIQTQQ	Negative
788.	H3BTN5:62	ICTIGPASRSVETLKEMIKSGMNVARLNF	Negative
789.	H3BTN5:66	GPASRSVETLKEMIKSGMNVARLNFSHGT	Negative
790.	H3BTN5:89	NFSHGTHEYHAETIKNVRTATESFASDPI	Negative
791.	H3BTN5:115	DPILYRPVAVALDTKGPEIRTGLIKGSGT	Negative
792.	H3BTN5:125	ALDTKGPEIRTGLIKGSGTAEVELKKGAT	Negative
793.	H3BTN5:135	TGLIKGSGTAEVELKKGATLKITLDNAYM	Negative
794.	H3BTN5:136	GLIKGSGTAEVELKKGATLKITLDNAYME	Negative
795.	H3BTN5:141	SGTAEVELKKGATLKITLDNAYMEKCDEN	Negative
796.	H3BTN5:151	GATLKITLDNAYMEKCDENILWLDYKNIC	Negative
797.	H3BTN5:162	YMEKCDENILWLDYKNICKVVEVGSKIYV	Negative
798.	H3BTN5:166	CDENILWLDYKNICKVVEVGSKIYVDDGL	Negative
799.	H3BTN5:173	LDYKNICKVVEVGSKIYVDDGLISLQVKQ	Negative
800.	H3BTN5:186	SKIYVDDGLISLQVKQKGADFLVTEVENG	Negative
801.	H3BTN5:188	IYVDDGLISLQVKQKGADFLVTEVENGGS	Negative
802.	H3BTN5:206	FLVTEVENGGSLGSKKGVNLPGAAVDLPA	Negative
803.	H3BTN5:207	LVTEVENGGSLGSKKGVNLPGAAVDLPAV	Negative
804.	H3BTN5:224	NLPGAAVDLPAVSEKDIQDLKFGVEQDVD	Negative
805.	H3BTN5:230	VDLPAVSEKDIQDLKFGVEQDVDMVFASF	Negative

806.	H3BTN5:247	VEQDVDMVFASFIRKASDVHEVRKVLGEK	Negative
807.	H3BTN5:256	ASFIRKASDVHEVRKVLGEKGKNIKIISK	Negative
808.	H3BTN5:261	KASDVHEVRKVLGEKGKNIKIISKIENHE	Negative
809.	H3BTN5:263	SDVHEVRKVLGEKGKNIKIISKIENHEGV	Negative
810.	H3BTN5:266	HEVRKVLGEKGKNIKIISKIENHEGVRRF	Negative
811.	H3BTN5:270	KVLGEKGKNIKIISKIENHEGVRRFDEIL	Negative
812.	H3BTN5:305	MVARGDLGIEIPAEKVFLAQKMMIGRCNR	Negative
813.	H3BTN5:311	LGIEIPAEKVFLAQKMMIGRCNRAGKPVI	Negative
814.	H3BTN5:322	LAQKMMIGRCNRAGKPVICATQMLESMIK	Negative
815.	H3BTN5:336	KPVICATQMLESMIKKPRPTRAEGSDVAN	Negative
816.	H3BTN5:337	PVICATQMLESMIKKPRPTRAEGSDVANA	Negative
817.	H3BTN5:393	HLIAREAEAAMFHRKLFEELVRASSHSTD	Negative
818.	H3BTN5:422	LMEAMAMGSVEASYKCLAAALIVLTESGR	Negative
819.	H3BTN5:475	RQAHLYRGIFPVLCKDPVQEAWAEDXXXX	Negative
820.	I7HPV9:7	XXXXXXXXMLLGGRKTTDIPLEGYLLSPI	Negative
821.	I7HPV9:26	PLEGYLLSPIQRICKYPLLLKELAKRTPG	Negative
822.	I7HPV9:32	LSPIQRICKYPLLLKELAKRTPGKHPDHT	Negative
823.	I7HPV9:36	QRICKYPLLLKELAKRTPGKHPDHTAVQS	Negative
824.	I7HPV9:41	YPLLLKELAKRTPGKHPDHTAVQSALQAM	Negative
825.	I7HPV9:56	HPDHTAVQSALQAMKTVCSNINETKRQME	Negative
826.	I7HPV9:66	LQAMKTVCSNINETKRQMEKLEALEQLQS	Negative
827.	I7HPV9:71	TVCSNINETKRQMEKLEALEQLQSHIEGW	Negative
828.	I7HPV9:105	TDICTELLLQGNLLKISAGNIQERAFFLF	Negative
829.	I7HPV9:127	ERAFFLFDNLLVYCKRKSRVTGSKKSTKR	Negative
830.	I7HPV9:129	AFFLFDNLLVYCKRKSRVTGSKKSTKRTK	Negative
831.	I7HPV9:136	LLVYCKRKSRVTGSKKSTKRTKSINGSLY	Negative
832.	I7HPV9:137	LVYCKRKSRVTGSKKSTKRTKSINGSLYI	Negative
833.	I7HPV9:140	CKRKSRVTGSKKSTKRTKSINGSLYIFRG	Negative
834.	I7HPV9:143	KSRVTGSKKSTKRTKSINGSLYIFRGRIN	Negative
835.	I7HPV9:185	ADYHSNGYTVTNGWKIHNTAKNKWFVCMA	Negative
836.	I7HPV9:191	GYTVTNGWKIHNTAKNKWFVCMAKTAEEK	Negative
837.	I7HPV9:193	TVTNGWKIHNTAKNKWFVCMAKTAEEKQK	Negative
838.	I7HPV9:200	IHNTAKNKWFVCMAKTAEEKQKWLDALIR	Negative
839.	I7HPV9:205	KNKWFVCMAKTAEEKQKWLDALIREREQR	Negative
840.	I7HPV9:207	KWFVCMAKTAEEKQKWLDALIREREQRES	Negative
841.	I7HPV9:223	LDALIREREQRESLKLGMERDAYVMIAEK	Negative
842.	I7HPV9:237	KLGMERDAYVMIAEKGEKLYHMMMSKKVN	Negative
843.	I7HPV9:240	MERDAYVMIAEKGEKLYHMMMSKKVNLIK	Negative
844.	I7HPV9:248	IAEKGEKLYHMMMSKKVNLIKDRRRKLST	Negative
845.	I7HPV9:249	AEKGEKLYHMMMSKKVNLIKDRRRKLSTV	Negative
846.	I7HPV9:254	KLYHMMMSKKVNLIKDRRRKLSTVPKCFL	Negative
847.	I7HPV9:259	MMSKKVNLIKDRRRKLSTVPKCFLGNEFV	Negative
848.	I7HPV9:265	NLIKDRRRKLSTVPKCFLGNEFVAWLLEI	Negative
849.	I7HPV9:284	NEFVAWLLEIGEISKTEEGVNLGQALLEN	Negative
850.	I7HPV9:307	QALLENGIIHHVSDKHQFKNEQVMYRFRY	Negative
851.	I7HPV9:311	ENGIIHHVSDKHQFKNEQVMYRFRYDDGT	Negative
852.	I7HPV9:327	EQVMYRFRYDDGTYKARSELEDIMSKGVR	Negative
853.	I7HPV9:338	GTYKARSELEDIMSKGVRLYCRLHSLYAP	Negative
854.	I7HPV9:355	RLYCRLHSLYAPVIKDRDYHLKTYKSVVP	Negative
855.	I7HPV9:362	SLYAPVIKDRDYHLKTYKSVVPGSKLVDW	Negative
856.	I7HPV9:365	APVIKDRDYHLKTYKSVVPGSKLVDWLLA	Negative
857.	I7HPV9:372	DYHLKTYKSVVPGSKLVDWLLAQGDCQTR	Negative
858.	I7HPV9:408	VGLCNNGFMHHVLEKSEFKDESQYFRFHA	Negative
859.	I7HPV9:412	NNGFMHHVLEKSEFKDESQYFRFHADEEM	Negative

860.	I7HPV9:432	FRFHADEEMEGTSSKNKQLRNDFKLVENI	Negative
861.	I7HPV9:434	FHADEEMEGTSSKNKQLRNDFKLVENILA	Negative
862.	I7HPV9:441	EGTSSKNKQLRNDFKLVENILAKRLLIPP	Negative
863.	I7HPV9:449	QLRNDFKLVENILAKRLLIPPQEDDYGFD	Negative
864.	I7HPV9:467	IPPQEDDYGFDLEEKNKAVVVKSVQRGSL	Negative
865.	I7HPV9:469	PQEDDYGFDLEEKNKAVVVKSVQRGSLAE	Negative
866.	I7HPV9:474	YGFDLEEKNKAVVVKSVQRGSLAEMAGLQ	Negative
867.	I7HPV9:492	RGSLAEMAGLQAGRKIYSINEDLVFLRPF	Negative
868.	I7HPV9:530	FFCSRRPLRLLVATKAKETIKVPDHPEAL	Negative
869.	I7HPV9:532	CSRRPLRLLVATKAKETIKVPDHPEALSF	Negative
870.	I7HPV9:536	PLRLLVATKAKETIKVPDHPEALSFQIRG	Negative
871.	I7HPV9:578	EAVAAGLCAGQCILKVNGTSVANDGALEV	Negative
872.	I7HPV9:686	EHGVVYEYMSTAGAKCHVLEKIVEPRGCF	Negative
873.	I7HPV9:692	EYMSTAGAKCHVLEKIVEPRGCFRLAAKI	Negative
874.	I7HPV9:705	EKIVEPRGCFRLAAKILEAFAVDDSIFVQ	Negative
875.	I7HPV9:746	VTMSHYEFHNICDTKLESIGQRIACYQEF	Negative
876.	I7HPV9:765	GQRIACYQEFAAQLKSRVSPPFKQASLEP	Negative
877.	I7HPV9:773	EFAAQLKSRVSPPFKQASLEPHPLCGLDF	Negative
878.	I7HPV9:803	PTNCHVNLMEVSYPKTTPSVGRSFSIRFG	Negative
879.	I7HPV9:819	TPSVGRSFSIRFGRKPSLIGLDPEQGLNP	Negative
880.	I7HPV9:850	YTQHCITTMAAPSWKCSPAVDEDSQGQGL	Negative
881.	I7HPV9:888	GAPSQQDRGLSFLLKQEDREIQDAYLQLF	Negative
882.	I7HPV9:904	EDREIQDAYLQLFTKLDVALKEMKQYVTQ	Negative
883.	I7HPV9:910	DAYLQLFTKLDVALKEMKQYVTQINRLLS	Negative
884.	I7HPV9:913	LQLFTKLDVALKEMKQYVTQINRLLSTIT	Negative
885.	I7HPV9:966	SEESEVDRTDHSGIKKVCFKVSEDEQEDS	Negative
886.	I7HPV9:967	EESEVDRTDHSGIKKVCFKVSEDEQEDSG	Negative
887.	I7HPV9:1035	SGDELPCDMRIPSDKQDKLHGCLEHLFNQ	Negative
888.	I7HPV9:1038	ELPCDMRIPSDKQDKLHGCLEHLFNQVDS	Negative
889.	I7HPV9:1058	EHLFNQVDSIHALLKGPVMSRAFEETRHF	Negative
890.	I7HPV9:1075	VMSRAFEETRHFPMKHSWQEFKQKEECTV	Negative
891.	I7HPV9:1082	ETRHFPMKHSWQEFKQKEECTVRGRNLIQ	Negative
892.	I7HPV9:1084	RHFPMKHSWQEFKQKEECTVRGRNLIQIS	Negative
893.	I7HPV9:1125	RTLVDNIQQYVEDGKNQLLLALLKCTDTE	Negative
894.	I7HPV9:1134	YVEDGKNQLLLALLKCTDTELQLRRDAVF	Negative
895.	I7HPV9:1187	NNGEYEESSRDASRKWLEQVAATGVLLHW	Negative
896.	I7HPV9:1211	VLLHWQSLLAPASVKEERTMLEDIWVTLS	Negative
897.	I7HPV9:1235	WVTLSELDNVTFSFKQLDENSVANTNVFY	Negative
898.	I7HPV9:1259	TNVFYHIEGSRQALKVVFYLDGFHFSRLP	Negative
899.	I7HPV9:1291	EGGASLRLHTVLFTKALESVEGPPPPGNQ	Negative
900.	I7HPV9:1321	AEELQQEINAQSLEKVQQYYRKLRAFYLE	Negative
901.	I7HPV9:1328	INAQSLEKVQQYYRKLRAFYLERSNLPTD	Negative
902.	I7HPV9:1349	ERSNLPTDAGATAVKIDQLIRPINALDEL	Negative
903.	I7HPV9:1368	IRPINALDELYRLMKTFVHPKAGAAGSLG	Negative
904.	I7HPV9:1374	LDELYRLMKTFVHPKAGAAGSLGAGLIPV	Negative
905.	I7HPV9:1432	EQAAILARSHGLLPKCVMQATDIMRKQGP	Negative
906.	I7HPV9:1443	LLPKCVMQATDIMRKQGPRVEILAKNLRI	Negative
907.	I7HPV9:1453	DIMRKQGPRVEILAKNLRIKDPMPQGAPR	Negative
908.	I7HPV9:1458	QGPRVEILAKNLRIKDPMPQGAPRLYQLC	Negative
909.	J3KMM5:7	XXXXXXXXMENAHTKTVEEVLGHFGVNES	Negative
910.	J3KMM5:30	FGVNESTGLSLEQVKKLKERWGSNELPAE	Negative
911.	J3KMM5:31	GVNESTGLSLEQVKKLKERWGSNELPAEE	Negative
912.	J3KMM5:33	NESTGLSLEQVKKLKERWGSNELPAEEGK	Negative
913.	J3KMM5:47	KERWGSNELPAEEGKTLLELVIEQFEDLL	Negative

914.	J3KMM5:120	VWQERNAENAIEALKEYEPEMGKVYRQDR	Negative
915.	J3KMM5:128	NAIEALKEYEPEMGKVYRQDRKSVQRIKA	Negative
916.	J3KMM5:135	EYEPEMGKVYRQDRKSVQRIKAKDIVPGD	Negative
917.	J3KMM5:141	GKVYRQDRKSVQRIKAKDIVPGDIVEIAV	Negative
918.	J3KMM5:143	VYRQDRKSVQRIKAKDIVPGDIVEIAVGD	Negative
919.	J3KMM5:158	DIVPGDIVEIAVGDKVPADIRLTSIKSTT	Negative
920.	J3KMM5:169	VGDKVPADIRLTSIKSTTLRVDQSILTGE	Negative
921.	J3KMM5:189	VDQSILTGESVSVIKHTDPVPDPRAVNQD	Negative
922.	J3KMM5:204	HTDPVPDPRAVNQDKKNMLFSGTNIAAGK	Negative
923.	J3KMM5:218	KKNMLFSGTNIAAGKAMGVVVATGVNTEI	Negative
924.	J3KMM5:234	MGVVVATGVNTEIGKIRDEMVATEQERTP	Negative
925.	J3KMM5:252	EMVATEQERTPLQQKLDEFGEQLSKVISL	Negative
926.	J3KMM5:262	PLQQKLDEFGEQLSKVISLICIAVWIINI	Negative
927.	J3KMM5:297	VHGGSWIRGAIYYFKIAVALAVAAIPEGL	Negative
928.	J3KMM5:328	VITTCLALGTRRMAKKNAIVRSLPSVETL	Negative
929.	J3KMM5:329	ITTCLALGTRRMAKKNAIVRSLPSVETLG	Negative
930.	J3KMM5:352	SVETLGCTSVICSDKTGTLTTNQMSVCRM	Negative
931.	J3KMM5:371	TTNQMSVCRMFILDKVEGDTCSLNEFSIT	Negative
932.	J3KMM5:397	SITGSTYAPIGEVQKDDKPVKCHQYDGLV	Negative
933.	J3KMM5:400	GSTYAPIGEVQKDDKPVKCHQYDGLVELA	Negative
934.	J3KMM5:403	YAPIGEVQKDDKPVKCHQYDGLVELATIC	Negative
935.	J3KMM5:431	CALCNDSALDYNEAKGVYEKVGEATETAL	Negative
936.	J3KMM5:436	DSALDYNEAKGVYEKVGEATETALTCLVE	Negative
937.	J3KMM5:451	VGEATETALTCLVEKMNVFDTELKGLSKI	Negative
938.	J3KMM5:460	TCLVEKMNVFDTELKGLSKIERANACNSV	Negative
939.	J3KMM5:464	EKMNVFDTELKGLSKIERANACNSVIKQL	Negative
940.	J3KMM5:476	LSKIERANACNSVIKQLMKKEFTLEFSRD	Negative
941.	J3KMM5:480	ERANACNSVIKQLMKKEFTLEFSRDRKSM	Negative
942.	J3KMM5:481	RANACNSVIKQLMKKEFTLEFSRDRKSMS	Negative
943.	J3KMM5:492	LMKKEFTLEFSRDRKSMSVYCTPNKPSRT	Negative
944.	J3KMM5:502	SRDRKSMSVYCTPNKPSRTSMSKMFVKGA	Negative
945.	J3KMM5:510	VYCTPNKPSRTSMSKMFVKGAPEGVIDRC	Negative
946.	J3KMM5:514	PNKPSRTSMSKMFVKGAPEGVIDRCTHIR	Negative
947.	J3KMM5:533	GVIDRCTHIRVGSTKVPMTPGVKQKIMSV	Negative
948.	J3KMM5:541	IRVGSTKVPMTPGVKQKIMSVIREWGSGS	Negative
949.	J3KMM5:543	VGSTKVPMTPGVKQKIMSVIREWGSGSDT	Negative
950.	J3KMM5:571	TLRCLALATHDNPLKREEMHLEDSANFIK	Negative
951.	J3KMM5:585	KREEMHLEDSANFIKYETNLTFVGCVGML	Negative
952.	J3KMM5:611	GMLDPPRIEVASSVKLCRQAGIRVIMITG	Negative
953.	J3KMM5:628	RQAGIRVIMITGDNKGTAVAICRRIGIFG	Negative
954.	J3KMM5:650	RRIGIFGQDEDVTSKAFTGREFDELSPSA	Negative
955.	J3KMM5:683	CLNARCFARVEPSHKSKIVEFLQSFDEIT	Negative
956.	J3KMM5:685	NARCFARVEPSHKSKIVEFLQSFDEITAM	Negative
957.	J3KMM5:711	TAMTGDGVNDAPALKKSEIGIAMGSGTAV	Negative
958.	J3KMM5:712	AMTGDGVNDAPALKKSEIGIAMGSGTAVA	Negative
959.	J3KMM5:727	SEIGIAMGSGTAVAKTASEMVLADDNFST	Negative
960.	J3KMM5:757	VAAVEEGRAIYNNMKQFIRYLISSNVGEV	Negative
961.	J3KMM5:818	TALGFNPPDLDIMNKPPRNPKEPLISGWL	Negative
962.	J3KMM5:824	PPDLDIMNKPPRNPKEPLISGWLFFRYLA	Negative
963.	J3KMM5:876	PRVSFYQLSHFLQCKEDNPDFDGVDCAIF	Negative
964.	J3KMM5:971	QITPLNLTQWLMVLKISLPVILMDETLKF	Negative
965.	J3KMM5:984	LKISLPVILMDETLKFVARNYLEQPDCLE	Negative
966.	J3KRX8:32	APRFGGSRAGPLSGKKFGNPGEKLVKKKW	Negative
967.	J3KRX8:33	PRFGGSRAGPLSGKKFGNPGEKLVKKKWN	Negative

968.	J3KRX8:40	AGPLSGKKFGNPGEKLVKKKWNLDELPKF	Negative
969.	J3KRX8:43	LSGKKFGNPGEKLVKKKWNLDELPKFEKN	Negative
970.	J3KRX8:44	SGKKFGNPGEKLVKKKWNLDELPKFEKNF	Negative
971.	J3KRX8:45	GKKFGNPGEKLVKKKWNLDELPKFEKNFY	Negative
972.	J3KRX8:53	EKLVKKKWNLDELPKFEKNFYQEHPDLAR	Negative
973.	J3KRX8:56	VKKKWNLDELPKFEKNFYQEHPDLARRTA	Negative
974.	J3KRX8:80	ARRTAQEVETYRRSKEITVRGHNCPKPVL	Negative
975.	J3QMG3:12	XXXMCNTPTYCDLGKAAKDVFNKGYGFGM	Negative
976.	J3QMG3:28	AKDVFNKGYGFGMVKIDLKTKSCSGVVEF	Negative
977.	J3QMG3:32	FNKGYGFGMVKIDLKTKSCSGVVEFSTSG	Negative
978.	J3QMG3:34	KGYGFGMVKIDLKTKSCSGVVEFSTSGHA	Negative
979.	J3QMG3:54	VEFSTSGHAYTDTGKASGNLETKYKVCNY	Negative
980.	J3QMG3:62	AYTDTGKASGNLETKYKVCNYGLTFTQKW	Negative
981.	J3QMG3:64	TDTGKASGNLETKYKVCNYGLTFTQKWNT	Negative
982.	J3QMG3:75	TKYKVCNYGLTFTQKWNTDNTLGTEISWE	Negative
983.	J3QMG3:91	NTDNTLGTEISWENKLAEGLKLTLDTIFV	Negative
984.	J3QMG3:97	GTEISWENKLAEGLKLTLDTIFVPNTGKK	Negative
985.	J3QMG3:110	LKLTLDTIFVPNTGKKSGKLKASYRRDCF	Negative
986.	J3QMG3:111	KLTLDTIFVPNTGKKSGKLKASYRRDCFS	Negative
987.	J3QMG3:114	LDTIFVPNTGKKSGKLKASYRRDCFSLGS	Negative
988.	J3QMG3:116	TIFVPNTGKKSGKLKASYRRDCFSLGSNV	Negative
989.	J3QMG3:162	EGWLAGYQMSFDTAKSKLSQNNFALGYKA	Negative
990.	J3QMG3:164	WLAGYQMSFDTAKSKLSQNNFALGYKAAD	Negative
991.	J3QMG3:175	AKSKLSQNNFALGYKAADFQLHTHVNDGT	Negative
992.	J3QMG3:198	HVNDGTEFGGSIYQKVNERIETSINLAWT	Negative
993.	J3QMG3:225	WTAGSNNTRFGIAAKYKLDCRTSLSAKVN	Negative
994.	J3QMG3:227	AGSNNTRFGIAAKYKLDCRTSLSAKVNNA	Negative
995.	J3QMG3:237	AAKYKLDCRTSLSAKVNNASLIGLGYTQT	Negative
996.	J3QMG3:257	LIGLGYTQTLRPGVKLTLSALIDGKNFNA	Negative
997.	J3QMG3:267	RPGVKLTLSALIDGKNFNAGGHKVGLGFE	Negative
998.	J3QMG3:275	SALIDGKNFNAGGHKVGLGFELEAXXXXX	Negative
999.	J3QNG0:4	XXXXXXXXXXXMSSKGSVVLAYSGGLDTS	Negative
1000.	J3QNG0:25	YSGGLDTSCILVWLKEQGYDVIAYLANIG	Negative
1001.	J3QNG0:41	QGYDVIAYLANIGQKEDFEEARKKALKLG	Negative
1002.	J3QNG0:49	LANIGQKEDFEEARKKALKLGAKKVFIED	Negative
1003.	J3QNG0:50	ANIGQKEDFEEARKKALKLGAKKVFIEDV	Negative
1004.	J3QNG0:53	GQKEDFEEARKKALKLGAKKVFIEDVSKE	Negative
1005.	J3QNG0:57	DFEEARKKALKLGAKKVFIEDVSKEFVEE	Negative
1006.	J3QNG0:58	FEEARKKALKLGAKKVFIEDVSKEFVEEF	Negative
1007.	J3QNG0:66	LKLGAKKVFIEDVSKEFVEEFIWPAVQSS	Negative
1008.	J3QNG0:112	IARRQVEIAQREGAKYVSHGATGKGNDQV	Negative
1009.	J3QNG0:121	QREGAKYVSHGATGKGNDQVRFELTCYSL	Negative
1010.	J3QNG0:140	VRFELTCYSLAPQIKVIAPWRMPEFYNRF	Negative
1011.	J3QNG0:155	VIAPWRMPEFYNRFKGRNDLMEYAKQHGI	Negative
1012.	J3QNG0:165	YNRFKGRNDLMEYAKQHGIPIPVSPKSPW	Negative
1013.	J3QNG0:176	EYAKQHGIPIPVSPKSPWSMDENLMHISY	Negative
1014.	J3QNG0:199	LMHISYEAGILENPKNQAPPGLYTKTQDP	Negative
1015.	J3QNG0:209	LENPKNQAPPGLYTKTQDPAKAPNTPDVL	Negative
1016.	J3QNG0:215	QAPPGLYTKTQDPAKAPNTPDVLEIEFKK	Negative
1017.	J3QNG0:228	AKAPNTPDVLEIEFKKGVPVKVTNIKDGT	Negative
1018.	J3QNG0:229	KAPNTPDVLEIEFKKGVPVKVTNIKDGTT	Negative
1019.	J3QNG0:234	PDVLEIEFKKGVPVKVTNIKDGTTRTTSL	Negative
1020.	J3QNG0:239	IEFKKGVPVKVTNIKDGTTRTTSLELFMY	Negative
1021.	J3QNG0:260	TSLELFMYLNEVAGKHGVGRIDIVENRFI	Negative

1022.	J3QNG0:277	VGRIDIVENRFIGMKSRGIYETPAGTILY	Negative
1023.	J3QNG0:308	HLDIEAFTMDREVRKIKQGLGLKFAELVY	Negative
1024.	J3QNG0:310	DIEAFTMDREVRKIKQGLGLKFAELVYTG	Negative
1025.	J3QNG0:316	MDREVRKIKQGLGLKFAELVYTGFWHSPE	Negative
1026.	J3QNG0:348	VRHCIQKSQERVEGKVQVSVFKGQVYILG	Negative
1027.	J3QNG0:355	SQERVEGKVQVSVFKGQVYILGRESPLSL	Negative
1028.	J3QNG0:400	IDATGFININSLRLKEYHRLQSKVTAKXX	Negative
1029.	J3QNG0:408	INSLRLKEYHRLQSKVTAKXXXXXXXXXX	Negative
1030.	J3QNG0:412	RLKEYHRLQSKVTAKXXXXXXXXXXXXXX	Negative
1031.	O09172:33	HLQTGNLLNWGRLRKKCPSTHSEELRDCI	Negative
1032.	O09172:34	LQTGNLLNWGRLRKKCPSTHSEELRDCIQ	Negative
1033.	O09172:49	CPSTHSEELRDCIQKTLNEWSSQISPDLV	Negative
1034.	O09172:80	FPDVLECTMSHAVEKINPDEREEMKVSAK	Negative
1035.	O09172:90	HAVEKINPDEREEMKVSAKLFIVGSNSSS	Negative
1036.	O09172:94	KINPDEREEMKVSAKLFIVGSNSSSSTRS	Negative
1037.	O09172:157	LQPYWEELENLVQSKKIVAIGTSDLDKTQ	Negative
1038.	O09172:158	QPYWEELENLVQSKKIVAIGTSDLDKTQL	Negative
1039.	O09172:169	QSKKIVAIGTSDLDKTQLEQLYQWAQVKP	Negative
1040.	O09172:182	DKTQLEQLYQWAQVKPNSNQVNLASCCVM	Negative
1041.	O09172:205	ASCCVMPPDLTAFAKQFDIQLLTHNDPKE	Negative
1042.	O09172:218	AKQFDIQLLTHNDPKELLSEASFQEALQE	Negative
1043.	O09172:255	DWVPLWLLRYSVIVKSRGIIKSKGYILQA	Negative
1044.	O09172:261	LLRYSVIVKSRGIIKSKGYILQAKRRGSX	Negative
1045.	O09172:270	SRGIIKSKGYILQAKRRGSXXXXXXXXXX	Negative
1046.	O70250:33	ENRFCGWFDAELSEKGAEEAKRGATAIKD	Negative
1047.	O70250:39	WFDAELSEKGAEEAKRGATAIKDAKIEFD	Negative
1048.	O70250:46	EKGAEEAKRGATAIKDAKIEFDICYTSVL	Negative
1049.	O70250:49	AEEAKRGATAIKDAKIEFDICYTSVLKRA	Negative
1050.	O70250:61	DAKIEFDICYTSVLKRAIRTLWTILDVTD	Negative
1051.	O70250:106	YGGLTGLNKAETAAKHGEEQVKIWRRSFD	Negative
1052.	O70250:113	NKAETAAKHGEEQVKIWRRSFDTPPPPMD	Negative
1053.	O70250:129	WRRSFDTPPPPMDEKHNYYTSISKDRRYA	Negative
1054.	O70250:138	PPMDEKHNYYTSISKDRRYAGLKPEELPT	Negative
1055.	O70250:146	YYTSISKDRRYAGLKPEELPTCESLKDTI	Negative
1056.	O70250:157	AGLKPEELPTCESLKDTIARALPFWNEEI	Negative
1057.	O70250:174	IARALPFWNEEIAPKIKAGQRVLIAAHGN	Negative
1058.	O70250:176	RALPFWNEEIAPKIKAGQRVLIAAHGNSL	Negative
1059.	O70250:195	VLIAAHGNSLRGIVKHLEGMSDQAIMELN	Negative
1060.	O70250:225	PTGIPIVYELDQNLKPTKPMRFLGDEETV	Negative
1061.	O70250:228	IPIVYELDQNLKPTKPMRFLGDEETVRKA	Negative
1062.	O70250:241	TKPMRFLGDEETVRKAMEAVAAQGKAKXX	Negative
1063.	O70250:251	ETVRKAMEAVAAQGKAKXXXXXXXXXXXX	Negative
1064.	O70250:253	VRKAMEAVAAQGKAKXXXXXXXXXXXXXX	Negative
1065.	O88844:4	XXXXXXXXXXXMSRKIQGGSVVEMQGDEM	Negative
1066.	O88844:27	MQGDEMTRIIWELIKEKLILPYVELDLHS	Negative
1067.	O88844:29	GDEMTRIIWELIKEKLILPYVELDLHSYD	Negative
1068.	O88844:58	LGIENRDATNDQVTKDAAEAIKKYNVGVK	Negative
1069.	O88844:65	ATNDQVTKDAAEAIKKYNVGVKCATITPD	Negative
1070.	O88844:66	TNDQVTKDAAEAIKKYNVGVKCATITPDE	Negative
1071.	O88844:72	KDAAEAIKKYNVGVKCATITPDEKRVEEF	Negative
1072.	O88844:81	YNVGVKCATITPDEKRVEEFKLKQMWKSP	Negative
1073.	O88844:87	CATITPDEKRVEEFKLKQMWKSPNGTIRN	Negative
1074.	O88844:89	TITPDEKRVEEFKLKQMWKSPNGTIRNIL	Negative
1075.	O88844:93	DEKRVEEFKLKQMWKSPNGTIRNILGGTV	Negative

1076.	O88844:115	NILGGTVFREAIICKNIPRLVTGWVKPII	Negative
1077.	O88844:126	IICKNIPRLVTGWVKPIIIGRHAYGDQYR	Negative
1078.	O88844:151	DQYRATDFVVPGPGKVEITYTPKDGTQKV	Negative
1079.	O88844:159	VVPGPGKVEITYTPKDGTQKVTYMVHDFE	Negative
1080.	O88844:164	GKVEITYTPKDGTQKVTYMVHDFEEGGGV	Negative
1081.	O88844:187	EEGGGVAMGMYNQDKSIEDFAHSSFQMAL	Negative
1082.	O88844:203	IEDFAHSSFQMALSKGWPLYLSTKNTILK	Negative
1083.	O88844:212	QMALSKGWPLYLSTKNTILKKYDGRFKDI	Negative
1084.	O88844:217	KGWPLYLSTKNTILKKYDGRFKDIFQEIY	Negative
1085.	O88844:218	GWPLYLSTKNTILKKYDGRFKDIFQEIYD	Negative
1086.	O88844:233	YDGRFKDIFQEIYDKKYKSQFEAQKICYE	Negative
1087.	O88844:234	DGRFKDIFQEIYDKKYKSQFEAQKICYEH	Negative
1088.	O88844:236	RFKDIFQEIYDKKYKSQFEAQKICYEHRL	Negative
1089.	O88844:243	EIYDKKYKSQFEAQKICYEHRLIDDMVAQ	Negative
1090.	O88844:260	YEHRLIDDMVAQAMKSEGGFIWACKNYDG	Negative
1091.	O88844:270	AQAMKSEGGFIWACKNYDGDVQSDSVAQG	Negative
1092.	O88844:301	SLGMMTSVLICPDGKTVEAEAAHGTVTRH	Negative
1093.	O88844:321	AAHGTVTRHYRMYQKGQETSTNPIASIFA	Negative
1094.	O88844:345	ASIFAWSRGLAHRAKLDNNTELSFFAKAL	Negative
1095.	O88844:357	RAKLDNNTELSFFAKALEDVCIETIEAGF	Negative
1096.	O88844:374	EDVCIETIEAGFMTKDLAACIKGLPNVQR	Negative
1097.	O88844:381	IEAGFMTKDLAACIKGLPNVQRSDYLNTF	Negative
1098.	O88844:400	VQRSDYLNTFEFMDKLGENLKAKLAQAKL	Negative
1099.	O88844:406	LNTFEFMDKLGENLKAKLAQAKLXXXXXX	Negative
1100.	O88844:408	TFEFMDKLGENLKAKLAQAKLXXXXXXXX	Negative
1101.	O88844:413	DKLGENLKAKLAQAKLXXXXXXXXXXXXX	Negative
1102.	P01942:8	XXXXXXXMVLSGEDKSNIKAAWGKIGGHG	Negative
1103.	P01942:41	AEALERMFASFPTTKTYFPHFDVSHGSAQ	Negative
1104.	P01942:61	FDVSHGSAQVKGHGKKVADALASAAGHLD	Negative
1105.	P01942:62	DVSHGSAQVKGHGKKVADALASAAGHLDD	Negative
1106.	P01942:91	LPGALSALSDLHAHKLRVDPVNFKLLSHC	Negative
1107.	P01942:100	DLHAHKLRVDPVNFKLLSHCLLVTLASHH	Negative
1108.	P01942:128	HPADFTPAVHASLDKFLASVSTVLTSKYR	Negative
1109.	P01942:140	LDKFLASVSTVLTSKYRXXXXXXXXXXXX	Negative
1110.	P06733:5	XXXXXXXXXMSILKIHAREIFDSRGNPT	Negative
1111.	P06733:28	SRGNPTVEVDLFTSKGLFRAAVPSGASTG	Negative
1112.	P06733:54	STGIYEALELRDNDKTRYMGKGVSKAVEH	Negative
1113.	P06733:60	ALELRDNDKTRYMGKGVSKAVEHINKTIA	Negative
1114.	P06733:64	RDNDKTRYMGKGVSKAVEHINKTIAPALV	Negative
1115.	P06733:71	YMGKGVSKAVEHINKTIAPALVSKKLNVT	Negative
1116.	P06733:80	VEHINKTIAPALVSKKLNVTEQEKIDKLM	Negative
1117.	P06733:81	EHINKTIAPALVSKKLNVTEQEKIDKLMI	Negative
1118.	P06733:89	PALVSKKLNVTEQEKIDKLMIEMDGTENK	Negative
1119.	P06733:92	VSKKLNVTEQEKIDKLMIEMDGTENKSKF	Negative
1120.	P06733:103	KIDKLMIEMDGTENKSKFGANAILGVSLA	Negative
1121.	P06733:105	DKLMIEMDGTENKSKFGANAILGVSLAVC	Negative
1122.	P06733:120	FGANAILGVSLAVCKAGAVEKGVPLYRHI	Negative
1123.	P06733:126	LGVSLAVCKAGAVEKGVPLYRHIADLAGN	Negative
1124.	P06733:162	PAFNVINGGSHAGNKLAMQEFMILPVGAA	Negative
1125.	P06733:193	REAMRIGAEVYHNLKNVIKEKYGKDATNV	Negative
1126.	P06733:197	RIGAEVYHNLKNVIKEKYGKDATNVGDEG	Negative
1127.	P06733:199	GAEVYHNLKNVIKEKYGKDATNVGDEGGF	Negative
1128.	P06733:202	VYHNLKNVIKEKYGKDATNVGDEGGFAPN	Negative
1129.	P06733:221	VGDEGGFAPNILENKEGLELLKTAIGKAG	Negative

1130.	P06733:228	APNILENKEGLELLKTAIGKAGYTDKVVI	Negative
1131.	P06733:233	ENKEGLELLKTAIGKAGYTDKVVIGMDVA	Negative
1132.	P06733:239	ELLKTAIGKAGYTDKVVIGMDVAASEFFR	Negative
1133.	P06733:256	IGMDVAASEFFRSGKYDLDFKSPDDPSRY	Negative
1134.	P06733:262	ASEFFRSGKYDLDFKSPDDPSRYISPDQL	Negative
1135.	P06733:281	PSRYISPDQLADLYKSFIKDYPVVSIEDP	Negative
1136.	P06733:285	ISPDQLADLYKSFIKDYPVVSIEDPFDQD	Negative
1137.	P06733:306	IEDPFDQDDWGAWQKFTASAGIQVVGDDL	Negative
1138.	P06733:326	GIQVVGDDLTVTNPKRIAKAVNEKSCNCL	Negative
1139.	P06733:330	VGDDLTVTNPKRIAKAVNEKSCNCLLLKV	Negative
1140.	P06733:358	VNQIGSVTESLQACKLAQANGWGVMVSHR	Negative
1141.	P06733:394	FIADLVVGLCTGQIKTGAPCRSERLAKYN	Negative
1142.	P06733:406	QIKTGAPCRSERLAKYNQLLRIEEELGSK	Negative
1143.	P06733:420	KYNQLLRIEEELGSKAKFAGRNFRNPLAK	Negative
1144.	P06733:422	NQLLRIEEELGSKAKFAGRNFRNPLAKXX	Negative
1145.	P06733:434	KAKFAGRNFRNPLAKXXXXXXXXXXXXXX	Negative
1146.	P07724:2	XXXXXXXXXXXXXMKWVTFLLLLFVSGSA	Negative
1147.	P07724:28	GSAFSRGVFRREAHKSEIAHRYNDLGEQH	Negative
1148.	P07724:44	EIAHRYNDLGEQHFKGLVLIAFSQYLQKC	Negative
1149.	P07724:57	FKGLVLIAFSQYLQKCSYDEHAKLVQEVT	Negative
1150.	P07724:65	FSQYLQKCSYDEHAKLVQEVTDFAKTCVA	Negative
1151.	P07724:75	DEHAKLVQEVTDFAKTCVADESAANCDKS	Negative
1152.	P07724:88	AKTCVADESAANCDKSLHTLFGDKLCAIP	Negative
1153.	P07724:97	AANCDKSLHTLFGDKLCAIPNLRENYGEL	Negative
1154.	P07724:117	NLRENYGELADCCTKQEPERNECFLQHKD	Negative
1155.	P07724:130	TKQEPERNECFLQHKDDNPSLPPFERPEA	Negative
1156.	P07724:152	PFERPEAEAMCTSFKENPTTFMGHYLHEV	Negative
1157.	P07724:198	QYNEILTQCCAEADKESCLTPKLDGVKEK	Negative
1158.	P07724:205	QCCAEADKESCLTPKLDGVKEKALVSSVR	Negative
1159.	P07724:210	ADKESCLTPKLDGVKEKALVSSVRQRMKC	Negative
1160.	P07724:212	KESCLTPKLDGVKEKALVSSVRQRMKCSS	Negative
1161.	P07724:223	VKEKALVSSVRQRMKCSSMQKFGERAFKA	Negative
1162.	P07724:229	VSSVRQRMKCSSMQKFGERAFKAWAVARL	Negative
1163.	P07724:257	LSQTFPNADFAEITKLATDLTKVNKECCH	Negative
1164.	P07724:264	ADFAEITKLATDLTKVNKECCHGDLLECA	Negative
1165.	P07724:267	AEITKLATDLTKVNKECCHGDLLECADDR	Negative
1166.	P07724:286	GDLLECADDRAELAKYMCENQATISSKLQ	Negative
1167.	P07724:298	LAKYMCENQATISSKLQTCCDKPLLKKAH	Negative
1168.	P07724:305	NQATISSKLQTCCDKPLLKKAHCLSEVEH	Negative
1169.	P07724:309	ISSKLQTCCDKPLLKKAHCLSEVEHDTMP	Negative
1170.	P07724:310	SSKLQTCCDKPLLKKAHCLSEVEHDTMPA	Negative
1171.	P07724:341	PAIAADFVEDQEVCKNYAEAKDVFLGTFL	Negative
1172.	P07724:347	FVEDQEVCKNYAEAKDVFLGTFLYEYSRR	Negative
1173.	P07724:375	RHPDYSVSLLLRLAKKYEATLEKCCAEAN	Negative
1174.	P07724:376	HPDYSVSLLLRLAKKYEATLEKCCAEANP	Negative
1175.	P07724:383	LLLRLAKKYEATLEKCCAEANPPACYGTV	Negative
1176.	P07724:409	GTVLAEFQPLVEEPKNLVKTNCDLYEKLG	Negative
1177.	P07724:413	AEFQPLVEEPKNLVKTNCDLYEKLGEYGF	Negative
1178.	P07724:421	EPKNLVKTNCDLYEKLGEYGFQNAILVRY	Negative
1179.	P07724:438	EYGFQNAILVRYTQKAPQVSTPTLVEAAR	Negative
1180.	P07724:460	TLVEAARNLGRVGTKCCTLPEDQRLPCVE	Negative
1181.	P07724:490	YLSAILNRVCLLHEKTPVSEHVTKCCSGS	Negative
1182.	P07724:499	CLLHEKTPVSEHVTKCCSGSLVERRPCFS	Negative
1183.	P07724:524	PCFSALTVDETYVPKEFKAETFTFHSDIC	Negative

1184.	P07724:527	SALTVDETYVPKEFKAETFTFHSDICTLP	Negative
1185.	P07724:543	ETFTFHSDICTLPEKEKQIKKQTALAELV	Negative
1186.	P07724:545	FTFHSDICTLPEKEKQIKKQTALAELVKH	Negative
1187.	P07724:548	HSDICTLPEKEKQIKKQTALAELVKHKPK	Negative
1188.	P07724:549	SDICTLPEKEKQIKKQTALAELVKHKPKA	Negative
1189.	P07724:558	EKQIKKQTALAELVKHKPKATAEQLKTVM	Negative
1190.	P07724:560	QIKKQTALAELVKHKPKATAEQLKTVMDD	Negative
1191.	P07724:562	KKQTALAELVKHKPKATAEQLKTVMDDFA	Negative
1192.	P07724:569	ELVKHKPKATAEQLKTVMDDFAQFLDTCC	Negative
1193.	P07724:584	TVMDDFAQFLDTCCKAADKDTCFSTEGPN	Negative
1194.	P07724:588	DFAQFLDTCCKAADKDTCFSTEGPNLVTR	Negative
1195.	P07724:604	TCFSTEGPNLVTRCKDALAXXXXXXXXXX	Negative
1196.	P11214:2	XXXXXXXXXXXXXMKRELLCVLLLCGLAF	Negative
1197.	P11214:114	FSDFVCQCPDGFVGKRCDIDTRATCFEEQ	Negative
1198.	P11214:156	GAECINWNSSVLSLKPYNARRPNAIKLGL	Negative
1199.	P11214:184	LGNHNYCRNPDRDLKPWCYVFKAGKYTTE	Negative
1200.	P11214:191	RNPDRDLKPWCYVFKAGKYTTEFCSTPAC	Negative
1201.	P11214:194	DRDLKPWCYVFKAGKYTTEFCSTPACPKG	Negative
1202.	P11214:207	GKYTTEFCSTPACPKGKSEDCYVGKGVTY	Negative
1203.	P11214:209	YTTEFCSTPACPKGKSEDCYVGKGVTYRG	Negative
1204.	P11214:217	PACPKGKSEDCYVGKGVTYRGTHSLTTSQ	Negative
1205.	P11214:245	QASCLPWNSIVLMGKSYTAWRTNSQALGL	Negative
1206.	P11214:280	RNPDGDARPWCHVMKDRKLTWEYCDMSPC	Negative
1207.	P11214:283	DGDARPWCHVMKDRKLTWEYCDMSPCSTC	Negative
1208.	P11214:303	CDMSPCSTCGLRQYKRPQFRIKGGLYTDI	Negative
1209.	P11214:310	TCGLRQYKRPQFRIKGGLYTDITSHPWQA	Negative
1210.	P11214:329	TDITSHPWQAAIFVKNKRSPGERFLCGGV	Negative
1211.	P11214:331	ITSHPWQAAIFVKNKRSPGERFLCGGVLI	Negative
1212.	P11214:367	AAHCFLERFPPNHLKVVLGRTYRVVPGEE	Negative
1213.	P11214:389	RVVPGEEEQTFEIEKYIVHEEFDDDTYDN	Negative
1214.	P11214:415	YDNDIALLQLRSQSKQCAQESSSVGTACL	Negative
1215.	P11214:449	QLPDWTECELSGYGKHEASSPFFSDRLKE	Negative
1216.	P11214:462	GKHEASSPFFSDRLKEAHVRLYPSSRCTS	Negative
1217.	P11214:482	LYPSSRCTSQHLFNKTVTNNMLCAGDTRS	Negative
1218.	P11214:520	CQGDSGGPLVCMINKQMTLTGIISWGLGC	Negative
1219.	P11214:537	TLTGIISWGLGCGQKDVPGVYTKVTNYLD	Negative
1220.	P11214:545	GLGCGQKDVPGVYTKVTNYLDWIHDNMKQ	Negative
1221.	P11214:558	TKVTNYLDWIHDNMKQXXXXXXXXXXXXX	Negative
1222.	P11881:4	XXXXXXXXXXXMSDKMSSFLHIGDICSLY	Negative
1223.	P11881:51	CVVQPEAGDLNNPPKKFRDCLFKLCPMNR	Negative
1224.	P11881:52	VVQPEAGDLNNPPKKFRDCLFKLCPMNRY	Negative
1225.	P11881:59	DLNNPPKKFRDCLFKLCPMNRYSAQKQFW	Negative
1226.	P11881:70	CLFKLCPMNRYSAQKQFWKAAKPGANSTT	Negative
1227.	P11881:74	LCPMNRYSAQKQFWKAAKPGANSTTDAVL	Negative
1228.	P11881:77	MNRYSAQKQFWKAAKPGANSTTDAVLLNK	Negative
1229.	P11881:91	KPGANSTTDAVLLNKLHHAADLEKKQNET	Negative
1230.	P11881:100	AVLLNKLHHAADLEKKQNETENRKLLGTV	Negative
1231.	P11881:101	VLLNKLHHAADLEKKQNETENRKLLGTVI	Negative
1232.	P11881:109	AADLEKKQNETENRKLLGTVIQYGNVIQL	Negative
1233.	P11881:127	TVIQYGNVIQLLHLKSNKYLTVNKRLPAL	Negative
1234.	P11881:130	QYGNVIQLLHLKSNKYLTVNKRLPALLEK	Negative
1235.	P11881:136	QLLHLKSNKYLTVNKRLPALLEKNAMRVT	Negative
1236.	P11881:144	KYLTVNKRLPALLEKNAMRVTLDEAGNEG	Negative

1237.	P11881:168	AGNEGSWFYIQPFYKLRSIGDSVVIGDKV	Negative
1238.	P11881:181	YKLRSIGDSVVIGDKVVLNPVNAGQPLHA	Negative
1239.	P11881:219	GCNEVNSVNCNTSWKIVLFMKWSDNKDDI	Negative
1240.	P11881:225	SVNCNTSWKIVLFMKWSDNKDDILKGGDV	Negative
1241.	P11881:230	TSWKIVLFMKWSDNKDDILKGGDVVRLFH	Negative
1242.	P11881:235	VLFMKWSDNKDDILKGGDVVRLFHAEQEK	Negative
1243.	P11881:249	KGGDVVRLFHAEQEKFLTCDEHRKKQHVF	Negative
1244.	P11881:258	HAEQEKFLTCDEHRKKQHVFLRTTGRQSA	Negative
1245.	P11881:259	AEQEKFLTCDEHRKKQHVFLRTTGRQSAT	Negative
1246.	P11881:279	RTTGRQSATSATSSKALWEVEVVQHDPCR	Negative
1247.	P11881:306	CRGGAGYWNSLFRFKHLATGHYLAAEVDP	Negative
1248.	P11881:350	DQDASRSRLRNAQEKMVYSLVSVPEGNDI	Negative
1249.	P11881:408	CTNTWVHSTNIPIDKEEEKPVMLKIGTSP	Negative
1250.	P11881:412	WVHSTNIPIDKEEEKPVMLKIGTSPLKED	Negative
1251.	P11881:417	NIPIDKEEEKPVMLKIGTSPLKEDKEAFA	Negative
1252.	P11881:424	EEKPVMLKIGTSPLKEDKEAFAIVPVSPA	Negative
1253.	P11881:427	PVMLKIGTSPLKEDKEAFAIVPVSPAEVR	Negative
1254.	P11881:451	PAEVRDLDFANDASKVLGSIAGKLEKGTI	Negative
1255.	P11881:459	FANDASKVLGSIAGKLEKGTITQNERRSV	Negative
1256.	P11881:462	DASKVLGSIAGKLEKGTITQNERRSVTKL	Negative
1257.	P11881:475	EKGTITQNERRSVTKLLEDLVYFVTGGTN	Negative
1258.	P11881:501	GTNSGQDVLEVVFSKPNRERQKLMREQNI	Negative
1259.	P11881:508	VLEVVFSKPNRERQKLMREQNILKQIFKL	Negative
1260.	P11881:517	NRERQKLMREQNILKQIFKLLQAPFTDCG	Negative
1261.	P11881:521	QKLMREQNILKQIFKLLQAPFTDCGDGPM	Negative
1262.	P11881:576	HSQQDYRKNQEYIAKQFGFMQKQIGYDVL	Negative
1263.	P11881:583	KNQEYIAKQFGFMQKQIGYDVLAEDTITA	Negative
1264.	P11881:604	LAEDTITALLHNNRKLLEKHITAAEIDTF	Negative
1265.	P11881:608	TITALLHNNRKLLEKHITAAEIDTFVSLV	Negative
1266.	P11881:624	ITAAEIDTFVSLVRKNREPRFLDYLSDLC	Negative
1267.	P11881:643	RFLDYLSDLCVSMNKSIPVTQELICKAVL	Negative
1268.	P11881:654	SMNKSIPVTQELICKAVLNPTNADILIET	Negative
1269.	P11881:669	AVLNPTNADILIETKLVLSRFEFEGVSTG	Negative
1270.	P11881:705	EDEEEVWLFWRDSNKEIRSKSVRELAQDA	Negative
1271.	P11881:710	VWLFWRDSNKEIRSKSVRELAQDAKEGQK	Negative
1272.	P11881:720	EIRSKSVRELAQDAKEGQKEDRDILSYYR	Negative
1273.	P11881:724	KSVRELAQDAKEGQKEDRDILSYYRYQLN	Negative
1274.	P11881:802	MHVDRDPQEQVTPVKYARLWSEIPSEIAI	Negative
1275.	P11881:826	SEIAIDDYDSSGTSKDEIKERFAQTMEFV	Negative
1276.	P11881:830	IDDYDSSGTSKDEIKERFAQTMEFVEEYL	Negative
1277.	P11881:857	YLRDVVCQRFPFSDKEKNKLTFEVVNLAR	Negative
1278.	P11881:859	RDVVCQRFPFSDKEKNKLTFEVVNLARNL	Negative
1279.	P11881:861	VVCQRFPFSDKEKNKLTFEVVNLARNLIY	Negative
1280.	P11881:889	YFGFYNFSDLLRLTKILLAILDCVHVTTI	Negative
1281.	P11881:908	ILDCVHVTTIFPISKMTKGEENKGSNVMR	Negative
1282.	P11881:911	CVHVTTIFPISKMTKGEENKGSNVMRSIH	Negative
1283.	P11881:916	TIFPISKMTKGEENKGSNVMRSIHGVGEL	Negative
1284.	P11881:956	LPMTPMAAAPEGNVKQAEPEKEDIMVMDT	Negative
1285.	P11881:962	AAAPEGNVKQAEPEKEDIMVMDTKLKIIE	Negative
1286.	P11881:971	QAEPEKEDIMVMDTKLKIIEILQFILNVR	Negative
1287.	P11881:973	EPEKEDIMVMDTKLKIIEILQFILNVRLD	Negative
1288.	P11881:998	VRLDYRISCLLCIFKREFDESNSQSSETS	Negative
1289.	P11881:1098	FRHFSQRQEVLQAFKQVQLLVTSQDVDNY	Negative
1290.	P11881:1113	QVQLLVTSQDVDNYKQIKQDLDQLRSIVE	Negative

1291.	P11881:1116	LLVTSQDVDNYKQIKQDLDQLRSIVEKSE	Negative
1292.	P11881:1128	QIKQDLDQLRSIVEKSELWVYKGQGPDEP	Negative
1293.	P11881:1135	QLRSIVEKSELWVYKGQGPDEPMDGASGE	Negative
1294.	P11881:1153	PDEPMDGASGENEHKKTEEGTSKPLKHES	Negative
1295.	P11881:1154	DEPMDGASGENEHKKTEEGTSKPLKHEST	Negative
1296.	P11881:1161	SGENEHKKTEEGTSKPLKHESTSSYNYRV	Negative
1297.	P11881:1164	NEHKKTEEGTSKPLKHESTSSYNYRVVKE	Negative
1298.	P11881:1177	LKHESTSSYNYRVVKEILIRLSKLCVQES	Negative
1299.	P11881:1185	YNYRVVKEILIRLSKLCVQESASVRKSRK	Negative
1300.	P11881:1196	RLSKLCVQESASVRKSRKQQQRLLRNMGA	Negative
1301.	P11881:1199	KLCVQESASVRKSRKQQQRLLRNMGAHAV	Negative
1302.	P11881:1224	AHAVVLELLQIPYEKAEDTKMQEIMRLAH	Negative
1303.	P11881:1229	LELLQIPYEKAEDTKMQEIMRLAHEFLQN	Negative
1304.	P11881:1257	NFCAGNQQNQALLHKHINLFLNPGILEAV	Negative
1305.	P11881:1310	VHCIETHGRNVQYIKFLQTIVKAEGKFIK	Negative
1306.	P11881:1317	GRNVQYIKFLQTIVKAEGKFIKKCQDMVM	Negative
1307.	P11881:1321	QYIKFLQTIVKAEGKFIKKCQDMVMAELV	Negative
1308.	P11881:1324	KFLQTIVKAEGKFIKKCQDMVMAELVNSG	Negative
1309.	P11881:1325	FLQTIVKAEGKFIKKCQDMVMAELVNSGE	Negative
1310.	P11881:1388	HIHLVELLAVCTEGKNVYTEIKCNSLLPL	Negative
1311.	P11881:1395	LAVCTEGKNVYTEIKCNSLLPLDDIVRVV	Negative
1312.	P11881:1419	IVRVVTHEDCIPEVKIAYINFLNHCYVDT	Negative
1313.	P11881:1438	NFLNHCYVDTEVEMKEIYTSNHMWKLFEN	Negative
1314.	P11881:1448	EVEMKEIYTSNHMWKLFENFLVDICRACN	Negative
1315.	P11881:1468	LVDICRACNNTSDRKHADSILEKYVTEIV	Negative
1316.	P11881:1476	NNTSDRKHADSILEKYVTEIVMSIVTTFF	Negative
1317.	P11881:1529	VFRVYHCNWLMPSQKASVESCIRVLSDVA	Negative
1318.	P11881:1544	ASVESCIRVLSDVAKSRAIAIPVDLDSQV	Negative
1319.	P11881:1564	IPVDLDSQVNNLFLKSHNIVQKTALNWRL	Negative
1320.	P11881:1571	QVNNLFLKSHNIVQKTALNWRLSARNAAR	Negative
1321.	P11881:1645	RPELLFPENTDARRKCESGGFICKLIKHT	Negative
1322.	P11881:1654	TDARRKCESGGFICKLIKHTKQLLEENEE	Negative
1323.	P11881:1657	RRKCESGGFICKLIKHTKQLLEENEEKLC	Negative
1324.	P11881:1660	CESGGFICKLIKHTKQLLEENEEKLCIKV	Negative
1325.	P11881:1669	LIKHTKQLLEENEEKLCIKVLQTLREMMT	Negative
1326.	P11881:1673	TKQLLEENEEKLCIKVLQTLREMMTKDRG	Negative
1327.	P11881:1684	LCIKVLQTLREMMTKDRGYGEKQISIDES	Negative
1328.	P11881:1691	TLREMMTKDRGYGEKQISIDESENAELPQ	Negative
1329.	P11881:1730	ELEPSPPLRQLEDHKRGEALRQILVNRYY	Negative
1330.	P11881:1771	TSFGNGPLSPGGPSKPGGGGGGPGSSSTS	Negative
1331.	P11881:1800	RGEMSLAEVQCHLDKEGASNLVIDLIMNA	Negative
1332.	P11881:1850	TTIQHSFFCRLTEDKKSEKFFKVFYDRMK	Negative
1333.	P11881:1851	TIQHSFFCRLTEDKKSEKFFKVFYDRMKV	Negative
1334.	P11881:1854	HSFFCRLTEDKKSEKFFKVFYDRMKVAQQ	Negative
1335.	P11881:1857	FCRLTEDKKSEKFFKVFYDRMKVAQQEIK	Negative
1336.	P11881:1864	KKSEKFFKVFYDRMKVAQQEIKATVTVNT	Negative
1337.	P11881:1871	KVFYDRMKVAQQEIKATVTVNTSDLGNKK	Negative
1338.	P11881:1884	IKATVTVNTSDLGNKKKDDEVDRDAPSRK	Negative
1339.	P11881:1885	KATVTVNTSDLGNKKKDDEVDRDAPSRKK	Negative
1340.	P11881:1886	ATVTVNTSDLGNKKKDDEVDRDAPSRKKA	Negative
1341.	P11881:1898	KKKDDEVDRDAPSRKKAKEPTTQITEEVR	Negative
1342.	P11881:1899	KKDDEVDRDAPSRKKAKEPTTQITEEVRD	Negative
1343.	P11881:1901	DDEVDRDAPSRKKAKEPTTQITEEVRDQL	Negative
1344.	P11881:1924	EVRDQLLEASAATRKAFTTFRREADPDDH	Negative

1345.	P11881:1951	DHYQSGEGTQATTDKAKDDLEMSAVITIM	Negative
1346.	P11881:1953	YQSGEGTQATTDKAKDDLEMSAVITIMQP	Negative
1347.	P11881:1993	HNRDLQNFLRCQNNKTNYNLVCETLQFLD	Negative
1348.	P11881:2027	TTGGLGLLGLYINEKNVALINQTLESLTE	Negative
1349.	P11881:2078	IITALILNDINPLGKKRMDLVLELKNNAS	Negative
1350.	P11881:2079	ITALILNDINPLGKKRMDLVLELKNNASK	Negative
1351.	P11881:2088	NPLGKKRMDLVLELKNNASKLLLAIMESR	Negative
1352.	P11881:2093	KRMDLVLELKNNASKLLLAIMESRHDSEN	Negative
1353.	P11881:2118	DSENAERILYNMRPKELVEVIKKAYMQGE	Negative
1354.	P11881:2125	ILYNMRPKELVEVIKKAYMQGEVEFEDGE	Negative
1355.	P11881:2126	LYNMRPKELVEVIKKAYMQGEVEFEDGEN	Negative
1356.	P11881:2167	HNIYILAHQLARHNKELQTMLKPGGQVDG	Negative
1357.	P11881:2174	HQLARHNKELQTMLKPGGQVDGDEALEFY	Negative
1358.	P11881:2190	GGQVDGDEALEFYAKHTAQIEIVRLDRTM	Negative
1359.	P11881:2220	QIVFPVPSICEFLTKESKLRIYYTTERDE	Negative
1360.	P11881:2223	FPVPSICEFLTKESKLRIYYTTERDEQGS	Negative
1361.	P11881:2238	LRIYYTTERDEQGSKINDFFLRSEDLFNE	Negative
1362.	P11881:2257	FLRSEDLFNEMNWQKKLRAQPVLYWCARN	Negative
1363.	P11881:2258	LRSEDLFNEMNWQKKLRAQPVLYWCARNM	Negative
1364.	P11881:2297	AVLMNLLVAFFYPFKGVRGGTLEPHWSGL	Negative
1365.	P11881:2328	TAMLISLAIVIALPKPHGIRALIASTILR	Negative
1366.	P11881:2364	QPTLFLLGAFNVCNKIIFLMSFVGNCGTF	Negative
1367.	P11881:2431	FDLVYREETLLNVIKSVTRNGRSIILTAV	Negative
1368.	P11881:2463	ILVYLFSIVGYLFFKDDFILEVDRLPNET	Negative
1369.	P11881:2510	CRVETGENCTSPAPKEELLPAEETEQDKE	Negative
1370.	P11881:2523	PKEELLPAEETEQDKEHTCETLLMCIVTV	Negative
1371.	P11881:2554	HGLRSGGGVGDVLRKPSKEEPLFAARVIY	Negative
1372.	P11881:2557	RSGGGVGDVLRKPSKEEPLFAARVIYDLL	Negative
1373.	P11881:2599	FGVIIDTFADLRSEKQKKEEILKTTCFIC	Negative
1374.	P11881:2601	VIIDTFADLRSEKQKKEEILKTTCFICGL	Negative
1375.	P11881:2602	IIDTFADLRSEKQKKEEILKTTCFICGLE	Negative
1376.	P11881:2607	ADLRSEKQKKEEILKTTCFICGLERDKFD	Negative
1377.	P11881:2619	ILKTTCFICGLERDKFDNKTVTFEEHIKE	Negative
1378.	P11881:2623	TCFICGLERDKFDNKTVTFEEHIKEEHNM	Negative
1379.	P11881:2632	DKFDNKTVTFEEHIKEEHNMWHYLCFIVL	Negative
1380.	P11881:2648	EHNMWHYLCFIVLVKVKDSTEYTGPESYV	Negative
1381.	P11881:2650	NMWHYLCFIVLVKVKDSTEYTGPESYVAE	Negative
1382.	P11881:2700	DSEGEQNELRNLQEKLESTMKLVTNLSGQ	Negative
1383.	P11881:2706	NELRNLQEKLESTMKLVTNLSGQLSELKD	Negative
1384.	P11881:2719	MKLVTNLSGQLSELKDQMTEQRKQKQRIG	Negative
1385.	P11881:2727	GQLSELKDQMTEQRKQKQRIGLLGHPPHM	Negative
1386.	P11881:2729	LSELKDQMTEQRKQKQRIGLLGHPPHMNV	Negative
1387.	P11983:33	SQNVMAAASIANIVKSSFGPVGLDKMLVD	Negative
1388.	P11983:43	ANIVKSSFGPVGLDKMLVDDIGDVTITND	Negative
1389.	P11983:63	IGDVTITNDGATILKLLEVEHPAAKVLCE	Negative
1390.	P11983:73	ATILKLLEVEHPAAKVLCELADLQDKEVG	Negative
1391.	P11983:84	PAAKVLCELADLQDKEVGDGTTSVVIIAA	Negative
1392.	P11983:102	DGTTSVVIIAAELLKNADELVKQKIHPTS	Negative
1393.	P11983:109	IIAAELLKNADELVKQKIHPTSVISGYRL	Negative
1394.	P11983:111	AAELLKNADELVKQKIHPTSVISGYRLAC	Negative
1395.	P11983:126	IHPTSVISGYRLACKEAVRYINENLIINT	Negative
1396.	P11983:153	NTDELGRDCLINAAKTSMSSKIIGINGDY	Negative
1397.	P11983:159	RDCLINAAKTSMSSKIIGINGDYFANMVV	Negative
1398.	P11983:180	DYFANMVVDAVLAVKYTDARGQPRYPVNS	Negative

1399.	P11983:199	RGQPRYPVNSVNILKAHGRSQIESMLING	Negative
1400.	P11983:227	GYALNCVVGSQGMPKRIVNAKIACLDFSL	Negative
1401.	P11983:233	VVGSQGMPKRIVNAKIACLDFSLQKTKMK	Negative
1402.	P11983:245	NAKIACLDFSLQKTKMKLGVQVVITDPEK	Negative
1403.	P11983:247	KIACLDFSLQKTKMKLGVQVVITDPEKLD	Negative
1404.	P11983:259	KMKLGVQVVITDPEKLDQIRQRESDITKE	Negative
1405.	P11983:272	EKLDQIRQRESDITKERIQKILATGANVI	Negative
1406.	P11983:277	IRQRESDITKERIQKILATGANVILTTGG	Negative
1407.	P11983:298	NVILTTGGIDDMYLKYFVEAGAMAVRRVL	Negative
1408.	P11983:313	YFVEAGAMAVRRVLKRDLKHVAKASGASI	Negative
1409.	P11983:317	AGAMAVRRVLKRDLKHVAKASGASILSTL	Negative
1410.	P11983:321	AVRRVLKRDLKHVAKASGASILSTLANLE	Negative
1411.	P11983:365	VVQERICDDELILIKNTKARTSASIILRG	Negative
1412.	P11983:368	ERICDDELILIKNTKARTSASIILRGAND	Negative
1413.	P11983:400	DEMERSLHDALCVVKRVLELKSVVPGGGA	Negative
1414.	P11983:406	LHDALCVVKRVLELKSVVPGGGAVEAALS	Negative
1415.	P11983:466	LAVNAAQDSTDLVAKLRAFHNEAQVNPER	Negative
1416.	P11983:481	LRAFHNEAQVNPERKNLKWIGLDLVHGKP	Negative
1417.	P11983:484	FHNEAQVNPERKNLKWIGLDLVHGKPRDN	Negative
1418.	P11983:494	RKNLKWIGLDLVHGKPRDNKQAGVFEPTI	Negative
1419.	P11983:499	WIGLDLVHGKPRDNKQAGVFEPTIVKVKS	Negative
1420.	P11983:510	RDNKQAGVFEPTIVKVKSLKFATEAAITI	Negative
1421.	P11983:512	NKQAGVFEPTIVKVKSLKFATEAAITILR	Negative
1422.	P11983:515	AGVFEPTIVKVKSLKFATEAAITILRIDD	Negative
1423.	P11983:532	TEAAITILRIDDLIKLHPESKDDKHGSYE	Negative
1424.	P11983:538	ILRIDDLIKLHPESKDDKHGSYENAVHSG	Negative
1425.	P11983:541	IDDLIKLHPESKDDKHGSYENAVHSGALD	Negative
1426.	P12710:6	XXXXXXXXXMNFSGKYQLQSQENFEPFMK	Negative
1427.	P12710:20	KYQLQSQENFEPFMKAIGLPEDLIQKGKD	Negative
1428.	P12710:31	PFMKAIGLPEDLIQKGKDIKGVSEIVHEG	Negative
1429.	P12710:33	MKAIGLPEDLIQKGKDIKGVSEIVHEGKK	Negative
1430.	P12710:36	IGLPEDLIQKGKDIKGVSEIVHEGKKIKL	Negative
1431.	P12710:46	GKDIKGVSEIVHEGKKIKLTITYGPKVVR	Negative
1432.	P12710:47	KDIKGVSEIVHEGKKIKLTITYGPKVVRN	Negative
1433.	P12710:49	IKGVSEIVHEGKKIKLTITYGPKVVRNEF	Negative
1434.	P12710:57	HEGKKIKLTITYGPKVVRNEFTLGEECEL	Negative
1435.	P12710:78	TLGEECELETMTGEKVKAVVKLEGDNKMV	Negative
1436.	P12710:80	GEECELETMTGEKVKAVVKLEGDNKMVTT	Negative
1437.	P12710:84	ELETMTGEKVKAVVKLEGDNKMVTTFKGI	Negative
1438.	P12710:90	GEKVKAVVKLEGDNKMVTTFKGIKSVTEL	Negative
1439.	P12710:99	LEGDNKMVTTFKGIKSVTELNGDTITNTM	Negative
1440.	P12710:121	DTITNTMTLGDIVYKRVSKRIXXXXXXXX	Negative
1441.	P12710:125	NTMTLGDIVYKRVSKRIXXXXXXXXXXXX	Negative
1442.	P12790:25	VLLSLFLLLVRGHAKIHGHLPPGPHPLPL	Negative
1443.	P12790:53	LLGNLLQMDRGGLLKCFIQLQEKHGDVFT	Negative
1444.	P12790:61	DRGGLLKCFIQLQEKHGDVFTVHLGPRPV	Negative
1445.	P12790:122	MQDYGIFFASGQRWKTLRRFSLATMKEFG	Negative
1446.	P12790:133	QRWKTLRRFSLATMKEFGMGKRSVEERIK	Negative
1447.	P12790:139	RRFSLATMKEFGMGKRSVEERIKEEAQCL	Negative
1448.	P12790:147	KEFGMGKRSVEERIKEEAQCLVEELKKYQ	Negative
1449.	P12790:158	ERIKEEAQCLVEELKKYQGVPLDPTFLFQ	Negative
1450.	P12790:159	RIKEEAQCLVEELKKYQGVPLDPTFLFQC	Negative
1451.	P12790:204	YTDDQFLHLLNLMYKIFSLLSSFSGQMFE	Negative
1452.	P12790:225	SFSGQMFELFSGFLKYFPGVHRQIVKKQQ	Negative

1453.	P12790:236	GFLKYFPGVHRQIVKKQQELLDYIAHSVE	Negative
1454.	P12790:251	KQQELLDYIAHSVEKHKATLDPSAPRDYI	Negative
1455.	P12790:253	QELLDYIAHSVEKHKATLDPSAPRDYIDT	Negative
1456.	P12790:274	APRDYIDTYLLRMEKEKSNHNTEFHHQNL	Negative
1457.	P12790:276	RDYIDTYLLRMEKEKSNHNTEFHHQNLMM	Negative
1458.	P12790:316	TTSATLHYGVLLMLKYPHVTEKVQKEIDQ	Negative
1459.	P12790:323	YGVLLMLKYPHVTEKVQKEIDQVIGSHRL	Negative
1460.	P12790:326	LLMLKYPHVTEKVQKEIDQVIGSHRLPTL	Negative
1461.	P12790:345	VIGSHRLPTLDDRTKMPYTDAVIHEIQRF	Negative
1462.	P12790:370	IQRFSDLVPIGLPHKVIKDTLFRGYLLPK	Negative
1463.	P12790:373	FSDLVPIGLPHKVIKDTLFRGYLLPKNTE	Negative
1464.	P12790:384	KVIKDTLFRGYLLPKNTEVYPVLSSALHD	Negative
1465.	P12790:407	SSALHDPQYFEQPDKFNPEQFLDANGALK	Negative
1466.	P12790:421	KFNPEQFLDANGALKKCEAFLPFSTGKRI	Negative
1467.	P12790:422	FNPEQFLDANGALKKCEAFLPFSTGKRIC	Negative
1468.	P12790:433	ALKKCEAFLPFSTGKRICLGESIARNELF	Negative
1469.	P12790:466	TILQNFSVASPVAPKDIDLTPKESGIGKI	Negative
1470.	P12790:473	VASPVAPKDIDLTPKESGIGKIPPAHQIY	Negative
1471.	P12790:479	PKDIDLTPKESGIGKIPPAHQIYFLARXX	Negative
1472.	P14174:33	FLSELTQQLAQATGKPPQYIAVHVVPDQL	Negative
1473.	P14174:67	SSEPCALCSLHSIGKIGGAQNRSYSKLLC	Negative
1474.	P16015:3	XXXXXXXXXXXMAKEWGYASHNGPDHWH	Negative
1475.	P16015:24	HNGPDHWHELYPIAKGDNQSPIELHTKDI	Negative
1476.	P16015:36	IAKGDNQSPIELHTKDIKHDPSLQPWSAS	Negative
1477.	P16015:39	GDNQSPIELHTKDIKHDPSLQPWSASYDP	Negative
1478.	P16015:57	SLQPWSASYDPGSAKTILNNGKTCRVVFD	Negative
1479.	P16015:113	SSDDHGSEHTVDGVKYAAELHLVHWNPKY	Negative
1480.	P16015:126	VKYAAELHLVHWNPKYNTFGEALKQPDGI	Negative
1481.	P16015:135	VHWNPKYNTFGEALKQPDGIAVVGIFLKI	Negative
1482.	P16015:148	LKQPDGIAVVGIFLKIGREKGEFQILLDA	Negative
1483.	P16015:153	GIAVVGIFLKIGREKGEFQILLDALDKIK	Negative
1484.	P16015:165	REKGEFQILLDALDKIKTKGKEAPFTHFD	Negative
1485.	P16015:167	KGEFQILLDALDKIKTKGKEAPFTHFDPS	Negative
1486.	P16015:169	EFQILLDALDKIKTKGKEAPFTHFDPSCL	Negative
1487.	P16015:171	QILLDALDKIKTKGKEAPFTHFDPSCLFP	Negative
1488.	P16015:212	TTPPCEECIVWLLLKEPMTVSSDQMAKLR	Negative
1489.	P16015:224	LLKEPMTVSSDQMAKLRSLFSSAENEPPV	Negative
1490.	P16015:251	PVPLVGNWRPPQPVKGRVVRASFKXXXXX	Negative
1491.	P16015:260	PPQPVKGRVVRASFKXXXXXXXXXXXXXX	Negative
1492.	P16879:32	QMQEAELRLLEGMRKWMAQRVKSDREYAG	Negative
1493.	P16879:39	RLLEGMRKWMAQRVKSDREYAGLLHHMSL	Negative
1494.	P16879:101	LRQHAEDLNSGPLSKLSVLIRERQHLRKT	Negative
1495.	P16879:114	SKLSVLIRERQHLRKTYNEQWQQLQQELT	Negative
1496.	P16879:129	TYNEQWQQLQQELTKTHSQDIEKLKTQYR	Negative
1497.	P16879:137	LQQELTKTHSQDIEKLKTQYRTLVRDSTQ	Negative
1498.	P16879:139	QELTKTHSQDIEKLKTQYRTLVRDSTQAR	Negative
1499.	P16879:155	QYRTLVRDSTQARRKYQEASKDKDRDKAK	Negative
1500.	P16879:163	STQARRKYQEASKDKDRDKAKDKYVRSLW	Negative
1501.	P16879:167	RRKYQEASKDKDRDKAKDKYVRSLWKLFA	Negative
1502.	P16879:169	KYQEASKDKDRDKAKDKYVRSLWKLFAHH	Negative
1503.	P16879:171	QEASKDKDRDKAKDKYVRSLWKLFAHHNR	Negative
1504.	P16879:178	DRDKAKDKYVRSLWKLFAHHNRYVLGVRA	Negative
1505.	P16879:224	QSLQDLHEEMAGILKDILQEYLEISSLVQ	Negative

1506.	P16879:322	HTLTSVTDELAVATKEVLSRQEMVSQLQR	Negative
1507.	P16879:356	EQNTHPRERVQLLSKRQMLQEAIQGLQIA	Negative
1508.	P16879:376	EAIQGLQIALCSQDKLQAQQELLQSKMEQ	Negative
1509.	P16879:387	SQDKLQAQQELLQSKMEQLGTGEPPAVPL	Negative
1510.	P16879:428	QEREGGRTPTLEILKSHFSGIFRPKFSIP	Negative
1511.	P16879:438	LEILKSHFSGIFRPKFSIPPPLQLVPEVQ	Negative
1512.	P16879:453	FSIPPPLQLVPEVQKPLYEQLWYHGAIPR	Negative
1513.	P16879:488	LTHSGDFLVRESQGKQEYVLSVMWDGQPR	Negative
1514.	P16879:539	LLITHLLSSQQPLTKKSGVVLFRAVPKDK	Negative
1515.	P16879:540	LITHLLSSQQPLTKKSGVVLFRAVPKDKW	Negative
1516.	P16879:551	LTKKSGVVLFRAVPKDKWVLKHEDLVLGE	Negative
1517.	P16879:553	KKSGVVLFRAVPKDKWVLKHEDLVLGEQI	Negative
1518.	P16879:557	VVLFRAVPKDKWVLKHEDLVLGEQIGRGN	Negative
1519.	P16879:590	FSGRLRADNTPVAVKSCRETLPPDLKAKF	Negative
1520.	P16879:601	VAVKSCRETLPPDLKAKFLQEARILKQYN	Negative
1521.	P16879:603	VKSCRETLPPDLKAKFLQEARILKQYNHP	Negative
1522.	P16879:612	PDLKAKFLQEARILKQYNHPNIVRLIGVC	Negative
1523.	P16879:629	NHPNIVRLIGVCTQKQPIYIVMELVQGGD	Negative
1524.	P16879:658	FLTFLRTEGARLRVKTLLQMVGDAAAGME	Negative
1525.	P16879:677	MVGDAAAGMEYLESKCCIHRDLAARNCLV	Negative
1526.	P16879:694	IHRDLAARNCLVTEKNVLKISDFGMSREE	Negative
1527.	P16879:698	LAARNCLVTEKNVLKISDFGMSREEADGI	Negative
1528.	P16879:725	GIYAASAGLRQVPVKWTAPEALNYGRYSS	Negative
1529.	P16879:775	YPNLTNQQTREFVEKGHRLPCPELCPDAV	Negative
1530.	P16879:819	PSFSIICQELHSIRKRHRXXXXXXXXXXX	Negative
1531.	P18894:32	CIHERYHPTQPLHMKIYADRFTPFTTSDV	Negative
1532.	P18894:85	FDYLLSCLHSPNAEKMGLALISGYNLFRD	Negative
1533.	P18894:107	GYNLFRDEVPDPFWKNAVLGFRKLTPSEM	Negative
1534.	P18894:115	VPDPFWKNAVLGFRKLTPSEMDLFPDYGY	Negative
1535.	P18894:141	YGYGWFNTSLLLEGKSYLPWLTERLTERG	Negative
1536.	P18894:157	YLPWLTERLTERGVKLIHRKVESLEEVAR	Negative
1537.	P18894:162	TERLTERGVKLIHRKVESLEEVARGVDVI	Negative
1538.	P18894:209	PGRGQIIQVEAPWIKHFILTHDPSLGIYN	Negative
1539.	P18894:232	SLGIYNSPYIIPGSKTVTLGGIFQLGNWS	Negative
1540.	P18894:269	NTIWKSCCKLEPTLKNARIVGELTGFRPV	Negative
1541.	P18894:330	IHWGCAMEAANLFGKILEEKKLSRLPPSH	Negative
1542.	P18894:335	AMEAANLFGKILEEKKLSRLPPSHLXXXX	Negative
1543.	P18894:336	MEAANLFGKILEEKKLSRLPPSHLXXXXX	Negative
1544.	P22752:6	XXXXXXXXXMSGRGKQGGKARAKAKTRSS	Negative
1545.	P22752:10	XXXXXMSGRGKQGGKARAKAKTRSSRAGL	Negative
1546.	P22752:14	XMSGRGKQGGKARAKAKTRSSRAGLQFPV	Negative
1547.	P22752:16	SGRGKQGGKARAKAKTRSSRAGLQFPVGR	Negative
1548.	P22752:37	GLQFPVGRVHRLLRKGNYSERVGAGAPVY	Negative
1549.	P22752:75	AEILELAGNAARDNKKTRIIPRHLQLAIR	Negative
1550.	P22752:76	EILELAGNAARDNKKTRIIPRHLQLAIRN	Negative
1551.	P22752:119	QGGVLPNIQAVLLPKKTESHHKAKGKXXX	Negative
1552.	P22752:120	GGVLPNIQAVLLPKKTESHHKAKGKXXXX	Negative
1553.	P22752:126	IQAVLLPKKTESHHKAKGKXXXXXXXXXX	Negative
1554.	P22752:128	AVLLPKKTESHHKAKGKXXXXXXXXXXXX	Negative
1555.	P22752:130	LLPKKTESHHKAKGKXXXXXXXXXXXXXX	Negative
1556.	P23881:12	XXXMGLEEELLRIAKKLEKMVSRKKTEGA	Negative
1557.	P23881:13	XXMGLEEELLRIAKKLEKMVSRKKTEGAL	Negative
1558.	P23881:16	GLEEELLRIAKKLEKMVSRKKTEGALDLL	Negative
1559.	P23881:21	LLRIAKKLEKMVSRKKTEGALDLLKKLNS	Negative

1560.	P23881:22	LRIAKKLEKMVSRKKTEGALDLLKKLNSC	Negative
1561.	P23881:31	MVSRKKTEGALDLLKKLNSCQMSIQLLQT	Negative
1562.	P23881:32	VSRKKTEGALDLLKKLNSCQMSIQLLQTT	Negative
1563.	P23881:57	LQTTRIGVAVNGVRKHCSDKEVVSLAKVL	Negative
1564.	P23881:62	IGVAVNGVRKHCSDKEVVSLAKVLIKNWK	Negative
1565.	P23881:69	VRKHCSDKEVVSLAKVLIKNWKRLLDSPR	Negative
1566.	P23881:73	CSDKEVVSLAKVLIKNWKRLLDSPRTTKG	Negative
1567.	P23881:76	KEVVSLAKVLIKNWKRLLDSPRTTKGERE	Negative
1568.	P23881:86	IKNWKRLLDSPRTTKGEREEREKAKKEKG	Negative
1569.	P23881:94	DSPRTTKGEREEREKAKKEKGLGCSDWKP	Negative
1570.	P23881:96	PRTTKGEREEREKAKKEKGLGCSDWKPEA	Negative
1571.	P23881:97	RTTKGEREEREKAKKEKGLGCSDWKPEAG	Negative
1572.	P23881:99	TKGEREEREKAKKEKGLGCSDWKPEAGLS	Negative
1573.	P23881:107	EKAKKEKGLGCSDWKPEAGLSPPRKKGGG	Negative
1574.	P23881:117	CSDWKPEAGLSPPRKKGGGEPKTRRDSVD	Negative
1575.	P23881:118	SDWKPEAGLSPPRKKGGGEPKTRRDSVDS	Negative
1576.	P23881:124	AGLSPPRKKGGGEPKTRRDSVDSRSSTTS	Negative
1577.	P23881:141	RDSVDSRSSTTSSPKRPSLERSNSSKSKV	Negative
1578.	P23881:152	SSPKRPSLERSNSSKSKVETPTTPSSPST	Negative
1579.	P23881:154	PKRPSLERSNSSKSKVETPTTPSSPSTPT	Negative
1580.	P23881:189	LLAPCYLTGDSVRDKCVEMLSAALKAEDN	Negative
1581.	P23881:205	VEMLSAALKAEDNFKDYGVNCDKLASEIE	Negative
1582.	P23881:213	KAEDNFKDYGVNCDKLASEIEDHIYQELK	Negative
1583.	P23881:227	KLASEIEDHIYQELKSTDMKYRNRVRSRI	Negative
1584.	P23881:232	IEDHIYQELKSTDMKYRNRVRSRISNLKD	Negative
1585.	P23881:245	MKYRNRVRSRISNLKDPRNPGLRRNVLSG	Negative
1586.	P23881:268	RNVLSGAISPELIAKMTAEEMASDELREL	Negative
1587.	P23881:298	NAMTQEAIREHQMAKTGGTTTDLLRCSKC	Negative
1588.	P23881:311	AKTGGTTTDLLRCSKCKKKNCTYNQVQTR	Negative
1589.	P23881:313	TGGTTTDLLRCSKCKKKNCTYNQVQTRSA	Negative
1590.	P23881:314	GGTTTDLLRCSKCKKKNCTYNQVQTRSAD	Negative
1591.	P23881:315	GTTTDLLRCSKCKKKNCTYNQVQTRSADE	Negative
1592.	P23881:345	MTTFVLCNECGNRWKFCXXXXXXXXXXXX	Negative
1593.	P26040:3	XXXXXXXXXXXXMPKPINVRVTTMDAELE	Negative
1594.	P26040:27	DAELEFAIQPNTTGKQLFDQVVKTIGLRE	Negative
1595.	P26040:35	QPNTTGKQLFDQVVKTIGLREVWYFGLQY	Negative
1596.	P26040:53	LREVWYFGLQYVDNKGFPTWLKLDKKVSA	Negative
1597.	P26040:63	YVDNKGFPTWLKLDKKVSAQEVRKENPVQ	Negative
1598.	P26040:64	VDNKGFPTWLKLDKKVSAQEVRKENPVQF	Negative
1599.	P26040:72	WLKLDKKVSAQEVRKENPVQFKFRAKFYP	Negative
1600.	P26040:79	VSAQEVRKENPVQFKFRAKFYPEDVAEEL	Negative
1601.	P26040:83	EVRKENPVQFKFRAKFYPEDVAEELIQDI	Negative
1602.	P26040:100	PEDVAEELIQDITQKLFFLQVKDGILSDE	Negative
1603.	P26040:107	LIQDITQKLFFLQVKDGILSDEIYCPPET	Negative
1604.	P26040:133	PETAVLLGSYAVQAKFGDYNKEMHKSGYL	Negative
1605.	P26040:139	LGSYAVQAKFGDYNKEMHKSGYLSSERLI	Negative
1606.	P26040:143	AVQAKFGDYNKEMHKSGYLSSERLIPQRV	Negative
1607.	P26040:162	SSERLIPQRVMDQHKLSRDQWEDRIQVWH	Negative
1608.	P26040:184	DRIQVWHAEHRGMLKDSAMLEYLKIAQDL	Negative
1609.	P26040:193	HRGMLKDSAMLEYLKIAQDLEMYGINYFE	Negative
1610.	P26040:209	AQDLEMYGINYFEIKNKKGTDLWLGVDAL	Negative
1611.	P26040:211	DLEMYGINYFEIKNKKGTDLWLGVDALGL	Negative
1612.	P26040:212	LEMYGINYFEIKNKKGTDLWLGVDALGLN	Negative
1613.	P26040:230	LWLGVDALGLNIYEKDDKLTPKIGFPWSE	Negative

1614.	P26040:233	GVDALGLNIYEKDDKLTPKIGFPWSEIRN	Negative
1615.	P26040:237	LGLNIYEKDDKLTPKIGFPWSEIRNISFN	Negative
1616.	P26040:253	GFPWSEIRNISFNDKKFVIKPIDKKAPDF	Negative
1617.	P26040:254	FPWSEIRNISFNDKKFVIKPIDKKAPDFV	Negative
1618.	P26040:258	EIRNISFNDKKFVIKPIDKKAPDFVFYAP	Negative
1619.	P26040:262	ISFNDKKFVIKPIDKKAPDFVFYAPRLRI	Negative
1620.	P26040:263	SFNDKKFVIKPIDKKAPDFVFYAPRLRIN	Negative
1621.	P26040:278	APDFVFYAPRLRINKRILQLCMGNHELYM	Negative
1622.	P26040:296	QLCMGNHELYMRRRKPDTIEVQQMKAQAR	Negative
1623.	P26040:306	MRRRKPDTIEVQQMKAQAREEKHQKQLER	Negative
1624.	P26040:313	TIEVQQMKAQAREEKHQKQLERQQLETEK	Negative
1625.	P26040:316	VQQMKAQAREEKHQKQLERQQLETEKKRR	Negative
1626.	P26040:327	KHQKQLERQQLETEKKRRETVEREKEQML	Negative
1627.	P26040:328	HQKQLERQQLETEKKRRETVEREKEQMLR	Negative
1628.	P26040:337	LETEKKRRETVEREKEQMLREKEELMLRL	Negative
1629.	P26040:344	RETVEREKEQMLREKEELMLRLQDYEQKT	Negative
1630.	P26040:357	EKEELMLRLQDYEQKTKRAEKELSEQIEK	Negative
1631.	P26040:359	EELMLRLQDYEQKTKRAEKELSEQIEKAL	Negative
1632.	P26040:363	LRLQDYEQKTKRAEKELSEQIEKALQLEE	Negative
1633.	P26040:371	KTKRAEKELSEQIEKALQLEEERRRAQEE	Negative
1634.	P26040:400	AERLEADRMAALRAKEELERQAQDQIKSQ	Negative
1635.	P26040:412	RAKEELERQAQDQIKSQEQLAAELAEYTA	Negative
1636.	P26040:427	SQEQLAAELAEYTAKIALLEEARRRKEDE	Negative
1637.	P26040:438	YTAKIALLEEARRRKEDEVEEWQHRAKEA	Negative
1638.	P26040:450	RRKEDEVEEWQHRAKEAQDDLVKTKEELH	Negative
1639.	P26040:458	EWQHRAKEAQDDLVKTKEELHLVMTAPPP	Negative
1640.	P26040:460	QHRAKEAQDDLVKTKEELHLVMTAPPPPP	Negative
1641.	P26040:516	ELSSEGILDDRNEEKRITEAEKNERVQRQ	Negative
1642.	P26040:523	LDDRNEEKRITEAEKNERVQRQLLTLSNE	Negative
1643.	P26040:546	LTLSNELSQARDENKRTHNDIIHNENMRQ	Negative
1644.	P26040:564	NDIIHNENMRQGRDKYKTLRQIRQGNTKQ	Negative
1645.	P26040:566	IIHNENMRQGRDKYKTLRQIRQGNTKQRI	Negative
1646.	P26040:577	DKYKTLRQIRQGNTKQRIDEFEAMXXXXX	Negative
1647.	P26443:68	SEAAADREDDPNFFKMVEGFFDRGASIVE	Negative
1648.	P26443:84	VEGFFDRGASIVEDKLVEDLKTRESEEQK	Negative
1649.	P26443:90	RGASIVEDKLVEDLKTRESEEQKRNRVRG	Negative
1650.	P26443:98	KLVEDLKTRESEEQKRNRVRGILRIIKPC	Negative
1651.	P26443:110	EQKRNRVRGILRIIKPCNHVLSLSFPIRR	Negative
1652.	P26443:147	EGYRAQHSQHRTPCKGGIRYSTDVSVDEV	Negative
1653.	P26443:162	GGIRYSTDVSVDEVKALASLMTYKCAVVD	Negative
1654.	P26443:171	SVDEVKALASLMTYKCAVVDVPFGGAKAG	Negative
1655.	P26443:183	TYKCAVVDVPFGGAKAGVKINPKNYTDNE	Negative
1656.	P26443:187	AVVDVPFGGAKAGVKINPKNYTDNELEKI	Negative
1657.	P26443:191	VPFGGAKAGVKINPKNYTDNELEKITRRF	Negative
1658.	P26443:200	VKINPKNYTDNELEKITRRFTMELAKKGF	Negative
1659.	P26443:211	ELEKITRRFTMELAKKGFIGPGIDVPAPD	Negative
1660.	P26443:212	LEKITRRFTMELAKKGFIGPGIDVPAPDM	Negative
1661.	P26443:258	IGHYDINAHACVTGKPISQGGIHGRISAT	Negative
1662.	P26443:302	SYMSILGMTPGFGDKTFVVQGFGNVGLHS	Negative
1663.	P26443:326	VGLHSMRYLHRFGAKCVGVGESDGSIWNP	Negative
1664.	P26443:346	ESDGSIWNPDGIDPKELEDFKLQHGSILG	Negative
1665.	P26443:352	WNPDGIDPKELEDFKLQHGSILGFPKAKV	Negative
1666.	P26443:363	EDFKLQHGSILGFPKAKVYEGSILEADCD	Negative
1667.	P26443:365	FKLQHGSILGFPKAKVYEGSILEADCDIL	Negative

1668.	P26443:386	LEADCDILIPAASEKQLTKSNAPRVKAKI	Negative
1669.	P26443:390	CDILIPAASEKQLTKSNAPRVKAKIIAEG	Negative
1670.	P26443:397	ASEKQLTKSNAPRVKAKIIAEGANGPTTP	Negative
1671.	P26443:399	EKQLTKSNAPRVKAKIIAEGANGPTTPEA	Negative
1672.	P26443:415	IAEGANGPTTPEADKIFLERNIMVIPDLY	Negative
1673.	P26443:444	LNAGGVTVSYFEWLKNLNHVSYGRLTFKY	Negative
1674.	P26443:457	LKNLNHVSYGRLTFKYERDSNYHLLMSVQ	Negative
1675.	P26443:477	NYHLLMSVQESLERKFGKHGGTIPVVPTA	Negative
1676.	P26443:480	LLMSVQESLERKFGKHGGTIPVVPTAEFQ	Negative
1677.	P26443:527	TMERSARQIMRTAMKYNLGLDLRTAAYVN	Negative
1678.	P26443:545	GLDLRTAAYVNAIEKVFKVYNEAGVTFTX	Negative
1679.	P26443:548	LRTAAYVNAIEKVFKVYNEAGVTFTXXXX	Negative
1680.	P35585:12	XXXMSASAVYVLDLKGKVLICRNYRGDVD	Negative
1681.	P35585:14	XMSASAVYVLDLKGKVLICRNYRGDVDMS	Negative
1682.	P35585:40	DMSEVEHFMPILMEKEEEGMLSPILAHGG	Negative
1683.	P35585:61	SPILAHGGVRFMWIKHNNLYLVATSKKNA	Negative
1684.	P35585:72	MWIKHNNLYLVATSKKNACVSLVFSFLYK	Negative
1685.	P35585:73	WIKHNNLYLVATSKKNACVSLVFSFLYKV	Negative
1686.	P35585:86	KKNACVSLVFSFLYKVVQVFSEYFKELEE	Negative
1687.	P35585:96	SFLYKVVQVFSEYFKELEEESIRDNFVII	Negative
1688.	P35585:129	DELMDFGYPQTTDSKILQEYITQEGHKLE	Negative
1689.	P35585:141	DSKILQEYITQEGHKLETGAPRPPATVTN	Negative
1690.	P35585:165	ATVTNAVSWRSEGIKYRKNEVFLDVIEAV	Negative
1691.	P35585:168	TNAVSWRSEGIKYRKNEVFLDVIEAVNLL	Negative
1692.	P35585:199	ANGNVLRSEIVGSIKMRVFLSGMPELRLG	Negative
1693.	P35585:217	FLSGMPELRLGLNDKVLFDNTGRGKSKSV	Negative
1694.	P35585:227	GLNDKVLFDNTGRGKSKSVELEDVKFHQC	Negative
1695.	P35585:229	NDKVLFDNTGRGKSKSVELEDVKFHQCVR	Negative
1696.	P35585:237	TGRGKSKSVELEDVKFHQCVRLSRFENDR	Negative
1697.	P35585:274	GEFELMSYRLNTHVKPLIWIESVIEKHSH	Negative
1698.	P35585:285	THVKPLIWIESVIEKHSHSRIEYMVKAKS	Negative
1699.	P35585:298	EKHSHSRIEYMVKAKSQFKRRSTANNVEI	Negative
1700.	P35585:302	HSRIEYMVKAKSQFKRRSTANNVEIHIPV	Negative
1701.	P35585:324	VEIHIPVPNDADSPKFKTTVGSVKWVPEN	Negative
1702.	P35585:326	IHIPVPNDADSPKFKTTVGSVKWVPENSE	Negative
1703.	P35585:333	DADSPKFKTTVGSVKWVPENSEIVWSVKS	Negative
1704.	P35585:346	VKWVPENSEIVWSVKSFPGGKEYLMRAHF	Negative
1705.	P35585:352	NSEIVWSVKSFPGGKEYLMRAHFGLPSVE	Negative
1706.	P35585:370	MRAHFGLPSVEAEDKEGKPPISVKFEIPY	Negative
1707.	P35585:373	HFGLPSVEAEDKEGKPPISVKFEIPYFTT	Negative
1708.	P35585:379	VEAEDKEGKPPISVKFEIPYFTTSGIQVR	Negative
1709.	P35585:396	IPYFTTSGIQVRYLKIIEKSGYQALPWVR	Negative
1710.	P35585:400	TTSGIQVRYLKIIEKSGYQALPWVRYITQ	Negative
1711.	P47806:82	FPPPPPPRSSVKLTKKRALSISPLSDASL	Negative
1712.	P47806:83	PPPPPPRSSVKLTKKRALSISPLSDASLD	Negative
1713.	P47806:146	MSPSLGFPPQMSHQKGTSPPYGVQPCVPH	Negative
1714.	P47806:183	LHPQSRGPRATCQLKSELDMMVGKCPEDP	Negative
1715.	P47806:192	ATCQLKSELDMMVGKCPEDPLEGDMSSPN	Negative
1716.	P47806:229	LGMLDGREDLEREEKPEPESVYETDCRWD	Negative
1717.	P47806:269	LVHHINSEHIHGERKEFVCHWGGCSRELR	Negative
1718.	P47806:286	VCHWGGCSRELRPFKAQYMLVVHMRRHTG	Negative
1719.	P47806:302	QYMLVVHMRRHTGEKPHKCTFEGCRKSYS	Negative
1720.	P47806:305	LVVHMRRHTGEKPHKCTFEGCRKSYSRLE	Negative
1721.	P47806:313	TGEKPHKCTFEGCRKSYSRLENLKTHLRS	Negative

1722.	P47806:322	FEGCRKSYSRLENLKTHLRSHTGEKPYMC	Negative
1723.	P47806:332	LENLKTHLRSHTGEKPYMCEQEGCSKAFS	Negative
1724.	P47806:343	TGEKPYMCEQEGCSKAFSNASDRAKHQNR	Negative
1725.	P47806:353	EGCSKAFSNASDRAKHQNRTHSNEKPYVC	Negative
1726.	P47806:363	SDRAKHQNRTHSNEKPYVCKLPGCTKRYT	Negative
1727.	P47806:368	HQNRTHSNEKPYVCKLPGCTKRYTDPSSL	Negative
1728.	P47806:374	SNEKPYVCKLPGCTKRYTDPSSLRKHVKT	Negative
1729.	P47806:384	PGCTKRYTDPSSLRKHVKTVHGPDAHVTK	Negative
1730.	P47806:387	TKRYTDPSSLRKHVKTVHGPDAHVTKRHR	Negative
1731.	P47806:398	KHVKTVHGPDAHVTKRHRGDGPLPRAQPL	Negative
1732.	P47806:418	GPLPRAQPLSTVEPKREREGGSGREESRL	Negative
1733.	P47806:520	DQLHQLRPIGSRGLKLPSLTHAGAPVSRR	Negative
1734.	P47806:660	RAADHPAPARVQRFKSLGCVHTPPSVATG	Negative
1735.	P47806:795	WGEFPSHAGVYPSNKAPGAAYSQCPRLEH	Negative
1736.	P47806:816	SQCPRLEHYGQVQVKPEQGCPVGSDSTGL	Negative
1737.	P47806:896	LNFNPSSSHSTGQLKAQLVCNYVQSQQEL	Negative
1738.	P47806:931	RGGLPNQELPYQSPKFLGGSQVSQSPAKT	Negative
1739.	P47806:944	PKFLGGSQVSQSPAKTPAAAAAAYGSGFA	Negative
1740.	P47806:965	AAYGSGFAPASANHKSGSYPAPSPCHETF	Negative
1741.	P47806:1008	PRLLPPLSPCYGPLKVGDTNPSCGHPEVG	Negative
1742.	P47806:1077	GLSPPLSHEQGDSSKNTPSPSGPPNMAVG	Negative
1743.	P47857:10	XXXXXMTHEEHHAAKTLGIGKAIAVLTSG	Negative
1744.	P47857:16	THEEHHAAKTLGIGKAIAVLTSGGDAQGM	Negative
1745.	P47857:90	MLQLGGTVIGSARCKDFREREGRLRAAHN	Negative
1746.	P47857:107	REREGRLRAAHNLVKRGITNLCVIGGDGS	Negative
1747.	P47857:141	TFRSEWSDLLNDLQKDGKITAEEATKSSY	Negative
1748.	P47857:144	SEWSDLLNDLQKDGKITAEEATKSSYLNI	Negative
1749.	P47857:152	DLQKDGKITAEEATKSSYLNIVGLVGSID	Negative
1750.	P47857:269	SRLNIIIVAEGAIDKNGKPITSEDIKNLV	Negative
1751.	P47857:272	NIIIVAEGAIDKNGKPITSEDIKNLVVKR	Negative
1752.	P47857:280	AIDKNGKPITSEDIKNLVVKRLGYDTRVT	Negative
1753.	P47857:285	GKPITSEDIKNLVVKRLGYDTRVTVLGHV	Negative
1754.	P47857:356	QAVRLPLMECVQVTKDVTKAMDEKRFDEA	Negative
1755.	P47857:360	LPLMECVQVTKDVTKAMDEKRFDEAIKLR	Negative
1756.	P47857:365	CVQVTKDVTKAMDEKRFDEAIKLRGRSFM	Negative
1757.	P47857:372	VTKAMDEKRFDEAIKLRGRSFMNNWEVYK	Negative
1758.	P47857:386	KLRGRSFMNNWEVYKLLAHVRPPVSKGGL	Negative
1759.	P47857:397	EVYKLLAHVRPPVSKGGLHTVAVMNVGAP	Negative
1760.	P47857:445	NRVLVVHDGFEGLAKGQIEEAGWSYVGGW	Negative
1761.	P47857:466	GWSYVGGWTGQGGSKLGTKRTLPKKNLEQ	Negative
1762.	P47857:470	VGGWTGQGGSKLGTKRTLPKKNLEQISAN	Negative
1763.	P47857:475	GQGGSKLGTKRTLPKKNLEQISANITKFN	Negative
1764.	P47857:476	QGGSKLGTKRTLPKKNLEQISANITKFNI	Negative
1765.	P47857:487	LPKKNLEQISANITKFNIQGLVIIGGFEA	Negative
1766.	P47857:513	FEAYTGGLELMEGRKQFDELCIPFVVIPA	Negative
1767.	P47857:557	DTALNTICTTCDRIKQSAAGTKRRVFIIE	Negative
1768.	P47857:564	CTTCDRIKQSAAGTKRRVFIIETMGGYCG	Negative
1769.	P47857:615	TIRDLQVNVEHLVQKMKTTVKRGLVLRNE	Negative
1770.	P47857:617	RDLQVNVEHLVQKMKTTVKRGLVLRNEKC	Negative
1771.	P47857:621	VNVEHLVQKMKTTVKRGLVLRNEKCNENY	Negative
1772.	P47857:630	MKTTVKRGLVLRNEKCNENYTTDFIFNLY	Negative
1773.	P47857:649	YTTDFIFNLYSEEGKGIFDSRKNVLGHMQ	Negative
1774.	P47857:656	NLYSEEGKGIFDSRKNVLGHMQQGGSPTP	Negative
1775.	P47857:682	PTPFDRNFATKMGAKAMNWMSGKIKESYR	Negative

1776.	P47857:690	ATKMGAKAMNWMSGKIKESYRNGRIFANT	Negative
1777.	P47857:692	KMGAKAMNWMSGKIKESYRNGRIFANTPD	Negative
1778.	P47857:715	FANTPDSGCVLGMRKRALVFQPVTELKDQ	Negative
1779.	P47857:727	MRKRALVFQPVTELKDQTDFEHRIPKEQW	Negative
1780.	P47857:738	TELKDQTDFEHRIPKEQWWLKLRPILKIL	Negative
1781.	P47857:744	TDFEHRIPKEQWWLKLRPILKILAKYEID	Negative
1782.	P47857:750	IPKEQWWLKLRPILKILAKYEIDLDTSDH	Negative
1783.	P47857:754	QWWLKLRPILKILAKYEIDLDTSDHAHLE	Negative
1784.	P47857:773	LDTSDHAHLEHISRKRSGEAAVXXXXXXX	Negative
1785.	P48962:10	XXXXXMGDQALSFLKDFLAGGIAAAVSKT	Negative
1786.	P48962:23	LKDFLAGGIAAAVSKTAVAPIERVKLLLQ	Negative
1787.	P48962:33	AAVSKTAVAPIERVKLLLQVQHASKQISA	Negative
1788.	P48962:43	IERVKLLLQVQHASKQISAEKQYKGIIDC	Negative
1789.	P48962:49	LLQVQHASKQISAEKQYKGIIDCVVRIPK	Negative
1790.	P48962:52	VQHASKQISAEKQYKGIIDCVVRIPKEQG	Negative
1791.	P48962:63	KQYKGIIDCVVRIPKEQGFLSFWRGNLAN	Negative
1792.	P48962:94	RYFPTQALNFAFKDKYKQIFLGGVDRHKQ	Negative
1793.	P48962:107	DKYKQIFLGGVDRHKQFWRYFAGNLASGG	Negative
1794.	P48962:147	PLDFARTRLAADVGKGSSQREFNGLGDCL	Negative
1795.	P48962:163	SSQREFNGLGDCLTKIFKSDGLKGLYQGF	Negative
1796.	P48962:166	REFNGLGDCLTKIFKSDGLKGLYQGFSVS	Negative
1797.	P48962:171	LGDCLTKIFKSDGLKGLYQGFSVSVQGII	Negative
1798.	P48962:199	IIYRAAYFGVYDTAKGMLPDPKNVHIIVS	Negative
1799.	P48962:206	FGVYDTAKGMLPDPKNVHIIVSWMIAQSV	Negative
1800.	P48962:245	FDTVRRRMMMQSGRKGADIMYTGTLDCWR	Negative
1801.	P48962:260	GADIMYTGTLDCWRKIAKDEGANAFFKGA	Negative
1802.	P48962:263	IMYTGTLDCWRKIAKDEGANAFFKGAWSN	Negative
1803.	P48962:272	WRKIAKDEGANAFFKGAWSNVLRGMGGAF	Negative
1804.	P48962:295	GMGGAFVLVLYDEIKKYVXXXXXXXXXXX	Negative
1805.	P48962:296	MGGAFVLVLYDEIKKYVXXXXXXXXXXXX	Negative
1806.	P49006:7	XXXXXXXXMGSQSSKAPRGDVTAEEAAGA	Negative
1807.	P49006:25	GDVTAEEAAGASPAKANGQENGHVKSNGD	Negative
1808.	P49006:35	ASPAKANGQENGHVKSNGDLSPKGEGESP	Negative
1809.	P49006:43	QENGHVKSNGDLSPKGEGESPPVNGTDEA	Negative
1810.	P49006:77	DAIEPAPPSQGAEAKGEVPPKETPKKKKK	Negative
1811.	P49006:83	PPSQGAEAKGEVPPKETPKKKKKFSFKKP	Negative
1812.	P49006:87	GAEAKGEVPPKETPKKKKKFSFKKPFKLS	Negative
1813.	P49006:88	AEAKGEVPPKETPKKKKKFSFKKPFKLSG	Negative
1814.	P49006:89	EAKGEVPPKETPKKKKKFSFKKPFKLSGL	Negative
1815.	P49006:90	AKGEVPPKETPKKKKKFSFKKPFKLSGLS	Negative
1816.	P49006:91	KGEVPPKETPKKKKKFSFKKPFKLSGLSF	Negative
1817.	P49006:95	PPKETPKKKKKFSFKKPFKLSGLSFKRNR	Negative
1818.	P49006:96	PKETPKKKKKFSFKKPFKLSGLSFKRNRK	Negative
1819.	P49006:99	TPKKKKKFSFKKPFKLSGLSFKRNRKEGG	Negative
1820.	P49006:106	FSFKKPFKLSGLSFKRNRKEGGGDSSASS	Negative
1821.	P49006:110	KPFKLSGLSFKRNRKEGGGDSSASSPTEE	Negative
1822.	P49006:157	GKAAATPESQEPQAKGAEASAASEEEAGP	Negative
1823.	P49722:18	RGYSFSLTTFSPSGKLVQIEYALAAVAGG	Negative
1824.	P49722:39	ALAAVAGGAPSVGIKAANGVVLATEKKQK	Negative
1825.	P49722:50	VGIKAANGVVLATEKKQKSILYDERSVHK	Negative
1826.	P49722:51	GIKAANGVVLATEKKQKSILYDERSVHKV	Negative
1827.	P49722:53	KAANGVVLATEKKQKSILYDERSVHKVEP	Negative
1828.	P49722:64	KKQKSILYDERSVHKVEPITKHIGLVYSG	Negative

1829.	P49722:70	LYDERSVHKVEPITKHIGLVYSGMGPDYR	Negative
1830.	P49722:159	YLFQSDPSGAYFAWKATAMGKNYVNGKTF	Negative
1831.	P49722:165	PSGAYFAWKATAMGKNYVNGKTFLEKRYN	Negative
1832.	P49722:171	AWKATAMGKNYVNGKTFLEKRYNEDLELE	Negative
1833.	P49722:176	AMGKNYVNGKTFLEKRYNEDLELEDAIHT	Negative
1834.	P49722:196	LELEDAIHTAILTLKESFEGQMTEDNIEV	Negative
1835.	P51814:39	VRRERTPEARIHSVKRYPDLSPGPKGRSS	Negative
1836.	P51814:49	IHSVKRYPDLSPGPKGRSSADHAALNSIV	Negative
1837.	P51814:80	QASVSFEDVTVDFSKEEWQHLDPAQRRLY	Negative
1838.	P51814:114	ENYSHLLSVGYQIPKSEAAFKLEQGEGPW	Negative
1839.	P51814:120	LSVGYQIPKSEAAFKLEQGEGPWMLEGEA	Negative
1840.	P51814:146	GEAPHQSCSGEAIGKMQQQGIPGGIFFHC	Negative
1841.	P51814:201	EQRQENQNNLLSHVKVLIKERGYEHKNIE	Negative
1842.	P51814:205	ENQNNLLSHVKVLIKERGYEHKNIEKIIH	Negative
1843.	P51814:212	SHVKVLIKERGYEHKNIEKIIHVTTKLVP	Negative
1844.	P51814:216	VLIKERGYEHKNIEKIIHVTTKLVPSIKR	Negative
1845.	P51814:223	YEHKNIEKIIHVTTKLVPSIKRLHNCDTI	Negative
1846.	P51814:229	EKIIHVTTKLVPSIKRLHNCDTILKHTLN	Negative
1847.	P51814:239	VPSIKRLHNCDTILKHTLNSHNHNRNSAT	Negative
1848.	P51814:254	HTLNSHNHNRNSATKNLGKIFGNGNNFPH	Negative
1849.	P51814:258	SHNHNRNSATKNLGKIFGNGNNFPHSPSS	Negative
1850.	P51814:274	FGNGNNFPHSPSSTKNENAKTGANSCEHD	Negative
1851.	P51814:279	NFPHSPSSTKNENAKTGANSCEHDHYEKH	Negative
1852.	P51814:292	AKTGANSCEHDHYEKHLSHKQAPTHHQKI	Negative
1853.	P51814:305	EKHLSHKQAPTHHQKIHPEEKLYVCTECV	Negative
1854.	P51814:311	KQAPTHHQKIHPEEKLYVCTECVMGFTQK	Negative
1855.	P51814:325	KLYVCTECVMGFTQKSHLFEHQRIHAGEK	Negative
1856.	P51814:339	KSHLFEHQRIHAGEKSRECDKSNKVFPQK	Negative
1857.	P51814:345	HQRIHAGEKSRECDKSNKVFPQKPQVDVH	Negative
1858.	P51814:348	IHAGEKSRECDKSNKVFPQKPQVDVHPSV	Negative
1859.	P51814:353	KSRECDKSNKVFPQKPQVDVHPSVYTGEK	Negative
1860.	P51814:367	KPQVDVHPSVYTGEKPYLCTQCGKVFTLK	Negative
1861.	P51814:376	VYTGEKPYLCTQCGKVFTLKSNLITHQKI	Negative
1862.	P51814:381	KPYLCTQCGKVFTLKSNLITHQKIHTGQK	Negative
1863.	P51814:389	GKVFTLKSNLITHQKIHTGQKPYKCSECG	Negative
1864.	P51814:395	KSNLITHQKIHTGQKPYKCSECGKAFFQR	Negative
1865.	P51814:398	LITHQKIHTGQKPYKCSECGKAFFQRSDL	Negative
1866.	P51814:404	IHTGQKPYKCSECGKAFFQRSDLFRHLRI	Negative
1867.	P51814:423	RSDLFRHLRIHTGEKPYECSECGKGFSQN	Negative
1868.	P51814:432	IHTGEKPYECSECGKGFSQNSDLSIHQKT	Negative
1869.	P51814:445	GKGFSQNSDLSIHQKTHTGEKHYECNECG	Negative
1870.	P51814:451	NSDLSIHQKTHTGEKHYECNECGKAFTRK	Negative
1871.	P51814:460	THTGEKHYECNECGKAFTRKSALRMHQRI	Negative
1872.	P51814:465	KHYECNECGKAFTRKSALRMHQRIHTGEK	Negative
1873.	P51814:479	KSALRMHQRIHTGEKPYVCADCGKAFIQK	Negative
1874.	P51814:488	IHTGEKPYVCADCGKAFIQKSHFNTHQRI	Negative
1875.	P51814:493	KPYVCADCGKAFIQKSHFNTHQRIHTGEK	Negative
1876.	P51814:507	KSHFNTHQRIHTGEKPYECSDCGKSFTKK	Negative
1877.	P51814:516	IHTGEKPYECSDCGKSFTKKSQLHVHQRI	Negative
1878.	P51814:520	EKPYECSDCGKSFTKKSQLHVHQRIHTGE	Negative
1879.	P51814:521	KPYECSDCGKSFTKKSQLHVHQRIHTGEK	Negative
1880.	P51814:535	KSQLHVHQRIHTGEKPYICTECGKVFTHR	Negative
1881.	P51814:544	IHTGEKPYICTECGKVFTHRTNLTTHQKT	Negative
1882.	P51814:557	GKVFTHRTNLTTHQKTHTGEKPYMCAECG	Negative

1883.	P51814:563	RTNLTTHQKTHTGEKPYMCAECGKAFTDQ	Negative
1884.	P51814:572	THTGEKPYMCAECGKAFTDQSNLIKHQKT	Negative
1885.	P51814:582	AECGKAFTDQSNLIKHQKTHTGEKPYKCN	Negative
1886.	P51814:585	GKAFTDQSNLIKHQKTHTGEKPYKCNGCG	Negative
1887.	P51814:591	QSNLIKHQKTHTGEKPYKCNGCGKAFIWK	Negative
1888.	P51814:594	LIKHQKTHTGEKPYKCNGCGKAFIWKSRL	Negative
1889.	P51814:600	THTGEKPYKCNGCGKAFIWKSRLKIHQKS	Negative
1890.	P51814:605	KPYKCNGCGKAFIWKSRLKIHQKSHIGER	Negative
1891.	P51814:609	CNGCGKAFIWKSRLKIHQKSHIGERHYEC	Negative
1892.	P51814:613	GKAFIWKSRLKIHQKSHIGERHYECKDCG	Negative
1893.	P51814:624	IHQKSHIGERHYECKDCGKAFIQKSTLSV	Negative
1894.	P51814:628	SHIGERHYECKDCGKAFIQKSTLSVHQRI	Negative
1895.	P51814:633	RHYECKDCGKAFIQKSTLSVHQRIHTGEK	Negative
1896.	P51814:647	KSTLSVHQRIHTGEKPYVCPECGKAFIQK	Negative
1897.	P51814:656	IHTGEKPYVCPECGKAFIQKSHFIAHHRI	Negative
1898.	P51814:661	KPYVCPECGKAFIQKSHFIAHHRIHTGEK	Negative
1899.	P51814:675	KSHFIAHHRIHTGEKPYECSDCGKCFTKK	Negative
1900.	P51814:684	IHTGEKPYECSDCGKCFTKKSQLRVHQKI	Negative
1901.	P51814:688	EKPYECSDCGKCFTKKSQLRVHQKIHTGE	Negative
1902.	P51814:689	KPYECSDCGKCFTKKSQLRVHQKIHTGEK	Negative
1903.	P51814:697	GKCFTKKSQLRVHQKIHTGEKPNICAECG	Negative
1904.	P51814:703	KSQLRVHQKIHTGEKPNICAECGKAFTDR	Negative
1905.	P51814:712	IHTGEKPNICAECGKAFTDRSNLITHQKI	Negative
1906.	P51814:725	GKAFTDRSNLITHQKIHTREKPYECGDCG	Negative
1907.	P51814:731	RSNLITHQKIHTREKPYECGDCGKTFTWK	Negative
1908.	P51814:740	IHTREKPYECGDCGKTFTWKSRLNIHQKS	Negative
1909.	P51814:745	KPYECGDCGKTFTWKSRLNIHQKSHTGER	Negative
1910.	P51814:753	GKTFTWKSRLNIHQKSHTGERHYECSKCG	Negative
1911.	P51814:765	HQKSHTGERHYECSKCGKAFIQKATLSMH	Negative
1912.	P51814:768	SHTGERHYECSKCGKAFIQKATLSMHQII	Negative
1913.	P51814:773	RHYECSKCGKAFIQKATLSMHQIIHTGKK	Negative
1914.	P51814:786	QKATLSMHQIIHTGKKPYACTECQKAFTD	Negative
1915.	P51814:787	KATLSMHQIIHTGKKPYACTECQKAFTDR	Negative
1916.	P51814:796	IHTGKKPYACTECQKAFTDRSNLIKHQKM	Negative
1917.	P51814:806	TECQKAFTDRSNLIKHQKMHSGEKRYKAS	Negative
1918.	P51814:809	QKAFTDRSNLIKHQKMHSGEKRYKASDXX	Negative
1919.	P51814:815	RSNLIKHQKMHSGEKRYKASDXXXXXXXX	Negative
1920.	P51814:818	LIKHQKMHSGEKRYKASDXXXXXXXXXXX	Negative
1921.	P60174:43	DLQRLGSSAMAPSRKFFVGGNWKMNGRKQ	Negative
1922.	P60174:51	AMAPSRKFFVGGNWKMNGRKQSLGELIGT	Negative
1923.	P60174:56	RKFFVGGNWKMNGRKQSLGELIGTLNAAK	Negative
1924.	P60174:70	KQSLGELIGTLNAAKVPADTEVVCAPPTA	Negative
1925.	P60174:92	VCAPPTAYIDFARQKLDPKIAVAAQNCYK	Negative
1926.	P60174:96	PTAYIDFARQKLDPKIAVAAQNCYKVTNG	Negative
1927.	P60174:106	KLDPKIAVAAQNCYKVTNGAFTGEISPGM	Negative
1928.	P60174:122	TNGAFTGEISPGMIKDCGATWVVLGHSER	Negative
1929.	P60174:150	RRHVFGESDELIGQKVAHALAEGLGVIAC	Negative
1930.	P60174:168	ALAEGLGVIACIGEKLDEREAGITEKVVF	Negative
1931.	P60174:179	IGEKLDEREAGITEKVVFEQTKVIADNVK	Negative
1932.	P60174:186	REAGITEKVVFEQTKVIADNVKDWSKVVL	Negative
1933.	P60174:193	KVVFEQTKVIADNVKDWSKVVLAYEPVWA	Negative
1934.	P60174:197	EQTKVIADNVKDWSKVVLAYEPVWAIGTG	Negative
1935.	P60174:212	VVLAYEPVWAIGTGKTATPQQAQEVHEKL	Negative
1936.	P60174:225	GKTATPQQAQEVHEKLRGWLKSNVSDAVA	Negative

1937.	P60174:231	QQAQEVHEKLRGWLKSNVSDAVAQSTRII	Negative
1938.	P60174:275	QPDVDGFLVGGASLKPEFVDIINAKQXXX	Negative
1939.	P60174:285	GASLKPEFVDIINAKQXXXXXXXXXXXXX	Negative
1940.	P62814:64	VSGVNGPLVILDHVKFPRYAEIVHLTLPD	Negative
1941.	P62814:81	RYAEIVHLTLPDGTKRSGQVLEVSGSKAV	Negative
1942.	P62814:93	GTKRSGQVLEVSGSKAVVQVFEGTSGIDA	Negative
1943.	P62814:108	AVVQVFEGTSGIDAKKTSCEFTGDILRTP	Negative
1944.	P62814:109	VVQVFEGTSGIDAKKTSCEFTGDILRTPV	Negative
1945.	P62814:137	VSEDMLGRVFNGSGKPIDRGPVVLAEDFL	Negative
1946.	P62814:188	ISAIDGMNSIARGQKIPIFSAAGLPHNEI	Negative
1947.	P62814:214	NEIAAQICRQAGLVKKSKDVVDYSEENFA	Negative
1948.	P62814:215	EIAAQICRQAGLVKKSKDVVDYSEENFAI	Negative
1949.	P62814:217	AAQICRQAGLVKKSKDVVDYSEENFAIVF	Negative
1950.	P62814:245	FAAMGVNMETARFFKSDFEENGSMDNVCL	Negative
1951.	P62814:291	LALTTAEFLAYQCEKHVLVILTDMSSYAE	Negative
1952.	P62814:403	YPPINVLPSLSRLMKSAIGEGMTRKDHAD	Negative
1953.	P62814:413	SRLMKSAIGEGMTRKDHADVSNQLYACYA	Negative
1954.	P62814:430	ADVSNQLYACYAIGKDVQAMKAVVGEEAL	Negative
1955.	P62814:436	LYACYAIGKDVQAMKAVVGEEALTSDDLL	Negative
1956.	P62814:457	ALTSDDLLYLEFLQKFEKNFITQGPYENR	Negative
1957.	P62814:460	SDDLLYLEFLQKFEKNFITQGPYENRTVY	Negative
1958.	P62814:489	ETLDIGWQLLRIFPKEMLKRIPQSTLSEF	Negative
1959.	P62814:493	IGWQLLRIFPKEMLKRIPQSTLSEFYPRD	Negative
1960.	P62814:510	PQSTLSEFYPRDSAKHXXXXXXXXXXXXX	Negative
1961.	P62900:5	XXXXXXXXXMAPAKKGGEKKKGRSAINE	Negative
1962.	P62900:6	XXXXXXXXXMAPAKKGGEKKKGRSAINEV	Negative
1963.	P62900:10	XXXXXMAPAKKGGEKKKGRSAINEVVTRE	Negative
1964.	P62900:11	XXXXMAPAKKGGEKKKGRSAINEVVTREY	Negative
1965.	P62900:12	XXXMAPAKKGGEKKKGRSAINEVVTREYT	Negative
1966.	P62900:31	INEVVTREYTINIHKRIHGVGFKKRAPRA	Negative
1967.	P62900:39	YTINIHKRIHGVGFKKRAPRALKEIRKFA	Negative
1968.	P62900:40	TINIHKRIHGVGFKKRAPRALKEIRKFAM	Negative
1969.	P62900:47	IHGVGFKKRAPRALKEIRKFAMKEMGTPD	Negative
1970.	P62900:51	GFKKRAPRALKEIRKFAMKEMGTPDVRID	Negative
1971.	P62900:55	RAPRALKEIRKFAMKEMGTPDVRIDTRLN	Negative
1972.	P62900:75	DVRIDTRLNKAVWAKGIRNVPYRIRVRLS	Negative
1973.	P62900:91	IRNVPYRIRVRLSRKRNEDEDSPNKLYTL	Negative
1974.	P62900:101	RLSRKRNEDEDSPNKLYTLVTYVPVTTFK	Negative
1975.	P62900:115	KLYTLVTYVPVTTFKNLQTVNVDENXXXX	Negative
1976.	P62908:7	XXXXXXXXMAVQISKKRKFVADGIFKAEL	Negative
1977.	P62908:8	XXXXXXXMAVQISKKRKFVADGIFKAELN	Negative
1978.	P62908:10	XXXXXMAVQISKKRKFVADGIFKAELNEF	Negative
1979.	P62908:18	QISKKRKFVADGIFKAELNEFLTRELAED	Negative
1980.	P62908:62	IIILATRTQNVLGEKGRRIRELTAVVQKR	Negative
1981.	P62908:75	EKGRRIRELTAVVQKRFGFPEGSVELYAE	Negative
1982.	P62908:90	RFGFPEGSVELYAEKVATRGLCAIAQAES	Negative
1983.	P62908:108	RGLCAIAQAESLRYKLLGGLAVRRACYGV	Negative
1984.	P62908:132	ACYGVLRFIMESGAKGCEVVVSGKLRGQR	Negative
1985.	P62908:141	MESGAKGCEVVVSGKLRGQRAKSMKFVDG	Negative
1986.	P62908:148	CEVVVSGKLRGQRAKSMKFVDGLMIHSGD	Negative
1987.	P62908:151	VVSGKLRGQRAKSMKFVDGLMIHSGDPVN	Negative
1988.	P62908:185	AVRHVLLRQGVLGIKVKIMLPWDPSGKIG	Negative
1989.	P62908:187	RHVLLRQGVLGIKVKIMLPWDPSGKIGPK	Negative
1990.	P62908:197	GIKVKIMLPWDPSGKIGPKKPLPDHVSIV	Negative

1991.	P62908:202	IMLPWDPSGKIGPKKPLPDHVSIVEPKDE	Negative
1992.	P62908:214	PKKPLPDHVSIVEPKDEILPTTPISEQKG	Negative
1993.	P62908:227	PKDEILPTTPISEQKGGKPEPPAMPQPVP	Negative
1994.	P62908:230	EILPTTPISEQKGGKPEPPAMPQPVPTAX	Negative
1995.	P68104:3	XXXXXXXXXXXMGKEKTHINIVVIGHVD	Negative
1996.	P68104:5	XXXXXXXXXMGKEKTHINIVVIGHVDSG	Negative
1997.	P68104:20	THINIVVIGHVDSGKSTTTGHLIYKCGGI	Negative
1998.	P68104:30	VDSGKSTTTGHLIYKCGGIDKRTIEKFEK	Negative
1999.	P68104:36	TTTGHLIYKCGGIDKRTIEKFEKEAAEMG	Negative
2000.	P68104:41	LIYKCGGIDKRTIEKFEKEAAEMGKGSFK	Negative
2001.	P68104:44	KCGGIDKRTIEKFEKEAAEMGKGSFKYAW	Negative
2002.	P68104:51	RTIEKFEKEAAEMGKGSFKYAWVLDKLKA	Negative
2003.	P68104:55	KFEKEAAEMGKGSFKYAWVLDKLKAERER	Negative
2004.	P68104:62	EMGKGSFKYAWVLDKLKAERERGITIDIS	Negative
2005.	P68104:64	GKGSFKYAWVLDKLKAERERGITIDISLW	Negative
2006.	P68104:79	AERERGITIDISLWKFETSKYYVTIIDAP	Negative
2007.	P68104:84	GITIDISLWKFETSKYYVTIIDAPGHRDF	Negative
2008.	P68104:100	YVTIIDAPGHRDFIKNMITGTSQADCAVL	Negative
2009.	P68104:129	IVAAGVGEFEAGISKNGQTREHALLAYTL	Negative
2010.	P68104:146	QTREHALLAYTLGVKQLIVGVNKMDSTEP	Negative
2011.	P68104:154	AYTLGVKQLIVGVNKMDSTEPPYSQKRYE	Negative
2012.	P68104:165	GVNKMDSTEPPYSQKRYEEIVKEVSTYIK	Negative
2013.	P68104:172	TEPPYSQKRYEEIVKEVSTYIKKIGYNPD	Negative
2014.	P68104:179	KRYEEIVKEVSTYIKKIGYNPDTVAFVPI	Negative
2015.	P68104:180	RYEEIVKEVSTYIKKIGYNPDTVAFVPIS	Negative
2016.	P68104:212	GDNMLEPSANMPWFKGWKVTRKDGNASGT	Negative
2017.	P68104:215	MLEPSANMPWFKGWKVTRKDGNASGTTLL	Negative
2018.	P68104:219	SANMPWFKGWKVTRKDGNASGTTLLEALD	Negative
2019.	P68104:244	EALDCILPPTRPTDKPLRLPLQDVYKIGG	Negative
2020.	P68104:255	PTDKPLRLPLQDVYKIGGIGTVPVGRVET	Negative
2021.	P68104:273	IGTVPVGRVETGVLKPGMVVTFAPVNVTT	Negative
2022.	P68104:290	MVVTFAPVNVTTEVKSVEMHHEALSEALP	Negative
2023.	P68104:313	LSEALPGDNVGFNVKNVSVKDVRRGNVAG	Negative
2024.	P68104:318	PGDNVGFNVKNVSVKDVRRGNVAGDSKND	Negative
2025.	P68104:371	YAPVLDCHTAHIACKFAELKEKIDRRSGK	Negative
2026.	P68104:376	DCHTAHIACKFAELKEKIDRRSGKKLEDG	Negative
2027.	P68104:378	HTAHIACKFAELKEKIDRRSGKKLEDGPK	Negative
2028.	P68104:385	KFAELKEKIDRRSGKKLEDGPKFLKSGDA	Negative
2029.	P68104:386	FAELKEKIDRRSGKKLEDGPKFLKSGDAA	Negative
2030.	P68104:392	KIDRRSGKKLEDGPKFLKSGDAAIVDMVP	Negative
2031.	P68104:395	RRSGKKLEDGPKFLKSGDAAIVDMVPGKP	Negative
2032.	P68104:408	LKSGDAAIVDMVPGKPMCVESFSDYPPLG	Negative
2033.	P68104:439	AVRDMRQTVAVGVIKAVDKKAAGAGKVTK	Negative
2034.	P68104:443	MRQTVAVGVIKAVDKKAAGAGKVTKSAQK	Negative
2035.	P68104:444	RQTVAVGVIKAVDKKAAGAGKVTKSAQKA	Negative
2036.	P68104:450	GVIKAVDKKAAGAGKVTKSAQKAQKAKXX	Negative
2037.	P68104:453	KAVDKKAAGAGKVTKSAQKAQKAKXXXXX	Negative
2038.	P68104:457	KKAAGAGKVTKSAQKAQKAKXXXXXXXXX	Negative
2039.	P68104:460	AGAGKVTKSAQKAQKAKXXXXXXXXXXXX	Negative
2040.	P68104:462	AGKVTKSAQKAQKAKXXXXXXXXXXXXXX	Negative
2041.	P99028:8	XXXXXXXMGLEDERKMLTGSGDPKEEEEE	Negative
2042.	P99028:17	LEDERKMLTGSGDPKEEEEEELVDPLTTV	Negative
2043.	P99028:40	DPLTTVREHCEQLEKCVKARERLELCDNR	Negative

2044.	P99028:43	TTVREHCEQLEKCVKARERLELCDNRVSS	Negative
2045.	P99028:86	FLHARDHCVAHKLFKNLKXXXXXXXXXXX	Negative
2046.	P99028:89	ARDHCVAHKLFKNLKXXXXXXXXXXXXXX	Negative
2047.	Q13541:57	FSTTPGGTRIIYDRKFLMECRNSPVTKTP	Negative
2048.	Q13541:105	MEASQSHLRNSPEDKRAGGEESQFEMDIX	Negative
2049.	Q3UJB0:10	XXXXXMAAEHPEPPKGELQLPPPPPPGHY	Negative
2050.	Q3UJB0:77	IVLNRPVLRGEDGDKAAPPPMSAQLSGIP	Negative
2051.	Q3UJB0:148	RVGEPVALSEEERLKLAQQQAALLMQQEE	Negative
2052.	Q3UJB0:165	QQQAALLMQQEERAKQAAVLMEQERQQEI	Negative
2053.	Q3UJB0:181	AAVLMEQERQQEIAKMGTAVPRPPQDMGQ	Negative
2054.	Q3UJB0:251	GDENREMDDPSVGPKIPQALEKILQLKES	Negative
2055.	Q3UJB0:258	DDPSVGPKIPQALEKILQLKESRQEEMNS	Negative
2056.	Q3UJB0:263	GPKIPQALEKILQLKESRQEEMNSQQEEE	Negative
2057.	Q3UJB0:303	QSASETEEDTVSISKKEKNRKRRNRKKKK	Negative
2058.	Q3UJB0:304	SASETEEDTVSISKKEKNRKRRNRKKKKK	Negative
2059.	Q3UJB0:306	SETEEDTVSISKKEKNRKRRNRKKKKKPQ	Negative
2060.	Q3UJB0:309	EEDTVSISKKEKNRKRRNRKKKKKPQRVR	Negative
2061.	Q3UJB0:314	SISKKEKNRKRRNRKKKKKPQRVRAASSE	Negative
2062.	Q3UJB0:315	ISKKEKNRKRRNRKKKKKPQRVRAASSES	Negative
2063.	Q3UJB0:316	SKKEKNRKRRNRKKKKKPQRVRAASSESS	Negative
2064.	Q3UJB0:317	KKEKNRKRRNRKKKKKPQRVRAASSESSG	Negative
2065.	Q3UJB0:318	KEKNRKRRNRKKKKKPQRVRAASSESSGD	Negative
2066.	Q3UJB0:335	RVRAASSESSGDREKDSGRSRGSDPPAAD	Negative
2067.	Q3UJB0:370	TEEPEIYEPNFIFFKRIFEAFKLTDDVKK	Negative
2068.	Q3UJB0:377	EPNFIFFKRIFEAFKLTDDVKKEKEKEPE	Negative
2069.	Q3UJB0:383	FKRIFEAFKLTDDVKKEKEKEPEKLDKME	Negative
2070.	Q3UJB0:384	KRIFEAFKLTDDVKKEKEKEPEKLDKMES	Negative
2071.	Q3UJB0:386	IFEAFKLTDDVKKEKEKEPEKLDKMESSA	Negative
2072.	Q3UJB0:388	EAFKLTDDVKKEKEKEPEKLDKMESSAVP	Negative
2073.	Q3UJB0:392	LTDDVKKEKEKEPEKLDKMESSAVPKKKG	Negative
2074.	Q3UJB0:395	DVKKEKEKEPEKLDKMESSAVPKKKGFEE	Negative
2075.	Q3UJB0:403	EPEKLDKMESSAVPKKKGFEEEHKDSDDD	Negative
2076.	Q3UJB0:404	PEKLDKMESSAVPKKKGFEEEHKDSDDDS	Negative
2077.	Q3UJB0:405	EKLDKMESSAVPKKKGFEEEHKDSDDDSS	Negative
2078.	Q3UJB0:412	SSAVPKKKGFEEEHKDSDDDSSDDEQEKK	Negative
2079.	Q3UJB0:425	HKDSDDDSSDDEQEKKPEAPKLSKKKLRR	Negative
2080.	Q3UJB0:426	KDSDDDSSDDEQEKKPEAPKLSKKKLRRM	Negative
2081.	Q3UJB0:431	DSSDDEQEKKPEAPKLSKKKLRRMNRFTV	Negative
2082.	Q3UJB0:434	DDEQEKKPEAPKLSKKKLRRMNRFTVAEL	Negative
2083.	Q3UJB0:435	DEQEKKPEAPKLSKKKLRRMNRFTVAELK	Negative
2084.	Q3UJB0:436	EQEKKPEAPKLSKKKLRRMNRFTVAELKQ	Negative
2085.	Q3UJB0:449	KKLRRMNRFTVAELKQLVARPDVVEMHDV	Negative
2086.	Q3UJB0:469	PDVVEMHDVTAQDPKLLVHLKATRNSVPV	Negative
2087.	Q3UJB0:475	HDVTAQDPKLLVHLKATRNSVPVPRHWCF	Negative
2088.	Q3UJB0:490	ATRNSVPVPRHWCFKRKYLQGKRGIEKPP	Negative
2089.	Q3UJB0:492	RNSVPVPRHWCFKRKYLQGKRGIEKPPFE	Negative
2090.	Q3UJB0:497	VPRHWCFKRKYLQGKRGIEKPPFELPDFI	Negative
2091.	Q3UJB0:502	CFKRKYLQGKRGIEKPPFELPDFIKRTGI	Negative
2092.	Q3UJB0:512	RGIEKPPFELPDFIKRTGIQEMREALQEK	Negative
2093.	Q3UJB0:526	KRTGIQEMREALQEKEEQKTMKSKMREKV	Negative
2094.	Q3UJB0:530	IQEMREALQEKEEQKTMKSKMREKVRPKM	Negative
2095.	Q3UJB0:533	MREALQEKEEQKTMKSKMREKVRPKMGKI	Negative
2096.	Q3UJB0:535	EALQEKEEQKTMKSKMREKVRPKMGKIDI	Negative
2097.	Q3UJB0:539	EKEEQKTMKSKMREKVRPKMGKIDIDYQK	Negative

2098.	Q3UJB0:543	QKTMKSKMREKVRPKMGKIDIDYQKLHDA	Negative
2099.	Q3UJB0:546	MKSKMREKVRPKMGKIDIDYQKLHDAFFK	Negative
2100.	Q3UJB0:553	KVRPKMGKIDIDYQKLHDAFFKWQTKPKL	Negative
2101.	Q3UJB0:560	KIDIDYQKLHDAFFKWQTKPKLTIHGDLY	Negative
2102.	Q3UJB0:564	DYQKLHDAFFKWQTKPKLTIHGDLYYEGK	Negative
2103.	Q3UJB0:566	QKLHDAFFKWQTKPKLTIHGDLYYEGKEF	Negative
2104.	Q3UJB0:578	KPKLTIHGDLYYEGKEFETRLKEKKPGDL	Negative
2105.	Q3UJB0:585	GDLYYEGKEFETRLKEKKPGDLSDELRIS	Negative
2106.	Q3UJB0:587	LYYEGKEFETRLKEKKPGDLSDELRISLG	Negative
2107.	Q3UJB0:588	YYEGKEFETRLKEKKPGDLSDELRISLGM	Negative
2108.	Q3UJB0:610	LRISLGMPVGPNAHKVPPPWLIAMQRYGP	Negative
2109.	Q3UJB0:632	AMQRYGPPPSYPNLKIPGLNSPIPESCSF	Negative
2110.	Q3UJB0:655	PESCSFGYHAGGWGKPPVDETGKPLYGDV	Negative
2111.	Q3UJB0:663	HAGGWGKPPVDETGKPLYGDVFGTNAAEF	Negative
2112.	Q3UJB0:680	YGDVFGTNAAEFQTKTEEEEIDRTPWGEL	Negative
2113.	Q3UJB0:714	ESSEEEEEEESDEDKPDETGFITPADSGL	Negative
2114.	Q3UJB0:751	VPAGMETPELIELRKKKIEEAMDGSETPQ	Negative
2115.	Q3UJB0:752	PAGMETPELIELRKKKIEEAMDGSETPQL	Negative
2116.	Q3UJB0:753	AGMETPELIELRKKKIEEAMDGSETPQLF	Negative
2117.	Q3UJB0:773	DGSETPQLFTVLPEKRTATVGGAMMGSTH	Negative
2118.	Q3UJB0:798	GSTHIYDMSTVMSRKGPAPELQGVEVALA	Negative
2119.	Q3UJB0:826	APEELELDPMAMTQKYEEHVREQQAQVEK	Negative
2120.	Q3UJB0:840	KYEEHVREQQAQVEKEDFSDMVAEHAAKQ	Negative
2121.	Q3UJB0:855	EDFSDMVAEHAAKQKQKKRKAQPQDSRGG	Negative
2122.	Q3UJB0:857	FSDMVAEHAAKQKQKKRKAQPQDSRGGSK	Negative
2123.	Q3UJB0:858	SDMVAEHAAKQKQKKRKAQPQDSRGGSKK	Negative
2124.	Q3UJB0:860	MVAEHAAKQKQKKRKAQPQDSRGGSKKYK	Negative
2125.	Q3UJB0:871	KKRKAQPQDSRGGSKKYKEFKFXXXXXXX	Negative
2126.	Q3UJB0:872	KRKAQPQDSRGGSKKYKEFKFXXXXXXXX	Negative
2127.	Q3UJB0:874	KAQPQDSRGGSKKYKEFKFXXXXXXXXXX	Negative
2128.	Q3UJB0:877	PQDSRGGSKKYKEFKFXXXXXXXXXXXXX	Negative
2129.	Q3UL97:7	XXXXXXXXMESPDRKRQKVLKAKKTMPTS	Negative
2130.	Q3UL97:10	XXXXXMESPDRKRQKVLKAKKTMPTSYQK	Negative
2131.	Q3UL97:13	XXMESPDRKRQKVLKAKKTMPTSYQKQLE	Negative
2132.	Q3UL97:15	MESPDRKRQKVLKAKKTMPTSYQKQLEIL	Negative
2133.	Q3UL97:16	ESPDRKRQKVLKAKKTMPTSYQKQLEILN	Negative
2134.	Q3UL97:24	KVLKAKKTMPTSYQKQLEILNKSTNVEAP	Negative
2135.	Q3UL97:31	TMPTSYQKQLEILNKSTNVEAPKTTVGTN	Negative
2136.	Q3UL97:39	QLEILNKSTNVEAPKTTVGTNIPNGHNQK	Negative
2137.	Q3UL97:57	GTNIPNGHNQKMFSKNKENVKVMKVSEQI	Negative
2138.	Q3UL97:59	NIPNGHNQKMFSKNKENVKVMKVSEQINE	Negative
2139.	Q3UL97:63	GHNQKMFSKNKENVKVMKVSEQINENACG	Negative
2140.	Q3UL97:66	QKMFSKNKENVKVMKVSEQINENACGALE	Negative
2141.	Q3UL97:90	CGALERHTALLEQVKHWIRQEICMINCNL	Negative
2142.	Q3UL97:107	IRQEICMINCNLFDKKLNELNERIGKTQC	Negative
2143.	Q3UL97:108	RQEICMINCNLFDKKLNELNERIGKTQCK	Negative
2144.	Q3UL97:118	LFDKKLNELNERIGKTQCKSRHEAIAGEL	Negative
2145.	Q3UL97:122	KLNELNERIGKTQCKSRHEAIAGELFVKI	Negative
2146.	Q3UL97:135	CKSRHEAIAGELFVKIRRLQKRIKTVLSS	Negative
2147.	Q3UL97:141	AIAGELFVKIRRLQKRIKTVLSSQRNCLE	Negative
2148.	Q3UL97:144	GELFVKIRRLQKRIKTVLSSQRNCLEPNT	Negative
2149.	Q3UL97:166	NCLEPNTLPSNTVCKVTDSEAMNLNVTQK	Negative
2150.	Q3UL97:180	KVTDSEAMNLNVTQKSVKSRSKRISSVNH	Negative
2151.	Q3UL97:183	DSEAMNLNVTQKSVKSRSKRISSVNHTPL	Negative

2152.	Q3UL97:187	MNLNVTQKSVKSRSKRISSVNHTPLNSSE	Negative
2153.	Q3UL97:202	RISSVNHTPLNSSEKAGRKTNLPSTCVEF	Negative
2154.	Q3UL97:206	VNHTPLNSSEKAGRKTNLPSTCVEFASES	Negative
2155.	Q3UL97:231	ASESNTDDVMLISVKNSNLTTSITSEQTE	Negative
2156.	Q3UL97:248	NLTTSITSEQTEIRKNTSRNLSNSPNSMI	Negative
2157.	Q3UL97:263	NTSRNLSNSPNSMIKVGPVEKKFDFVIDL	Negative
2158.	Q3UL97:269	SNSPNSMIKVGPVEKKFDFVIDLTREGPS	Negative
2159.	Q3UL97:270	NSPNSMIKVGPVEKKFDFVIDLTREGPSN	Negative
2160.	Q3UL97:295	GPSNYSIESPSFTLKSTSKAVLRSKEIIP	Negative
2161.	Q3UL97:299	YSIESPSFTLKSTSKAVLRSKEIIPVAEN	Negative
2162.	Q3UL97:305	SFTLKSTSKAVLRSKEIIPVAENGNEGFG	Negative
2163.	Q3UL97:340	PLPEPPAPLPEMADKIKDTLPPQKPELKV	Negative
2164.	Q3UL97:342	PEPPAPLPEMADKIKDTLPPQKPELKVKW	Negative
2165.	Q3UL97:349	PEMADKIKDTLPPQKPELKVKWVLRPTSI	Negative
2166.	Q3UL97:353	DKIKDTLPPQKPELKVKWVLRPTSIALTW	Negative
2167.	Q3UL97:355	IKDTLPPQKPELKVKWVLRPTSIALTWNI	Negative
2168.	Q3UL97:371	VLRPTSIALTWNIPKVNPNCAPVESYHLF	Negative
2169.	Q3UL97:397	HLFLYYENSDHLTWKKIAEIKALPLPMAC	Negative
2170.	Q3UL97:398	LFLYYENSDHLTWKKIAEIKALPLPMACT	Negative
2171.	Q3UL97:403	ENSDHLTWKKIAEIKALPLPMACTLSQNL	Negative
2172.	Q3UL97:421	LPMACTLSQNLASTKYYFAVQSKDIFGRY	Negative
2173.	Q3UL97:429	QNLASTKYYFAVQSKDIFGRYGPFCNIKS	Negative
2174.	Q3UL97:442	SKDIFGRYGPFCNIKSIPRFSENLTXXXX	Negative
2175.	Q3UNI1:34	AMEAASQPADEPLRKRPRRDGPGLGRSPG	Negative
2176.	Q3UNI1:156	QQHLMIGTDPRTILKDLLPETIPPPELDD	Negative
2177.	Q3UNI1:186	TLWQIVINILSEPPKRKKRKDINTIEDAV	Negative
2178.	Q3UNI1:188	WQIVINILSEPPKRKKRKDINTIEDAVKL	Negative
2179.	Q3UNI1:189	QIVINILSEPPKRKKRKDINTIEDAVKLL	Negative
2180.	Q3UNI1:191	VINILSEPPKRKKRKDINTIEDAVKLLQE	Negative
2181.	Q3UNI1:201	RKKRKDINTIEDAVKLLQECKKIIVLTGA	Negative
2182.	Q3UNI1:207	INTIEDAVKLLQECKKIIVLTGAGVSVSC	Negative
2183.	Q3UNI1:208	NTIEDAVKLLQECKKIIVLTGAGVSVSCG	Negative
2184.	Q3UNI1:257	LPDPQAMFDIEYFRKDPRPFFKFAKEIYP	Negative
2185.	Q3UNI1:264	FDIEYFRKDPRPFFKFAKEIYPGQFQPSL	Negative
2186.	Q3UNI1:267	EYFRKDPRPFFKFAKEIYPGQFQPSLCHK	Negative
2187.	Q3UNI1:281	KEIYPGQFQPSLCHKFIALSDKEGKLLRN	Negative
2188.	Q3UNI1:288	FQPSLCHKFIALSDKEGKLLRNYTQNIDT	Negative
2189.	Q3UNI1:291	SLCHKFIALSDKEGKLLRNYTQNIDTLEQ	Negative
2190.	Q3UNI1:328	QCHGSFATASCLICKYKVDCEAVRGDIFN	Negative
2191.	Q3UNI1:330	HGSFATASCLICKYKVDCEAVRGDIFNQV	Negative
2192.	Q3UNI1:361	RCPRCPADEPLAIMKPEIVFFGENLPEQF	Negative
2193.	Q3UNI1:380	FFGENLPEQFHRAMKYDKDEVDLLIVIGS	Negative
2194.	Q3UNI1:383	ENLPEQFHRAMKYDKDEVDLLIVIGSSLK	Negative
2195.	Q3UNI1:397	KDEVDLLIVIGSSLKVRPVALIPSSIPHE	Negative
2196.	Q3UNI1:452	IINELCHRLGGEYAKLCCNPVKLSEITEK	Negative
2197.	Q3UNI1:459	RLGGEYAKLCCNPVKLSEITEKPPRPQKE	Negative
2198.	Q3UNI1:466	KLCCNPVKLSEITEKPPRPQKELVHLSEL	Negative
2199.	Q3UNI1:472	VKLSEITEKPPRPQKELVHLSELPPTPLH	Negative
2200.	Q3UNI1:531	VNDLEVSESSCVEEKPQEVQTSRNVENIN	Negative
2201.	Q3UNI1:552	SRNVENINVENPDFKAVGSSTADKNERTS	Negative
2202.	Q3UNI1:561	ENPDFKAVGSSTADKNERTSVAETVRKCW	Negative
2203.	Q3UNI1:573	ADKNERTSVAETVRKCWPNRLAKEQISKR	Negative
2204.	Q3UNI1:581	VAETVRKCWPNRLAKEQISKRLEGNQYLF	Negative
2205.	Q3UNI1:586	RKCWPNRLAKEQISKRLEGNQYLFVPPNR	Negative

2206.	Q5JSZ5:10	XXXXXMSDRLGQITKGKDGKSKYSTLSLF	Negative
2207.	Q5JSZ5:12	XXXMSDRLGQITKGKDGKSKYSTLSLFDK	Negative
2208.	Q5JSZ5:15	MSDRLGQITKGKDGKSKYSTLSLFDKYKG	Negative
2209.	Q5JSZ5:17	DRLGQITKGKDGKSKYSTLSLFDKYKGKS	Negative
2210.	Q5JSZ5:26	KDGKSKYSTLSLFDKYKGKSVDAIRSSVI	Negative
2211.	Q5JSZ5:28	GKSKYSTLSLFDKYKGKSVDAIRSSVIPR	Negative
2212.	Q5JSZ5:30	SKYSTLSLFDKYKGKSVDAIRSSVIPRHG	Negative
2213.	Q5JSZ5:50	RSSVIPRHGLQSLGKVAAARRMPPPANLP	Negative
2214.	Q5JSZ5:67	AARRMPPPANLPSLKSENKGNDPNIVIVP	Negative
2215.	Q5JSZ5:71	MPPPANLPSLKSENKGNDPNIVIVPKDGT	Negative
2216.	Q5JSZ5:82	SENKGNDPNIVIVPKDGTGWANKQDQQDP	Negative
2217.	Q5JSZ5:90	NIVIVPKDGTGWANKQDQQDPKSSSATAS	Negative
2218.	Q5JSZ5:97	DGTGWANKQDQQDPKSSSATASQPPESLP	Negative
2219.	Q5JSZ5:117	ASQPPESLPQPGLQKSVSNLQKPTQSISQ	Negative
2220.	Q5JSZ5:124	LPQPGLQKSVSNLQKPTQSISQENTNSVP	Negative
2221.	Q5JSZ5:142	SISQENTNSVPGGPKSWAQLNGKPVGHEG	Negative
2222.	Q5JSZ5:150	SVPGGPKSWAQLNGKPVGHEGGLRGSSRL	Negative
2223.	Q5JSZ5:176	SRLLSFSPEEFPTLKAAGGQDKAGKEKGV	Negative
2224.	Q5JSZ5:183	PEEFPTLKAAGGQDKAGKEKGVLDLSYGP	Negative
2225.	Q5JSZ5:186	FPTLKAAGGQDKAGKEKGVLDLSYGPGPS	Negative
2226.	Q5JSZ5:188	TLKAAGGQDKAGKEKGVLDLSYGPGPSLR	Negative
2227.	Q5JSZ5:251	TGDGAPSSACTSDSKDPSLRPAQPVRKGA	Negative
2228.	Q5JSZ5:263	DSKDPSLRPAQPVRKGASQFMGNVYHPPT	Negative
2229.	Q5JSZ5:290	PTYHDMLPAFMCSPKSSENQGTVERGSFP	Negative
2230.	Q5JSZ5:327	RVPFRQFQMNDQDGKENRLGLSRPLRPLR	Negative
2231.	Q5JSZ5:359	ERAPRPTIINAENLKGLDDLDADADDGWA	Negative
2232.	Q5JSZ5:384	DGWAGLHEEVDYSEKLKFSDDEEEEEVVK	Negative
2233.	Q5JSZ5:403	DDEEEEEVVKDGRPKWNSWDPRRQRQLSM	Negative
2234.	Q5JSZ5:426	QRQLSMSSADSADAKRTREEGKDWAEAVG	Negative
2235.	Q5JSZ5:433	SADSADAKRTREEGKDWAEAVGASRVVRK	Negative
2236.	Q5JSZ5:447	KDWAEAVGASRVVRKAPDPQPPPRKLHGW	Negative
2237.	Q5JSZ5:457	RVVRKAPDPQPPPRKLHGWAPGPDYQKSS	Negative
2238.	Q5JSZ5:469	PRKLHGWAPGPDYQKSSMGSMFRQQSIED	Negative
2239.	Q5JSZ5:484	SSMGSMFRQQSIEDKEDKPPPRQKFIQSE	Negative
2240.	Q5JSZ5:487	GSMFRQQSIEDKEDKPPPRQKFIQSEMSE	Negative
2241.	Q5JSZ5:493	QSIEDKEDKPPPRQKFIQSEMSEAVERAR	Negative
2242.	Q5JSZ5:508	FIQSEMSEAVERARKRREEEERRAREERL	Negative
2243.	Q5JSZ5:528	ERRAREERLAACAAKLKQLDQKCKQARKA	Negative
2244.	Q5JSZ5:530	RAREERLAACAAKLKQLDQKCKQARKAGE	Negative
2245.	Q5JSZ5:535	RLAACAAKLKQLDQKCKQARKAGEARKQA	Negative
2246.	Q5JSZ5:537	AACAAKLKQLDQKCKQARKAGEARKQAEK	Negative
2247.	Q5JSZ5:541	AKLKQLDQKCKQARKAGEARKQAEKEVPW	Negative
2248.	Q5JSZ5:547	DQKCKQARKAGEARKQAEKEVPWSPSAEK	Negative
2249.	Q5JSZ5:551	KQARKAGEARKQAEKEVPWSPSAEKASPQ	Negative
2250.	Q5JSZ5:561	KQAEKEVPWSPSAEKASPQENGPAVHKGS	Negative
2251.	Q5JSZ5:573	AEKASPQENGPAVHKGSPEFPAQETPTTF	Negative
2252.	Q5JSZ5:619	EEEAREAGSPAQEFKYQKSLPPRFQRQQQ	Negative
2253.	Q5JSZ5:622	AREAGSPAQEFKYQKSLPPRFQRQQQQQQ	Negative
2254.	Q5JSZ5:642	FQRQQQQQQEQLYKMQHWQPVYPPPSHP	Negative
2255.	Q5JSZ5:706	VDFYPSALHPSGLMKPMMPQESLNGTGCR	Negative
2256.	Q5JSZ5:734	RSEDQNCVPPLQERKVTPIDSPPVWSPEG	Negative
2257.	Q5JSZ5:755	PPVWSPEGYMALQSKGYPLPHPKSSDTLA	Negative
2258.	Q5JSZ5:763	YMALQSKGYPLPHPKSSDTLAMDMRVRNE	Negative
2259.	Q5JSZ5:810	LFEERGEEYLSAFDKKAQADFDSCISSQR	Negative

2260.	Q5JSZ5:811	FEERGEEYLSAFDKKAQADFDSCISSQRI	Negative
2261.	Q5JSZ5:864	LRCSPLEPDFVPDEKKPECGSWDVSHQPE	Negative
2262.	Q5JSZ5:865	RCSPLEPDFVPDEKKPECGSWDVSHQPET	Negative
2263.	Q5JSZ5:904	TPREGTAFNISSWDKNGSPNKQPSSEPEW	Negative
2264.	Q5JSZ5:910	AFNISSWDKNGSPNKQPSSEPEWTPEPRS	Negative
2265.	Q5JSZ5:943	HPEQTGRTRRSGPIKKPVLKALKVEDKEK	Negative
2266.	Q5JSZ5:944	PEQTGRTRRSGPIKKPVLKALKVEDKEKE	Negative
2267.	Q5JSZ5:948	GRTRRSGPIKKPVLKALKVEDKEKELEKI	Negative
2268.	Q5JSZ5:951	RRSGPIKKPVLKALKVEDKEKELEKIKQE	Negative
2269.	Q5JSZ5:955	PIKKPVLKALKVEDKEKELEKIKQELGEE	Negative
2270.	Q5JSZ5:957	KKPVLKALKVEDKEKELEKIKQELGEEST	Negative
2271.	Q5JSZ5:961	LKALKVEDKEKELEKIKQELGEESTRLAK	Negative
2272.	Q5JSZ5:963	ALKVEDKEKELEKIKQELGEESTRLAKEK	Negative
2273.	Q5JSZ5:975	KIKQELGEESTRLAKEKEQSPTAEKDEDE	Negative
2274.	Q5JSZ5:977	KQELGEESTRLAKEKEQSPTAEKDEDEEN	Negative
2275.	Q5JSZ5:985	TRLAKEKEQSPTAEKDEDEENDASLANSS	Negative
2276.	Q5JSZ5:1006	DASLANSSTTTLEDKGPGHATFGREATKF	Negative
2277.	Q5JSZ5:1019	DKGPGHATFGREATKFEEEEKPDKAWEAR	Negative
2278.	Q5JSZ5:1025	ATFGREATKFEEEEKPDKAWEARPPRESS	Negative
2279.	Q5JSZ5:1028	GREATKFEEEEKPDKAWEARPPRESSDVP	Negative
2280.	Q5JSZ5:1045	EARPPRESSDVPPMKRNNWIFIDEEQAFG	Negative
2281.	Q5JSZ5:1125	GLREFARPEDCPRAKPRRRVASETHSEGS	Negative
2282.	Q5JSZ5:1146	SETHSEGSEYEELPKRRRQRGSENGNEGS	Negative
2283.	Q5JSZ5:1170	GNEGSLLEREESTLKKGDCRDSWRSNKGC	Negative
2284.	Q5JSZ5:1171	NEGSLLEREESTLKKGDCRDSWRSNKGCS	Negative
2285.	Q5JSZ5:1182	TLKKGDCRDSWRSNKGCSEDHSGLDAKSR	Negative
2286.	Q5JSZ5:1194	SNKGCSEDHSGLDAKSRGPRAFGRALPPR	Negative
2287.	Q5JSZ5:1222	RLSNCGYGRRTFVSKESPHWQSKSPGSSW	Negative
2288.	Q5JSZ5:1230	RRTFVSKESPHWQSKSPGSSWQEYGPSDT	Negative
2289.	Q5JSZ5:1279	AFGGRGFEDSRAEDKRSFFQDEHVADSEN	Negative
2290.	Q5JSZ5:1309	ENRPFRRRRPPRQDKPPRFRRLRQERESL	Negative
2291.	Q5JSZ5:1344	EEPHLLAGQWPGRPKLCSGDKSGTVGRRS	Negative
2292.	Q5JSZ5:1350	AGQWPGRPKLCSGDKSGTVGRRSPELSYQ	Negative
2293.	Q5JSZ5:1413	SQVDGGLSGASLGEKKELAKRSFSSQRPV	Negative
2294.	Q5JSZ5:1414	QVDGGLSGASLGEKKELAKRSFSSQRPVV	Negative
2295.	Q5JSZ5:1418	GLSGASLGEKKELAKRSFSSQRPVVDRQS	Negative
2296.	Q5JSZ5:1434	SFSSQRPVVDRQSRKLEPGGFGEKPVRPG	Negative
2297.	Q5JSZ5:1443	DRQSRKLEPGGFGEKPVRPGGGDTSPRYE	Negative
2298.	Q5JSZ5:1466	TSPRYESQQNGTPLKVKRSPDEALPGGLS	Negative
2299.	Q5JSZ5:1468	PRYESQQNGTPLKVKRSPDEALPGGLSGC	Negative
2300.	Q5JSZ5:1509	RAAHASADLPEASSKKAEKEAKLAAPRAG	Negative
2301.	Q5JSZ5:1510	AAHASADLPEASSKKAEKEAKLAAPRAGE	Negative
2302.	Q5JSZ5:1513	ASADLPEASSKKAEKEAKLAAPRAGEQGE	Negative
2303.	Q5JSZ5:1516	DLPEASSKKAEKEAKLAAPRAGEQGEAMK	Negative
2304.	Q5JSZ5:1530	KLAAPRAGEQGEAMKQFDLNYGSAIIENC	Negative
2305.	Q5JSZ5:1568	VGSMVGEGFIEVLTKKQRRLLEEERRKKE	Negative
2306.	Q5JSZ5:1569	GSMVGEGFIEVLTKKQRRLLEEERRKKEQ	Negative
2307.	Q5JSZ5:1580	LTKKQRRLLEEERRKKEQAVQVPVKGRGL	Negative
2308.	Q5JSZ5:1581	TKKQRRLLEEERRKKEQAVQVPVKGRGLS	Negative
2309.	Q5JSZ5:1590	EERRKKEQAVQVPVKGRGLSSRIPPRFAK	Negative
2310.	Q5JSZ5:1604	KGRGLSSRIPPRFAKKQNNLCLEQGDVTV	Negative
2311.	Q5JSZ5:1605	GRGLSSRIPPRFAKKQNNLCLEQGDVTVP	Negative
2312.	Q5JSZ5:1647	QALPVQAPANDSWRKAVTAFSSTETGSAE	Negative
2313.	Q5JSZ5:1665	AFSSTETGSAEQGFKSSQGDSGVDLSAES	Negative

2314.	Q5JSZ5:1697	SATSSQRSSPYGTLKPEEMSGPGLAEPKA	Negative
2315.	Q5JSZ5:1710	LKPEEMSGPGLAEPKADSHKEQAPKPSEQ	Negative
2316.	Q5JSZ5:1715	MSGPGLAEPKADSHKEQAPKPSEQKDSEQ	Negative
2317.	Q5JSZ5:1720	LAEPKADSHKEQAPKPSEQKDSEQGSGQS	Negative
2318.	Q5JSZ5:1725	ADSHKEQAPKPSEQKDSEQGSGQSKEHRP	Negative
2319.	Q5JSZ5:1735	PSEQKDSEQGSGQSKEHRPGPIGNERSLK	Negative
2320.	Q5JSZ5:1749	KEHRPGPIGNERSLKNRKGSEGAERLQGA	Negative
2321.	Q5JSZ5:1752	RPGPIGNERSLKNRKGSEGAERLQGAVVP	Negative
2322.	Q5JSZ5:1791	SVLPVPPIEFGVSPKDSDFSLPPGSASGP	Negative
2323.	Q5JSZ5:1812	PPGSASGPTGSPVVKLQDALASNAGLTQS	Negative
2324.	Q5JSZ5:1855	GLSPMSFPTADLTLKMESARKAWENSPSL	Negative
2325.	Q5JSZ5:1861	FPTADLTLKMESARKAWENSPSLPEQSSP	Negative
2326.	Q5JSZ5:2019	SPPSTMILSGGTALKPPYSAFPGMQPLEM	Negative
2327.	Q5JSZ5:2035	PYSAFPGMQPLEMVKPQSGSPYQPMSGNQ	Negative
2328.	Q5JSZ5:2134	PVLNTSREPSQMEMKGFHFADSKQNVPSG	Negative
2329.	Q5JSZ5:2142	PSQMEMKGFHFADSKQNVPSGGPVPSPQT	Negative
2330.	Q5JSZ5:2167	SPQTYRPSSASPSGKPSGSAVNMGSVQGH	Negative
2331.	Q5JSZ5:2187	VNMGSVQGHYVQQAKQRVDEKPSLGAVKL	Negative
2332.	Q5JSZ5:2193	QGHYVQQAKQRVDEKPSLGAVKLQEAPSA	Negative
2333.	Q5JSZ5:2200	AKQRVDEKPSLGAVKLQEAPSAASQMKRT	Negative
2334.	Q5JSZ5:2212	AVKLQEAPSAASQMKRTGAIKPRAVKVEE	Negative
2335.	Q5JSZ5:2218	APSAASQMKRTGAIKPRAVKVEESKAXXX	Negative
2336.	Q5JSZ5:2223	SQMKRTGAIKPRAVKVEESKAXXXXXXXX	Negative
2337.	Q5JSZ5:2228	TGAIKPRAVKVEESKAXXXXXXXXXXXXX	Negative
2338.	Q5RL73:26	FDHHVQRAVCDTRAKYREGRRPRAVKVYT	Negative
2339.	Q5RL73:37	TRAKYREGRRPRAVKVYTINLESQYLLIQ	Negative
2340.	Q5RL73:60	QYLLIQGVPAVGVMKELVERFALYGAIEQ	Negative
2341.	Q5RL73:93	DEYPAEDFTEVYLIKFMNLQSARTAKRKM	Negative
2342.	Q5RL73:104	YLIKFMNLQSARTAKRKMDEQSFFGGLLH	Negative
2343.	Q5RL73:106	IKFMNLQSARTAKRKMDEQSFFGGLLHVC	Negative
2344.	Q5RL73:133	VCYAPEFETVEETRKKLQMRKAYVVKTTE	Negative
2345.	Q5RL73:134	CYAPEFETVEETRKKLQMRKAYVVKTTEN	Negative
2346.	Q5RL73:139	FETVEETRKKLQMRKAYVVKTTENKDHYV	Negative
2347.	Q5RL73:144	ETRKKLQMRKAYVVKTTENKDHYVTKKKL	Negative
2348.	Q5RL73:149	LQMRKAYVVKTTENKDHYVTKKKLVTEHK	Negative
2349.	Q5RL73:155	YVVKTTENKDHYVTKKKLVTEHKDTEDFR	Negative
2350.	Q5RL73:156	VVKTTENKDHYVTKKKLVTEHKDTEDFRQ	Negative
2351.	Q5RL73:157	VKTTENKDHYVTKKKLVTEHKDTEDFRQD	Negative
2352.	Q5RL73:163	KDHYVTKKKLVTEHKDTEDFRQDFHSEMS	Negative
2353.	Q5RL73:181	DFRQDFHSEMSGFCKAALNTSAGNSNPYL	Negative
2354.	Q5RL73:209	LPYSCELPLCYFSSKCMCSSGGPVDRAPD	Negative
2355.	Q5RL73:226	CSSGGPVDRAPDSSKDGRNHHKTMGHYNH	Negative
2356.	Q5RL73:233	DRAPDSSKDGRNHHKTMGHYNHNDSLRKT	Negative
2357.	Q5RL73:246	HKTMGHYNHNDSLRKTQINSLKNSVACPG	Negative
2358.	Q5RL73:253	NHNDSLRKTQINSLKNSVACPGAQKAITS	Negative
2359.	Q5RL73:263	INSLKNSVACPGAQKAITSSEAVDRFMPR	Negative
2360.	Q5RL73:285	VDRFMPRTTQLQERKRRREDDRKLGTFLQ	Negative
2361.	Q5RL73:293	TQLQERKRRREDDRKLGTFLQTNPTGNEI	Negative
2362.	Q5RL73:318	GNEIMIGPLLPDISKVDMHDDSLNTTANL	Negative
2363.	Q5RL73:336	HDDSLNTTANLIRHKLKEVISSVPKPPED	Negative
2364.	Q5RL73:338	DSLNTTANLIRHKLKEVISSVPKPPEDKP	Negative
2365.	Q5RL73:346	LIRHKLKEVISSVPKPPEDKPEDVHTSHP	Negative
2366.	Q5RL73:362	PEDKPEDVHTSHPLKQRRRIXXXXXXXXX	Negative
2367.	Q64FW2:37	LYAASSPNPFAEDVKRPPEPLVTDKEARK	Negative

2368.	Q64FW2:47	AEDVKRPPEPLVTDKEARKKVLKQAFSVS	Negative
2369.	Q64FW2:51	KRPPEPLVTDKEARKKVLKQAFSVSRVPE	Negative
2370.	Q64FW2:52	RPPEPLVTDKEARKKVLKQAFSVSRVPEK	Negative
2371.	Q64FW2:66	KVLKQAFSVSRVPEKLDAVVIGSGIGGLA	Negative
2372.	Q64FW2:87	GSGIGGLASAAVLAKAGKRVLVLEQHTKA	Negative
2373.	Q64FW2:90	IGGLASAAVLAKAGKRVLVLEQHTKAGGC	Negative
2374.	Q64FW2:100	AKAGKRVLVLEQHTKAGGCCHTFGENGLE	Negative
2375.	Q64FW2:164	SPFDLMILEGPNGRKEFPMYSGRKEYIQG	Negative
2376.	Q64FW2:173	GPNGRKEFPMYSGRKEYIQGLKKKFPKEE	Negative
2377.	Q64FW2:180	FPMYSGRKEYIQGLKKKFPKEEAVIDKYM	Negative
2378.	Q64FW2:181	PMYSGRKEYIQGLKKKFPKEEAVIDKYME	Negative
2379.	Q64FW2:182	MYSGRKEYIQGLKKKFPKEEAVIDKYMEL	Negative
2380.	Q64FW2:185	GRKEYIQGLKKKFPKEEAVIDKYMELVKV	Negative
2381.	Q64FW2:192	GLKKKFPKEEAVIDKYMELVKVVARGVSH	Negative
2382.	Q64FW2:198	PKEEAVIDKYMELVKVVARGVSHAVLLKF	Negative
2383.	Q64FW2:211	VKVVARGVSHAVLLKFLPLPLTQLLSKFG	Negative
2384.	Q64FW2:223	LLKFLPLPLTQLLSKFGLLTRFSPFCRAS	Negative
2385.	Q64FW2:336	VLLDSAGRACGVSVKKGQELVNIYCPVVI	Negative
2386.	Q64FW2:337	LLDSAGRACGVSVKKGQELVNIYCPVVIS	Negative
2387.	Q64FW2:374	QHLLPETVRHLPDVKKQLAMVRPGLSMLS	Negative
2388.	Q64FW2:375	HLLPETVRHLPDVKKQLAMVRPGLSMLSI	Negative
2389.	Q64FW2:394	VRPGLSMLSIFICLKGTKEDLKLQSTNYY	Negative
2390.	Q64FW2:397	GLSMLSIFICLKGTKEDLKLQSTNYYVYF	Negative
2391.	Q64FW2:401	LSIFICLKGTKEDLKLQSTNYYVYFDTDM	Negative
2392.	Q64FW2:417	QSTNYYVYFDTDMDKAMERYVSMPKEKAP	Negative
2393.	Q64FW2:427	TDMDKAMERYVSMPKEKAPEHIPLLFIAF	Negative
2394.	Q64FW2:429	MDKAMERYVSMPKEKAPEHIPLLFIAFPS	Negative
2395.	Q64FW2:445	PEHIPLLFIAFPSSKDPTWEERFPDRSTM	Negative
2396.	Q64FW2:478	PMAFEWFEEWQEEPKGKRGVDYETLKNAF	Negative
2397.	Q64FW2:480	AFEWFEEWQEEPKGKRGVDYETLKNAFVE	Negative
2398.	Q64FW2:489	EEPKGKRGVDYETLKNAFVEASMSVIMKL	Negative
2399.	Q64FW2:502	LKNAFVEASMSVIMKLFPQLEGKVESVTG	Negative
2400.	Q64FW2:510	SMSVIMKLFPQLEGKVESVTGGSPLTNQY	Negative
2401.	Q64FW2:588	GALQGALLCSSAILKRNLYSDLQALGSKV	Negative
2402.	Q64FW2:601	LKRNLYSDLQALGSKVKAQKKKMXXXXXX	Negative
2403.	Q64FW2:603	RNLYSDLQALGSKVKAQKKKMXXXXXXXX	Negative
2404.	Q64FW2:606	YSDLQALGSKVKAQKKKMXXXXXXXXXXX	Negative
2405.	Q64FW2:607	SDLQALGSKVKAQKKKMXXXXXXXXXXXX	Negative
2406.	Q64FW2:608	DLQALGSKVKAQKKKMXXXXXXXXXXXXX	Negative
2407.	Q6PKG0:27	ATLLQAEEHGGLVRKKPPPAPEGKGEPGP	Negative
2408.	Q6PKG0:28	TLLQAEEHGGLVRKKPPPAPEGKGEPGPN	Negative
2409.	Q6PKG0:36	GGLVRKKPPPAPEGKGEPGPNDVRGGEPD	Negative
2410.	Q6PKG0:62	EPDGSARRPRPPCAKPHKEGTGQQERESP	Negative
2411.	Q6PKG0:65	GSARRPRPPCAKPHKEGTGQQERESPRPL	Negative
2412.	Q6PKG0:123	AGAGRRDFVEAPPPKVNPWTKNALPPVLT	Negative
2413.	Q6PKG0:129	DFVEAPPPKVNPWTKNALPPVLTTVNGQS	Negative
2414.	Q6PKG0:152	TVNGQSPPEHSAPAKVVRAAVPKQRKGSK	Negative
2415.	Q6PKG0:160	EHSAPAKVVRAAVPKQRKGSKVGDFGDAI	Negative
2416.	Q6PKG0:163	APAKVVRAAVPKQRKGSKVGDFGDAINWP	Negative
2417.	Q6PKG0:166	KVVRAAVPKQRKGSKVGDFGDAINWPTPG	Negative
2418.	Q6PKG0:185	GDAINWPTPGEIAHKSVQPQSHKPQPTRK	Negative
2419.	Q6PKG0:193	PGEIAHKSVQPQSHKPQPTRKLPPKKDMK	Negative
2420.	Q6PKG0:199	KSVQPQSHKPQPTRKLPPKKDMKEQEKGE	Negative
2421.	Q6PKG0:203	PQSHKPQPTRKLPPKKDMKEQEKGEGSDS	Negative

2422.	Q6PKG0:204	QSHKPQPTRKLPPKKDMKEQEKGEGSDSK	Negative
2423.	Q6PKG0:207	KPQPTRKLPPKKDMKEQEKGEGSDSKESP	Negative
2424.	Q6PKG0:211	TRKLPPKKDMKEQEKGEGSDSKESPKTKS	Negative
2425.	Q6PKG0:218	KDMKEQEKGEGSDSKESPKTKSDESGEEK	Negative
2426.	Q6PKG0:222	EQEKGEGSDSKESPKTKSDESGEEKNGDE	Negative
2427.	Q6PKG0:224	EKGEGSDSKESPKTKSDESGEEKNGDEDC	Negative
2428.	Q6PKG0:232	KESPKTKSDESGEEKNGDEDCQRGGQKKK	Negative
2429.	Q6PKG0:244	EEKNGDEDCQRGGQKKKGNKHKWVPLQID	Negative
2430.	Q6PKG0:245	EKNGDEDCQRGGQKKKGNKHKWVPLQIDM	Negative
2431.	Q6PKG0:246	KNGDEDCQRGGQKKKGNKHKWVPLQIDMK	Negative
2432.	Q6PKG0:249	DEDCQRGGQKKKGNKHKWVPLQIDMKPEV	Negative
2433.	Q6PKG0:251	DCQRGGQKKKGNKHKWVPLQIDMKPEVPR	Negative
2434.	Q6PKG0:260	KGNKHKWVPLQIDMKPEVPREKLASRPTR	Negative
2435.	Q6PKG0:267	VPLQIDMKPEVPREKLASRPTRPPEPRHI	Negative
2436.	Q6PKG0:289	PPEPRHIPANRGEIKGSESATYVPVAPPT	Negative
2437.	Q6PKG0:311	VPVAPPTPAWQPEIKPEPAWHDQDETSSV	Negative
2438.	Q6PKG0:326	PEPAWHDQDETSSVKSDGAGGARASFRGR	Negative
2439.	Q6PKG0:367	GGTRTHFDYQFGYRKFDGVEGPRTPKYMN	Negative
2440.	Q6PKG0:378	GYRKFDGVEGPRTPKYMNNITYYFDNVSS	Negative
2441.	Q6PKG0:404	VSSTELYSVDQELLKDYIKRQIEYYFSVD	Negative
2442.	Q6PKG0:408	ELYSVDQELLKDYIKRQIEYYFSVDNLER	Negative
2443.	Q6PKG0:429	FSVDNLERDFFLRRKMDADGFLPITLIAS	Negative
2444.	Q6PKG0:462	QALTTDISLIFAALKDSKVVEIVDEKVRR	Negative
2445.	Q6PKG0:465	TTDISLIFAALKDSKVVEIVDEKVRRREE	Negative
2446.	Q6PKG0:473	AALKDSKVVEIVDEKVRRREEPEKWPLPP	Negative
2447.	Q6PKG0:482	EIVDEKVRRREEPEKWPLPPIVDYSQTDF	Negative
2448.	Q6PKG0:513	LLNCPEFVPRQHYQKETESAPGSPRAVTP	Negative
2449.	Q6PKG0:531	SAPGSPRAVTPVPTKTEEVSNLKTLPKGL	Negative
2450.	Q6PKG0:539	VTPVPTKTEEVSNLKTLPKGLSASLPDLD	Negative
2451.	Q6PKG0:543	PTKTEEVSNLKTLPKGLSASLPDLDSENW	Negative
2452.	Q6PKG0:561	ASLPDLDSENWIEVKKRPRPSPARPKKSE	Negative
2453.	Q6PKG0:562	SLPDLDSENWIEVKKRPRPSPARPKKSEE	Negative
2454.	Q6PKG0:572	IEVKKRPRPSPARPKKSEESRFSHLTSLP	Negative
2455.	Q6PKG0:573	EVKKRPRPSPARPKKSEESRFSHLTSLPQ	Negative
2456.	Q6PKG0:597	TSLPQQLPSQQLMSKDQDEQEELDFLFDE	Negative
2457.	Q6PKG0:620	DFLFDEEMEQMDGRKNTFTAWSDEESDYE	Negative
2458.	Q6PKG0:642	DEESDYEIDDRDVNKILIVTQTPHYMRRH	Negative
2459.	Q6PKG0:670	HPGGDRTGNHTSRAKMSAELAKVINDGLF	Negative
2460.	Q6PKG0:677	GNHTSRAKMSAELAKVINDGLFYYEQDLW	Negative
2461.	Q6PKG0:694	NDGLFYYEQDLWAEKFEPEYSQIKQEVEN	Negative
2462.	Q6PKG0:703	DLWAEKFEPEYSQIKQEVENFKKVNMISR	Negative
2463.	Q6PKG0:710	EPEYSQIKQEVENFKKVNMISREQFDTLT	Negative
2464.	Q6PKG0:711	PEYSQIKQEVENFKKVNMISREQFDTLTP	Negative
2465.	Q6PKG0:753	PPRFQQVPTDALANKLFGAPEPSTIARSL	Negative
2466.	Q6PKG0:792	RNTRTPRTPRTPQLKDSSQTSRFYPVVKE	Negative
2467.	Q6PKG0:805	LKDSSQTSRFYPVVKEGRTLDAKMPRKRK	Negative
2468.	Q6PKG0:813	RFYPVVKEGRTLDAKMPRKRKTRHSSNPP	Negative
2469.	Q6PKG0:817	VVKEGRTLDAKMPRKRKTRHSSNPPLESH	Negative
2470.	Q6PKG0:819	KEGRTLDAKMPRKRKTRHSSNPPLESHVG	Negative
2471.	Q6PKG0:871	PTVGSYGCTPQSLPKFQHPSHELLKENGF	Negative
2472.	Q6PKG0:881	QSLPKFQHPSHELLKENGFTQHVYHKYRR	Negative
2473.	Q6PKG0:892	ELLKENGFTQHVYHKYRRRCLNERKRLGI	Negative
2474.	Q6PKG0:902	HVYHKYRRRCLNERKRLGIGQSQEMNTLF	Negative
2475.	Q6PKG0:929	LFRFWSFFLRDHFNKKMYEEFKQLALEDA	Negative

2476.	Q6PKG0:930	FRFWSFFLRDHFNKKMYEEFKQLALEDAK	Negative
2477.	Q6PKG0:936	FLRDHFNKKMYEEFKQLALEDAKEGYRYG	Negative
2478.	Q6PKG0:944	KMYEEFKQLALEDAKEGYRYGLECLFRYY	Negative
2479.	Q6PKG0:964	GLECLFRYYSYGLEKKFRLDIFKDFQEET	Negative
2480.	Q6PKG0:965	LECLFRYYSYGLEKKFRLDIFKDFQEETV	Negative
2481.	Q6PKG0:972	YSYGLEKKFRLDIFKDFQEETVKDYEAGQ	Negative
2482.	Q6PKG0:980	FRLDIFKDFQEETVKDYEAGQLYGLEKFW	Negative
2483.	Q6PKG0:992	TVKDYEAGQLYGLEKFWAFLKYSKAKNLD	Negative
2484.	Q6PKG0:998	AGQLYGLEKFWAFLKYSKAKNLDIDPKLQ	Negative
2485.	Q6PKG0:1001	LYGLEKFWAFLKYSKAKNLDIDPKLQEYL	Negative
2486.	Q6PKG0:1003	GLEKFWAFLKYSKAKNLDIDPKLQEYLGK	Negative
2487.	Q6PKG0:1010	FLKYSKAKNLDIDPKLQEYLGKFRRLEDF	Negative
2488.	Q6PKG0:1017	KNLDIDPKLQEYLGKFRRLEDFRVDPPMG	Negative
2489.	Q6PKG0:1037	DFRVDPPMGEEGNHKRHSVVAGGGGGEGR	Negative
2490.	Q6PKG0:1052	RHSVVAGGGGGEGRKRCPSQSSSRPAAMI	Negative
2491.	Q6PKG0:1096	AKWTSQHSNTQTLGKXXXXXXXXXXXXXX	Negative
2492.	Q80ZV3:49	LPAIQPRLVAVSKTKPADMVIEAYGHGQR	Negative
2493.	Q80ZV3:78	TFGENYILSSCPEIKWHFIGHLQKQNVNK	Negative
2494.	Q80ZV3:87	SCPEIKWHFIGHLQKQNVNKLMAVPNLSM	Negative
2495.	Q80ZV3:92	KWHFIGHLQKQNVNKLMAVPNLSMLETVD	Negative
2496.	Q80ZV3:109	AVPNLSMLETVDSVKLADKVNSSWQKKGP	Negative
2497.	Q80ZV3:113	LSMLETVDSVKLADKVNSSWQKKGPTEPL	Negative
2498.	Q80ZV3:120	DSVKLADKVNSSWQKKGPTEPLKVMVQIN	Negative
2499.	Q80ZV3:121	SVKLADKVNSSWQKKGPTEPLKVMVQINT	Negative
2500.	Q80ZV3:128	VNSSWQKKGPTEPLKVMVQINTSGEDSKX	Negative
2501.	Q80ZV3:141	LKVMVQINTSGEDSKXXXXXXXXXXXXXX	Negative
2502.	Q86UP2:30	IVITVIFLFFWLFMKETLYDEVLAKQKRE	Negative
2503.	Q86UP2:40	WLFMKETLYDEVLAKQKREQKLIPTKTDK	Negative
2504.	Q86UP2:42	FMKETLYDEVLAKQKREQKLIPTKTDKKK	Negative
2505.	Q86UP2:46	TLYDEVLAKQKREQKLIPTKTDKKKAEKK	Negative
2506.	Q86UP2:51	VLAKQKREQKLIPTKTDKKKAEKKKNKKK	Negative
2507.	Q86UP2:54	KQKREQKLIPTKTDKKKAEKKKNKKKEIQ	Negative
2508.	Q86UP2:55	QKREQKLIPTKTDKKKAEKKKNKKKEIQN	Negative
2509.	Q86UP2:56	KREQKLIPTKTDKKKAEKKKNKKKEIQNG	Negative
2510.	Q86UP2:59	QKLIPTKTDKKKAEKKKNKKKEIQNGNLH	Negative
2511.	Q86UP2:60	KLIPTKTDKKKAEKKKNKKKEIQNGNLHE	Negative
2512.	Q86UP2:61	LIPTKTDKKKAEKKKNKKKEIQNGNLHES	Negative
2513.	Q86UP2:63	PTKTDKKKAEKKKNKKKEIQNGNLHESDS	Negative
2514.	Q86UP2:64	TKTDKKKAEKKKNKKKEIQNGNLHESDSE	Negative
2515.	Q86UP2:65	KTDKKKAEKKKNKKKEIQNGNLHESDSES	Negative
2516.	Q86UP2:85	NLHESDSESVPRDFKLSDALAVEDDQVAP	Negative
2517.	Q86UP2:115	PLNVVETSSSVRERKKKEKKQKPVLEEQV	Negative
2518.	Q86UP2:116	LNVVETSSSVRERKKKEKKQKPVLEEQVI	Negative
2519.	Q86UP2:117	NVVETSSSVRERKKKEKKQKPVLEEQVIK	Negative
2520.	Q86UP2:119	VETSSSVRERKKKEKKQKPVLEEQVIKES	Negative
2521.	Q86UP2:120	ETSSSVRERKKKEKKQKPVLEEQVIKESD	Negative
2522.	Q86UP2:122	SSSVRERKKKEKKQKPVLEEQVIKESDAS	Negative
2523.	Q86UP2:131	KEKKQKPVLEEQVIKESDASKIPGKKVEP	Negative
2524.	Q86UP2:137	PVLEEQVIKESDASKIPGKKVEPVPVTKQ	Negative
2525.	Q86UP2:141	EQVIKESDASKIPGKKVEPVPVTKQPTPP	Negative
2526.	Q86UP2:142	QVIKESDASKIPGKKVEPVPVTKQPTPPS	Negative
2527.	Q86UP2:150	SKIPGKKVEPVPVTKQPTPPSEAAASKKK	Negative
2528.	Q86UP2:162	VTKQPTPPSEAAASKKKPGQKKSKNGSDD	Negative

2529.	Q86UP2:163	TKQPTPPSEAAASKKKPGQKKSKNGSDDQ	Negative
2530.	Q86UP2:164	KQPTPPSEAAASKKKPGQKKSKNGSDDQD	Negative
2531.	Q86UP2:168	PPSEAAASKKKPGQKKSKNGSDDQDKKVE	Negative
2532.	Q86UP2:169	PSEAAASKKKPGQKKSKNGSDDQDKKVET	Negative
2533.	Q86UP2:171	EAAASKKKPGQKKSKNGSDDQDKKVETLM	Negative
2534.	Q86UP2:179	PGQKKSKNGSDDQDKKVETLMVPSKRQEA	Negative
2535.	Q86UP2:180	GQKKSKNGSDDQDKKVETLMVPSKRQEAL	Negative
2536.	Q86UP2:189	DDQDKKVETLMVPSKRQEALPLHQETKQE	Negative
2537.	Q86UP2:201	PSKRQEALPLHQETKQESGSGKKKASSKK	Negative
2538.	Q86UP2:208	LPLHQETKQESGSGKKKASSKKQKTENVF	Negative
2539.	Q86UP2:209	PLHQETKQESGSGKKKASSKKQKTENVFV	Negative
2540.	Q86UP2:210	LHQETKQESGSGKKKASSKKQKTENVFVD	Negative
2541.	Q86UP2:214	TKQESGSGKKKASSKKQKTENVFVDEPLI	Negative
2542.	Q86UP2:215	KQESGSGKKKASSKKQKTENVFVDEPLIH	Negative
2543.	Q86UP2:217	ESGSGKKKASSKKQKTENVFVDEPLIHAT	Negative
2544.	Q86UP2:248	IPLMDNADSSPVVDKREVIDLLKPDQVEG	Negative
2545.	Q86UP2:256	SSPVVDKREVIDLLKPDQVEGIQKSGTKK	Negative
2546.	Q86UP2:265	VIDLLKPDQVEGIQKSGTKKLKTETDKEN	Negative
2547.	Q86UP2:269	LKPDQVEGIQKSGTKKLKTETDKENAEVK	Negative
2548.	Q86UP2:270	KPDQVEGIQKSGTKKLKTETDKENAEVKF	Negative
2549.	Q86UP2:272	DQVEGIQKSGTKKLKTETDKENAEVKFKD	Negative
2550.	Q86UP2:277	IQKSGTKKLKTETDKENAEVKFKDFLLSL	Negative
2551.	Q86UP2:283	KKLKTETDKENAEVKFKDFLLSLKTMMFS	Negative
2552.	Q86UP2:285	LKTETDKENAEVKFKDFLLSLKTMMFSED	Negative
2553.	Q86UP2:309	MFSEDEALCVVDLLKEKSGVIQDALKKSS	Negative
2554.	Q86UP2:311	SEDEALCVVDLLKEKSGVIQDALKKSSKG	Negative
2555.	Q86UP2:320	DLLKEKSGVIQDALKKSSKGELTTLIHQL	Negative
2556.	Q86UP2:321	LLKEKSGVIQDALKKSSKGELTTLIHQLQ	Negative
2557.	Q86UP2:324	EKSGVIQDALKKSSKGELTTLIHQLQEKD	Negative
2558.	Q86UP2:337	SKGELTTLIHQLQEKDKLLAAVKEDAAAT	Negative
2559.	Q86UP2:339	GELTTLIHQLQEKDKLLAAVKEDAAATKD	Negative
2560.	Q86UP2:345	IHQLQEKDKLLAAVKEDAAATKDRCKQLT	Negative
2561.	Q86UP2:352	DKLLAAVKEDAAATKDRCKQLTQEMMTEK	Negative
2562.	Q86UP2:356	AAVKEDAAATKDRCKQLTQEMMTEKERSN	Negative
2563.	Q86UP2:366	KDRCKQLTQEMMTEKERSNVVITRMKDRI	Negative
2564.	Q86UP2:377	MTEKERSNVVITRMKDRIGTLEKEHNVFQ	Negative
2565.	Q86UP2:385	VVITRMKDRIGTLEKEHNVFQNKIHVSYQ	Negative
2566.	Q86UP2:393	RIGTLEKEHNVFQNKIHVSYQETQQMQMK	Negative
2567.	Q86UP2:407	KIHVSYQETQQMQMKFQQVREQMEAEIAH	Negative
2568.	Q86UP2:423	QQVREQMEAEIAHLKQENGILRDAVSNTT	Negative
2569.	Q86UP2:443	LRDAVSNTTNQLESKQSAELNKLRQDYAR	Negative
2570.	Q86UP2:450	TTNQLESKQSAELNKLRQDYARLVNELTE	Negative
2571.	Q86UP2:465	LRQDYARLVNELTEKTGKLQQEEVQKKNA	Negative
2572.	Q86UP2:468	DYARLVNELTEKTGKLQQEEVQKKNAEQA	Negative
2573.	Q86UP2:476	LTEKTGKLQQEEVQKKNAEQAATQLKVQL	Negative
2574.	Q86UP2:477	TEKTGKLQQEEVQKKNAEQAATQLKVQLQ	Negative
2575.	Q86UP2:487	EVQKKNAEQAATQLKVQLQEAERRWEEVQ	Negative
2576.	Q86UP2:506	EAERRWEEVQSYIRKRTAEHEAAQQDLQS	Negative
2577.	Q86UP2:521	RTAEHEAAQQDLQSKFVAKENEVQSLHSK	Negative
2578.	Q86UP2:525	HEAAQQDLQSKFVAKENEVQSLHSKLTDT	Negative
2579.	Q86UP2:535	KFVAKENEVQSLHSKLTDTLVSKQQLEQR	Negative
2580.	Q86UP2:543	VQSLHSKLTDTLVSKQQLEQRLMQLMESE	Negative
2581.	Q86UP2:559	QLEQRLMQLMESEQKRVNKEESLQMQVQD	Negative
2582.	Q86UP2:563	RLMQLMESEQKRVNKEESLQMQVQDILEQ	Negative

2583.	Q86UP2:582	QMQVQDILEQNEALKAQIQQFHSQIAAQT	Negative
2584.	Q86UP2:607	AAQTSASVLAEELHKVIAEKDKQIKQTED	Negative
2585.	Q86UP2:612	ASVLAEELHKVIAEKDKQIKQTEDSLASE	Negative
2586.	Q86UP2:614	VLAEELHKVIAEKDKQIKQTEDSLASERD	Negative
2587.	Q86UP2:617	EELHKVIAEKDKQIKQTEDSLASERDRLT	Negative
2588.	Q86UP2:633	TEDSLASERDRLTSKEEELKDIQNMNFLL	Negative
2589.	Q86UP2:638	ASERDRLTSKEEELKDIQNMNFLLKAEVQ	Negative
2590.	Q86UP2:648	EEELKDIQNMNFLLKAEVQKLQALANEQA	Negative
2591.	Q86UP2:653	DIQNMNFLLKAEVQKLQALANEQAAAAHE	Negative
2592.	Q86UP2:670	ALANEQAAAAHELEKMQQSVYVKDDKIRL	Negative
2593.	Q86UP2:678	AAHELEKMQQSVYVKDDKIRLLEEQLQHE	Negative
2594.	Q86UP2:681	ELEKMQQSVYVKDDKIRLLEEQLQHEISN	Negative
2595.	Q86UP2:696	IRLLEEQLQHEISNKMEEFKILNDQNKAL	Negative
2596.	Q86UP2:701	EQLQHEISNKMEEFKILNDQNKALKSEVQ	Negative
2597.	Q86UP2:708	SNKMEEFKILNDQNKALKSEVQKLQTLVS	Negative
2598.	Q86UP2:711	MEEFKILNDQNKALKSEVQKLQTLVSEQP	Negative
2599.	Q86UP2:716	ILNDQNKALKSEVQKLQTLVSEQPNKDVV	Negative
2600.	Q86UP2:727	EVQKLQTLVSEQPNKDVVEQMEKCIQEKD	Negative
2601.	Q86UP2:735	VSEQPNKDVVEQMEKCIQEKDEKLKTVEE	Negative
2602.	Q86UP2:740	NKDVVEQMEKCIQEKDEKLKTVEELLETG	Negative
2603.	Q86UP2:743	VVEQMEKCIQEKDEKLKTVEELLETGLIQ	Negative
2604.	Q86UP2:745	EQMEKCIQEKDEKLKTVEELLETGLIQVA	Negative
2605.	Q86UP2:761	VEELLETGLIQVATKEEELNAIRTENSSL	Negative
2606.	Q86UP2:777	EELNAIRTENSSLTKEVQDLKAKQNDQVS	Negative
2607.	Q86UP2:783	RTENSSLTKEVQDLKAKQNDQVSFASLVE	Negative
2608.	Q86UP2:785	ENSSLTKEVQDLKAKQNDQVSFASLVEEL	Negative
2609.	Q86UP2:800	QNDQVSFASLVEELKKVIHEKDGKIKSVE	Negative
2610.	Q86UP2:801	NDQVSFASLVEELKKVIHEKDGKIKSVEE	Negative
2611.	Q86UP2:806	FASLVEELKKVIHEKDGKIKSVEELLEAE	Negative
2612.	Q86UP2:809	LVEELKKVIHEKDGKIKSVEELLEAELLK	Negative
2613.	Q86UP2:811	EELKKVIHEKDGKIKSVEELLEAELLKVA	Negative
2614.	Q86UP2:823	KIKSVEELLEAELLKVANKEKTVQDLKQE	Negative
2615.	Q86UP2:827	VEELLEAELLKVANKEKTVQDLKQEIKAL	Negative
2616.	Q86UP2:829	ELLEAELLKVANKEKTVQDLKQEIKALKE	Negative
2617.	Q86UP2:835	LLKVANKEKTVQDLKQEIKALKEEIGNVQ	Negative
2618.	Q86UP2:839	ANKEKTVQDLKQEIKALKEEIGNVQLEKA	Negative
2619.	Q86UP2:842	EKTVQDLKQEIKALKEEIGNVQLEKAQQL	Negative
2620.	Q86UP2:852	IKALKEEIGNVQLEKAQQLSITSKVQELQ	Negative
2621.	Q86UP2:861	NVQLEKAQQLSITSKVQELQNLLKGKEEQ	Negative
2622.	Q86UP2:870	LSITSKVQELQNLLKGKEEQMNTMKAVLE	Negative
2623.	Q86UP2:872	ITSKVQELQNLLKGKEEQMNTMKAVLEEK	Negative
2624.	Q86UP2:880	QNLLKGKEEQMNTMKAVLEEKEKDLANTG	Negative
2625.	Q86UP2:886	KEEQMNTMKAVLEEKEKDLANTGKWLQDL	Negative
2626.	Q86UP2:888	EQMNTMKAVLEEKEKDLANTGKWLQDLQE	Negative
2627.	Q86UP2:895	AVLEEKEKDLANTGKWLQDLQEENESLKA	Negative
2628.	Q86UP2:908	GKWLQDLQEENESLKAHVQEVAQHNLKEA	Negative
2629.	Q86UP2:920	SLKAHVQEVAQHNLKEASSASQFEELEIV	Negative
2630.	Q86UP2:936	ASSASQFEELEIVLKEKENELKRLEAMLK	Negative
2631.	Q86UP2:938	SASQFEELEIVLKEKENELKRLEAMLKER	Negative
2632.	Q86UP2:943	EELEIVLKEKENELKRLEAMLKERESDLS	Negative
2633.	Q86UP2:950	KEKENELKRLEAMLKERESDLSSKTQLLQ	Negative
2634.	Q86UP2:959	LEAMLKERESDLSSKTQLLQDVQDENKLF	Negative
2635.	Q86UP2:971	SSKTQLLQDVQDENKLFKSQIEQLKQQNY	Negative
2636.	Q86UP2:974	TQLLQDVQDENKLFKSQIEQLKQQNYQQA	Negative

2637.	Q86UP2:981	QDENKLFKSQIEQLKQQNYQQASSFPPHE	Negative
2638.	Q86UP2:999	YQQASSFPPHEELLKVISEREKEISGLWN	Negative
2639.	Q86UP2:1006	PPHEELLKVISEREKEISGLWNELDSLKD	Negative
2640.	Q86UP2:1019	EKEISGLWNELDSLKDAVEHQRKKNNDLR	Negative
2641.	Q86UP2:1027	NELDSLKDAVEHQRKKNNDLREKNWEAME	Negative
2642.	Q86UP2:1028	ELDSLKDAVEHQRKKNNDLREKNWEAMEA	Negative
2643.	Q86UP2:1035	AVEHQRKKNNDLREKNWEAMEALASTEKM	Negative
2644.	Q86UP2:1048	EKNWEAMEALASTEKMLQDKVNKTSKERQ	Negative
2645.	Q86UP2:1053	AMEALASTEKMLQDKVNKTSKERQQQVEA	Negative
2646.	Q86UP2:1056	ALASTEKMLQDKVNKTSKERQQQVEAVEL	Negative
2647.	Q86UP2:1059	STEKMLQDKVNKTSKERQQQVEAVELEAK	Negative
2648.	Q86UP2:1073	KERQQQVEAVELEAKEVLKKLFPKVSVPS	Negative
2649.	Q86UP2:1077	QQVEAVELEAKEVLKKLFPKVSVPSNLSY	Negative
2650.	Q86UP2:1078	QVEAVELEAKEVLKKLFPKVSVPSNLSYG	Negative
2651.	Q86UP2:1082	VELEAKEVLKKLFPKVSVPSNLSYGEWLH	Negative
2652.	Q86UP2:1100	PSNLSYGEWLHGFEKKAKECMAGTSGSEE	Negative
2653.	Q86UP2:1101	SNLSYGEWLHGFEKKAKECMAGTSGSEEV	Negative
2654.	Q86UP2:1103	LSYGEWLHGFEKKAKECMAGTSGSEEVKV	Negative
2655.	Q86UP2:1116	AKECMAGTSGSEEVKVLEHKLKEADEMHT	Negative
2656.	Q86UP2:1121	AGTSGSEEVKVLEHKLKEADEMHTLLQLE	Negative
2657.	Q86UP2:1123	TSGSEEVKVLEHKLKEADEMHTLLQLECE	Negative
2658.	Q86UP2:1138	EADEMHTLLQLECEKYKSVLAETEGILQK	Negative
2659.	Q86UP2:1140	DEMHTLLQLECEKYKSVLAETEGILQKLQ	Negative
2660.	Q86UP2:1152	KYKSVLAETEGILQKLQRSVEQEENKWKV	Negative
2661.	Q86UP2:1163	ILQKLQRSVEQEENKWKVKVDESHKTIKQ	Negative
2662.	Q86UP2:1165	QKLQRSVEQEENKWKVKVDESHKTIKQMQ	Negative
2663.	Q86UP2:1167	LQRSVEQEENKWKVKVDESHKTIKQMQSS	Negative
2664.	Q86UP2:1173	QEENKWKVKVDESHKTIKQMQSSFTSSEQ	Negative
2665.	Q86UP2:1176	NKWKVKVDESHKTIKQMQSSFTSSEQELE	Negative
2666.	Q86UP2:1197	TSSEQELERLRSENKDIENLRREREHLEM	Negative
2667.	Q86UP2:1215	NLRREREHLEMELEKAEMERSTYVTEVRE	Negative
2668.	Q86UP2:1231	EMERSTYVTEVRELKDLLTELQKKLDDSY	Negative
2669.	Q86UP2:1239	TEVRELKDLLTELQKKLDDSYSEAVRQNE	Negative
2670.	Q86UP2:1240	EVRELKDLLTELQKKLDDSYSEAVRQNEE	Negative
2671.	Q86UP2:1259	YSEAVRQNEELNLLKAQLNETLTKLRTEQ	Negative
2672.	Q86UP2:1268	ELNLLKAQLNETLTKLRTEQNERQKVAGD	Negative
2673.	Q86UP2:1278	ETLTKLRTEQNERQKVAGDLHKAQQSLEL	Negative
2674.	Q86UP2:1285	TEQNERQKVAGDLHKAQQSLELIQSKIVK	Negative
2675.	Q86UP2:1296	DLHKAQQSLELIQSKIVKAAGDTTVIENS	Negative
2676.	Q86UP2:1299	KAQQSLELIQSKIVKAAGDTTVIENSDVS	Negative
2677.	Q86UP2:1321	IENSDVSPETESSEKETMSVSLNQTVTQL	Negative
2678.	Q86UP2:1348	QLQQLLQAVNQQLTKEKEHYQVLEXXXXX	Negative
2679.	Q86UP2:1350	QQLLQAVNQQLTKEKEHYQVLEXXXXXXX	Negative
2680.	Q8BH04:85	TAILALLEEQGLIRKLPKYKNCWLARTDP	Negative
2681.	Q8BH04:88	LALLEEQGLIRKLPKYKNCWLARTDPKDV	Negative
2682.	Q8BH04:90	LLEEQGLIRKLPKYKNCWLARTDPKDVAR	Negative
2683.	Q8BH04:100	LPKYKNCWLARTDPKDVARVESKTVIVTP	Negative
2684.	Q8BH04:108	LARTDPKDVARVESKTVIVTPSQRDTVPL	Negative
2685.	Q8BH04:209	GTPVLQALGDGDFIKCLHSVGQPLTGHGD	Negative
2686.	Q8BH04:234	GHGDPVGQWPCNPEKTLIGHVPDQREIVS	Negative
2687.	Q8BH04:261	VSFGSGYGGNSLLGKKCFALRIASRLARD	Negative
2688.	Q8BH04:294	AEHMLILGITNPAGKKRYVAAAFPSACGK	Negative
2689.	Q8BH04:295	EHMLILGITNPAGKKRYVAAAFPSACGKT	Negative
2690.	Q8BH04:308	KKRYVAAAFPSACGKTNLAMMRPALPGWK	Negative

2691.	Q8BH04:322	KTNLAMMRPALPGWKVECVGDDIAWMRFD	Negative
2692.	Q8BH04:407	QPLPPGVTITSWLGKPWKPGDKEPCAHPN	Negative
2693.	Q8BH04:410	PPGVTITSWLGKPWKPGDKEPCAHPNSRF	Negative
2694.	Q8BH04:414	TITSWLGKPWKPGDKEPCAHPNSRFCVPA	Negative
2695.	Q8BH04:457	GVPIDAIIFGGRRPKGVPLVYEAFNWRHG	Negative
2696.	Q8BH04:489	GSAMRSESTAAAEHKGKTIMHDPFAMRPF	Negative
2697.	Q8BH04:491	AMRSESTAAAEHKGKTIMHDPFAMRPFFG	Negative
2698.	Q8BH04:522	FGRYLEHWLSMEGQKGARLPRIFHVNWFR	Negative
2699.	Q8BH04:578	GEDSAQETPIGLVPKEGALDLSGLSAVDT	Negative
2700.	Q8BH04:600	GLSAVDTSQLFSIPKDFWEQEVRDIRGYL	Negative
2701.	Q8BH04:624	IRGYLTEQVNQDLPKEVLAELEALEGRVQ	Negative
2702.	Q8BH04:639	EVLAELEALEGRVQKMXXXXXXXXXXXXX	Negative
2703.	Q8BMS1:46	SSALLTRTHINYGVKGDVAVIRINSPNSK	Negative
2704.	Q8BMS1:60	KGDVAVIRINSPNSKVNTLNKEVQSEFIE	Negative
2705.	Q8BMS1:66	IRINSPNSKVNTLNKEVQSEFIEVMNEIW	Negative
2706.	Q8BMS1:94	WANDQIRSAVLISSKPGCFVAGADINMLS	Negative
2707.	Q8BMS1:129	EATRISQEGQRMFEKLEKSPKPVVAAISG	Negative
2708.	Q8BMS1:132	RISQEGQRMFEKLEKSPKPVVAAISGSCL	Negative
2709.	Q8BMS1:135	QEGQRMFEKLEKSPKPVVAAISGSCLGGG	Negative
2710.	Q8BMS1:163	GLELAIACQYRIATKDRKTVLGVPEVLLG	Negative
2711.	Q8BMS1:166	LAIACQYRIATKDRKTVLGVPEVLLGILP	Negative
2712.	Q8BMS1:190	LGILPGAGGTQRLPKMVGVPAAFDMMLTG	Negative
2713.	Q8BMS1:213	DMMLTGRNIRADRAKKMGLVDQLVEPLGP	Negative
2714.	Q8BMS1:230	GLVDQLVEPLGPGIKSPEERTIEYLEEVA	Negative
2715.	Q8BMS1:249	RTIEYLEEVAVNFAKGLADRKVSAKQSKG	Negative
2716.	Q8BMS1:255	EEVAVNFAKGLADRKVSAKQSKGLVEKLT	Negative
2717.	Q8BMS1:259	VNFAKGLADRKVSAKQSKGLVEKLTTYAM	Negative
2718.	Q8BMS1:262	AKGLADRKVSAKQSKGLVEKLTTYAMTVP	Negative
2719.	Q8BMS1:267	DRKVSAKQSKGLVEKLTTYAMTVPFVRQQ	Negative
2720.	Q8BMS1:284	TYAMTVPFVRQQVYKTVEEKVKKQTKGLY	Negative
2721.	Q8BMS1:289	VPFVRQQVYKTVEEKVKKQTKGLYPAPLK	Negative
2722.	Q8BMS1:291	FVRQQVYKTVEEKVKKQTKGLYPAPLKII	Negative
2723.	Q8BMS1:292	VRQQVYKTVEEKVKKQTKGLYPAPLKIID	Negative
2724.	Q8BMS1:295	QVYKTVEEKVKKQTKGLYPAPLKIIDAVK	Negative
2725.	Q8BMS1:303	KVKKQTKGLYPAPLKIIDAVKAGLEQGSD	Negative
2726.	Q8BMS1:309	KGLYPAPLKIIDAVKAGLEQGSDAGYLAE	Negative
2727.	Q8BMS1:326	LEQGSDAGYLAESQKFGELALTKESKALM	Negative
2728.	Q8BMS1:334	YLAESQKFGELALTKESKALMGLYNGQVL	Negative
2729.	Q8BMS1:337	ESQKFGELALTKESKALMGLYNGQVLCKK	Negative
2730.	Q8BMS1:350	SKALMGLYNGQVLCKKNKFGAPQKNVQQL	Negative
2731.	Q8BMS1:351	KALMGLYNGQVLCKKNKFGAPQKNVQQLA	Negative
2732.	Q8BMS1:353	LMGLYNGQVLCKKNKFGAPQKNVQQLAIL	Negative
2733.	Q8BMS1:359	GQVLCKKNKFGAPQKNVQQLAILGAGLMG	Negative
2734.	Q8BMS1:383	AGLMGAGIAQVSVDKGLKTLLKDTTVTGL	Negative
2735.	Q8BMS1:386	MGAGIAQVSVDKGLKTLLKDTTVTGLGRG	Negative
2736.	Q8BMS1:390	IAQVSVDKGLKTLLKDTTVTGLGRGQQQV	Negative
2737.	Q8BMS1:406	TTVTGLGRGQQQVFKGLNDKVKKKALTSF	Negative
2738.	Q8BMS1:411	LGRGQQQVFKGLNDKVKKKALTSFERDSI	Negative
2739.	Q8BMS1:413	RGQQQVFKGLNDKVKKKALTSFERDSIFS	Negative
2740.	Q8BMS1:414	GQQQVFKGLNDKVKKKALTSFERDSIFSN	Negative
2741.	Q8BMS1:415	QQQVFKGLNDKVKKKALTSFERDSIFSNL	Negative
2742.	Q8BMS1:436	RDSIFSNLIGQLDYKGFEKADMVIEAVFE	Negative
2743.	Q8BMS1:440	FSNLIGQLDYKGFEKADMVIEAVFEDLGV	Negative
2744.	Q8BMS1:455	ADMVIEAVFEDLGVKHKVLKEVESVTPEH	Negative

2745.	Q8BMS1:457	MVIEAVFEDLGVKHKVLKEVESVTPEHCI	Negative
2746.	Q8BMS1:460	EAVFEDLGVKHKVLKEVESVTPEHCIFAS	Negative
2747.	Q8BMS1:489	NTSALPINQIAAVSKRPEKVIGMHYFSPV	Negative
2748.	Q8BMS1:493	LPINQIAAVSKRPEKVIGMHYFSPVDKMQ	Negative
2749.	Q8BMS1:505	PEKVIGMHYFSPVDKMQLLEIITTDKTSK	Negative
2750.	Q8BMS1:516	PVDKMQLLEIITTDKTSKDTTASAVAVGL	Negative
2751.	Q8BMS1:519	KMQLLEIITTDKTSKDTTASAVAVGLRQG	Negative
2752.	Q8BMS1:534	DTTASAVAVGLRQGKVIIVVKDGPGFYTT	Negative
2753.	Q8BMS1:540	VAVGLRQGKVIIVVKDGPGFYTTRCLAPM	Negative
2754.	Q8BMS1:569	MSEVMRILQEGVDPKKLDALTTGFGFPVG	Negative
2755.	Q8BMS1:570	SEVMRILQEGVDPKKLDALTTGFGFPVGA	Negative
2756.	Q8BMS1:605	VGVDVAQHVAEDLGKAFGERFGGGSVELL	Negative
2757.	Q8BMS1:620	AFGERFGGGSVELLKQMVSKGFLGRKSGK	Negative
2758.	Q8BMS1:625	FGGGSVELLKQMVSKGFLGRKSGKGFYIY	Negative
2759.	Q8BMS1:631	ELLKQMVSKGFLGRKSGKGFYIYQEGSKN	Negative
2760.	Q8BMS1:634	KQMVSKGFLGRKSGKGFYIYQEGSKNKSL	Negative
2761.	Q8BMS1:644	RKSGKGFYIYQEGSKNKSLNSEMDNILAN	Negative
2762.	Q8BMS1:646	SGKGFYIYQEGSKNKSLNSEMDNILANLR	Negative
2763.	Q8BMS1:664	SEMDNILANLRLPAKPEVSSDEDVQYRVI	Negative
2764.	Q8BMS1:728	LGGPFRFVDLYGAQKVVDRLRKYESAYGT	Negative
2765.	Q8BMS1:735	VDLYGAQKVVDRLRKYESAYGTQFTPCQL	Negative
2766.	Q8BMS1:759	TPCQLLLDHANNSSKKFYQXXXXXXXXXX	Negative
2767.	Q8BMS1:760	PCQLLLDHANNSSKKFYQXXXXXXXXXXX	Negative
2768.	Q8C196:9	XXXXXXMTRILTACKVVKTLKSGFGFANV	Negative
2769.	Q8C196:12	XXXMTRILTACKVVKTLKSGFGFANVTTK	Negative
2770.	Q8C196:15	MTRILTACKVVKTLKSGFGFANVTTKRQW	Negative
2771.	Q8C196:26	KTLKSGFGFANVTTKRQWDFSRPGIRLLS	Negative
2772.	Q8C196:42	QWDFSRPGIRLLSVKAKTAHIVLEDGTKM	Negative
2773.	Q8C196:44	DFSRPGIRLLSVKAKTAHIVLEDGTKMKG	Negative
2774.	Q8C196:55	VKAKTAHIVLEDGTKMKGYSFGHPSSVAG	Negative
2775.	Q8C196:57	AKTAHIVLEDGTKMKGYSFGHPSSVAGEV	Negative
2776.	Q8C196:90	GLGGYPEALTDPAYKGQILTMANPIIGNG	Negative
2777.	Q8C196:119	GAPDTTARDELGLNKYMESDGIKVAGLLV	Negative
2778.	Q8C196:127	DELGLNKYMESDGIKVAGLLVLNYSNDYN	Negative
2779.	Q8C196:147	VLNYSNDYNHWLATKSLGQWLQEEKVPAI	Negative
2780.	Q8C196:157	WLATKSLGQWLQEEKVPAIYGVDTRMLTK	Negative
2781.	Q8C196:171	KVPAIYGVDTRMLTKIIRDKGTMLGKIEF	Negative
2782.	Q8C196:176	YGVDTRMLTKIIRDKGTMLGKIEFEGQSV	Negative
2783.	Q8C196:182	MLTKIIRDKGTMLGKIEFEGQSVDFVDPN	Negative
2784.	Q8C196:197	IEFEGQSVDFVDPNKQNLIAEVSTKDVKV	Negative
2785.	Q8C196:207	VDPNKQNLIAEVSTKDVKVFGKGNPTKVV	Negative
2786.	Q8C196:210	NKQNLIAEVSTKDVKVFGKGNPTKVVAVD	Negative
2787.	Q8C196:219	STKDVKVFGKGNPTKVVAVDCGIKNNVIR	Negative
2788.	Q8C196:237	VDCGIKNNVIRLLVKRGAEVHLVPWNHDF	Negative
2789.	Q8C196:279	PGNPALAQPLIQNVKKILESDRKEPLFGI	Negative
2790.	Q8C196:280	GNPALAQPLIQNVKKILESDRKEPLFGIS	Negative
2791.	Q8C196:287	PLIQNVKKILESDRKEPLFGISTGNIITG	Negative
2792.	Q8C196:307	ISTGNIITGLAAGAKSYKMSMANRGQNQP	Negative
2793.	Q8C196:310	GNIITGLAAGAKSYKMSMANRGQNQPVLN	Negative
2794.	Q8C196:350	NHGYALDNTLPAGWKPLFVNVNDQTNEGI	Negative
2795.	Q8C196:369	NVNDQTNEGIMHESKPFFAVQFHPEVSPG	Negative
2796.	Q8C196:399	TDTEYLFDSFFSLIKKGKGTTITSVLPKP	Negative
2797.	Q8C196:400	DTEYLFDSFFSLIKKGKGTTITSVLPKPA	Negative
2798.	Q8C196:402	EYLFDSFFSLIKKGKGTTITSVLPKPALV	Negative

2799.	Q8C196:412	IKKGKGTTITSVLPKPALVASRVEVSKVL	Negative
2800.	Q8C196:424	LPKPALVASRVEVSKVLILGSGGLSIGQA	Negative
2801.	Q8C196:450	GQAGEFDYSGSQAVKAMKEENVKTVLMNP	Negative
2802.	Q8C196:453	GEFDYSGSQAVKAMKEENVKTVLMNPNIA	Negative
2803.	Q8C196:458	SGSQAVKAMKEENVKTVLMNPNIASVQTN	Negative
2804.	Q8C196:477	NPNIASVQTNEVGLKQADAVYFLPITPQF	Negative
2805.	Q8C196:497	YFLPITPQFVTEVIKAERPDGLILGMGGQ	Negative
2806.	Q8C196:522	MGGQTALNCGVELFKRGVLKEYGVKVLGT	Negative
2807.	Q8C196:527	ALNCGVELFKRGVLKEYGVKVLGTSVESI	Negative
2808.	Q8C196:532	VELFKRGVLKEYGVKVLGTSVESIMATED	Negative
2809.	Q8C196:553	ESIMATEDRQLFSDKLNEINEKIAPSFAV	Negative
2810.	Q8C196:560	DRQLFSDKLNEINEKIAPSFAVESMEDAL	Negative
2811.	Q8C196:575	IAPSFAVESMEDALKAADTIGYPVMIRSA	Negative
2812.	Q8C196:603	AYALGGLGSGICPNKETLIDLGTKAFAMT	Negative
2813.	Q8C196:612	GICPNKETLIDLGTKAFAMTNQILVERSV	Negative
2814.	Q8C196:630	MTNQILVERSVTGWKEIEYEVVRDADDNC	Negative
2815.	Q8C196:728	EVNARLSRSSALASKATGYPLAFIAAKIA	Negative
2816.	Q8C196:740	ASKATGYPLAFIAAKIALGIPLPEIKNVV	Negative
2817.	Q8C196:751	IAAKIALGIPLPEIKNVVSGKTSACFEPS	Negative
2818.	Q8C196:757	LGIPLPEIKNVVSGKTSACFEPSLDYMVT	Negative
2819.	Q8C196:772	TSACFEPSLDYMVTKIPRWDLDRFHGTSS	Negative
2820.	Q8C196:793	DRFHGTSSRIGSSMKSVGEVMAIGRTFEE	Negative
2821.	Q8C196:831	HPSVDGFTPRLPMNKEWPANLDLKKELSE	Negative
2822.	Q8C196:840	RLPMNKEWPANLDLKKELSEPSSTRIYAI	Negative
2823.	Q8C196:841	LPMNKEWPANLDLKKELSEPSSTRIYAIA	Negative
2824.	Q8C196:856	ELSEPSSTRIYAIAKALENNMSLDEIVRL	Negative
2825.	Q8C196:875	NMSLDEIVRLTSIDKWFLYKMRDILNMDK	Negative
2826.	Q8C196:880	EIVRLTSIDKWFLYKMRDILNMDKTLKGL	Negative
2827.	Q8C196:889	KWFLYKMRDILNMDKTLKGLNSDSVTEET	Negative
2828.	Q8C196:892	LYKMRDILNMDKTLKGLNSDSVTEETLRK	Negative
2829.	Q8C196:906	KGLNSDSVTEETLRKAKEIGFSDKQISKC	Negative
2830.	Q8C196:908	LNSDSVTEETLRKAKEIGFSDKQISKCLG	Negative
2831.	Q8C196:915	EETLRKAKEIGFSDKQISKCLGLTEAQTR	Negative
2832.	Q8C196:919	RKAKEIGFSDKQISKCLGLTEAQTRELRL	Negative
2833.	Q8C196:934	CLGLTEAQTRELRLKKNIHPWVKQIDTLA	Negative
2834.	Q8C196:935	LGLTEAQTRELRLKKNIHPWVKQIDTLAA	Negative
2835.	Q8C196:942	TRELRLKKNIHPWVKQIDTLAAEYPSVTN	Negative
2836.	Q8C196:970	NYLYVTYNGQEHDIKFDEHGIMVLGCGPY	Negative
2837.	Q8C196:1008	WCAVSSIRTLRQLGKKTVVVNCNPETVST	Negative
2838.	Q8C196:1009	CAVSSIRTLRQLGKKTVVVNCNPETVSTD	Negative
2839.	Q8C196:1029	CNPETVSTDFDECDKLYFEELSLERILDI	Negative
2840.	Q8C196:1070	VGGQIPNNLAVPLYKNGVKIMGTSPLQID	Negative
2841.	Q8C196:1074	IPNNLAVPLYKNGVKIMGTSPLQIDRAED	Negative
2842.	Q8C196:1100	AEDRSIFSAVLDELKVAQAPWKAVNTLNE	Negative
2843.	Q8C196:1107	SAVLDELKVAQAPWKAVNTLNEALEFANS	Negative
2844.	Q8C196:1149	SGSAMNVVFSEDEMKRFLEEATRVSQEHP	Negative
2845.	Q8C196:1168	EATRVSQEHPVVLTKFVEGAREVEMDAVG	Negative
2846.	Q8C196:1183	FVEGAREVEMDAVGKEGRVISHAISEHVE	Negative
2847.	Q8C196:1222	LMLPTQTISQGAIEKVKDATRKIAKAFAI	Negative
2848.	Q8C196:1224	LPTQTISQGAIEKVKDATRKIAKAFAISG	Negative
2849.	Q8C196:1229	ISQGAIEKVKDATRKIAKAFAISGPFNVQ	Negative
2850.	Q8C196:1232	GAIEKVKDATRKIAKAFAISGPFNVQFLV	Negative
2851.	Q8C196:1247	AFAISGPFNVQFLVKGNDVLVIECNLRAS	Negative
2852.	Q8C196:1269	ECNLRASRSFPFVSKTLGVDFIDVATKVM	Negative

2853.	Q8C196:1281	VSKTLGVDFIDVATKVMIGESIDEKRLPT	Negative
2854.	Q8C196:1291	DVATKVMIGESIDEKRLPTLEQPIIPSDY	Negative
2855.	Q8C196:1309	TLEQPIIPSDYVAIKAPMFSWPRLRDADP	Negative
2856.	Q8C196:1348	EVACFGEGIHTAFLKAMLSTGFKIPQKGI	Negative
2857.	Q8C196:1356	IHTAFLKAMLSTGFKIPQKGILIGIQQSF	Negative
2858.	Q8C196:1360	FLKAMLSTGFKIPQKGILIGIQQSFRPRF	Negative
2859.	Q8C196:1387	RFLGVAEQLHNEGFKLFATEATSDWLNAN	Negative
2860.	Q8C196:1425	PSQEGQNPSLSSIRKLIRDGSIDLVINLP	Negative
2861.	Q8C196:1444	GSIDLVINLPNNNTKFVHDNYVIRRTAVD	Negative
2862.	Q8C196:1471	VDSGIALLTNFQVTKLFAEAVQKSRTVDS	Negative
2863.	Q8C196:1486	LFAEAVQKSRTVDSKSLFHYRQYSAGKAA	Negative
2864.	Q8C196:1498	DSKSLFHYRQYSAGKAAXXXXXXXXXXXX	Negative
2865.	Q8VD72-2:19	MEPLLRAWSYFRRRKFQLCADLCTQMLEK	Negative
2866.	Q8VD72-2:44	MLEKSPYDQAAWILKARALTEMVYIDEID	Negative
2867.	Q8VD72-2:85	ENAIAQVPRPGTSLKLPGTNQTGGPTQAV	Negative
2868.	Q8VD72-2:174	PDGPFINLSRLNLTKYSQKPKLAKALFEY	Negative
2869.	Q8VD72-2:178	FINLSRLNLTKYSQKPKLAKALFEYILHH	Negative
2870.	Q8VD72-2:180	NLSRLNLTKYSQKPKLAKALFEYILHHEN	Negative
2871.	Q8VD72-2:183	RLNLTKYSQKPKLAKALFEYILHHENDVK	Negative
2872.	Q8VD72-2:197	KALFEYILHHENDVKMALDLASLSTEYSQ	Negative
2873.	Q8VD72-2:213	ALDLASLSTEYSQYKDWWWKVQIGKCYYR	Negative
2874.	Q8VD72-2:218	SLSTEYSQYKDWWWKVQIGKCYYRLGMYR	Negative
2875.	Q8VD72-2:223	YSQYKDWWWKVQIGKCYYRLGMYREAEKQ	Negative
2876.	Q8VD72-2:236	GKCYYRLGMYREAEKQFKSALKQQEMVDT	Negative
2877.	Q8VD72-2:239	YYRLGMYREAEKQFKSALKQQEMVDTFLY	Negative
2878.	Q8VD72-2:243	GMYREAEKQFKSALKQQEMVDTFLYLAKV	Negative
2879.	Q8VD72-2:256	LKQQEMVDTFLYLAKVYIILDQPVTALNL	Negative
2880.	Q8VD72-2:272	YIILDQPVTALNLFKQGLDKFPGEVTLLC	Negative
2881.	Q8VD72-2:277	QPVTALNLFKQGLDKFPGEVTLLCGIARI	Negative
2882.	Q8VD72-2:306	YEEMNNSSSAAEYYKEVLKQDNTHVEAIA	Negative
2883.	Q8VD72-2:310	NNSSSAAEYYKEVLKQDNTHVEAIACIGS	Negative
2884.	Q8VD72-2:434	HAEAYNNLAVLEMRKGHVEQARALLQTAS	Negative
2885.	Q8VD72-2:467	HMYEPHFNFATVSDKIGDLQRSYVAAQKS	Negative
2886.	Q8VD72-2:480	DKIGDLQRSYVAAQKSEVAFPEHVDTQHL	Negative

2887.	Q8VD72-2:496 Negative	EVAFPEHVDTQHLIKQLKQHFAMLXXXXX	
2888.	Q8VD72-2:499 Negative	FPEHVDTQHLIKQLKQHFAMLXXXXXXXX	
2889.	Q91V76:22	FHMPSLEELAEVLQKGLTDNFADVQVSVV	Negative
2890.	Q91V76:43	ADVQVSVVDCPDLTKEPFTFPVRGICGQT	Negative
2891.	Q91V76:74	AEVGGVPYLLPLVNKKKVYDLNEIAKVIK	Negative
2892.	Q91V76:76	VGGVPYLLPLVNKKKVYDLNEIAKVIKLP	Negative
2893.	Q91V76:85	LVNKKKVYDLNEIAKVIKLPGAFILGAGA	Negative
2894.	Q91V76:88	KKKVYDLNEIAKVIKLPGAFILGAGAGPF	Negative
2895.	Q91V76:133	SEHNQPVNGSYFAHKNPADGACLLEKYSQ	Negative
2896.	Q91V76:144	FAHKNPADGACLLEKYSQKYHDFGCALLA	Negative
2897.	Q91V76:148	NPADGACLLEKYSQKYHDFGCALLANLFA	Negative
2898.	Q91V76:169	ALLANLFASEGQPGKVIEVQAKRRTGELN	Negative
2899.	Q91V76:176	ASEGQPGKVIEVQAKRRTGELNFVSCMRQ	Negative
2900.	Q91V76:199	VSCMRQTLEEHYGDKPVGMGGTFIVQKGK	Negative
2901.	Q91V76:211	GDKPVGMGGTFIVQKGKVKAHIMPAEFSS	Negative
2902.	Q91V76:213	KPVGMGGTFIVQKGKVKAHIMPAEFSSCP	Negative
2903.	Q91V76:215	VGMGGTFIVQKGKVKAHIMPAEFSSCPLN	Negative
2904.	Q91V76:236	EFSSCPLNSDEAVNKWLHFYEMKAPLVCL	Negative
2905.	Q91V76:244	SDEAVNKWLHFYEMKAPLVCLPVFVSKDP	Negative
2906.	Q91V76:256	EMKAPLVCLPVFVSKDPGLDLRLEHTHFF	Negative
2907.	Q91V76:307	YFSPAQFLYRIDQPKETHAFGRDXXXXXX	Negative
2908.	Q920E5:6	XXXXXXXXXMNGNQKLDAYNQEKQNFIQH	Negative
2909.	Q920E5:14	XMNGNQKLDAYNQEKQNFIQHFSQIVKVL	Negative
2910.	Q920E5:26	QEKQNFIQHFSQIVKVLTEKELGHPEIGD	Negative
2911.	Q920E5:31	FIQHFSQIVKVLTEKELGHPEIGDAIARL	Negative
2912.	Q920E5:46	ELGHPEIGDAIARLKEVLEYNALGGKYNR	Negative
2913.	Q920E5:75	GLTVVQAFQELVEPKKQDAESLQRALTVG	Negative
2914.	Q920E5:76	LTVVQAFQELVEPKKQDAESLQRALTVGW	Negative
2915.	Q920E5:121	DSSLTRRGQICWYQKPGIGLDAINDALLL	Negative
2916.	Q920E5:144	NDALLLEASIYRLLKFYCREQPYYLNLLE	Negative
2917.	Q920E5:191	TAPQGHVDLGRYTEKRYKSIVKYKTAFYS	Negative
2918.	Q920E5:194	QGHVDLGRYTEKRYKSIVKYKTAFYSFYL	Negative
2919.	Q920E5:198	DLGRYTEKRYKSIVKYKTAFYSFYLPIAA	Negative
2920.	Q920E5:200	GRYTEKRYKSIVKYKTAFYSFYLPIAAAM	Negative
2921.	Q920E5:223	PIAAAMYMAGIDGEKEHANALKILMEMGE	Negative
2922.	Q920E5:230	MAGIDGEKEHANALKILMEMGEFFQVQDD	Negative
2923.	Q920E5:257	DDYLDLFGDPSVTGKVGTDIQDNKCSWLV	Negative
2924.	Q920E5:266	PSVTGKVGTDIQDNKCSWLVVQCLLRASP	Negative
2925.	Q920E5:293	SPQQRQILEENYGQKDPEKVARVKALYEA	Negative
2926.	Q920E5:297	RQILEENYGQKDPEKVARVKALYEALDLQ	Negative
2927.	Q920E5:302	ENYGQKDPEKVARVKALYEALDLQSAFFK	Negative
2928.	Q920E5:316	KALYEALDLQSAFFKYEEDSYNRLKSLIE	Negative
2929.	Q920E5:326	SAFFKYEEDSYNRLKSLIEQCSAPLPPSI	Negative
2930.	Q920E5:347	SAPLPPSIFMELANKIYKRRKXXXXXXXX	Negative
2931.	Q920E5:350	LPPSIFMELANKIYKRRKXXXXXXXXXXX	Negative
2932.	Q920E5:353	SIFMELANKIYKRRKXXXXXXXXXXXXXX	Negative
2933.	Q93092:7	XXXXXXXXMSGSPVKRQRMESALDQLKQF	Negative
2934.	Q93092:19	PVKRQRMESALDQLKQFTTVVADTGDFNA	Negative
2935.	Q93092:38	VVADTGDFNAIDEYKPQDATTNPSLILAA	Negative
2936.	Q93092:70	PAYQELVEEAIAYGKKLGGPQEEQIKNAI	Negative
2937.	Q93092:71	AYQELVEEAIAYGKKLGGPQEEQIKNAID	Negative
2938.	Q93092:81	AYGKKLGGPQEEQIKNAIDKLFVLFGAEI	Negative

2939.	Q93092:86	LGGPQEEQIKNAIDKLFVLFGAEILKKIP	Negative
2940.	Q93092:97	AIDKLFVLFGAEILKKIPGRVSTEVDARL	Negative
2941.	Q93092:98	IDKLFVLFGAEILKKIPGRVSTEVDARLS	Negative
2942.	Q93092:115	GRVSTEVDARLSFDKDAMVARARRLIELY	Negative
2943.	Q93092:130	DAMVARARRLIELYKEAGVGKDRILIKLS	Negative
2944.	Q93092:136	ARRLIELYKEAGVGKDRILIKLSSTWEGI	Negative
2945.	Q93092:142	LYKEAGVGKDRILIKLSSTWEGIQAGKEL	Negative
2946.	Q93092:154	LIKLSSTWEGIQAGKELEEQHGIHCNMTL	Negative
2947.	Q93092:203	FVGRILDWHVANTDKKSYEPQEDPGVKSV	Negative
2948.	Q93092:204	VGRILDWHVANTDKKSYEPQEDPGVKSVT	Negative
2949.	Q93092:215	TDKKSYEPQEDPGVKSVTKIYNYYKKFGY	Negative
2950.	Q93092:219	SYEPQEDPGVKSVTKIYNYYKKFGYKTIV	Negative
2951.	Q93092:225	DPGVKSVTKIYNYYKKFGYKTIVMGASFR	Negative
2952.	Q93092:226	PGVKSVTKIYNYYKKFGYKTIVMGASFRN	Negative
2953.	Q93092:230	SVTKIYNYYKKFGYKTIVMGASFRNTGEI	Negative
2954.	Q93092:245	TIVMGASFRNTGEIKALAGCDFLTISPKL	Negative
2955.	Q93092:258	IKALAGCDFLTISPKLLGELLKDNSKLAP	Negative
2956.	Q93092:265	DFLTISPKLLGELLKDNSKLAPALSVKAA	Negative
2957.	Q93092:269	ISPKLLGELLKDNSKLAPALSVKAAQTSD	Negative
2958.	Q93092:277	LLKDNSKLAPALSVKAAQTSDSEKIHLDE	Negative
2959.	Q93092:286	PALSVKAAQTSDSEKIHLDEKAFRWLHNE	Negative
2960.	Q93092:292	AAQTSDSEKIHLDEKAFRWLHNEDQMAVE	Negative
2961.	Q93092:307	AFRWLHNEDQMAVEKLSDGIRKFAADAIK	Negative
2962.	Q93092:321	KLSDGIRKFAADAIKLERMLTERMFSAEN	Negative
2963.	Q93092:337	ERMLTERMFSAENGKXXXXXXXXXXXXXX	Negative
2964.	Q9BYV9:6	XXXXXXXXXMSVDEKPDSPMYVYESTVHC	Negative
2965.	Q9BYV9:32	VHCTNILLGLNDQRKKDILCDVTLIVERK	Negative
2966.	Q9BYV9:33	HCTNILLGLNDQRKKDILCDVTLIVERKE	Negative
2967.	Q9BYV9:46	KKDILCDVTLIVERKEFRAHRAVLAACSE	Negative
2968.	Q9BYV9:71	ACSEYFWQALVGQTKNDLVVSLPEEVTAR	Negative
2969.	Q9BYV9:98	ARGFGPLLQFAYTAKLLLSRENIREVIRC	Negative
2970.	Q9BYV9:145	TQLLNSEDGLFVCRKDAACQRPHEDCENS	Negative
2971.	Q9BYV9:202	EPISFEAAAIPVAEKEEALLPEPDVPTDT	Negative
2972.	Q9BYV9:217	EEALLPEPDVPTDTKESSEKDALTQYPRY	Negative
2973.	Q9BYV9:222	PEPDVPTDTKESSEKDALTQYPRYKKYQL	Negative
2974.	Q9BYV9:232	ESSEKDALTQYPRYKKYQLACTKNVYNAS	Negative
2975.	Q9BYV9:233	SSEKDALTQYPRYKKYQLACTKNVYNASS	Negative
2976.	Q9BYV9:240	TQYPRYKKYQLACTKNVYNASSHSTSGFA	Negative
2977.	Q9BYV9:267	FASTFREDNSSNSLKPGLARGQIKSEPPS	Negative
2978.	Q9BYV9:276	SSNSLKPGLARGQIKSEPPSEENEEESIT	Negative
2979.	Q9BYV9:301	ESITLCLSGDEPDAKDRAGDVEMDRKQPS	Negative
2980.	Q9BYV9:312	PDAKDRAGDVEMDRKQPSPAPTPTAPAGA	Negative
2981.	Q9BYV9:349	VASPSCLRSLFSITKSVELSGLPSTSQQH	Negative
2982.	Q9BYV9:374	SQQHFARSPACPFDKGITQGDLKTDYTPF	Negative
2983.	Q9BYV9:382	PACPFDKGITQGDLKTDYTPFTGNYGQPH	Negative
2984.	Q9BYV9:400	TPFTGNYGQPHVGQKEVSNFTMGSPLRGP	Negative
2985.	Q9BYV9:421	MGSPLRGPGLEALCKQEGELDRRSVIFSS	Negative
2986.	Q9BYV9:456	STSVHSYSGVSSLDKDLSEPVPKGLWVGA	Negative
2987.	Q9BYV9:464	GVSSLDKDLSEPVPKGLWVGAGQSLPSSQ	Negative
2988.	Q9BYV9:505	PGRMRPNTSCPVPIKVCPRSPPLETRTRT	Negative
2989.	Q9BYV9:580	LMGDGMYNQVRPQIKCEQSYGTNSSDESG	Negative
2990.	Q9BYV9:614	DSESCPVQDRGQEVKLPFPVDQITDLPRN	Negative
2991.	Q9BYV9:635	QITDLPRNDFQMMIKMHKLTSEQLEFIHD	Negative
2992.	Q9BYV9:638	DLPRNDFQMMIKMHKLTSEQLEFIHDVRR	Negative

2993.	Q9BYV9:655	SEQLEFIHDVRRRSKNRIAAQRCRKRKLD	Negative
2994.	Q9BYV9:665	RRRSKNRIAAQRCRKRKLDCIQNLECEIR	Negative
2995.	Q9BYV9:667	RSKNRIAAQRCRKRKLDCIQNLECEIRKL	Negative
2996.	Q9BYV9:680	RKLDCIQNLECEIRKLVCEKEKLLSERNQ	Negative
2997.	Q9BYV9:685	IQNLECEIRKLVCEKEKLLSERNQLKACM	Negative
2998.	Q9BYV9:687	NLECEIRKLVCEKEKLLSERNQLKACMGE	Negative
2999.	Q9BYV9:696	VCEKEKLLSERNQLKACMGELLDNFSCLS	Negative
3000.	Q9BYV9:829	SQTVTVDFCQEMTDKCTTDEQPRKDYTXX	Negative
3001.	Q9BYV9:838	QEMTDKCTTDEQPRKDYTXXXXXXXXXXX	Negative
3002.	Q9C026:9	XXXXXXMEEMEEELKCPVCGSFYREPIIL	Negative
3003.	Q9C026:67	AGSGVSDYDYLDLDKMSLYSEADSGYGSY	Negative
3004.	Q9C026:94	SYGGFASAPTTPCQKSPNGVRVFPPAMPP	Negative
3005.	Q9C026:147	RSLILDDRGLRGFPKNRVLEGVIDRYQQS	Negative
3006.	Q9C026:162	NRVLEGVIDRYQQSKAAALKCQLCEKAPK	Negative
3007.	Q9C026:167	GVIDRYQQSKAAALKCQLCEKAPKEATVM	Negative
3008.	Q9C026:173	QQSKAAALKCQLCEKAPKEATVMCEQCDV	Negative
3009.	Q9C026:176	KAAALKCQLCEKAPKEATVMCEQCDVFYC	Negative
3010.	Q9C026:206	PCRLRCHPPRGPLAKHRLVPPAQGRVSRR	Negative
3011.	Q9C026:225	PPAQGRVSRRLSPRKVSTCTDHELENHSM	Negative
3012.	Q9C026:245	DHELENHSMYCVQCKMPVCYQCLEEGKHS	Negative
3013.	Q9C026:257	QCKMPVCYQCLEEGKHSSHEVKALGAMWK	Negative
3014.	Q9C026:264	YQCLEEGKHSSHEVKALGAMWKLHKSQLS	Negative
3015.	Q9C026:271	KHSSHEVKALGAMWKLHKSQLSQALNGLS	Negative
3016.	Q9C026:274	SHEVKALGAMWKLHKSQLSQALNGLSDRA	Negative
3017.	Q9C026:289	SQLSQALNGLSDRAKEAKEFLVQLRNMVQ	Negative
3018.	Q9C026:292	SQALNGLSDRAKEAKEFLVQLRNMVQQIQ	Negative
3019.	Q9C026:331	VAQCDALIDALNRRKAQLLARVNKEHEHK	Negative
3020.	Q9C026:340	ALNRRKAQLLARVNKEHEHKLKVVRDQIS	Negative
3021.	Q9C026:345	KAQLLARVNKEHEHKLKVVRDQISHCTVK	Negative
3022.	Q9C026:359	KLKVVRDQISHCTVKLRQTTGLMEYCLEV	Negative
3023.	Q9C026:375	RQTTGLMEYCLEVIKENDPSGFLQISDAL	Negative
3024.	Q9C026:402	ALIRRVHLTEDQWGKGTLTPRMTTDFDLS	Negative
3025.	Q9C026:435	PLLQSIHQLDFVQVKASSPVPATPILQLE	Negative
3026.	Q9C026:463	EECCTHNNSATLSWKQPPLSTVPADGYIL	Negative
3027.	Q9C026:494	DDGNGGQFREVYVGKETMCTVDGLHFNST	Negative
3028.	Q9C026:514	VDGLHFNSTYNARVKAFNKTGVSPYSKTL	Negative
3029.	Q9C026:518	HFNSTYNARVKAFNKTGVSPYSKTLVLQT	Negative
3030.	Q9C026:526	RVKAFNKTGVSPYSKTLVLQTSEVAWFAF	Negative
3031.	Q9C026:571	TVTCSSYDDRVVLGKTGFSKGIHYWELTV	Negative
3032.	Q9C026:576	SYDDRVVLGKTGFSKGIHYWELTVDRYDN	Negative
3033.	Q9C026:605	HPDPAFGVARMDVMKDVMLGKDDKAWAMY	Negative
3034.	Q9C026:611	GVARMDVMKDVMLGKDDKAWAMYVDNNRS	Negative
3035.	Q9C026:614	RMDVMKDVMLGKDDKAWAMYVDNNRSWFM	Negative
3036.	Q9C026:643	HNNSHTNRTEGGITKGATIGVLLDLNRKN	Negative
3037.	Q9C026:656	TKGATIGVLLDLNRKNLTFFINDEQQGPI	Negative
3038.	Q9D020:34	VVAGVVLAQYIFTLKRKTGRKTKIIEMMP	Negative
3039.	Q9D020:36	AGVVLAQYIFTLKRKTGRKTKIIEMMPEF	Negative
3040.	Q9D020:40	LAQYIFTLKRKTGRKTKIIEMMPEFQKSS	Negative
3041.	Q9D020:42	QYIFTLKRKTGRKTKIIEMMPEFQKSSVR	Negative
3042.	Q9D020:52	GRKTKIIEMMPEFQKSSVRIKNPTRVEEI	Negative
3043.	Q9D020:58	IEMMPEFQKSSVRIKNPTRVEEIICGLIK	Negative
3044.	Q9D020:72	KNPTRVEEIICGLIKGGAAKLQIITDFDM	Negative
3045.	Q9D020:77	VEEIICGLIKGGAAKLQIITDFDMTLSRF	Negative

3046.	Q9D020:96	TDFDMTLSRFSYNGKRCPTCHNIIDNCKL	Negative
3047.	Q9D020:109	GKRCPTCHNIIDNCKLVTDECRRKLLQLK	Negative
3048.	Q9D020:118	IIDNCKLVTDECRRKLLQLKEQYYAIEVD	Negative
3049.	Q9D020:140	YYAIEVDPVLTVEEKFPYMVEWYTKSHGL	Negative
3050.	Q9D020:150	TVEEKFPYMVEWYTKSHGLLIEQGIPKAK	Negative
3051.	Q9D020:162	YTKSHGLLIEQGIPKAKLKEIVADSDVML	Negative
3052.	Q9D020:164	KSHGLLIEQGIPKAKLKEIVADSDVMLKE	Negative
3053.	Q9D020:166	HGLLIEQGIPKAKLKEIVADSDVMLKEGY	Negative
3054.	Q9D020:177	AKLKEIVADSDVMLKEGYENFFGKLQQHG	Negative
3055.	Q9D020:186	SDVMLKEGYENFFGKLQQHGIPVFIFSAG	Negative
3056.	Q9D020:220	EEVIRQAGVYHSNVKVVSNFMDFDENGVL	Negative
3057.	Q9D020:235	VVSNFMDFDENGVLKGFKGELIHVFNKHD	Negative
3058.	Q9D020:238	NFMDFDENGVLKGFKGELIHVFNKHDGAL	Negative
3059.	Q9D020:247	VLKGFKGELIHVFNKHDGALKNTDYFSQL	Negative
3060.	Q9D020:253	GELIHVFNKHDGALKNTDYFSQLKDNSNI	Negative
3061.	Q9D020:262	HDGALKNTDYFSQLKDNSNIILLGDSQGD	Negative
3062.	Q9D020:291	LRMADGVANVEHILKIGYLNDRVDELLEK	Negative
3063.	Q9D020:305	KIGYLNDRVDELLEKYMDSYDIVLVKEES	Negative
3064.	Q9D020:316	LLEKYMDSYDIVLVKEESLEVVNSILQKT	Negative
3065.	Q9D020:329	VKEESLEVVNSILQKTLXXXXXXXXXXXX	Negative
3066.	Q9DAY2:27	PLLLLVVSNLLLWEKAASNLPCVAEEGGC	Negative
3067.	Q9DAY2:55	CWNPLLETFNSATQKAETLHNLADQLYVE	Negative
3068.	Q9DAY2:92	GQFWDFSSQIIRQDKTVVRAGSYCHSSLT	Negative
3069.	Q9DAY2:124	NTGVHINIEIASYLKTLINFVGSWISPLF	Negative
3070.	Q9DAY2:148	ISPLFHLVIELSATKDVPETILSKAKEIE	Negative
3071.	Q9DAY2:157	ELSATKDVPETILSKAKEIEENNRQILSD	Negative
3072.	Q9DAY2:159	SATKDVPETILSKAKEIEENNRQILSDLR	Negative
3073.	Q9DAY2:178	NNRQILSDLRWILTKVSPAAEMTEEFPHW	Negative
3074.	Q9DAY2:199	MTEEFPHWEYLSFLKSSDKNNKFLAMFNL	Negative
3075.	Q9DAY2:203	FPHWEYLSFLKSSDKNNKFLAMFNLSYCI	Negative
3076.	Q9DAY2:222	LAMFNLSYCIDHDSKYILLQLRLLKCLIT	Negative
3077.	Q9DAY2:232	DHDSKYILLQLRLLKCLITGKDCXXXXXX	Negative
3078.	Q9DAY2:238	ILLQLRLLKCLITGKDCXXXXXXXXXXXX	Negative
3079.	Q9DB16:2	XXXXXXXXXXXXXMKKMPLFSKSHKNPAE	Negative
3080.	Q9DB16:9	XXXXXXMKKMPLFSKSHKNPAEIVKILKD	Negative
3081.	Q9DB16:12	XXXMKKMPLFSKSHKNPAEIVKILKDNLA	Negative
3082.	Q9DB16:19	PLFSKSHKNPAEIVKILKDNLAILEKQDK	Negative
3083.	Q9DB16:22	SKSHKNPAEIVKILKDNLAILEKQDKKTD	Negative
3084.	Q9DB16:30	EIVKILKDNLAILEKQDKKTDKASEEVSK	Negative
3085.	Q9DB16:33	KILKDNLAILEKQDKKTDKASEEVSKSLQ	Negative
3086.	Q9DB16:34	ILKDNLAILEKQDKKTDKASEEVSKSLQA	Negative
3087.	Q9DB16:37	DNLAILEKQDKKTDKASEEVSKSLQAMKE	Negative
3088.	Q9DB16:44	KQDKKTDKASEEVSKSLQAMKEILCGTND	Negative
3089.	Q9DB16:50	DKASEEVSKSLQAMKEILCGTNDKEPPTE	Negative
3090.	Q9DB16:59	SLQAMKEILCGTNDKEPPTEAVAQLAQEL	Negative
3091.	Q9DB16:94	VTLIADLQLIDFEGKKDVTQIFNNILRRQ	Negative
3092.	Q9DB16:95	TLIADLQLIDFEGKKDVTQIFNNILRRQI	Negative
3093.	Q9DB16:131	EYISSHPHILFMLLKGYEAPQIALRCGIM	Negative
3094.	Q9DB16:157	GIMLRECIRHEPLAKIIILFSNQFRDFFKY	Negative
3095.	Q9DB16:170	AKIIILFSNQFRDFFKYVELSTFDIASDAF	Negative
3096.	Q9DB16:188	LSTFDIASDAFATFKDLLTRHKVLVADFL	Negative
3097.	Q9DB16:195	SDAFATFKDLLTRHKVLVADFLEQNYDTI	Negative
3098.	Q9DB16:215	FLEQNYDTIFEDYEKLLQSENYVTKRQSL	Negative
3099.	Q9DB16:225	EDYEKLLQSENYVTKRQSLKLLGELILDR	Negative

3100.	Q9DB16:230	LLQSENYVTKRQSLKLLGELILDRHNFTI	Negative
3101.	Q9DB16:247	GELILDRHNFTIMTKYISKPENLKLMMNL	Negative
3102.	Q9DB16:251	LDRHNFTIMTKYISKPENLKLMMNLLRDK	Negative
3103.	Q9DB16:256	FTIMTKYISKPENLKLMMNLLRDKSPNIQ	Negative
3104.	Q9DB16:265	KPENLKLMMNLLRDKSPNIQFEAFHVFKV	Negative
3105.	Q9DB16:278	DKSPNIQFEAFHVFKVFVASPHKTQPIVE	Negative
3106.	Q9DB16:286	EAFHVFKVFVASPHKTQPIVEILLKNQPK	Negative
3107.	Q9DB16:296	ASPHKTQPIVEILLKNQPKLIEFLSSFQK	Negative
3108.	Q9DB16:300	KTQPIVEILLKNQPKLIEFLSSFQKERTD	Negative
3109.	Q9DB16:310	KNQPKLIEFLSSFQKERTDDEQFADEKNY	Negative
3110.	Q9DB16:322	FQKERTDDEQFADEKNYLIKQIRDLKKAA	Negative
3111.	Q9DB16:327	TDDEQFADEKNYLIKQIRDLKKAAPXXXX	Negative
3112.	Q9DB16:333	ADEKNYLIKQIRDLKKAAPXXXXXXXXXX	Negative
3113.	Q9DB16:334	DEKNYLIKQIRDLKKAAPXXXXXXXXXXX	Negative
3114.	Q9EP89:55	AGGLGLGLGLALGAKLVVGLRGAVPIQSP	Negative
3115.	Q9EP89:117	SRAIESSRDLLHRIKDEVGAPGIVVGVSV	Negative
3116.	Q9EP89:134	VGAPGIVVGVSVDGKEVWSEGLGYADVEN	Negative
3117.	Q9EP89:153	EGLGYADVENRVPCKPETVMRIASISKSL	Negative
3118.	Q9EP89:165	PCKPETVMRIASISKSLTMVALAKLWEAG	Negative
3119.	Q9EP89:174	IASISKSLTMVALAKLWEAGKLDLDLPVQ	Negative
3120.	Q9EP89:180	SLTMVALAKLWEAGKLDLDLPVQHYVPEF	Negative
3121.	Q9EP89:197	LDLPVQHYVPEFPEKEYEGEKVSVTTRLL	Negative
3122.	Q9EP89:203	HYVPEFPEKEYEGEKVSVTTRLLISHLSG	Negative
3123.	Q9EP89:223	RLLISHLSGIRHYEKDIKKVKEEKAYKAL	Negative
3124.	Q9EP89:226	ISHLSGIRHYEKDIKKVKEEKAYKALKMV	Negative
3125.	Q9EP89:227	SHLSGIRHYEKDIKKVKEEKAYKALKMVK	Negative
3126.	Q9EP89:229	LSGIRHYEKDIKKVKEEKAYKALKMVKGT	Negative
3127.	Q9EP89:232	IRHYEKDIKKVKEEKAYKALKMVKGTPPP	Negative
3128.	Q9EP89:235	YEKDIKKVKEEKAYKALKMVKGTPPPSDQ	Negative
3129.	Q9EP89:238	DIKKVKEEKAYKALKMVKGTPPPSDQEKE	Negative
3130.	Q9EP89:241	KVKEEKAYKALKMVKGTPPPSDQEKELKE	Negative
3131.	Q9EP89:251	LKMVKGTPPPSDQEKELKEKGGKNNEKSD	Negative
3132.	Q9EP89:254	VKGTPPPSDQEKELKEKGGKNNEKSDAPK	Negative
3133.	Q9EP89:256	GTPPPSDQEKELKEKGGKNNEKSDAPKAK	Negative
3134.	Q9EP89:259	PPSDQEKELKEKGGKNNEKSDAPKAKVEQ	Negative
3135.	Q9EP89:263	QEKELKEKGGKNNEKSDAPKAKVEQDSEA	Negative
3136.	Q9EP89:268	KEKGGKNNEKSDAPKAKVEQDSEARCRSA	Negative
3137.	Q9EP89:270	KGGKNNEKSDAPKAKVEQDSEARCRSAKP	Negative
3138.	Q9EP89:283	AKVEQDSEARCRSAKPGKKKNDFEQGELY	Negative
3139.	Q9EP89:286	EQDSEARCRSAKPGKKKNDFEQGELYLKE	Negative
3140.	Q9EP89:287	QDSEARCRSAKPGKKKNDFEQGELYLKEK	Negative
3141.	Q9EP89:288	DSEARCRSAKPGKKKNDFEQGELYLKEKF	Negative
3142.	Q9EP89:299	GKKKNDFEQGELYLKEKFENSIESLRLFK	Negative
3143.	Q9EP89:301	KKNDFEQGELYLKEKFENSIESLRLFKND	Negative
3144.	Q9EP89:313	KEKFENSIESLRLFKNDPLFFKPGSQFLY	Negative
3145.	Q9EP89:320	IESLRLFKNDPLFFKPGSQFLYSTFGYTL	Negative
3146.	Q9EP89:346	YTLLAAIVERASGYKYLDYMQKIFHDLDM	Negative
3147.	Q9EP89:353	VERASGYKYLDYMQKIFHDLDMLTTVQEE	Negative
3148.	Q9EP89:384	PVIYNRARFYVYNKKKRLVNTPYVDNSYK	Negative
3149.	Q9EP89:385	VIYNRARFYVYNKKKRLVNTPYVDNSYKW	Negative
3150.	Q9EP89:398	KKRLVNTPYVDNSYKWAGGGFLSTVGDLL	Negative
3151.	Q9EP89:413	WAGGGFLSTVGDLLKFGNAMLYGYQVGQF	Negative
3152.	Q9EP89:428	FGNAMLYGYQVGQFKNSNENLLPGYLKPE	Negative
3153.	Q9EP89:440	QFKNSNENLLPGYLKPETMVMMWTPVPNT	Negative

3154.	Q9EP89:460	MMWTPVPNTEMSWDKEGKYAMAWGVVEKK	Negative
3155.	Q9EP89:463	TPVPNTEMSWDKEGKYAMAWGVVEKKQTY	Negative
3156.	Q9EP89:473	DKEGKYAMAWGVVEKKQTYGSCRKQRHYA	Negative
3157.	Q9EP89:474	KEGKYAMAWGVVEKKQTYGSCRKQRHYAS	Negative
3158.	Q9EP89:482	WGVVEKKQTYGSCRKQRHYASHTGGAVGA	Negative
3159.	Q9EP89:515	LVLPEELDSEAVNNKVPPRGIIVSIICNM	Negative
3160.	Q9EP89:540	ICNMQSVGLNSTALKIALEFDKDRADXXX	Negative
3161.	Q9EP89:547	GLNSTALKIALEFDKDRADXXXXXXXXXX	Negative
3162.	Q9EQF5:57	EDASRGLRILDAAGKLVLPGGIDTHTHMQ	Negative
3163.	Q9EQF5:88	FMGSQSVDDFYQGTKAALAGGTTMIIDFA	Negative
3164.	Q9EQF5:106	AGGTTMIIDFAIPQKGSSLIEAFETWRNW	Negative
3165.	Q9EQF5:124	LIEAFETWRNWADPKVCCDYSLHVAVTWW	Negative
3166.	Q9EQF5:141	CDYSLHVAVTWWSDKVKEEMKTLARDKGV	Negative
3167.	Q9EQF5:143	YSLHVAVTWWSDKVKEEMKTLARDKGVNS	Negative
3168.	Q9EQF5:147	VAVTWWSDKVKEEMKTLARDKGVNSFKMF	Negative
3169.	Q9EQF5:153	SDKVKEEMKTLARDKGVNSFKMFMAYKGL	Negative
3170.	Q9EQF5:159	EMKTLARDKGVNSFKMFMAYKGLYMVQDE	Negative
3171.	Q9EQF5:165	RDKGVNSFKMFMAYKGLYMVQDEQLYAAF	Negative
3172.	Q9EQF5:183	MVQDEQLYAAFSQCKEIGAIAQVHAENGD	Negative
3173.	Q9EQF5:204	QVHAENGDLIAEGAKKMLALGITGPEGHE	Negative
3174.	Q9EQF5:205	VHAENGDLIAEGAKKMLALGITGPEGHEL	Negative
3175.	Q9EQF5:252	SAVNCPLYVVHVMSKSAAKVVADARRAGN	Negative
3176.	Q9EQF5:335	TTTGSDNCTFNTCQKALGKDDFTKIPNGV	Negative
3177.	Q9EQF5:339	SDNCTFNTCQKALGKDDFTKIPNGVNGVE	Negative
3178.	Q9EQF5:344	FNTCQKALGKDDFTKIPNGVNGVEDRMSV	Negative
3179.	Q9EQF5:362	GVNGVEDRMSVIWEKGVHSGKMDENRFVA	Negative
3180.	Q9EQF5:368	DRMSVIWEKGVHSGKMDENRFVAVTSTNA	Negative
3181.	Q9EQF5:384	DENRFVAVTSTNAAKIFNLYPKKGRIAVG	Negative
3182.	Q9EQF5:391	VTSTNAAKIFNLYPKKGRIAVGSDADIVI	Negative
3183.	Q9EQF5:392	TSTNAAKIFNLYPKKGRIAVGSDADIVIW	Negative
3184.	Q9EQF5:417	IVIWDPEATRRISAKTHHQAVNFNIFEGM	Negative
3185.	Q9EQF5:461	VYEAGVFNVTAGHGKFIPRQPFAEYIYKR	Negative
3186.	Q9EQF5:474	GKFIPRQPFAEYIYKRIKQRDQTCTPVPV	Negative
3187.	Q9EQF5:477	IPRQPFAEYIYKRIKQRDQTCTPVPVKRA	Negative
3188.	Q9EQF5:489	RIKQRDQTCTPVPVKRAPYKGEVTTLKAR	Negative
3189.	Q9EQF5:494	DQTCTPVPVKRAPYKGEVTTLKARETKED	Negative
3190.	Q9EQF5:501	PVKRAPYKGEVTTLKARETKEDDTAGTRM	Negative
3191.	Q9EQF5:506	PYKGEVTTLKARETKEDDTAGTRMQGHSX	Negative
3192.	Q9EQP2:61	EFHSPALEDADFENKPMILLVGQYSTGKT	Negative
3193.	Q9EQP2:74	NKPMILLVGQYSTGKTTFIRYLLEQDFPG	Negative
3194.	Q9EQP2:122	TEGSTPGNALVVDPKKPFRKLSRFGNAFL	Negative
3195.	Q9EQP2:123	EGSTPGNALVVDPKKPFRKLSRFGNAFLN	Negative
3196.	Q9EQP2:127	PGNALVVDPKKPFRKLSRFGNAFLNRFMC	Negative
3197.	Q9EQP2:150	LNRFMCSQLPNQVLKSISIIDSPGILSGE	Negative
3198.	Q9EQP2:165	SISIIDSPGILSGEKQRISRGYDFCQVLQ	Negative
3199.	Q9EQP2:196	AERVDRIILLFDAHKLDISDEFSEAIKAF	Negative
3200.	Q9EQP2:208	AHKLDISDEFSEAIKAFRGQDDKIRVVLN	Negative
3201.	Q9EQP2:216	EFSEAIKAFRGQDDKIRVVLNKADQVDTQ	Negative
3202.	Q9EQP2:223	AFRGQDDKIRVVLNKADQVDTQQLMRVYG	Negative
3203.	Q9EQP2:245	QLMRVYGALMWSLGKVINTPEVLRVYIGS	Negative
3204.	Q9EQP2:291	EAQDLFRDIQSLPQKAAVRKLNDLIKRAR	Negative
3205.	Q9EQP2:296	FRDIQSLPQKAAVRKLNDLIKRARLAKVH	Negative
3206.	Q9EQP2:302	LPQKAAVRKLNDLIKRARLAKVHAYIISY	Negative
3207.	Q9EQP2:308	VRKLNDLIKRARLAKVHAYIISYLKKEMP	Negative

3208.	Q9EQP2:318	ARLAKVHAYIISYLKKEMPNMFGKENKKR	Negative
3209.	Q9EQP2:319	RLAKVHAYIISYLKKEMPNMFGKENKKRE	Negative
3210.	Q9EQP2:327	IISYLKKEMPNMFGKENKKRELIYRLPEI	Negative
3211.	Q9EQP2:330	YLKKEMPNMFGKENKKRELIYRLPEIYVQ	Negative
3212.	Q9EQP2:331	LKKEMPNMFGKENKKRELIYRLPEIYVQL	Negative
3213.	Q9EQP2:360	QREYQISAGDFPEVKAMQEQLENYDFTKF	Negative
3214.	Q9EQP2:373	VKAMQEQLENYDFTKFHSLKPKLIEAVDN	Negative
3215.	Q9EQP2:378	EQLENYDFTKFHSLKPKLIEAVDNMLTNK	Negative
3216.	Q9EQP2:380	LENYDFTKFHSLKPKLIEAVDNMLTNKIS	Negative
3217.	Q9EQP2:392	KPKLIEAVDNMLTNKISSLMGLISQEEMN	Negative
3218.	Q9EQP2:434	TTEGPFNQGYGEGAKEGADEEEWVVAKDK	Negative
3219.	Q9EQP2:446	GAKEGADEEEWVVAKDKPVYDELFYTLSP	Negative
3220.	Q9EQP2:448	KEGADEEEWVVAKDKPVYDELFYTLSPIN	Negative
3221.	Q9EQP2:464	VYDELFYTLSPINGKISGVNAKKEMVTSK	Negative
3222.	Q9EQP2:471	TLSPINGKISGVNAKKEMVTSKLPNSVLG	Negative
3223.	Q9EQP2:472	LSPINGKISGVNAKKEMVTSKLPNSVLGK	Negative
3224.	Q9EQP2:478	KISGVNAKKEMVTSKLPNSVLGKIWKLAD	Negative
3225.	Q9EQP2:486	KEMVTSKLPNSVLGKIWKLADCDCDGMLD	Negative
3226.	Q9EQP2:489	VTSKLPNSVLGKIWKLADCDCDGMLDEEE	Negative
3227.	Q9EQP2:508	DCDGMLDEEEFALAKHLIKIKLDGYELPN	Negative
3228.	Q9EQP2:512	MLDEEEFALAKHLIKIKLDGYELPNSLPP	Negative
3229.	Q9EQP2:514	DEEEFALAKHLIKIKLDGYELPNSLPPHL	Negative
3230.	Q9EQP2:535	PNSLPPHLVPPSHRKSLPKADXXXXXXXX	Negative
3231.	Q9EQP2:539	PPHLVPPSHRKSLPKADXXXXXXXXXXXX	Negative
3232.	Q9H1E3:9	XXXXXXMSRPVRNRKVVDYSQFQESDDAD	Negative
3233.	Q9H1E3:35	DADEDYGRDSGPPTKKIRSSPREAKNKRR	Negative
3234.	Q9H1E3:36	ADEDYGRDSGPPTKKIRSSPREAKNKRRS	Negative
3235.	Q9H1E3:45	GPPTKKIRSSPREAKNKRRSGKNSQEDSE	Negative
3236.	Q9H1E3:47	PTKKIRSSPREAKNKRRSGKNSQEDSEDS	Negative
3237.	Q9H1E3:52	RSSPREAKNKRRSGKNSQEDSEDSEDKDV	Negative
3238.	Q9H1E3:64	SGKNSQEDSEDSEDKDVKTKKDDSHSAED	Negative
3239.	Q9H1E3:67	NSQEDSEDSEDKDVKTKKDDSHSAEDSED	Negative
3240.	Q9H1E3:69	QEDSEDSEDKDVKTKKDDSHSAEDSEDEK	Negative
3241.	Q9H1E3:70	EDSEDSEDKDVKTKKDDSHSAEDSEDEKE	Negative
3242.	Q9H1E3:83	KKDDSHSAEDSEDEKEDHKNVRQQRQAAS	Negative
3243.	Q9H1E3:87	SHSAEDSEDEKEDHKNVRQQRQAASKAAS	Negative
3244.	Q9H1E3:98	EDHKNVRQQRQAASKAASKQREMLMEDVG	Negative
3245.	Q9H1E3:102	NVRQQRQAASKAASKQREMLMEDVGSEEE	Negative
3246.	Q9H1E3:128	EEEQEEEDEAPFQEKDSGSDEDFLMEDDD	Negative
3247.	Q9H1E3:150	FLMEDDDDSDYGSSKKKNKKMVKKSKPER	Negative
3248.	Q9H1E3:151	LMEDDDDSDYGSSKKKNKKMVKKSKPERK	Negative
3249.	Q9H1E3:152	MEDDDDSDYGSSKKKNKKMVKKSKPERKE	Negative
3250.	Q9H1E3:154	DDDDSDYGSSKKKNKKMVKKSKPERKEKK	Negative
3251.	Q9H1E3:155	DDDSDYGSSKKKNKKMVKKSKPERKEKKM	Negative
3252.	Q9H1E3:158	SDYGSSKKKNKKMVKKSKPERKEKKMPKP	Negative
3253.	Q9H1E3:159	DYGSSKKKNKKMVKKSKPERKEKKMPKPR	Negative
3254.	Q9H1E3:161	GSSKKKNKKMVKKSKPERKEKKMPKPRLK	Negative
3255.	Q9H1E3:165	KKNKKMVKKSKPERKEKKMPKPRLKATVT	Negative
3256.	Q9H1E3:167	NKKMVKKSKPERKEKKMPKPRLKATVTPS	Negative
3257.	Q9H1E3:168	KKMVKKSKPERKEKKMPKPRLKATVTPSP	Negative
3258.	Q9H1E3:171	VKKSKPERKEKKMPKPRLKATVTPSPVKG	Negative
3259.	Q9H1E3:175	KPERKEKKMPKPRLKATVTPSPVKGKGKV	Negative
3260.	Q9H1E3:184	PKPRLKATVTPSPVKGKGKVGRPTASKAS	Negative
3261.	Q9H1E3:186	PRLKATVTPSPVKGKGKVGRPTASKASKE	Negative

3262.	Q9H1E3:188	LKATVTPSPVKGKGKVGRPTASKASKEKT	Negative
3263.	Q9H1E3:196	PVKGKGKVGRPTASKASKEKTPSPKEEDE	Negative
3264.	Q9H1E3:199	GKGKVGRPTASKASKEKTPSPKEEDEEPE	Negative
3265.	Q9H1E3:201	GKVGRPTASKASKEKTPSPKEEDEEPESP	Negative
3266.	Q9H1E3:206	PTASKASKEKTPSPKEEDEEPESPPEKKT	Negative
3267.	Q9H1E3:218	SPKEEDEEPESPPEKKTSTSPPPEKSGDE	Negative
3268.	Q9H1E3:219	PKEEDEEPESPPEKKTSTSPPPEKSGDEG	Negative
3269.	Q9QXX4:5	XXXXXXXXXXMAAAKVALTKRADPAELKA	Negative
3270.	Q9QXX4:10	XXXXXMAAAKVALTKRADPAELKAIFLKY	Negative
3271.	Q9QXX4:18	AKVALTKRADPAELKAIFLKYASIEKNGE	Negative
3272.	Q9QXX4:23	TKRADPAELKAIFLKYASIEKNGEFFMSP	Negative
3273.	Q9QXX4:29	AELKAIFLKYASIEKNGEFFMSPHDFVTR	Negative
3274.	Q9QXX4:56	TRYLNIFGESQPNPKTVELLSGVVDQTKD	Negative
3275.	Q9QXX4:69	PKTVELLSGVVDQTKDGLISFQEFVAFES	Negative
3276.	Q9QXX4:101	APDALFMVAFQLFDKAGKGEVTFEDVKQI	Negative
3277.	Q9QXX4:104	ALFMVAFQLFDKAGKGEVTFEDVKQIFGQ	Negative
3278.	Q9QXX4:113	FDKAGKGEVTFEDVKQIFGQTTIHQHIPF	Negative
3279.	Q9QXX4:140	PFNWDSEFVQLHFGKERKRHLTYAEFTQF	Negative
3280.	Q9QXX4:143	WDSEFVQLHFGKERKRHLTYAEFTQFLLE	Negative
3281.	Q9QXX4:164	EFTQFLLEIQLEHAKQAFVQRDNAKTGKV	Negative
3282.	Q9QXX4:174	LEHAKQAFVQRDNAKTGKVSAIDFRDIMV	Negative
3283.	Q9QXX4:235	NGFNSLLNNMELIRKIYSTLAGNRKDVEV	Negative
3284.	Q9QXX4:245	ELIRKIYSTLAGNRKDVEVTKEEFALAAQ	Negative
3285.	Q9QXX4:251	YSTLAGNRKDVEVTKEEFALAAQKFGQVT	Negative
3286.	Q9QXX4:260	DVEVTKEEFALAAQKFGQVTPMEVDILFQ	Negative
3287.	Q9QXX4:313	GMLPFNLAEAQRQQKASGDAARPFLLQLA	Negative
3288.	Q9QXX4:354	GAVGATAVYPIDLVKTRMQNQRSTGSFVG	Negative
3289.	Q9QXX4:373	NQRSTGSFVGELMYKNSFDCFKKVLRYEG	Negative
3290.	Q9QXX4:380	FVGELMYKNSFDCFKKVLRYEGFFGLYRG	Negative
3291.	Q9QXX4:381	VGELMYKNSFDCFKKVLRYEGFFGLYRGL	Negative
3292.	Q9QXX4:406	YRGLLPQLLGVAPEKAIKLTVNDFVRDKF	Negative
3293.	Q9QXX4:409	LLPQLLGVAPEKAIKLTVNDFVRDKFMHK	Negative
3294.	Q9QXX4:419	EKAIKLTVNDFVRDKFMHKDGSVPLLAEI	Negative
3295.	Q9QXX4:423	KLTVNDFVRDKFMHKDGSVPLLAEIFAGG	Negative
3296.	Q9QXX4:454	GGSQVIFTNPLEIVKIRLQVAGEITTGPR	Negative
3297.	Q9QXX4:485	ALSVVRDLGFFGIYKGAKACFLRDIPFSA	Negative
3298.	Q9QXX4:488	VVRDLGFFGIYKGAKACFLRDIPFSAIYF	Negative
3299.	Q9QXX4:509	IPFSAIYFPCYAHVKASFANEDGQVSPGS	Negative
3300.	Q9QXX4:546	GMPAASLVTPADVIKTRLQVAARAGQTTY	Negative
3301.	Q9QXX4:569	AGQTTYNGVTDCFRKILREEGPKALWKGV	Negative
3302.	Q9QXX4:577	VTDCFRKILREEGPKALWKGVAARVFRSS	Negative
3303.	Q9QXX4:581	FRKILREEGPKALWKGVAARVFRSSPQFG	Negative
3304.	Q9QXX4:616	ELLQRWFYVDFGGVKPVGSEPVPKSRITL	Negative
3305.	Q9QXX4:625	DFGGVKPVGSEPVPKSRITLPAPNPDHVG	Negative
3306.	Q9QXX4:642	ITLPAPNPDHVGGYKLAVATFAGIENKFG	Negative
3307.	Q9QXX4:654	GYKLAVATFAGIENKFGLYLPLFKPSAST	Negative
3308.	Q9QXX4:663	AGIENKFGLYLPLFKPSASTSKVTAGDSX	Negative
3309.	Q9QXX4:670	GLYLPLFKPSASTSKVTAGDSXXXXXXXX	Negative
3310.	Q9Y597:81	DPAAFAPILNFLRTKELDLRGVSINVLRH	Negative
3311.	Q9Y597:138	LFHGYLPPPGIPSRKINNTVRSADSRNGL	Negative
3312.	Q9Y597:186	GEETVRLGFPVDPRKVLIVAGHHNWIVAA	Negative
3313.	Q9Y597:211	IVAAYAHFAVCYRIKESSGWQQVFTSPYL	Negative
3314.	Q9Y597:237	PYLDWTIERVALNAKVVGGPHGDKDKMVA	Negative
3315.	Q9Y597:246	VALNAKVVGGPHGDKDKMVAVASESSIIL	Negative

3316.	Q9Y597:248	LNAKVVGGPHGDKDKMVAVASESSIILWS	Negative
3317.	Q9Y597:299	FFIGNQLVATSHTGKVGVWNAVTQHWQVQ	Negative
3318.	Q9Y597:344	LGCNNGSIYYIDMQKFPLRMKDNDLLVTE	Negative
3319.	Q9Y597:350	SIYYIDMQKFPLRMKDNDLLVTELYHDPS	Negative
3320.	Q9Y597:378	SNDAITALSVYLTPKTSVSGNWIEIAYGT	Negative
3321.	Q9Y597:426	QLFQTFTVHRSPVTKIMLSEKHLVSVCAD	Negative
3322.	Q9Y597:432	TVHRSPVTKIMLSEKHLVSVCADNNHVRT	Negative
3323.	Q9Y597:469	MISTQPGSTPLASFKILSLEETESHGSYS	Negative
3324.	Q9Y597:503	GPFGERDDQQVFIQKVVPITNKLFVRLSS	Negative
3325.	Q9Y597:510	DQQVFIQKVVPITNKLFVRLSSTGKRICE	Negative
3326.	Q9Y597:520	PITNKLFVRLSSTGKRICEIQAVDCTTIS	Negative
3327.	Q9Y597:578	IQMWDLTTAMDMVNKSEDKDVGGPTEEEL	Negative
3328.	Q9Y597:582	DLTTAMDMVNKSEDKDVGGPTEEELLKLL	Negative
3329.	Q9Y597:594	EDKDVGGPTEEELLKLLDQCDLSTSRCAT	Negative
3330.	Q9Y597:698	VPENGNLGPIQAEVKGATGECNISERKSP	Negative
3331.	Q9Y597:710	EVKGATGECNISERKSPGVEIKSLRELDS	Negative
3332.	Q9Y597:717	ECNISERKSPGVEIKSLRELDSGLEVHKI	Negative
3333.	Q9Y597:730	IKSLRELDSGLEVHKIAEGFSESKKRSSE	Negative
3334.	Q9Y597:739	GLEVHKIAEGFSESKKRSSEDENENKIEF	Negative
3335.	Q9Y597:740	LEVHKIAEGFSESKKRSSEDENENKIEFR	Negative
3336.	Q9Y597:750	SESKKRSSEDENENKIEFRKKGGFEGGGF	Negative
3337.	Q9Y597:755	RSSEDENENKIEFRKKGGFEGGGFLGRKK	Negative
3338.	Q9Y597:756	SSEDENENKIEFRKKGGFEGGGFLGRKKV	Negative
3339.	Q9Y597:768	RKKGGFEGGGFLGRKKVPYLASSPSTSDG	Negative
3340.	Q9Y597:769	KKGGFEGGGFLGRKKVPYLASSPSTSDGG	Negative
3341.	Q9Y597:804	ASPSPTKTTPSPRHKKSDSSGQEYSLXXX	Negative
3342.	Q9Y597:805	SPSPTKTTPSPRHKKSDSSGQEYSLXXXX	Negative
3343.	Q9Y619:2	XXXXXXXXXXXXMKSNPAIQAAIDLTAG	Negative
3344.	Q9Y619:34	GTACVLTGQPFDTMKVKMQTFPDLYRGLT	Negative
3345.	Q9Y619:36	ACVLTGQPFDTMKVKMQTFPDLYRGLTDC	Negative
3346.	Q9Y619:53	TFPDLYRGLTDCCLKTYSQVGFRGFYKGT	Negative
3347.	Q9Y619:65	CLKTYSQVGFRGFYKGTSPALIANIAENS	Negative
3348.	Q9Y619:94	VLFMCYGFCQQVVRKVAGLDKQAKLSDLQ	Negative
3349.	Q9Y619:100	GFCQQVVRKVAGLDKQAKLSDLQNAAAGS	Negative
3350.	Q9Y619:103	QQVVRKVAGLDKQAKLSDLQNAAAGSFAS	Negative
3351.	Q9Y619:131	SAFAALVLCPTELVKCRLQTMYEMETSGK	Negative
3352.	Q9Y619:148	LQTMYEMETSGKIAKSQNTVWSVIKSILR	Negative
3353.	Q9Y619:158	GKIAKSQNTVWSVIKSILRKDGPLGFYHG	Negative
3354.	Q9Y619:163	SQNTVWSVIKSILRKDGPLGFYHGLSSTL	Negative
3355.	Q9Y619:204	GYELSRSFFASGRSKDELGPVPLMLSGGV	Negative
3356.	Q9Y619:234	GICLWLAVYPVDCIKSRIQVLSMSGKQAG	Negative
3357.	Q9Y619:245	DCIKSRIQVLSMSGKQAGFIRTFINVVKN	Negative
3358.	Q9Y619:258	GKQAGFIRTFINVVKNEGITALYSGLKPT	Negative
3359.	Q9Y619:270	VVKNEGITALYSGLKPTMIRAFPANGALF	Negative
3360.	Q9Y619:292	PANGALFLAYEYSRKLMMNQLEAYXXXXX	Negative
