# Supplementary material for: predPhogly-Site: Predicting phosphoglycerylation sites by incorporating probabilistic sequence-coupling information into PseAAC and addressing data imbalance
Source: PLoS One. 2021 Apr 1;16(4):e0249396. doi: 10.1371/journal.pone.0249396 (PMC8016359; doi:10.1371/journal.pone.0249396)
Supplement: S2 File — Proteins which have been recently added to the PLMD database and completely unknown to the proposed system. (PDF) [file pone.0249396.s002.pdf]

S2 File: Proteins in Independent Test Set

>sp|O35215|Modified_Residue:110
MPFVELETNLPASRIPAGLENRLCAATATILDKPEDRVSVTIRPGMTLLMNKSTEPCAHLLVSSIGVVGT
AEQNRTHSASFFKFLTEELSLDQDRIVIRFFPLEAWQIGKKGTVMTFL

>sp|O55143|Modified_Residue:205
MENAHTKTVEEVLGHFGVNESTGLSLEQVKKLKERWGSNELPAEEGKTLLELVIEQFEDLLVRILLLAAC
ISFVLAWFEEGEETITAFVEPFVILLILVANAIVGVWQERNAENAIEALKEYEPEMGKVYRQDRKSVQRI
KAKDIVPGDIVEIAVGDKVPADIRLTSIKSTTLRVDQSILTGESVSVIKHTDPVPDPRAVNQDKKNMLFS
GTNIAAGKAMGVVVATGVNTEIGKIRDEMVATEQERTPLQQKLDEFGEQLSKVISLICIAVWIINIGHFN
DPVHGGSWIRGAIYYFKIAVALAVAAIPEGLPAVITTCLALGTRRMAKKNAIVRSLPSVETLGCTSVICS
DKTGTLTTNQMSVCRMFILDKVEGDTCSLNEFSITGSTYAPIGEVQKDDKPVKCHQYDGLVELATICALC
NDSALDYNEAKGVYEKVGEATETALTCLVEKMNVFDTELKGLSKIERANACNSVIKQLMKKEFTLEFSRD
RKSMSVYCTPNKPSRTSMSKMFVKGAPEGVIDRCTHIRVGSTKVPMTPGVKQKIMSVIREWGSGSDTLRC
LALATHDNPLKREEMHLEDSANFIKYETNLTFVGCVGMLDPPRIEVASSVKLCRQAGIRVIMITGDNKGT
AVAICRRIGIFGQDEDVTSKAFTGREFDELSPSAQRDACLNARCFARVEPSHKSKIVEFLQSFDEITAMT
GDGVNDAPALKKSEIGIAMGSGTAVAKTASEMVLADDNFSTIVAAVEEGRAIYNNMKQFIRYLISSNVGE
VVCIFLTAALGFPEALIPVQLLWVNLVTDGLPATALGFNPPDLDIMNKPPRNPKEPLISGWLFFRYLAIG
CYVGAATVGAAAWWFIAADGGPRVSFYQLSHFLQCKEDNPDFDGVDCAIFESPYPMTMALSVLVTIEMCN
ALNSLSENQSLLRMPPWENIWLVGSICLSMSLHFLILYVEPLPLIFQITPLNLTQWLMVLKISLPVILMD
ETLKFVARNYLEQPGKECVQPATKSSCSLSACTDGISWPFVLLIMPLVVWVYSTDTNFSDMFWS

>sp|O70133|Modified_Residue:1026
MGDIKNFLYAWCGKRKMTPAYEIRAVGNKNRQKFMCEVRVEGFNYAGMGNSTNKKDAQSNAARDFVNYLV
RINEVKSEEVPAVGIVPPPPILSDTSDSTASAAEGLPAPMGGPLPPHLALKAEENNSGVESSGYGSPGPT
WDRGANLKDYYSRKEEQEVQATLESEEVDLNAGLHGNWTLENAKARLNQYFQKEKIQGEYKYTQVGPDHN
RSFIAEMTIYIKQLGRRIFAREHGSNKKLAAQSCALSLVRQLYHLGVIEAYSGLTKKKEGERVEPYKVFL
SPDLELQLQNVVQELDLEIVPPPVDPSMPVILNIGKLAHFEPSQRQNAVGVVPWSPPQSNWNPWTSSNID
EGPLAYASTEQISMDLKNELTYQMEQDHNLQSVLQERELLPVKKFEAEILEAISSNSVVIIRGATGCGKT
TQVPQYILDDFIQNDRAAECNIVVTQPRRISAVAVAERVAYERGEEPGKSCGYSVRFESILPRPHASIMF
CTVGVLLRKLEAGIRGISHVIVDEIHERDINTDFLLVVLRDVVLAYPEVRIVLMSATIDTTMFCEYFFNC
PIIEVYGRTFPVQEYFLEDCIQMTQFIPPPKDKKKKDKEDDGGEDDDANCNLICGDEYGPETKLSMSQLN
EKETPFELIEALLKYIETLNVPGAVLVFLPGWNLIYTMQKHLENNSHFGSHRYQILPLHSQIPREEQRKV
FDPVPDGVTKVILSTNIAETSITINDVVYVIDSCKQKVKLFTAHNNMTNYATVWASKTNLEQRKGRAGRV
RPGFCFHLCSRARFDRLETHMTPEMFRTPLHEIALSIKLLRLGGIGQFLAKAIEPPPLDAIIEAEHTLRE
LDALDANDELTPLGRILAKLPIEPRFGKMMIMGCIFYVGDAVCTISAATCFPEPFISEGKRLGYIHRNFA
GNRFSDHVALLSVFQAWDDARMSGEEAEIRFCEQKRLNMATLRMTWEAKVQLKEILINSGFPEDCLLTQV
FTNTGPDNNLDVVISLLAFGVYPNVCYHKEKRKILTTEGRNALIHKSSVNCPFSSQDMKYPSPFFVFGEK
IRTRAISAKGMTLVTPLQLLLFASKKVQSDGQIVFIDDWIRLQISHEAAACITIRAAMEALVVEVSKQPN
IISQLDPVNEHMLNTIRQISRPSAAGINLMIGSVRYGDGPRPPKMARYDNGSGYRRGYGGGGYGGGGYGG
GYGSGGFGGGFGSGGGFGGGFNSGGGGFGSGGGGFGSGGGGFGGGGGGFSGGGGGGFGGGRGGGGGGFGG
SGGFGNGGGGYGVGGGGYGGGGGGGYGGGSGGYGGGGYGGGEGYSISPNSYRGNYGGGGGGYRGGSQGGY
RNNFGGDYRGSSGDYRGSGGGYRGSGGFQRRGYGGGYFGQGRGGGGGGGY

>sp|O70228|Modified_Residue:132
MTDSIPLQPVRHKKRVDSRPRAGCCEWLRCCGGGEPRPRTVWLGHPEKRDQRYPRNVINNQKYNFFTFLP
GVLFSQFRYFFNFYFLLLACSQFVPEMRLGALYTYWVPLGFVLAVTIIREAVEEIRCYVRDKEMNSQVYS
RLTSRGTVKVKSSNIQVGDLILVEKNQRVPADMIFLRTSEKNGSCFLRTDQLDGETDWKLRLPVACTQRL
PTAADLLQIRSYVYAEEPNIDIHNFLGTFTREDSDPPISESLSIENTLWAGTVIASGTVVGVVLYTGREL
RSVMNTSDPRSKIGLFDLEVNCLTKILFGALVVVSLVMVALQHFAGRWYLQIIRFLLLFSNIIPISLRVN
LDMGKIVYSWVIRRDSKIPGTVVRSSTIPEQLGRISYLLTDKTGTLTQNEMVFKRLHLGTVAYGLDSMDE
VQSHIFSIYTQQSQDPPAQKGPTVTTKVRRTMSSRVHEAVKAIALCHNVTPVYESNGVTDQAEAEKQFED
SCRVYQASSPDEVALVQWTESVGLTLVGRDQSSMQLRTPGDQVLNLTILQVFPFTYESKRMGIIVRDEST

GEITFYMKGADVVMAGIVQYNDWLEEECGNMAREGLRVLVVAKKSLTEEQYQDFEARYVQAKLSVHDRSL
KVATVIESLEMEMELLCLTGVEDQLQADVRPTLETLRNAGIKVWMLTGDKLETATCTAKNAHLVTRNQDI
HVFRLVTNRGEAHLELNAFRRKHDCALVISGDSLEVCLKYYEYEFMELACQCPAVVCCRCAPTQKAQIVR
LLQERTGKLTCAVGDGGNDVSMIQESDCGVGVEGKEGKQASLAADFSITQFKHLGRLLMVHGRNSYKRSA
ALSQFVIHRSLCISTMQAVFSSVFYFASVPLYQGFLIIGYSTIYTMFPVFSLVLDKDVKSEVAMLYPELY
KDLLKGRPLSYKTFLIWVLISIYQGSTIMYGALLLFESEFVHIVAISFTSLILTELLMVALTIQTWHWLM
TVAELLSLACYIASLVFLHEFIDVYFIATLSFLWKVSVITLVSCLPLYVLKYLRRRFSPPSYSKLTS

>sp|P05063|Modified_Residue:200
MPHSYPALSAEQKKELSDIALRIVTPGKGILAADESVGSMAKRLSQIGVENTEENRRLYRQVLFSADDRV
KKCIGGVIFFHETLYQKDDNGVPFVRTIQDKGILVGIKVDKGVVPLAGTDGETTTQGLDGLLERCAQYKK
DGADFAKWRCVLKISDRTPSALAILENANVLARYASICQQNGIVPIVEPEILPDGDHDLKRCQYVTEKVL
AAVYKALSDHHVYLEGTLLKPNMVTPGHACPIKYSPEEIAMATVTALRRTVPPAVPGVTFLSGGQSEEEA
SLNLNAINRCPLPRPWALTFSYGRALQASALNAWRGQRDNAGAATEEFIKRAEMNGLAAQGRYEGSGDGG
AAAQSLYIANHAY

>sp|P05387|Modified_Residue:99
MRYVASYLLAALGGNSSPSAKDIKKILDSVGIEADDDRLNKVISELNGKNIEDVIAQGIGKLASVPAGGA
VAVSAAPGSAAPAAGSAPAAAEEKKDEKKEESEESDDDMGFGLFD

>sp|P0DMV8|Modified_Residue:597
MAKAAAIGIDLGTTYSCVGVFQHGKVEIIANDQGNRTTPSYVAFTDTERLIGDAAKNQVALNPQNTVFDA
KRLIGRKFGDPVVQSDMKHWPFQVINDGDKPKVQVSYKGETKAFYPEEISSMVLTKMKEIAEAYLGYPVT
NAVITVPAYFNDSQRQATKDAGVIAGLNVLRIINEPTAAAIAYGLDRTGKGERNVLIFDLGGGTFDVSIL
TIDDGIFEVKATAGDTHLGGEDFDNRLVNHFVEEFKRKHKKDISQNKRAVRRLRTACERAKRTLSSSTQA
SLEIDSLFEGIDFYTSITRARFEELCSDLFRSTLEPVEKALRDAKLDKAQIHDLVLVGGSTRIPKVQKLL
QDFFNGRDLNKSINPDEAVAYGAAVQAAILMGDKSENVQDLLLLDVAPLSLGLETAGGVMTALIKRNSTI
PTKQTQIFTTYSDNQPGVLIQVYEGERAMTKDNNLLGRFELSGIPPAPRGVPQIEVTFDIDANGILNVTA
TDKSTGKANKITITNDKGRLSKEEIERMVQEAEKYKAEDEVQRERVSAKNALESYAFNMKSAVEDEGLKG
KISEADKKKVLDKCQEVISWLDANTLAEKDEFEHKRKELEQVCNPIISGLYQGAGGPGPGGFGAQGPKGG
SGSGPTIEEVD

>sp|P14618|Modified_Residue:367
MSKPHSEAGTAFIQTQQLHAAMADTFLEHMCRLDIDSPPITARNTGIICTIGPASRSVETLKEMIKSGMN
VARLNFSHGTHEYHAETIKNVRTATESFASDPILYRPVAVALDTKGPEIRTGLIKGSGTAEVELKKGATL
KITLDNAYMEKCDENILWLDYKNICKVVEVGSKIYVDDGLISLQVKQKGADFLVTEVENGGSLGSKKGVN
LPGAAVDLPAVSEKDIQDLKFGVEQDVDMVFASFIRKASDVHEVRKVLGEKGKNIKIISKIENHEGVRRF
DEILEASDGIMVARGDLGIEIPAEKVFLAQKMMIGRCNRAGKPVICATQMLESMIKKPRPTRAEGSDVAN
AVLDGADCIMLSGETAKGDYPLEAVRMQHLIAREAEAAIYHLQLFEELRRLAPITSDPTEATAVGAVEAS
FKCCSGAIIVLTKSGRSAHQVARYRPRAPIIAVTRNPQTARQAHLYRGIFPVLCKDPVQEAWAEDVDLRV
NFAMNVGKARGFFKKGDVVIVLTGWRPGSGFTNTMRVVPVP

>sp|P14824|Modified_Residue:442,445,446
MAKIAQGAMYRGSVHDFPEFDANQDAEALYTAMKGFGSDKESILELITSRSNKQRQEICQNYKSLYGKDL
IEDLKYELTGKFERLIVNLMRPLAYCDAKEIKDAISGVGTDEKCLIEILASRTNEQMHQLVAAYKDAYER
DLESDIIGDTSGHFQKMLVVLLQGTRENDDVVSEDLVQQDVQDLYEAGELKWGTDEAQFIYILGNRSKQH
LRLVFDEYLKTTGKPIEASIRGELSGDFEKLMLAVVKCIRSTPEYFAERLFKAMKGLGTRDNTLIRIMVS
RSELDMLDIREIFRTKYEKSLYSMIKNDTSGEYKKALLKLCGGDDDAAGQFFPEAAQVAYQMWELSAVSR
VELKGTVCAANDFNPDADAKALRKAMKGIGTDEATIIDIVTHRSNAQRQQIRQTFKSHFGRDLMADLKSE
ISGDLARLILGLMMPPAHYDAKQLKKAMEGAGTDEKTLIEILATRTNAEIRAINEAYKEDYHKSLEDALS
SDTSGHFRRILISLATGNREEGGENRDQAQEDAQVAAEILEIADTPSGDKTSLETRFMTVLCTRSYPHLR
RVFQEFIKKTNYDIEHVIKKEMSGDVKDAFVAIVQSVKNKPLFFADKLYKSMKGAGTDEKTLTRVMVSRS
EIDLLNIRREFIEKYDKSLHQAIEGDTSGDFMKALLALCGGED

>sp|P14869|Modified_Residue:297
MPREDRATWKSNYFLKIIQLLDDYPKCFIVGADNVGSKQMQQIRMSLRGKAVVLMGKNTMMRKAIRGHLE
NNPALEKLLPHIRGNVGFVFTKEDLTEIRDMLLANKVPAAARAGAIAPCEVTVPAQNTGLGPEKTSFFQA
LGITTKISRGTIEILSDVQLIKTGDKVGASEATLLNMLNISPFSFGLIIQQVFDNGSIYNPEVLDITEQA
LHSRFLEGVRNVASVCLQIGYPTVASVPHSIINGYKRVLALSVETEYTFPLTEKVKAFLADPSAFAAAAP
AAAATTAAPAAAAAPAKAEAKEESEESDEDMGFGLFD

>sp|P16460|Modified_Residue:340
MSSKGSVVLAYSGGLDTSCILVWLKEQGYDVIAYLANIGQKEDFEEARKKALKLGAKKVFIEDVSKEFVE
EFIWPAVQSSALYEDRYLLGTSLARPCIARRQVEIAQREGAKYVSHGATGKGNDQVRFELTCYSLAPQIK
VIAPWRMPEFYNRFKGRNDLMEYAKQHGIPIPVTPKSPWSMDENLMHISYEAGILENPKNQAPPGLYTKT
QDPAKAPNSPDVLEIEFKKGVPVKVTNIKDGTTRTTSLELFMYLNEVAGKHGVGRIDIVENRFIGMKSRG
IYETPAGTILYHAHLDIEAFTMDREVRKIKQGLGLKFAELVYTGFWHSPECEFVRHCIQKSQERVEGKVQ
VSVFKGQVYILGRESPLSLYNEELVSMNVQGDYEPIDATGFININSLRLKEYHRLQSKVTAK

>sp|P16858|Modified_Residue:143,213,217,225,261
MVKVGVNGFGRIGRLVTRAAICSGKVEIVAINDPFIDLNYMVYMFQYDSTHGKFNGTVKAENGKLVINGK
PITIFQERDPTNIKWGEAGAEYVVESTGVFTTMEKAGAHLKGGAKRVIISAPSADAPMFVMGVNHEKYDN
SLKIVSNASCTTNCLAPLAKVIHDNFGIVEGLMTTVHAITATQKTVDGPSGKLWRDGRGAAQNIIPASTG
AAKAVGKVIPELNGKLTGMAFRVPTPNVSVVDLTCRLEKPAKYDDIKKVVKQASEGPLKGILGYTEDQVV
SCDFNSNSHSSTFDAGAGIALNDNFVKLISWYDNEYGYSNRVVDLMAYMASKE

>sp|P17036|Modified_Residue:23
METQADLVSQEPQALLDSALPSKVPAFSDKDSLGDEMLAAALLKAKSQELVTFEDVAVYFIRKEWKRLEP
AQRDLYRDVMLENYGNVFSLDRETRTENDQEISEDTRSHGVLLGRFQKDISQGLKFKEAYEREVSLKRPL
GNSPGERLNRKMPDFGQVTVEEKLTPRGERSEKYNDFGNSFTVNSNLISHQRLPVGDRPHKCDECSKSFN
RTSDLIQHQRIHTGEKPYECNECGKAFSQSSHLIQHQRIHTGEKPYECSDCGKTFSCSSALILHRRIHTG
EKPYECNECGKTFSWSSTLTHHQRIHTGEKPYACNECGKAFSRSSTLIHHQRIHTGEKPYECNECGKAFS
QSSHLYQHQRIHTGEKPYECMECGGKFTYSSGLIQHQRIHTGENPYECSECGKAFRYSSALVRHQRIHTG
EKPLNGIGMSKSSLRVTTELNIREST

>sp|P17183|Modified_Residue:343
MSIEKIWAREILDSRGNPTVEVDLYTAKGLFRAAVPSGASTGIYEALELRDGDKQRYLGKGVLKAVDHIN
SRIAPALISSGISVVEQEKLDNLMLELDGTENKSKFGANAILGVSLAVCKAGAAERDLPLYRHIAQLAGN
SDLILPVPAFNVINGGSHAGNKLAMQEFMILPVGAESFRDAMRLGAEVYHTLKGVIKDKYGKDATNVGDE
GGFAPNILENSEALELVKEAIDKAGYTEKMVIGMDVAASEFYRDGKYDLDFKSPADPSRYITGDQLGALY
QDFVRNYPVVSIEDPFDQDDWAAWSKFTANVGIQIVGDDLTVTNPKRIERAVEEKACNCLLLKVNQIGSV
TEAIQACKLAQENGWGVMVSHRSGETEDTFIADLVVGLCTGQIKTGAPCRSERLAKYNQLMRIEEELGDE
ARFAGHNFRNPSVL

>sp|P17844|Modified_Residue:91
MSGYSSDRDRGRDRGFGAPRFGGSRAGPLSGKKFGNPGEKLVKKKWNLDELPKFEKNFYQEHPDLARRTA
QEVETYRRSKEITVRGHNCPKPVLNFYEANFPANVMDVIARQNFTEPTAIQAQGWPVALSGLDMVGVAQT
GSGKTLSYLLPAIVHINHQPFLERGDGPICLVLAPTRELAQQVQQVAAEYCRACRLKSTCIYGGAPKGPQ
IRDLERGVEICIATPGRLIDFLECGKTNLRRTTYLVLDEADRMLDMGFEPQIRKIVDQIRPDRQTLMWSA
TWPKEVRQLAEDFLKDYIHINIGALELSANHNILQIVDVCHDVEKDEKLIRLMEEIMSEKENKTIVFVET
KRRCDELTRKMRRDGWPAMGIHGDKSQQERDWVLNEFKHGKAPILIATDVASRGLDVEDVKFVINYDYPN
SSEDYIHRIGRTARSTKTGTAYTFFTPNNIKQVSDLISVLREANQAINPKLLQLVEDRGSGRSRGRGGMK
DDRRDRYSAGKRGGFNTFRDRENYDRGYSSLLKRDFGAKTQNGVYSAANYTNGSFGSNFVSAGIQTSFRT
GNPTGTYQNGYDSTQQYGSNVPNMHNGMNQQAYAYPATAAAPMIGYPMPTGYSQ

>sp|P31786|Modified_Residue:55
MSQAEFDKAAEEVKRLKTQPTDEEMLFIYSHFKQATVGDVNTDRPGLLDLKGKAKWDSWNKLKGTSKESA
MKTYVEKVDELKKKYGI

>sp|P41216|Modified_Residue:544,552
MEVHELFRYFRMPELIDIRQYVRTLPTNTLMGFGAFAALTTFWYATRPKALKPPCDLSMQSVEIAGTTDG
IRRSAVLEDDKLLVYYYDDVRTMYDGFQRGIQVSNNGPCLGSRKPNQPYEWISYKEVAELAECIGSGLIQ
KGFKPCSEQFIGLFSQNRPEWVIVEQGCFSYSMVVVPLYDTLGADAITYIVNKAELSVIFADKPEKAKLL
LEGVENKLTPCLKIIVIMDSYGSDLVERGKKCGVEIISLKALEDLGRVNRVKPKPPEPEDLAIICFTSGT
TGNPKGAMITHQNIINDCSGFIKATESAFIASTDDVLISFLPLAHMFETVVECVMLCHGAKIGFFQGDIR
LLMDDLKVLQPTIFPVVPRLLNRMFDRIFGQANTSLKRWLLDFASKRKEAELRSGIVRNNSLWDKLIFHK
IQSSLGGKVRLMITGAAPVSATVLTFLRTALGCQFYEGYGQTECTAGCCLSLPGDWTAGHVGAPMPCNYV
KLVDVEEMNYLASKGEGEVCVKGANVFKGYLKDPARTAEALDKDGWLHTGDIGKWLPNGTLKIIDRKKHI
FKLAQGEYIAPEKIENIYLRSEAVAQVFVHGESLQAFLIAVVVPDVESLPSWAQKRGLQGSFEELCRNKD
INKAILDDLLKLGKEAGLKPFEQVKGIAVHPELFSIDNGLLTPTLKAKRPELRNYFRSQIDELYATIKI

>sp|P47955|Modified_Residue:98
MASVSELACIYSALILHDDEVTVTEDKINALIKAAGVSVEPFWPGLFAKALANVNIGSLICNVGAGGPAP
AAGAAPAGGAAPSTAAAPAEEKKVEAKKEESEESEDDMGFGLFD

>sp|P61604|Modified_Residue:56
MAGQAFRKFLPLFDRVLVERSAAETVTKGGIMLPEKSQGKVLQATVVAVGSGSKGKGGEIQPVSVKVGDK
VLLPEYGGTKVVLDDKDYFLFRDGDILGKYVD

>sp|P61982|Modified_Residue:50
MVDREQLVQKARLAEQAERYDDMAAAMKNVTELNEPLSNEERNLLSVAYKNVVGARRSSWRVISSIEQKT
SADGNEKKIEMVRAYREKIEKELEAVCQDVLSLLDNYLIKNCSETQYESKVFYLKMKGDYYRYLAEVATG
EKRATVVESSEKAYSEAHEISKEHMQPTHPIRLGLALNYSVFYYEIQNAPEQACHLAKTAFDDAIAELDT
LNEDSYKDSTLIMQLLRDNLTLWTSDQQDDDGGEGNN

>sp|P97351|Modified_Residue:144
MAVGKNKRLTKGGKKGAKKKVVDPFSKKDWYDVKAPAMFNIRNIGKTLVTRTQGTKIASDGLKGRVFEVS
LADLQNDEVAFRKFKLITEDVQGKNCLTNFHGMDLTRDKMCSMVKKWQTMIEAHVDVKTTDGYLLRLFCV
GFTKKRNNQIRKTSYAQHQQVRQIRKKMMEIMTREVQTNDLKEVVNKLIPDSIGKDIEKACQSIYPLHDV
FVRKVKMLKKPKFELGKLMELHGEGGSSGKAAGDETGAKVERADGYEPPVQESV

>sp|Q5T4S7|Modified_Residue:4685
MATSGGEEAAAAAPAPGTPATGADTTPGWEVAVRPLLSASYSAFEMKELPQLVASVIESESEILHHEKQY
EPFYSSFVALSTHYITTVCSLIPRNQLQSVAAACKVLIEFSLLRLENPDEACAVSQKHLILLIKGLCTGC
SRLDRTEIITFTAMMKSAKLPQTVKTLSDVEDQKELASPVSPELRQKEVQMNFLNQLTSVFNPRTVASQP
ISTQTLVEGENDEQSSTDQASAIKTKNVFIAQNVASLQELGGSEKLLRVCLNLPYFLRYINRFQDAVLAN
SFFIMPATVADATAVRNGFHSLVIDVTMALDTLSLPVLEPLNPSRLQDVTVLSLSCLYAGVSVATCMAIL
HVGSAQQVRTGSTSSKEDDYESDAATIVQKCLEIYDMIGQAISSSRRAGGEHYQNFQLLGAWCLLNSLFL
ILNLSPTALADKGKEKDPLAALRVRDILSRTKEGVGSPKLGPGKGHQGFGVLSVILANHAIKLLTSLFQD
LQVEALHKGWETDGPPAALSIMAQSTSIQRIQRLIDSVPLMNLLLTLLSTSYRKACVLQRQRKGSMSSDA
SASTDSNTYYEDDFSSTEEDSSQDDDSEPILGQWFEETISPSKEKAAPPPPPPPPPLESSPRVKSPSKQA
PGEKGNILASRKDPELFLGLASNILNFITSSMLNSRNNFIRNYLSVSLSEHHMATLASIIKEVDKDGLKG
SSDEEFAAALYHFNHSLVTSDLQSPNLQNTLLQQLGVAPFSEGPWPLYIHPQSLSVLSRLLLIWQHKASA
QGDPDVPECLKVWDRFLSTMKQNALQGVVPSETEDLNVEHLQMLLLIFHNFTETGRRAILSLFVQIIQEL
SVNMDAQMRFVPLILARLLLIFDYLLHQYSKAPVYLFEQVQHNLLSPPFGWASGSQDSNSRRATTPLYHG
FKEVEENWSKHFSSDAVPHPRFYCVLSPEASEDDLNRLDSVACDVLFSKLVKYDELYAALTALLAAGSQL
DTVRRKENKNVTALEACALQYYFLILWRILGILPPSKTYINQLSMNSPEMSECDILHTLRWSSRLRISSY
VNWIKDHLIKQGMKAEHASSLLELASTTKCSSVKYDVEIVEEYFARQISSFCSIDCTTILQLHEIPSLQS
IYTLDAAISKVQVSLDEHFSKMAAETDPHKSSEITKNLLPATLQLIDTYASFTRAYLLQNFNEEGTTEKP
SKEKLQGFAAVLAIGSSRCKANTLGPTLVQNLPSSVQTVCESWNNINTNEFPNIGSWRNAFANDTIPSES
YISAVQAAHLGTLCSQSLPLAASLKHTLLSLVRLTGDLIVWSDEMNPPQVIRTLLPLLLESSTESVAEIS
SNSLERILGPAESDEFLARVYEKLITGCYNILANHADPNSGLDESILEECLQYLEKQLESSQARKAMEEF

FSDSGELVQIMMATANENLSAKFCNRVLKFFTKLFQLTEKSPNPSLLHLCGSLAQLACVEPVRLQAWLTR
MTTSPPKDSDQLDVIQENRQLLQLLTTYIVRENSQVGEGVCAVLLGTLTPMATEMLANGDGTGFPELMVV
MATLASAGQGAGHLQLHNAAVDWLSRCKKYLSQKNVVEKLNANVMHGKHVMILECTCHIMSYLADVTNAL
SQSNGQGPSHLSVDGEERAIEVDSDWVEELAVEEEDSQAEDSDEDSLCNKLCTFTITQKEFMNQHWYHCH
TCKMVDGVGVCTVCAKVCHKDHEISYAKYGSFFCDCGAKEDGSCLALVKRTPSSGMSSTMKESAFQSEPR
ISESLVRHASTSSPADKAKVTISDGKVADEEKPKKSSLCRTVEGCREELQNQANFSFAPLVLDMLNFLMD
AIQTNFQQASAVGSSSRAQQALSELHTVEKAVEMTDQLMVPTLGSQEGAFENVRMNYSGDQGQTIRQLIS
AHVLRRVAMCVLSSPHGRRQHLAVSHEKGKITVLQLSALLKQADSSKRKLTLTRLASAPVPFTVLSLTGN
PCKEDYLAVCGLKDCHVLTFSSSGSVSDHLVLHPQLATGNFIIKAVWLPGSQTELAIVTADFVKIYDLCV
DALSPTFYFLLPSSKIRDVTFLFNEEGKNIIVIMSSAGYIYTQLMEEASSAQQGPFYVTNVLEINHEDLK
DSNSQVAGGGVSVYYSHVLQMLFFSYCQGKSFAATISRTTLEVLQLFPINIKSSNGGSKTSPALCQWSEV
MNHPGLVCCVQQTTGVPLVVMVKPDTFLIQEIKTLPAKAKIQDMVAIRHTACNEQQRTTMILLCEDGSLR
IYMANVENTSYWLQPSLQPSSVISIMKPVRKRKTATITTRTSSQVTFPIDFFEHNQQLTDVEFGGNDLLQ
VYNAQQIKHRLNSTGMYVANTKPGGFTIEISNNNSTMVMTGMRIQIGTQAIERAPSYIEIFGRTMQLNLS
RSRWFDFPFTREEALQADKKLNLFIGASVDPAGVTMIDAVKIYGKTKEQFGWPDEPPEEFPSASVSNICP
SNLNQSNGTGDSDSAAPTTTSGTVLERLVVSSLEALESCFAVGPIIEKERNKNAAQELATLLLSLPAPAS
VQQQSKSLLASLHTSRSAYHSHKDQALLSKAVQCLNTSSKEGKDLDPEVFQRLVITARSIAIMRPNNLVH
FTESKLPQMETEGMDEGKEPQKQLEGDCCSFITQLVNHFWKLHASKPKNAFLAPACLPGLTHIEATVNAL
VDIIHGYCTCELDCINTASKIYMQMLLCPDPAVSFSCKQALIRVLRPRNKRRHVTLPSSPRSNTPMGDKD
DDDDDDADEKMQSSGIPNGGHIRQESQEQSEVDHGDFEMVSESMVLETAENVNNGNPSPLEALLAGAEGF
PPMLDIPPDADDETMVELAIALSLQQDQQGSSSSALGLQSLGLSGQAPSSSSLDAGTLSDTTASAPASDD
EGSTAATDGSTLRTSPADHGGSVGSESGGSAVDSVAGEHSVSGRSSAYGDATAEGHPAGPGSVSSSTGAI
STTTGHQEGDGSEGEGEGETEGDVHTSNRLHMVRLMLLERLLQTLPQLRNVGGVRAIPYMQVILMLTTDL
DGEDEKDKGALDNLLSQLIAELGMDKKDVSKKNERSALNEVHLVVMRLLSVFMSRTKSGSKSSICESSSL
ISSATAAALLSSGAVDYCLHVLKSLLEYWKSQQNDEEPVATSQLLKPHTTSSPPDMSPFFLRQYVKGHAA
DVFEAYTQLLTEMVLRLPYQIKKITDTNSRIPPPVFDHSWFYFLSEYLMIQQTPFVRRQVRKLLLFICGS
KEKYRQLRDLHTLDSHVRGIKKLLEEQGIFLRASVVTASSGSALQYDTLISLMEHLKACAEIAAQRTINW
QKFCIKDDSVLYFLLQVSFLVDEGVSPVLLQLLSCALCGSKVLAALAASSGSSSASSSSAPVAASSGQAT
TQSKSSTKKSKKEEKEKEKDGETSGSQEDQLCTALVNQLNKFADKETLIQFLRCFLLESNSSSVRWQAHC
LTLHIYRNSSKSQQELLLDLMWSIWPELPAYGRKAAQFVDLLGYFSLKTPQTEKKLKEYSQKAVEILRTQ
NHILTNHPNSNIYNTLSGLVEFDGYYLESDPCLVCNNPEVPFCYIKLSSIKVDTRYTTTQQVVKLIGSHT
ISKVTVKIGDLKRTKMVRTINLYYNNRTVQAIVELKNKPARWHKAKKVQLTPGQTEVKIDLPLPIVASNL
MIEFADFYENYQASTETLQCPRCSASVPANPGVCGNCGENVYQCHKCRSINYDEKDPFLCNACGFCKYAR
FDFMLYAKPCCAVDPIENEEDRKKAVSNINTLLDKADRVYHQLMGHRPQLENLLCKVNEAAPEKPQDDSG
TAGGISSTSASVNRYILQLAQEYCGDCKNSFDELSKIIQKVFASRKELLEYDLQQREAATKSSRTSVQPT
FTASQYRALSVLGCGHTSSTKCYGCASAVTEHCITLLRALATNPALRHILVSQGLIRELFDYNLRRGAAA
MREEVRQLMCLLTRDNPEATQQMNDLIIGKVSTALKGHWANPDLASSLQYEMLLLTDSISKEDSCWELRL
RCALSLFLMAVNIKTPVVVENITLMCLRILQKLIKPPAPTSKKNKDVPVEALTTVKPYCNEIHAQAQLWL
KRDPKASYDAWKKCLPIRGIDGNGKAPSKSELRHLYLTEKYVWRWKQFLSRRGKRTSPLDLKLGHNNWLR
QVLFTPATQAARQAACTIVEALATIPSRKQQVLDLLTSYLDELSIAGECAAEYLALYQKLITSAHWKVYL
AARGVLPYVGNLITKEIARLLALEEATLSTDLQQGYALKSLTGLLSSFVEVESIKRHFKSRLVGTVLNGY
LCLRKLVVQRTKLIDETQDMLLEMLEDMTTGTESETKAFMAVCIETAKRYNLDDYRTPVFIFERLCSIIY
PEENEVTEFFVTLEKDPQQEDFLQGRMPGNPYSSNEPGIGPLMRDIKNKICQDCDLVALLEDDSGMELLV
NNKIISLDLPVAEVYKKVWCTTNEGEPMRIVYRMRGLLGDATEEFIESLDSTTDEEEDEEEVYKMAGVMA
QCGGLECMLNRLAGIRDFKQGRHLLTVLLKLFSYCVKVKVNRQQLVKLEMNTLNVMLGTLNLALVAEQES
KDSGGAAVAEQVLSIMEIILDESNAEPLSEDKGNLLLTGDKDQLVMLLDQINSTFVRSNPSVLQGLLRII
PYLSFGEVEKMQILVERFKPYCNFDKYDEDHSGDDKVFLDCFCKIAAGIKNNSNGHQLKDLILQKGITQN
ALDYMKKHIPSAKNLDADIWKKFLSRPALPFILRLLRGLAIQHPGTQVLIGTDSIPNLHKLEQVSSDEGI
GTLAENLLEALREHPDVNKKIDAARRETRAEKKRMAMAMRQKALGTLGMTTNEKGQVVTKTALLKQMEEL
IEEPGLTCCICREGYKFQPTKVLGIYTFTKRVALEEMENKPRKQQGYSTVSHFNIVHYDCHLAAVRLARG
REEWESAALQNANTKCNGLLPVWGPHVPESAFATCLARHNTYLQECTGQREPTYQLNIHDIKLLFLRFAM
EQSFSADTGGGGRESNIHLIPYIIHTVLYVLNTTRATSREEKNLQGFLEQPKEKWVESAFEVDGPYYFTV
LALHILPPEQWRATRVEILRRLLVTSQARAVAPGGATRLTDKAVKDYSAYRSSLLFWALVDLIYNMFKKV
PTSNTEGGWSCSLAEYIRHNDMPIYEAADKALKTFQEEFMPVETFSEFLDVAGLLSEITDPESFLKDLLN

SVP

>sp|Q60930|Modified_Residue:32
MAECCVPVCPRPMCIPPPYADLGKAARDIFNKGFGFGLVKLDVKTKSCSGVEFSTSGSSNTDTGKVSGTL
ETKYKWCEYGLTFTEKWNTDNTLGTEIAIEDQICQGLKLTFDTTFSPNTGKKSGKIKSAYKRECINLGCD
VDFDFAGPAIHGSAVFGYEGWLAGYQMTFDSAKSKLTRSNFAVGYRTGDFQLHTNVNNGTEFGGSIYQKV
CEDFDTSVNLAWTSGTNCTRFGIAAKYQLDPTASISAKVNNSSLIGVGYTQTLRPGVKLTLSALVDGKSF
NAGGHKLGLALELEA

>sp|Q60931|Modified_Residue:15,20
MCNTPTYCDLGKAAKDVFNKGYGFGMVKIDLKTKSCSGVEFSTSGHAYTDTGKASGNLETKYKVCNYGLT
FTQKWNTDNTLGTEISWENKLAEGLKLTLDTIFVPNTGKKSGKLKASYRRDCFSLGSNVDIDFSGPTIYG
WAVLAFEGWLAGYQMSFDTAKSKLSQNNFALGYKAADFQLHTHVNDGTEFGGSIYQKVNERIETSINLAW
TAGSNNTRFGIAAKYKLDCRTSLSAKVNNASLIGLGYTQTLRPGVKLTLSALIDGKNFNAGGHKVGLGFE
LEA

>sp|Q60932|Modified_Residue:33
MCSFFLVLLLWQNMAVPPTYADLGKSARDVFTKGYGFGLIKLDLKTKSENGLEFTSSGSANTETTKVNGS
LETKYRWTEYGLTFTEKWNTDNTLGTEITVEDQLARGLKLTFDSSFSPNTGKKNAKIKTGYKREHINLGC
DVDFDIAGPSIRGALVLGYEGWLAGYQMNFETSKSRVTQSNFAVGYKTDEFQLHTNVNDGTEFGGSIYQK
VNKKLETAVNLAWTAGNSNTRFGIAAKYQVDPDACFSAKVNNSSLIGLGYTQTLKPGIKLTLSALLDGKN
VNAGGHKLGLGLEFQA

>sp|Q61387|Modified_Residue:56
MYYKFSSFTQKLAGAWASEAYTPQGLKPVSTEAPPIIFATPTKLTSSVTAYDYSGKNKVPELQKFFQKAD
GFHLKRGLPDQMLYRTTMALTLGGTIYCLIALYMASQPRNK

>sp|Q69ZK0|Modified_Residue:1141
MEAPGSGGGDGGGDPGGDGAHPDARGPVSGPCAAARDSERQLRLRLCVLNEILGTERDYVGTLRFLQSAF
LQRIRQNVADSVEKGLTEENVKVLFSNIEDILEVHKDFLAALEYCLHPEPQSQHELGNVFLKFKDKFCVY
EEYCSNHEKALRLLVELNKVPAVRAFLLSCMLLGGRKTTDIPLEGYLLSPIQRICKYPLLLKELAKRTPG
KHPDHTAVQSALQAMKTVCSNINETKRQMEKLEALEQLQSHIEGWEGSNLTDICTELLLQGNLLKISAGN
IQERAFFLFDNLLVYCKRKSRVTGSKKSTKRTKSINGSLYIFRGRINTEVMEVENVEDGTADYHSNGYTV
TNGWKIHNTAKNKWFVCMAKTAEEKQKWLDALIREREQRESLKLGMERDAYVMIAEKGEKLYHMMMSKKV
NLIKDRRRKLSTVPKCFLGNEFVAWLLEIGEISKTEEGVNLGQALLENGIIHHVSDKHQFKNEQVMYRFR
YDDGTYKARSELEDIMSKGVRLYCRLHSLYAPVIKDRDYHLKTYKSVVPGSKLVDWLLAQGDCQTREEAV
ALGVGLCNNGFMHHVLEKSEFKDESQYFRFHADEEMEGTSSKNKQLRNDFKLVENILAKRLLIPPQEDDY
GFDLEEKNKAVVVKSVQRGSLAEMAGLQAGRKIYSINEDLVFLRPFSEVETILNQFFCSRRPLRLLVATK
AKETIKVPDHPEALSFQIRGTAPPCVFAVGRGSEAVAAGLCAGQCILKVNGTSVANDGALEVLEHFQAFR
NHREEALGLYQWVYHSHEDAQLARASQGAPDEDPQEDDQPDSALPLLSLGPQLSLHEDSAVVSLTLDNVH
LEHGVVYEYMSTAGAKCHVLEKIVEPRGCFRLAAKILEAFAVDDSIFVQNCGRLMAMSSAIVTMSHYEFH
NICDTKLESIGQRIACYQEFAAQLKSRVSPPFKQASLEPHPLCGLDFCPTNCHVNLMEVSYPKTTPSVGR
SFSIRFGRKPSLIGLDPEQGLNPMAYTQHCITTMAAPSWKCSPAVDEDSQGQGLNDSSYGSASGAPSQQD
RGLSFLLKQEDREIQDAYLQLFTKLDVALKEMKQYVTQINRLLSTITEPTSAAPAPCDPSLVEETSSSPP
VSEESEVDRTDHSGIKKVCFKVSEDEQEDSGHDTMSYRDSYSECNSNRDSVLSYTSVRSNSSYLGSDEMG
SGDELPCDMRIPSDKQDKLHGCLEHLFNQVDSIHALLKGPVMSRAFEETRHFPMKHSWQEFKQKEECTVR
GRNLIQISIQEDPWNLPSSIRTLVDNIQQYVEDGKNQLLLALLKCTDTELQLRRDAVFCQALVAAVCTFS
EQLLAALDYRYNNNGEYEESSRDASRKWLEQVAATGVLLHWQSLLAPASVKEERTMLEDIWVTLSELDNV
TFSFKQLDENSVANTNVFYHIEGSRQALKVVFYLDGFHFSRLPSRLEGGASLRLHTVLFTKALESVEGPP
PPGNQAAEELQQEINAQSLEKVQQYYRKLRAFYLERSNLPTDAGATAVKIDQLIRPINALDELYRLMKTF
VHPKAGAAGSLGAGLIPVSSELCYRLGACQITMCGTGMQRSTLSVSLEQAAILARSHGLLPKCVMQATDI
MRKQGPRVEILAKNLRIKDPMPQGAPRLYQLCQPPVDGDL

>sp|Q6P6M7|Modified_Residue:173

MNPESFAAGERRVSPAYVRQGCEARRAHEHLIRLLLEQGKCPEDGWDESTLELFLHELAVMDSNNFLGNC
GVGEREGRVASALVARRHYRFIHGIGRSGDISAVQPKAAGSSLLNKITNSLVLNVIKLAGVHSVASCFVV
PMATGMSLTLCFLTLRHKRPKAKYIIWPRIDQKSCFKSMVTAGFEPVVIENVLEGDELRTDLKAVEAKIQ
ELGPEHILCLHSTTACFAPRVPDRLEELAVICANYDIPHVVNNAYGLQSSKCMHLIQQGARVGRIDAFVQ
SLDKNFMVPVGGAIIAGFNEPFIQDISKMYPGRASASPSLDVLITLLSLGCSGYRKLLKERKEMFVYLST
QLKKLAEAHNERLLQTPHNPISLAMTLKTIDGHHDKAVTQLGSMLFTRQVSGARAVPLGNVQTVSGHTFR
GFMSHADNYPCAYLNAAAAIGMKMQDVDLFIKRLDKCLNIVRKEQTRASVVSGADRNKAEDADIEEMALK
LDDVLGDVGQGPAL

>sp|Q6P8V7|Modified_Residue:47
MLRGGSMTAELGVGFALRAVNERVQQSVARRPRDLPAIQPRLVAVSKTKPADMVIEAYGHGQRTFGENYV
QELLEKASNPKILSSCPEIKWHFIGHLQKQNVNKLMAVPNLSMLETVDSVKLADKVNSSWQKKGPTEPLK
VMVQINTSGEDSK

>sp|Q8BLF2|Modified_Residue:104
MEMYETLGKVGEGSYGTVMKCKHKDTGRIVAIKIFYEKPEKSVNKIATREIKFLKQFRHENLVNLIEVFR
QKKKIHLVFEFIDHTVLDELQHYCHGLESKRLRKYLFQILRAIEYLHNNNIIHRDIKPENILVSQSGITK
LCDFGFARTLAAPGDVYTDYVATRWYRAPELVLKDTSYGKPVDIWALGCMIIEMATGHPFLPSSSDLDLL
HKIVLKVGNLTPHLHNIFSKSPIFAGVVLPQVQHPKTARKKYPKLNGLLADIVHACLQIDPAERTSSTDL
LRHDYFTRDGFIEKFIPELRAKLLQEAKVNSFIKPKENFKENEPVRDEKKSVFTNTLLYGNPSLYGKEVD
RDKRAKELKVRVIKAKGGKGDVPDQKKPEYEGDHRQQGTADDTQPSSLDKKPSVLELTNPLNPSENSDGV
KEDPHAGGCMIMPPINLTSSNLLAANLSSNLSHPNSRLTERTKKRRTSSQTIGQTLSNSRQEDTGPTQVQ
TEKGAFNERTGQNDQISSGNKRKLNFPKCDRKEFHFPELPFTVQAKEMKGMEVKQIKVLKRESKKTDSSK
IPTLLSMDPNQEKQEGGDGDCEGKNLKRNRFFFSR

>sp|Q922Q1|Modified_Residue:66
MGSSSSTALARLGLPGQPRSTWLGVAALGLAAVALGTVAWRRTRPRRRRQLQQVGTVSKVWIYPIKSCKG
VSVCETECTDMGLRCGKVRDRFWMVVKEDGHMVTARQEPRLVLVSITLENNYLTLEAPGMEQIVLPIKLP
SSNKIHNCRLFGLDIKGRDCGDEVAQWFTNYLKTQAYRLVQFDTSMKGRTTKKLYPSESYLQNYEVAYPD
CSPVHLISEASLVDLNTRLKKKVKMEYFRPNIVVSGCEAFEEDTWDELLIGDVEMKRVLSCPRCVLTTVD
PDTGIIDRKEPLETLKSYRLCDPSVKSIYQSSPLFGMYFSVEKLGSLRVGDPVYRMVD

>sp|Q99L47|Modified_Residue:152
MDPRKVSELRAFVKMCRQDPSVLHTEEMRFLREWVESMGGKVPPATHKAKSEENTKEEKRDKTTEENIKT
EELSSEESDLEIDNEGVIEPDTDAPQEMGDENAEITEEMMDEANEKKGAAIEALNDGELQKAIDLFTDAI
KLNPRLAILYAKRASVFVKLQKPNAAIRDCDRAIEINPDSAQPYKWRGKAHRLLGHWEEAAHDLALACKL
DYDEDASAMLREVQPRAQKIAEHRRKYERKREEREIKERIERVKKAREEHERAQREEEARRQSGSQYGSF
PGGFPGGMPGNFPGGMPGMGGAMPGMAGMAGMPGLNEILSDPEVLAAMQDPEVMVAFQDVAQNPSNMSKY
QSNPKVMNLISKLSAKFGGQS

>sp|Q9QWY8|Modified_Residue:675
MRSSASRLSSFSSRDSLWNRMPDQISVSEFIAETTEDYNSPTTSSFTTRLHNCRNTVTLLEEALDQDRTA
LQKVKKSVKAIYNSGQDHVQNEENYAQVLDKFGSNFLSRDNPDLGTAFVKFSTLTKELSTLLKNLLQGLS
HNVIFTLDSLLKGDLKGVKGDLKKPFDKAWKDYETKFTKIEKEKREHAKQHGMIRTEITGAEIAEEMEKE
RRLFQLQMCEYLIKVNEIKTKKGVDLLQNLIKYYHAQCNFFQDGLKTADKLKQYIEKLAADLYNIKQTQD
EEKKQLTALRDLIKSSLQLDPKEVGGLYVASRANSSRRDSQSRQGGYSMHQLQGNKEYGSEKKGFLLKKS
DGIRKVWQRRKCAVKNGILTISHATSNRQPAKLNLLTCQVKPNAEDKKSFDLISHNRTYHFQAEDEQDYI
AWISVLTNSKEEALTMAFRGEQSTGENSLEDLTKAIIEDVQRLPGNDICCDCGSSEPTWLSTNLGILTCI
ECSGIHREMGVHISRIQSLELDKLGTSELLLAKNVGNNSFNDIMEANLPSPSPKPTPSSDMTVRKEYITA
KYVDHRFSRKTCASSSAKLNELLEAIKSRDLLALIQVYAEGVELMEPLLEPGQELGETALHLAVRTADQT
SLHLVDFLVQNCGNLDKQTSVGNTVLHYCSMYGKPECLKLLLRSKPTVDIVNQNGETALDIAKRLKATQC
EDLLSQAKSGKFNPHVHVEYEWNLRQDEMDESDDDLDDKPSPIKKERSPRPQSFCHSSSISPQDKLALPG
FSTPRDKQRLSYGAFTNQIFASTSTDLPTSPTSEAPPLPPRNAGKGPTGPPSTLPLGTQTSSGSSTLSKK
RPPPPPPGHKRTLSDPPSPLPHGPPNKGAIPWGNDVGPLSSSKTANKFEGLSQQASTSSAKTALGPRVLP

KLPQKVALRKTETSHHLSLDRTNIPPETFQKSSQLTELPQKPPLGELPPKPVELAPKPQVGELPPKPGEL
PPKPQLGDLPPKPQLSDLPPKPQMKDLPPKPQLGDLLAKSQAGDVSAKVQPPSEVTQRSHTGDLSPNVQS
RDAIQKQASEDSNDLTPTLPETPVPLPRKINTGKNKVRRVKTIYDCQADNDDELTFIEGEVIIVTGEEDQ
EWWIGHIEGQPERKGVFPVSFVHILSD
