# Supplementary material for: predPhogly-Site: Predicting phosphoglycerylation sites by incorporating probabilistic sequence-coupling information into PseAAC and addressing data imbalance
Source: PLoS One. 2021 Apr 1;16(4):e0249396. doi: 10.1371/journal.pone.0249396 (PMC8016359; doi:10.1371/journal.pone.0249396)
Supplement: Attachment — Submitted filename: Response to Reviewers.docx [file pone.0249396.s006.docx]

08 February 2021

Dear Concern,

I have addressed the points raised by the reviewers and provided a response associated with those points. The manuscript entitled “predPhogly-Site: Predicting phosphoglycerylation sites by incorporating probabilistic sequence-coupling information into PseAAC and addressing data imbalance” is also revised accordingly and also highlighted with a distinct color.

Yours Sincerely,

Sabit Ahmed (on behalf of the authors)

Department of Computer Science and Engineering

Rajshahi University of Engineering and Technology, Bangladesh

E-mail: sabit.a.sirat@gmail.com

**Points raised by the reviewers:**

**Reviewer #1:**

**1.** The authors have addressed majority of the comments. However, a few comments were not addressed properly.

The authors have not provided algorithm with train and test sets so that the method can be replicated in-order to verify the results. The proposed method has achieved very high results and so it would be crucial to check and verify their result through the algorithm they have used.

**Answer:** Thank you very much for your feedback. We have tried addressing the comments which might not be covered in our previous submission. We have updated the flowchart for more insights on the overall procedure of constructing the predPhogly-Site predictor. It is mentioned in the “Introduction” section as well as in the “Dataset” section. Summarizing the steps included in our system, firstly, we have constructed the benchmark dataset from the CPLM database. The phosphoglycerylated proteins, as well as the segmented sequences with respective protein ID and positions, have been provided as File S1. Secondly, we have extracted the sequence-coupling features from the segmented sequences given in File S1. Then we have used 10 times 10-fold cross-validation scheme to train and evaluate our SVM based predictor with the optimal hyperparameters. The step-by-step guidelines are discussed in the “Validation of the proposed model” section and the optimal hyperparameters are given in Table 2. Later, we have performed an independent test where the test dataset contains newly added proteins (available as File S2), completely unknown to the predPhogly-Site predictor for further evaluation. We hope that our system is now reproducible and our results can be checked and verified easily.

**2.** The authors are requested to check the Precision calculation as it seems to be quite low for the other methods in Table 4 when compared to what Bigram-PGK has reported for the methods. The other metrics appear to be similar.

**Answer:** Thanks for your suggestion regarding the precision calculation. We have rechecked the precision calculation and explained this issue properly in Section “Comparative analysis of cross-validation performance” which reflects that the dataset used in the Bigram-PGK study contained only 111 positive samples and 224 negative samples and thus, the false-positive rates of the existing predictors i.e. iPGK-PseAAC, CKSAAPPhoglySite, and Phogly-PseAAC were lower and precision measures were higher. The Bigram-PGK dataset has not reflected the real future test data. Normally, in the future test dataset, the number of positive and negative sites will not be balanced. Moreover, the existing predictors i.e. iPGK-PseAAC, CKSAAPPhoglySite, and Phogly-PseAAC might not handle the real world imbalanced situation of the dataset appropriately. As a result, high false-positive rates and low precision measures were obtained on our benchmark dataset that contains 111 positive instances and 3249 negative instances.

**3.** Also, the references [1]-[5] are unnecessary and do not add any information.

**Answer:** We have removed a few references between [1]-[5] according to your suggestion. However, a few references have been still included as these references could be vital for further researches on phosphoglycerylation sites.

**Reviewer #2:** The authors have appropriately answered my comments. The manuscript structure and content have improved and I support publishing the work.

**Answer:** Thank you very much for your feedback.
