# Supplementary material for: predPhogly-Site: Predicting phosphoglycerylation sites by incorporating probabilistic sequence-coupling information into PseAAC and addressing data imbalance
Source: PLoS One. 2021 Apr 1;16(4):e0249396. doi: 10.1371/journal.pone.0249396 (PMC8016359; doi:10.1371/journal.pone.0249396)
Supplement: Attachment — Submitted filename: Response to Reviewers.docx [file pone.0249396.s007.docx]

08 March 2021

Dear Concern,

I have addressed the points raised by both the journal and reviewers and provided a response associated with those points. The manuscript entitled “predPhogly-Site: Predicting phosphoglycerylation sites by incorporating probabilistic sequence-coupling information into PseAAC and addressing data imbalance” is also revised accordingly and also highlighted with a distinct color.

Yours Sincerely,

Sabit Ahmed (on behalf of the authors)

Department of Computer Science and Engineering

Rajshahi University of Engineering and Technology, Bangladesh

E-mail: sabit.a.sirat@gmail.com

**Journal Requirements:**

Please review your reference list to ensure that it is complete and correct. If you have cited papers that have been retracted, please include the rationale for doing so in the manuscript text, or remove these references and replace them with relevant current references. Any changes to the reference list should be mentioned in the rebuttal letter that accompanies your revised manuscript. If you need to cite a retracted article, indicate the article’s retracted status in the References list and also include a citation and full reference for the retraction notice.

**Answer:** Thanks to the concern very much for the valuable suggestion. I would like to inform you that we have revised and corrected the reference list according to the journal requirements. It is to be included that two of the references had been removed in our previously submitted manuscript according to the suggestion of one of the reviewers. The reviewer indicated that the first five references were irrelevant and unimportant even though there were some vital references in that list. It was not clear why those references seemed so insignificant to the reviewer. However, we had removed only reference numbers three and four as per the request of the reviewer. I would like to include the full citations which had been removed previously as well as in the current revised manuscript.

3. Weissman JD, Raval A, Singer DS. Assay of an intrinsic acetyltransferase activity of the transcriptional coactivator CIITA. In: Methods in enzymology. vol. 370. Elsevier; 2003. p. 378–386.

4. Chou KC. Impacts of bioinformatics to medicinal chemistry. Medicinal chemistry. 2015;11(3):218–234.

**Points raised by the reviewers:**

**Reviewer #1: Major Revision.**

**1.** Previous comments to the authors are not adequately addressed and hence I am summarising it again here.

The authors have stated that the result can be replicated by going through various section of the paper such as ‘Introduction’, ‘Dataset’, ‘Validation of the proposed model’, etc. However, for the review process, the authors are requested to put their dataset and code in a repository from which the reviewers can easily verify the result without the need to go about replicating the result by duplicating their method from scratch.

**Answer:** Thank you for your kind response. We would like to inform you that a git repository containing the source code of predPhogly-Site is available at <https://github.com/Sabit-Ahmed/predPhogly-Site>, which could aid the reviewers in verifying the performance obtained by the predPhogly-Site predictor. All the relevant information i.e. the benchmark dataset, independent test dataset, extracted features, source code are provided either as supporting information or git repository and the results can be reproduced and verified. A web-server is also available at <http://103.99.176.239/predML-Site> for further verification.
